# Rule-based meta-analysis reveals the major role of PB2 in influencing influenza A virus virulence in mice

**DOI:** 10.1186/s12864-019-6295-8

**Published:** 2019-12-24

**Authors:** Fransiskus Xaverius Ivan, Chee Keong Kwoh

**Affiliations:** Biomedical Informatics Lab, School of Computer Science and Engineering, Nanyang Technological University, Singapore, Singapore

**Keywords:** Influenza A virus, Mouse models, Virulence, Proteins, Meta-analysis, Rule-based classification, Random forest

## Abstract

**Background:**

Influenza A virus (IAV) poses threats to human health and life. Many individual studies have been carried out in mice to uncover the viral factors responsible for the virulence of IAV infections. Nonetheless, a single study may not provide enough confident about virulence factors, hence combining several studies for a meta-analysis is desired to provide better views. For this, we documented more than 500 records of IAV infections in mice, whose viral proteins could be retrieved and the mouse lethal dose 50 or alternatively, weight loss and/or survival data, was/were available for virulence classification.

**Results:**

IAV virulence models were learned from various datasets containing aligned IAV proteins and the corresponding two virulence classes (avirulent and virulent) or three virulence classes (low, intermediate and high virulence). Three proven rule-based learning approaches, i.e., OneR, JRip and PART, and additionally random forest were used for modelling. PART models achieved the best performance, with moderate average model accuracies ranged from 65.0 to 84.4% and from 54.0 to 66.6% for the two-class and three-class problems, respectively. PART models were comparable to or even better than random forest models and should be preferred based on the Occam’s razor principle. Interestingly, the average accuracy of the models was improved when host information was taken into account. For model interpretation, we observed that although many sites in HA were highly correlated with virulence, PART models based on sites in PB2 could compete against and were often better than PART models based on sites in HA. Moreover, PART had a high preference to include sites in PB2 when models were learned from datasets containing the concatenated alignments of all IAV proteins. Several sites with a known contribution to virulence were found as the top protein sites, and site pairs that may synergistically influence virulence were also uncovered.

**Conclusion:**

Modelling IAV virulence is a challenging problem. Rule-based models generated using viral proteins are useful for its advantage in interpretation, but only achieve moderate performance. Development of more advanced approaches that learn models from features extracted from both viral and host proteins shall be considered for future works.

## Background

Influenza A virus (IAV) is a member of the family *Orthomyxoviridae* that circulates in humans, mammals and birds. The genome of the virus consists of 8 single-stranded, negative-sense viral RNA segments encoding at least 12 proteins that make up its proteome [1]. Segment 1 encodes for the basic RNA polymerase 2 (PB2); segment 2 encodes for the basic RNA polymerase 1 (PB1) and non-essential PB1-F2 protein; segment 3 encodes for the acidic RNA polymerase (PA) and non-essential PA-X protein; segment 4 encodes for the hemagglutinin (HA) membrane glycoprotein; segment 5 encodes for the nucleocapsid protein (NP); segment 6 encodes for the neuraminidase (NA) membrane glycoprotein; segment 7 encodes for the matrix protein 1 (M1) and matrix protein 2 (M2; also referred to as ion channel protein); and segment 8 encodes for the nonstructural protein 1 (NS1) and nonstructural protein 2 (NS2; also referred to as nuclear export protein).

The HA and NA determine the subtype of IAV. To date, 18 HA (H1-H18) and 11 NA (N1-N11) have been identified. The H1N1, H2N2, and H3N2 subtypes have been responsible for five pandemics of severe human respiratory diseases in the last 100 years, i.e., the 1918 Spanish Influenza (H1N1), 1957 Asian Influenza (H2N2), 1968 Hong Kong (H3N2), 1977 Russian Influenza (H1N1), and 2009 Swine-Origin Influenza (H1N1). The H1N1 and H3N2 subtypes also cause recurrent, seasonal epidemics. In the last few years, the seasonal human IAVs were mainly dominated by the 1968’s H3N2 and 2009’s H1N1 strains. In addition to epidemic and pandemic strains, several IAV subtypes have also infected humans, including the H5N1, H5N6, H6N1, H7N2, H7N3, H7N7, H7N9, H9N2, and H10N8 avian influenza viruses [2, 3]. Among them, the H5N1 and H7N9 subtypes have raised a major public health concern due to their ability to cause outbreaks with high fatality rate (about 60% (www.who.int) and 39% [4], respectively). Overall, IAV poses a threat to human health and life, and therefore further understanding about the virus is needed for a better surveillance and counteractive measures against it.

Many aspects of IAV and the disease it causes have been investigated in mice since the animals are not only cost-effective and easy to handle, but also available in various inbred, transgenic, and knockout strains. Moreover, the genomes of various inbred mice have been recently available. Mice have also allowed us to uncover host and viral molecular determinants of IAV virulence. Early outcome of IAV study in mice was the revelation of the protective role of interferon-induced gene Mx1 against the virus [5]. Recently, the gene has been shown to inhibit the assembly of functional viral ribonucleoprotein complex of IAV [6]. In the last 50 years, the importance of many more host genes in influenza pathogenesis has been discovered through experiments in mice, including RIG-I, IFITM3, TNF and IL-1R genes (reviewed in [7, 8]). Nonetheless, one limitation of the existing approaches in investigating host molecular determinants involved in IAV virulence is that it has not yet taken into account the contribution of allelic variation to differential host responses.

In contrast, the influence of variations in viral genes to IAV virulence have been investigated in a number of ways. These included the generation of mouse-adapted IAVs through serial lung-to-lung passaging and recombinant IAVs harboring specific mutations using plasmid-based reverse genetic techniques combined with mutagenesis approaches. The application of these techniques has provided various insights about viral mutations involved in IAV virulence. For example, the increased virulence of IAV during its adaptation in mice has been associated with mutations in the region 190-helix, 220-loop and 130-loop, which surround the receptor-binding site in the HA protein (reviewed in [9]). Mutations in PB2 have also been considered to play a significant role in the increased IAV virulence in mice, which include mutations E627K and D701N that are considered as general markers for IAV virulence in mice [7]. Interestingly, a single mutation N66S in the accessory protein PB1-F2 could also contribute to increased virulence [10]. Mutations in multiple sites of a specific viral protein and mutations in multiple genes have also been shown to have a synergistic effect on IAV virulence in mice. For example, synergistic effect of dual mutations S224P and N383D in PA led to increased polymerase activity and has been considered as a hallmark for natural adaptation of H1N1 and H5N1 viruses to mammals [11]. Another example is the synergistic action of two mutations D222G and K163E in HA and one mutation F35 L in PA of pandemic 2009 influenza H1N1 virus that causes lethality in the infected mice [12]. Furthermore, virulence may not only be encoded at protein level, but also at nucleotide and post-translational level. In a very recent study, synonymous codons were interestingly able to give rise different virulence levels [13]. On the other hand, the HA N-linked glycosylation is known to affect viral virulence by impacting the host immune response (reviewed in [14]).

The confidence of contribution of viral protein sites to the virulence of influenza infections could be better investigated through a meta-analysis approach, which is a systematic amalgamation of results from individual studies. Such approach, to our knowledge, has only been carried out using a Bayesian graphical model to investigate the viral protein sites important for virulence of influenza H5N1 in mammals [15]. Nevertheless, a meta-analysis approach using Naive Bayes approach at viral nucleotide level has recently been carried out to demonstrate the contribution of synonymous nucleotide mutations to IAV virulence [13]. In this paper we present a meta-analysis of viral protein sites that determine the virulence of infections with any subtype of IAV; however, instead of any mammal, we focus on the infections in mice. Our meta-analysis approach utilized rule-based machine learnings and random forest to predict IAV virulence from datasets we created. The creation of the datasets involved: (*i*) documentation of the virulence of infections involving particular IAV and mouse strains, (*ii*) classification of virulence levels, and (*iii*) collection and alignments of the corresponding IAV protein sequences. For learning IAV virulence models, each column of the alignments was considered as a feature vector and the virulence levels as a target vector. When host information was considered, the amino acids in the columns were tagged with a symbol representing the corresponding mouse strain. The models were developed using either all records in the datasets or records for a specific mouse strain or influenza subtype, and using the concatenated alignments of all IAV proteins or individual alignment of PB2, PB1, PA, HA, NP, NA, M1, NS1, PB1-F2, PA-X, M2, or NS2 proteins. Top protein sites and synergy between protein sites were then examined for some biological interpretations.

## Results

### Datasets for modelling IAV virulence

**Fig. 1:**
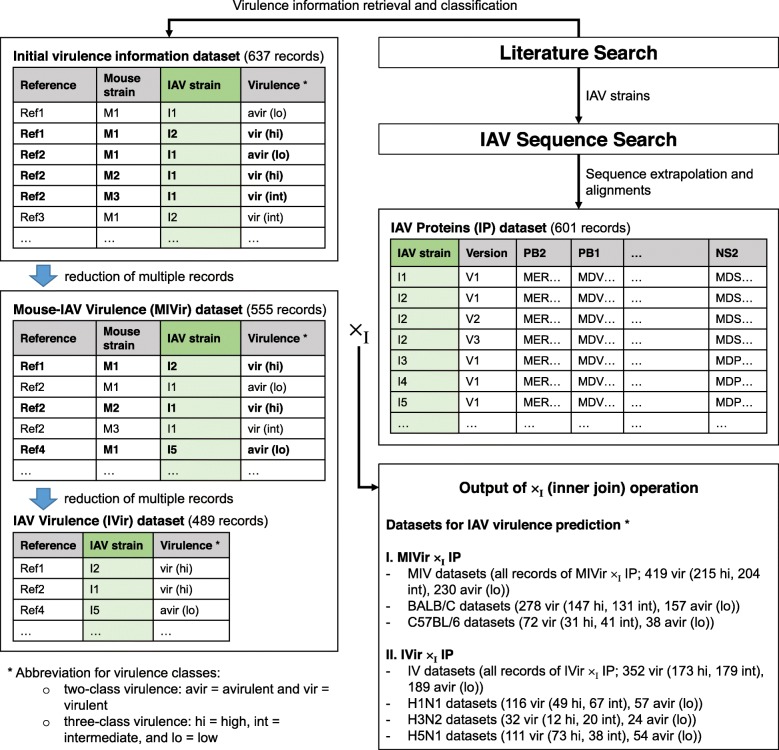
Creation of benchmark datasets for IAV virulence prediction. The dataset containing initial virulence information can be found in Table S1 (Additional file 5), while the Mouse-IAV Virulence (MVir) and IAV Virulence (IVir) datasets can be found in Table S2 and S3 (Additional files 6 and **7**), respectively

The steps in creating benchmark datasets for modeling IAV virulence is summarized in Fig. 1. Initially, a dataset containing 637 records of IAV infections in mice – of which the full or incomplete genomes of the IAVs could be retrieved from public sequence databases and the virulence class of the infection could be identified - was created according to information available in 84 journal publications (Additional file 5: Table S1). Of those records, 502 records have their MLD50 provided in the literature. Following RULE 6 (see Methods), multiple records involving specific IAV and mouse strain were reduced into a single record (Additional file 6: Table S2). This produced a new dataset containing 555 records and named as the Mouse-IAV Virulence (MIVir) dataset. Using the same rule, the MIVir dataset was further reduced to a dataset containing 489 records of IAV virulence across different mouse strains and named as the IAV Virulence (IVir) dataset (Additional file 7: Table S3).

The MIVir and IVir datasets were then inner joined with another dataset containing the 12 IAV proteins whose amino acids in their aligned position (named as the IAV Proteins (IP) dataset), producing the MIVir ×_I_ IP and IVir ×_I_ IP datasets, respectively. The keys for joining the dataset were the IAV strains listed in the MIVir or IVir dataset. Once again, note that some virus strains were represented by multiple records in the IP dataset and some proteins were generated from extrapolated genomes. The breakdowns of the two joined datasets are shown in Fig. 1, and a more detailed breakdown of the MIVir ×_I_ IP is shown in Table 1. As shown in the figure and table, the final datasets were mainly dominated by experiments involving BALB/C and C57BL/6 mice and H1N1, H3N2 and H5N1 viruses. Much fewer 129S1/SvImJ, 129S1/SvPasCrlVr, A/J, C3H, CAST/EiJ, CBA/J, CD-1, DBA/2, FVB/NJ, ICR, NOD/ShiLtJ, NZO/HILtJ, PWK/PhJ, SJL/JOrlCrl, and WSB/EiJ mice and H1N2, H3N8, H5N2, H5N5, H5N6, H5N8, H6N1, H7N1, H7N2, H7N3, H7N7, H7N9 and H9N2 viruses were in the datasets. Subsets of the MIVir ×_I_ IP dataset used in this study included the dataset containing all records (named as the MIV dataset) and datasets containing records of infections in BALB/C and C57BL/6 mice (the BALB/C and C57BL/6 datasets, respectively); while subsets of the IVir ×_I_ IP dataset used in this study included the dataset containing all records (the IV dataset) and datasets containing infections with H1N1, H3N2 and H5N1 viruses (the H1N1, H3N2 and H5N1 datasets, respectively). For virulence modelling, we further considered the subsets of the MIV, IV, BALB/C, C57BL/6, H1N1, H3N2 and H5N1 datasets, whether they contained the concatenated IAV protein alignments or individual alignment of PB2, PB1, PA, HA, NP, NA, M1, NS1, PB1-F2, PA-X, M2 or NS2 proteins.

**Table 1:** Cross-tabulation between mouse strains and IAV subtypes in the MIVir ×_I_ IP (MIV) dataset. The number at the top in each cell corresponds to the number of records of relevant infections, and its breakdown into high, intermediate and low virulence cases for the three-class classification problems are shown in order in parenthesis. The number of virulent cases for the two-class classification problems is the sum of the number of high and intermediate virulence cases, while the number of avirulent cases equals to the number of low virulence cases

Mouse strain	IAV subtype				
	H1N1	H3N2	H5N1	Others	Total
BALB/C	123 (35/40/48)	14 (4/2/8)	162 (69/40/53)	136 (39/49/48)	435 (147/131/157)
C57BL/6	61 (14/34/13)	17 (1/2/14)	6 (6/0/0)	26 (10/5/11)	110 (31/41/38)
CD-1	0 (0/0/0)	34 (5/16/13)	0 (0/0/0)	0 (0/0/0)	34 (5/16/13)
DBA/2	21 (14/5/2)	15 (2/5/8)	0 (0/0/0)	6 (2/2/2)	42 (18/12/12)
Others	19 (9/3/7)	7 (5/0/2)	1 (0/0/1)	1 (0/1/0)	28 (14/4/10)
Total	224 (72/82/70)	87 (17/25/45)	169 (75/40/54)	169 (51/57/61)	649 (215/204/230)

### Visualization of IV dataset

**Fig. 2:**
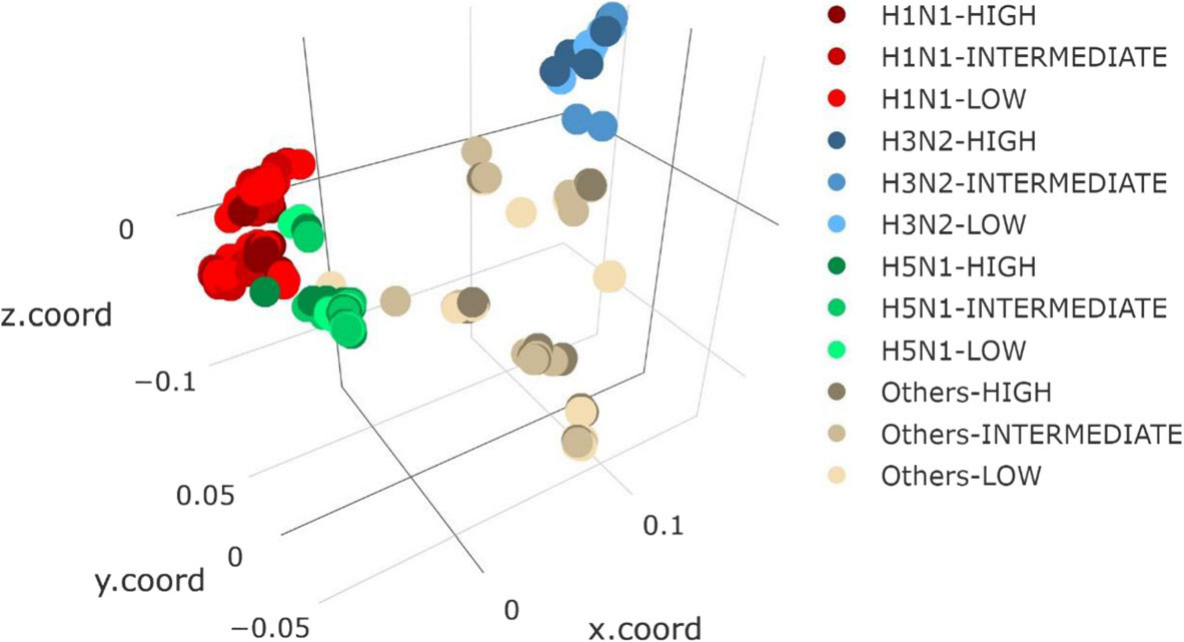
Three-dimensional multidimensional scaling plot of the concatenated alignments of all IAV proteins. Each data point, which represents a record of concatenated aligned proteins of a particular IAV strain, is colored based on the subtype and three-class virulence label

For an initial view of the IAV sequences being used for virulence prediction, the 3D multidimensional scaling plot that visualizes the level of similarity between the concatenated alignments of all IAV proteins in the IV dataset is presented in Fig. 2. While the clusters of dominant IAV subtypes can be easily observed in the plot, separation between virulence classes is lack and this illustrates the challenge in the prediction.

**Fig. 3:**
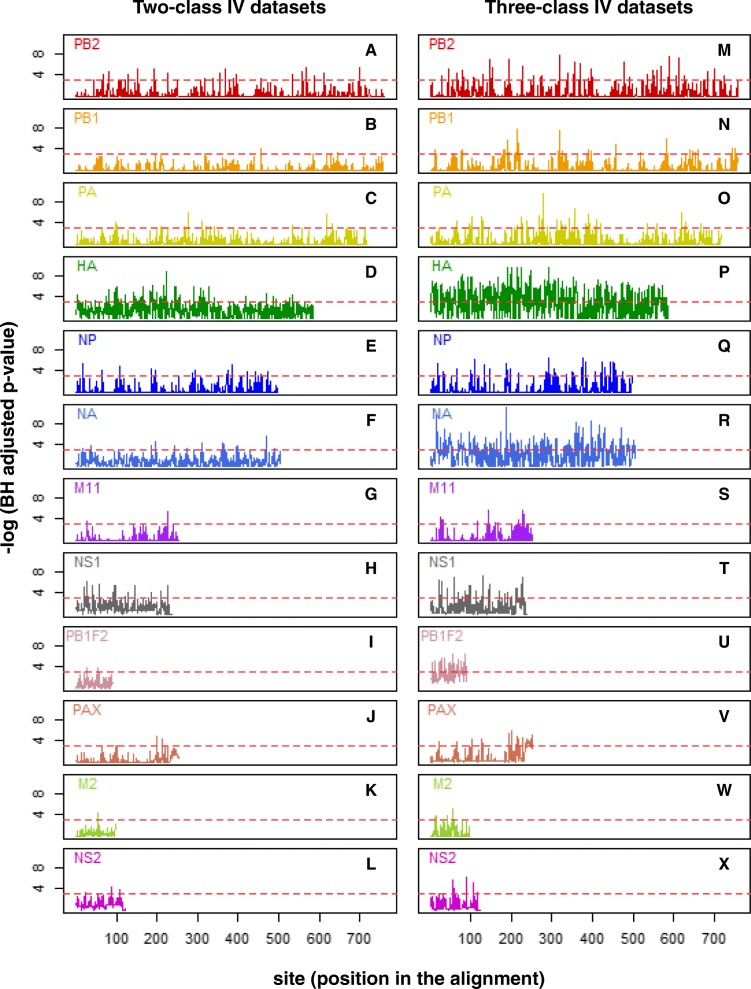
Line plots showing the correlations between sites in the IAV protein alignments and IAV virulence class in the two-class (on the left; subplots A-L) and three-class (on the right; subplots M-X) IV datasets. The correlations are measured using the negative log of the Benjamini-Hochberg (BH) adjusted *p*-values of the chi-square tests for independence between sites and IAV virulence. The red dashed horizontal line in each plot indicates the critical adjusted p-value based on the significance level of 0.05

In addition, the correlation between each site and the target virulence class in the IV dataset was also measured using the Benjamini-Hochberg (BH) adjusted *p*-value of the chi-square test of independence. The line plots showing the –log (BH adjusted p-value) over the alignment sites of each IAV protein for the two-class and three-class datasets are given in Fig. 3. Overall, HA had many more sites that had a significant correlation with the target virulence (BH adjusted p-value < 0.05), i.e., 72 and 283 sites for the two-class and three-class datasets, respectively. On the other hand, M2 had the least numbers of significant sites, i.e., 1 and 4 for the two-class and three-class datasets, respectively. The numbers of significant sites for other proteins and for the two-class and three-class datasets, respectively, are as follows: 26 and 44 for PB2, 6 and 30 for PB1, 14 and 33 for PA, 19 and 40 for NP, 19 and 167 for NA, 4 and 10 for M1, 18 and 32 for NS1, 3 and 30 for PB1-F2, 6 and 26 for PA-X, and 3 and 5 for NS2. Interestingly, while PB2, PA, NP, M1, NS1 and NS2 had their number of significant sites for the three-class dataset about twice the number of significant sites for the two-class dataset, the PB1, HA, NA, PB1-F2 and PA-X had a much higher fold increase in the number of significant sites.

### Performance of rule-based models for IAV virulence

Here we focus on the application of OneR, JRip and PART algorithms for developing rule-based models for IAV virulence from various datasets we created. Examples of the virulence models generated using the machine learning algorithms for the two-class and three-class MIV, IV, BALB/C, C57BL/6, H1N1, H3N2 and H5N1 datasets containing the concatenated protein alignments are provided in Tables S9-S15 (Additional files 13, 14, 15, 16, 17, 18 and 19), respectively. For each of the two-class and three-class datasets, containing either the concatenated protein alignments or individual protein alignment, 100 virulence models were generated for performance evaluation in this section and model characterization in the next section. Specifically, a three-way ANOVA (with interactions) model was built for each two-class and three-class dataset collection to evaluate the difference in accuracy between models. It revealed that the accuracy of the virulence models in both collections were influenced by the dataset, protein alignment, machine learning algorithm, as well as interactions among them. Following this, the Tukey’s HSD post hoc tests for multiple comparisons between pairs of models were carried out and some results are discussed here.

Table 2 highlights the performance of OneR, JRip and PART on the two-class and three-class datasets containing the concatenated IAV protein alignments. Overall, in terms of their average accuracy, precision and recall, PART models always outperformed OneR and JRip, while JRip were almost always better than OneR (the only case OneR consistently outperformed JRip was on the three-class H3N2 classification). However, statistical significant differences were mainly observed between PART and OneR/JRip models, and less frequently observed between OneR and JRip models mentioned (please inspect (Additional file 3: Figure S3) for MIV and IV and (Additional file 4: Figure S4) for BALB/C, C57BL/6, H1N1, H3N2 and H5N1). Nonetheless, PART had many more rules compared to JRip and OneR. For example, PART had on average 10.67 and 46.97 rules per model for the two-class and three-class IV dataset, respectively; while JRip had on average 3.89 and 4.55 rules, respectively, and OneR always had 1 rule.

**Table 2:** Average accuracy, precision and recall (standard deviations in parantheses) of the 100 OneR (1R), JRip (JR) on PART (PT) models learned independently from the two-class and three-class MIV, IV, BALB/C, C57BL/6, H1N1, H3N2 and H5N1 datasets containing the concatenated alignments of all IAV proteins

	Accuracy (%)			Precision (%)			Recall (%)		
	1R	JR	PT	1R	JR	PT	1R	JR	PT
Two-class datasets									
MIV	58.6 (3.6)	58.8 (5.9)	**71.8** (3.8)	59.1 (3.8)	59.9 (6.8)	**72.2** (3.8)	58.6 (3.6)	58.8 (5.9)	**71.8** (3.8)
IV	55.2 (4.0)	60.4 (6.1)	**72.4** (4.0)	55.8 (4.4)	61.2 (6.5)	**72.8** (4.1)	55.2 (4.0)	60.4 (6.1)	**72.4** (4.0)
BALB/C	54.6 (3.8)	57.5 (5.5)	**70.6** (4.8)	55.1 (4.3)	58.3 (6.4)	**71.0** (4.9)	54.6 (3.8)	57.5 (5.5)	**70.6** (4.8)
C57BL/6	70.7 (7.9)	73.4 (7.4)	**74.3** (7.1)	72.6 (8.6)	75.0 (7.5)	**75.4** (7.1)	70.7 (7.9)	73.4 (7.4)	**74.3** (7.1)
H1N1	58.7 (6.0)	59.2 (6.3)	**65.0** (7.5)	61.8 (8.0)	61.9 (8.1)	**65.8** (7.6)	58.7 (6.0)	59.2 (6.3)	**65.0** (7.5)
H3N2	72.1 (9.2)	80.7 (11.5)	**84.4** (8.4)	79.4 (8.8)	84.1 (9.7)	**86.5** (7.4)	72.1 (9.2)	80.7 (11.5)	**84.4** (8.4)
H5N1	57.3 (6.4)	64.9 (8.1)	**72.4** (6.9)	62.1 (10.6)	67.2 (8.8)	**73.3** (7.3)	57.3 (6.4)	64.9 (8.1)	**72.4** (6.9)
Three-class datasets									
MIV	45.7 (2.6)	44.5 (3.4)	**60.2** (3.0)	46.6 (3.1)	52.8 (5.3)	**60.3** (2.9)	45.7 (2.6)	44.5 (3.4)	**60.2** (3.0)
IV	42.1 (3.2)	42.5 (3.3)	**56.3** (3.5)	43.4 (4.4)	47.9 (6.5)	**56.6** (3.5)	42.1 (3.2)	42.5 (3.3)	**56.3** (3.5)
BALB/C	39.8 (3.5)	42.1 (4.2)	**55.4** (3.5)	40.7 (4.8)	49.1 (6.9)	**55.5** (3.5)	39.8 (3.5)	42.1 (4.2)	**55.4** (3.5)
C57BL/6	60.4 (5.8)	61.9 (7.2)	**66.6** (7.5)	65.6 (7.6)	66.3 (7.1)	**68.6** (7.8)	60.4 (5.8)	61.9 (7.2)	**66.6** (7.5)
H1N1	43.3 (5.0)	44.0 (7.1)	**54.6** (6.6)	48.4 (8.2)	50.3 (9.7)	**55.5** (7.0)	43.3 (5.0)	44.0 (7.1)	**54.6** (6.6)
H3N2	47.9 (8.9)	43.0 (9.5)	**60.9** (11.7)	61.4 (17.1)	59.3 (14.6)	**64.4** (13.6)	47.9 (8.9)	43.0 (9.5)	**60.9** (11.7)
H5N1	38.0 (5.8)	42.1 (6.9)	**54.0** (7.5)	39.7 (8.6)	47.6 (10.6)	**55.1** (7.8)	38.0 (5.8)	42.1 (6.9)	**54.0** (7.5)

Table 2 also shows that incorporating host information improved the accuracy of the three-class virulence classification but not for the two-class virulence classification – the average accuracies of PART models on the three-class MIV and IV datasets were 60.2 and 56.3% (Tukey’s HSD adjusted *p*-value for the difference was < 0.05), respectively, but they were about the same for the two-class virulence classification, i.e., 71.8% for MIV dataset and 72.4% for IV dataset (Tukey’s HSD adjusted p-value for the difference was close to 1). Furthermore, when consindering the host strains, the rule-based models were more accurate for the C57BL/6 datasets than the BALB/C datasets (statistically significant (Tukey’s HSD adjusted p-value < 0.05) for the three-class problem but not two-class problem); and when considering the IAV subtypes, the rule-based models were more accurate for the H3N2 datasets than the H1N1 and H5N1 datasets (statistically significant for all cases). However, it ought to be noted that the standard deviations for the C57BL/6 and H3N2 datasets were higher than the rest, and that aggregating all mouse and/or virus strains gave the smallest standard deviation while keeping accuracy competitive.

**Fig. 4:**
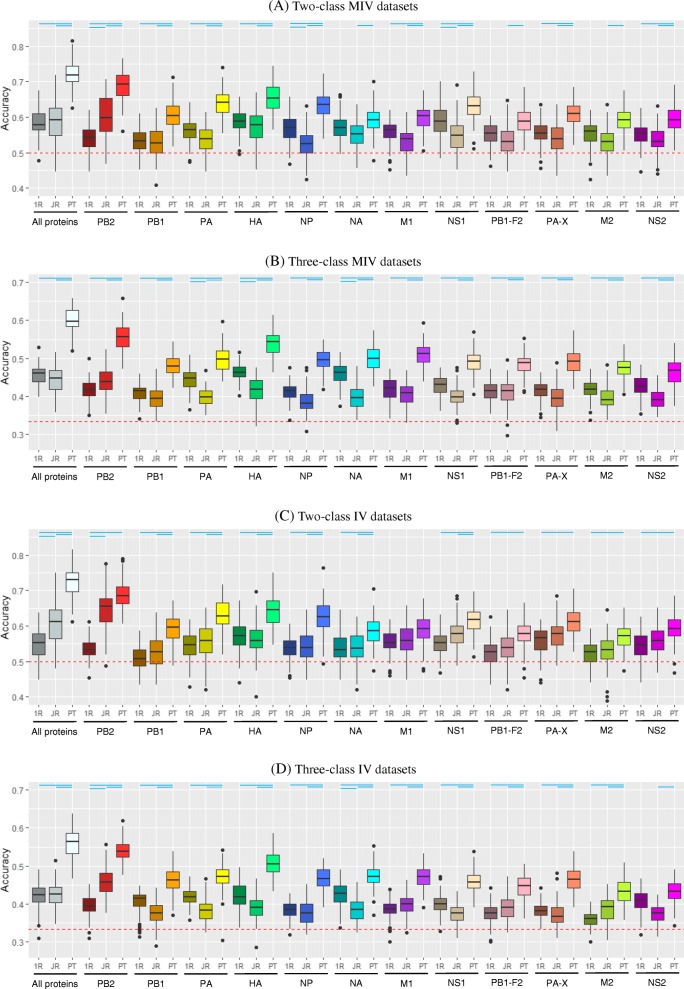
Accuracy distribution of 100 models learned independently from the two-class and three-class MIV (A and B, respectively) and IV (C and D, respectively) datasets using OneR (1R), JRip (JR) and PART (PT). The datasets contain either the concatenated alignments of all IAV proteins or individual alignment of PB2, PB1, PA, HA, NP, NA, M1, NS1, PB1-F2, PA-X, M2 or NS2 proteins. The red dashed horizontal line indicates the accuracy of zero rule learner, while the blue horizontal lines indicate significant difference (Tukey’s HSD adjusted p-value < 0.05) between two virulence models generated from the same protein alignment. Information about significant differences between all possible pairs of virulence models in each plot can be found in Figure S3 (Additional file 3)

The distributions of the accuracies of the 100 OneR/JRip/PART models learned from the two-class and three-class MIV and IV datasets containing either the concatenated protein alignments or an individual protein alignment are shown in Fig. 4 and those learned from the BALB/C, C57BL/6, H1N1, H3N2 and H5N1 datasets are shown in (Additional file 1: Figure S1). The results of the Tukey’s HSD post hoc test for multiple comparisons between pairs of models that appear in each plot in Fig. 4 and Additional file 1: Figure S1 are given in Figures S3 and S4 (Additional files 3 and 4), respectively. Once again, PART usually outperformed OneR and JRip, but it was not unusual that OneR outperformed JRip. Of interest, PART models that were built on the datasets containing the concatenated protein alignments almost always achieved the highest average accuracy, except for the three-class H3N2. The average accuracy was usually significantly higher than the accuracy of other competing models. In many cases, PART model that is based on PB2 or HA alignment could compete against PART model that is based on the concatenated protein alignments (no significant difference between their average accuracy; see Figure S3 and S4 (Additional files 3 and 4)).

Finally, we noted that RF models did not outperform PART models. In about 50% of the cases, PART even gave significantly better accuracies than RF (see (Additional file 2: Figure S2)). Nonetheless, the site importance ranking output by RF could provide valuable insights and hence, RF models were further explored.

### Top sites and synergy between sites for IAV virulence

As the performance of the models generated by a specific learning algorithm varied from one independent learning to another, the models themselves tended to vary a lot. This demonstrated the influence of selected training data. Hence, rather than inspecting the model one by one, it is more interesting to investigate individual sites that were frequently included in learned models or considered to have more impacts in the models. For this, the OneR’s single site model and RF’s site importance ranking naturally suit the purpose. For JRip and PART, we calculated the average contribution of each site to the accuracy of learned models. Table 3 summarizes the sites selected by OneR (ordered by their frequency; sites that were selected once are not shown), top 20 sites by JRip and PART (ordered by their average contribution to the accuracy of learned models), and top 20 influential sites by RF (ordered by the average mean decrease in accuracy) following 100 independent learnings from the two-class and three-class IV datasets containing the concatenated protein alignments.

**Table 3:** Top sites for modelling IAV virulence based on the 100 models generated from the (A) two-class and (B) three-class IV datasets containing the concatenated aligments of all IAV proteins. For OneR (1R), the numbers in parentheses are the frequency of the corresponding site being selected in the models; for JRip (JR) and PART (PT), they are the average contribution of the corresponding site to accuracy (in percent); and for random forest (RF), they are the average mean decrease in accuracy attributed to the corresponding site. Each number was calculated following 100 independent learnings from the two-class or three-class IV dataset. For 1R, only sites with frequency > 1 are shown, while for JR, PT and RF, only top 20 sites are shown

(A) Two-class IV dataset					
1R	HA-142 (28)	HA-188 (12)	HA-160 (7)	NA-46 (6)	HA-189 (4)
	PA-X-213 (4)	HA-219 (3)	HA-285 (3)	HA-397 (3)	NA-79 (3)
	NS1–171 (3)	NS1–95 (3)	HA-196 (2)	NA-86 (2)	NS1–226 (2)
JR	PB2–627 (4.07)	PB2–701 (3.03)	PA-97 (1.40)	HA-297 (1.26)	HA-452 (0.96)
	HA-218 (0.91)	NA-46 (0.89)	M1–227 (0.89)	NA-17 (0.71)	NA-164a (0.58)
	NS1–95 (0.55)	NS1–226 (0.53)	M1–15 (0.52)	NS1–171 (0.51)	PB2–508 (0.48)
	NA-151 (0.43)	PA-X-207 (0.43)	NA-29 (0.42)	NA-371 (0.40)	HA-278 (0.39)
PT	NS1–42 (20.29)	PA-97 (20.20)	PB2–714 (18.28)	PB2–110 (16.72)	PB2–153 (13.26)
	PB2–701 (11.53)	NA-276 (10.35)	NP-101 (10.19)	PA-556 (9.94)	PB2–318 (9.26)
	NP-492 (9.16)	NP-133 (8.92)	PB2–80 (8.71)	M1–215 (8.20)	NS1–123 (7.58)
	HA-485 (7.56)	PA-341 (6.67)	PB2–635 (6.23)	PB2–158 (6.08)	PB2–627 (5.83)
RF	PA-97 (6.75)	PB2–701 (6.54)	PA-X-97 (6.25)	NS1–42 (5.87)	HA-218 (5.53)
	PB2–355 (5.11)	NP-34 (4.83)	PB2–627 (4.76)	PB2–714 (4.55)	HA-186 (4.12)
	HA-227 (3.88)	NP-101 (3.78)	PB2–699 (3.68)	HA-485 (3.66)	PB2–318 (3.62)
	HA-142 (3.52)	M1–30 (3.49)	PB2–675 (3.46)	PB2–153 (3.43)	NA-46 (3.35)
(B) Three-class IV dataset					
1R	HA-188 (34)	NA-370 (16)	NA-16 (10)	HA-142 (9)	HA-53 (6)
	HA-94 (4)	NA-164a (4)	HA-8 (3)	HA-173 (2)	HA-285 (2)
JR	PB2–627 (4.98)	PB2–701 (1.73)	NA-151 (1.45)	NA-164a (1.37)	HA-218 (1.20)
	HA-297 (1.02)	HA-225 (0.94)	HA-452 (0.93)	PB1-F2–28 (0.88)	HA-327b (0.85)
	M2–28 (0.84)	HA-266 (0.74)	NS1–42 (0.71)	PA-97 (0.68)	NA-61 (0.68)
	PA-X-213 (0.59)	HA-482 (0.58)	M2–93 (0.54)	HA-160 (0.52)	PB1-F2–49 (0.51)
PT	PB2–158 (12.81)	PB2–110 (11.97)	NS1–42 (10.79)	PB2–153 (10.56)	NA-276 (10.31)
	PB2–80 (9.21)	NS2–67 (8.46)	PB2–265 (8.23)	PB2–66 (7.92)	PB2–627 (7.62)
	NA-441 (7.28)	NS1–28 (6.97)	M2–24 (6.87)	PB2–497 (6.54)	HA-294 (6.51)
	PB1–578 (6.20)	PA-97 (6.19)	NP-101 (6.18)	PB2–76 (6.07)	M1–215 (6.06)
RF	PB2–627 (6. 69)	NS1–42 (6.49)	HA-225 (6.41)	PB2–701 (6.34)	PA-97 (5.90)
	HA-218 (5.42)	PB2–355 (5.41)	PA-X-97 (5.26)	M1–215 (4.84)	PB2–699 (4.52)
	NP-133 (4.51)	NP-101 (4.48)	PB2–153 (4.41)	M1–30 (4.35)	NP-34 (4.31)
	HA-227 (4.22)	HA-156 (4.17)	PB2–714 (4.12)	HA-188 (4.12)	NA-49 (4.10)

Overall, for the top sites in Table 3, OneR and JRip preferred sites in HA and NA, PART had a high preference towards sites in PB2, and RF pointed out more sites in PB2 and HA were important. In terms of their consistency in selecting sites for the two-class and three-class virulence models, RF was the most consistent (15 shared sites), followed by PART (10 shared sites), JRip (8 shared sites) and finally OneR (only 4 sites). Furthermore, no site was shared by all four learners for either the two-class or three-class virulence models; but there were few sites shared by three learners: PB2–627, PB2–701, PA-97 and NA-46 for the two-class models, and PB2–627, PA-97 and NS1–42 for the three-class models.

**Fig. 5:**
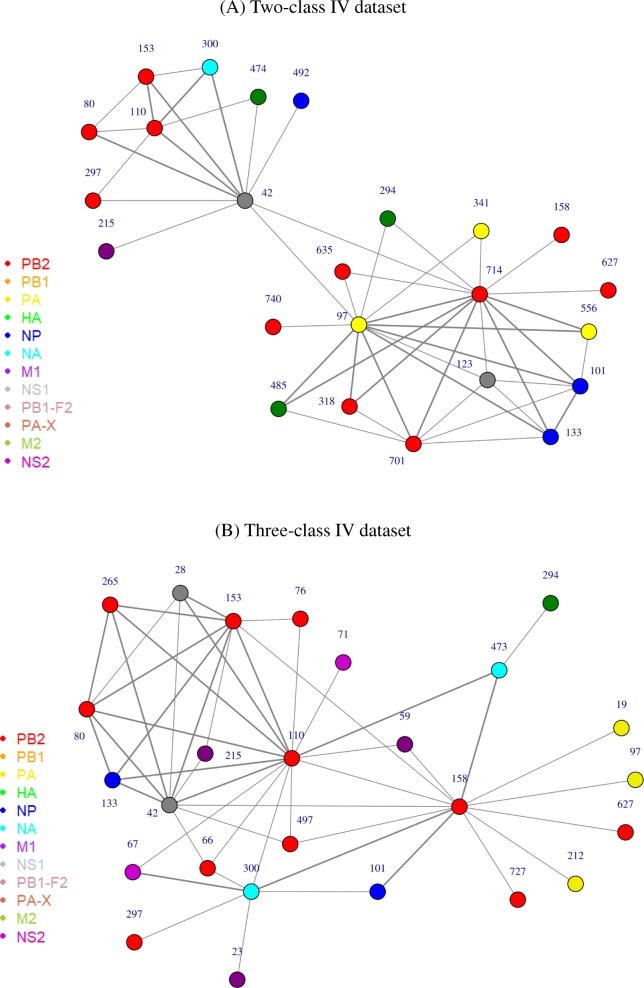
Synergistic graphs between IAV protein sites in determining virulence based on 100 PART models learned from the (A) two-class and (B) three-class IV datasets containing the concatenated alignments of all IAV proteins. Each node in the graphs represents an IAV protein site – the type of the protein is encoded by color and the site number is written above the node. Two sites are connected by an edge if they appear in the top 50 site pairs contributing to the accuracy of the corresponding PART models. The thickness of an edge indicates the level of contribution of the corresponding site pair to the accuracy

In addition to analyzing individual sites, it is also interesting to investigate the synergy between sites that determine IAV virulence. The rule-based models given by JRip and PART serve this purpose, but here we limit to PART models that usually gave the highest average accuracy. For this, in similar way to the identification of top individual sites, we extracted the average contribution of each pair of sites appearing in each rule in PART models to the overall accuracy. The synergistic networks arising from top 50 site pairs in PART models learned from the two-class and three-class IV datasets containing concatenated protein alignments are shown in Fig. 5A and B, respectively. As shown, the sites in both cases were interestingly fully connected and mainly involved sites in PB2. Top 4 sites that had high degree (number of connections) for the two-class virulence models included PB2–714 (degree = 14), PA-97 (13), NS1–42 (10) and PB2–701 (7), and interestingly, the pairing between top two sites PB2–714 and PA-97 had the highest contribution to accuracy. On the other hand, sites that had high degree for the three-class virulence models included PB2–110 (15), PB2–158 (13), NS1–42 (10) and PB2–153 (9), and the pairing between PB2–153 and NS1–42 had the highest contribution to accuracy.

## Discussion

In this influenza study, we systematically and extensively searched literature, collected infection records involving specific mouse and IAV strains, noted their virulence, classified the virulence level, and obtained related IAV proteins in order to develop predictive virulence models of IAV infections. Furthermore, we proposed a number of procedures to tackle various missing data. For virulence, the MLD50 value is the ultimate information we looked for; but in its absence, weight loss and/or survival data of infected mice were utilized to infer the lower or upper bound of MLD50 and subsequently, to label the virulence class. For IAV genomes, when the genomes were incomplete or contained partial sequences, extrapolation was performed using the closest genome relative identified with BLAST. These pre-processing works were done manually and ambiguity occasionally occurred. Hence, caution must be taken when dealing with the datasets and improvement in the pre-processing approach may be considered for future works. Alternatively, efforts in improving the current practice of storing IAV virulence information by research community such that it eases its reusability ought to be encouraged, e.g., by creating an online database that accepts submissions of IAV virulence related data and is able to generate high quality tables or figures of the input data (which then can be added into related manuscript).

Despite the limitations of the datasets due to the ways in handling missing MLD50, partial sequences and incomplete genomes, and also a recent critic of using LD50 as a virulence measure [16], the models learned from the datasets could provide insights about IAV virulence across mouse and virus strains. Rule-based models were chosen since their output can be easily interpreted and are congruent with the current practice in investigating IAV virulence experimentally. Three rule-based learning approaches were employed: OneR, JRip and PART. OneR approach outputs a single site model that gives the highest accuracy [17]; JRip and PART considers multiple sites and they construct a set of decision rules using different strategy. While JRip mainly uses separate-and-conquer algorithms [18], PART combines separate-and-conquer strategy and partial decision trees [19]. For a comparison in the performance, we also explored the RF approach [20] in modelling IAV virulence.

For the models and their performance, we first noted that OneR mainly selected sites in HA and NA for its single site models, and the OneR models could give significantly better average accuracies than the zero rule model (in which the accuracy is calculated by assigning all records to the class label that has the most observations). Among the sites, some have known functions while some others are not yet characterized. For example, site 188 in HA is known to be located at the helix 190 that surrounds the receptor-binding site and thus it affects host specificity [21], while site 142 in HA has not yet been well studied even though it was frequently selected as the top OneR classifier. On the other hand, JRip and PART generated multiple site models and while JRip usually did not outperform OneR, PART almost always outperformed OneR and JRip. Of interest, PART also outperformed RF in about 50% of the tested cases. Moreover, a higher accuracy generally could be achieved by PART when considering either the concatenated protein alignments or individual protein alignments. These results demonstrate a synergistic between sites within a single protein and sites in different proteins (in other words, the polygenic nature of IAV virulence in mice). This is consistent with the observations from various experimental studies, such as the ones that demonstrate intra-protein synergy in PB2 [22–27], PA [11], and NS1 [28, 29], and inter-protein synergy that involves combinations of PB2, PB1, PA, HA or NA [12, 30–36].

Further inspection on PART models across different IAV strains using IV dataset revealed that although HA had many more sites correlated with virulence, PB2 seemed to play more important role in determining IAV virulence. This was in agreement with the RF’s site importance ranking. In terms of their accuracy, PART models based on PB2 alone were usually as good as or even better than PART models based on HA; except when modelling the virulence of two-class H1N1, PART models based on HA were more superior (see (Additional file 2: Figure S2)). Moreover, PART models based on the concatenated IAV protein alignments had a high preference towards sites in PB2, and many sites in PB2 were also considered as the most important features for RF models (Table 3). Figure 5 that shows synergistic graphs for the two-class and three-class virulence models further clearly demonstrate this. Investigations on MIV dataset and datasets for specific IAV or mouse strain also revealed the dominance of PB2 in most of the cases (data not shown). When sites in PB2 did not dominate, the sites in HA dominated, such as in the case for the two-class H1N1 dataset.

The critical role of PB2 in determining virulence in mice have been indeed highlighted for various strains, including H3N2 [34, 37], H5N1 [22–24, 38, 39], H5N8 [26, 40], H7N9 [41–45], H9N2 [25, 27, 45, 46] and H10N8 [45]. Among the top 20 sites in PB2 for PART models, sites 627 and 701 have been repeatedly shown to affect IAV virulence in mammals including mice. Site 627 is considered critical for efficient replication, while site 701 influences polymerase activity via its interaction with the nuclear import factor importin α that mediates the transport of proteins into nucleus [47]. Other top sites in PB2 are also known to contribute to virulence. For examples, site 714 (top 20 for the two-class IV dataset) influences replication efficiency and IAV virulence in mice in combination with site 701 [23, 48, 49]; site 66 (top 20 for the three-class IV dataset) sets a prerequisite for acquiring virulence [50]; and site 158 (top 20 for the two-class and three-class IV dataset; specifically, top one for the three-class) strongly influences the virulence of both pandemic H1N1 and H5 influenza viruses in mice [51]. Experimental evidence for the contribution of other top sites in PB2 to virulence, e.g., sites 80, 110 and 153, are still none to our knowledge. On the other hand, some other sites not in the top list have been shown to play a role in dictating virulence, e.g., sites 147, 339 and 588 that can synergize to give rise a higher level of virulence [24].

Next, the synergistic graph for the two-class virulence models interestingly presented a clustering of two subgraphs for sites in PART virulence models, with sites PB2–714, PA-97 and NS1–42 act as a bottleneck (a node with high betweenness centrality, i.e., having many shortest paths going through it) connecting the two subgraphs. For the three-class models, the synergistic graph containing top site pairs concentrated and expanded in the subnetwork that included sites PB2–80, PB2–110, PB2–153, PB2–297, NA-300, NS1–42, and M1–215. This may indicate a greater role of these sites in sensitizing the virulence level of IAV infections. For example, site 42 within the RNA-binding domain of NS1 influences the capability of the protein in binding double-stranded RNA and it determines the degree of pathogenicity in mice [52]. This site also influences the activation of IRF3 and regulation of host interferon response, which subsequently influences the efficiency of viral replication [53]. Another site that has been experimentally explored is site 215 in M1, which also contributes to the degree of IAV virulence [54].

Overall, PART, with its approach that combines separate-and-conquer strategy and partial decision tree, has been a suitable method to generate sequence-based virulence models that are not only considerably good in performance, but also provides interpretable information. But here, rather than relying on a single model developed from a single training dataset, the information was extracted from 100 models learned independently from different training datasets. While bias due to imbalanced classes were resolved by under-sampling to obtain balanced classes, the iterations might help reducing bias due to over-sampling of a particular mouse or IAV strain. Furthermore, we also noted from the confusion matrix that PART models tended to misclassify the avirulent (or less virulent) strains as virulent (or more virulent) ones rather than misclassify the virulent (more virulent) strains as avirulent (or less virulent) ones. In practice, this is preferred since classifying the virulent strains as avirulent ones is a worse decision that can cost lives. Moreover, we also investigated the effect of increasing the training size for learning PART models (data not shown). Using the two-class and three-class IV datasets containing the concatenated protein alignments, the mean accuracy of PART models based on the training size of 80% or 90% of the total records was about 2–3% higher than the mean accuracy of PART models based on the training size of 60%, but it came at the cost of higher standard deviation (about 1.3–3.3% higher) and average number of rules (3–6 rules more for two-class and 12–20 rules more for three-class; in other words, more complex models). Increasing the training size up to 99% of the total records led not only to much higher variance, but also a drop in the mean accuracy. Thus, with additional consideration that there were high overlaps between top sites from PART models trained on 80% or 90% datasets and top sites trained on 60% datasets, and we observed that the top sites for models trained on 80 and 90% datasets were still dominated by sites in PB2, presenting results from models trained on 60% of datasets is justifiable.

In terms of their accuracy, PART models achieved moderate performance for various datasets being learned. The average accuracy over 100 models ranged between 65.0 and 84.4% (15.0–34.4% above baseline) for the two-class datasets that utilized all IAV proteins, and between 54.0 and 66.6% (20.7–33.3% above baseline) for the three-class datasets (see Table 2). Learning from subsets of specific mouse or IAV strains revealed that some strains were easier while others were harder to learn. Of interest, while the average accuracies were relatively the same for the two-class datasets regardless the host information was included or not, a significant improvement (3.9% in increase of accuracy) was observed when incorporating host information for the three-class dataset. Thus, using learning approaches that further incorporate host information shall be encouraged, especially since several laboratory experiments have demonstrated the importance of host genetic backgrounds in determining IAV virulence [55–61], even at a substrain level [62]. In particular, with the availability of genomes and proteomes of various mouse strains, sophisticated methods that are based on host-pathogen protein-protein interactions might be of interest. If successful, an implementation of such methods may be translated to human cases and other diseases to improve our understanding about disease mechanisms, establish a foundation for future personalized medicine, and provide a better surveillance. Nevertheless, the development of the approaches will be more fruitful if there is a significant increase in the number of influenza experiments carried out with mouse and IAV strains that are still limited in their number of studies.

## Conclusions

In summary, we have developed benchmark datasets and explored rule-based and RF approaches for modelling IAV virulence. To our knowledge, the datasets are currently the biggest aggregation of IAV infections in mice, and the number of the infection records can still grow. The creation of these benchmark datasets will be beneficial for further understanding the molecular principles underlying influenza mechanisms since mice have been a major animal model for influenza. In this study, we utilized the datasets to assess the predictability of IAV virulence for specific and across mouse and IAV strains, and to identify top proteins sites and synergy between protein sites that contribute to IAV virulence. Overall, our study confirmed the polygenic nature of IAV virulence, with several sites in PB2 playing more dominant roles. Not only sites that are well known as IAV virulence markers, e.g. 627, 701 and 714, but also some other sites in PB2 not yet known influencing virulence were identified. Nonetheless, modelling virulence is a very challenging problem due to the nature of complex interactions that underlie the phenotype, which involve not only viral factors, but also host factors. Hence, future works shall incorporate more host information, especially the host proteomic data that are now widely available for various mouse strains. Applying different machine learning approaches and protein features, and posing virulence modelling as a regression problem that predicts MLD50 shall also be considered.

## Methods

### Collection of IAV infections in mice with virulence information

Journal publications containing virulence information of IAV infections in non-transgenic and non-knock-out inbred mice – which were searched using Google or PubMed search engines (with keywords that included influenza, infection, mouse, virulence, virus and LD50), found in the citations of retrieved articles, or recommended automatically by ScienceDirect – were collected. Each unique infection involving specific IAV strain and specific mouse strain and with known value of MLD50 was recorded. Infections without MLD50 values but whose weight loss and/or survival data of infected mice per infection dose could be estimated from the relevant figures, were also recorded and used to estimate the lower or upper bound of MLD50; few of them were used to estimate the exact MLD50 using the Reed and Muench method [63]. Various MLD50 units, which included the plaque forming unit (PFU), focus forming unit (FFU), egg infectious dose (EID50), tissue culture infectious dose (TCID50), and cell culture infectious dose (CCID50), were assumed to measure the same quantity.

### Virulence classification

In addition to the assumption on the equality of various MLD50 units, the MLD50 thresholds of 10^3.0^ and 10^6.0^ were used for virulence classification. The thresholds are used by WHO when classifying influenza virulence in mice in EID50 unit [64]. In this regard, for the two-class problems, the levels of virulence were categorized into avirulent class if the MLD50 was > 10^6.0^ and virulent class otherwise. When the class of an infection could not be determined from the lower or upper bound of MLD50, then the following rules were used:

#### Rule 1

An infection is avirulent if:

(i) the infection dose between 10^4.0^ and 10^6.0^ leads to < 15% average weight loss;
(ii) the infection dose ≥10^5.0^ does not kill any mouse; or
(iii) the infection dose between 10^3.0^ and 10^4.0^ leads to ≤10% average weight loss.

#### Rule 2

An infection is virulent if:

(i) the infection dose ≤10^5.0^ leads to ≥15% average weight loss;
(ii) the infection dose ≤10^3.0^ leads to ≥10% average weight loss; or
(iii) the infection dose ≤10^3.5^ kills ≥10% mice.

For the three-class classification problems, the levels of virulence were categorized into low virulence if the MLD50 was > 10^6.0^, intermediate virulence if the MLD50 was ≤10^6.0^ and > 10^3.0^, and high virulent otherwise. When the class of an infection could not be determined from the lower or upper bound of MLD50, then the following rules were used:

#### Rule 3

An infection is low virulence if it is considered avirulent (as given in the two class labelling).

#### Rule 4

An infection is intermediate virulence if:

(i) the infection dose < 10^4.0^ leads to ≥10% average weight loss;
(ii) the infection dose between 10^4.0^ and 10^5.0^ leads to ≥15% average weight loss; or
(iii) the infection dose between 10^5.0^ and 10^6.0^ leads to ≥20% average weight loss.

#### Rule 5

An infection is high virulence if:

(i) the infection dose ≤10^6.0^ kills ≥80% mice or leads to ≥25% average weight loss; or
(ii) the infection dose ≤10^1.0^ kills ≥20% mice.

The above procedure created the initial dataset for IAV infections in mice with virulence information for this study (Additional file 5: Table S1). Following this, multiple records of infections involving specific IAV and mouse strains were reduced into a single record (Additional file 6: Table S2) by the following procedure (termed as **RULE 6**):

(i) Specify the majority class of the three-class virulence assignment for those records; when no majority, consider the class that is more or the most virulent.
(ii) Select the record with:
  the highest lower bound of MLD50 value when only the lower bound of MLD50 values is presented;
  the lowest exact or upper bound of MLD50 value when they are available; but when the highest lower bound of MLD50 value is lower than this value, then calculate the average of those two values and assign the virulence class as described previously.

This procedure selected a record that had the more or most virulent information among the records, except when only the lower bound of MLD50 values was available; or alternatively, with the majority class if it could be determined. Note that when applying this procedure, the recombinants of naturally occurring or wild-type IAV strains were considered representing the wild-type version. In a similar fashion, we applied this procedure to reduce multiple records of infections of a specific IAV strain in different mouse strains into a single record (Additional file 7: Table S3).

### Collection of related genomes and main proteins

The availability of the sequences of IAV strains in the public databases, when they are not suggested in the literature, were checked online using Google, GenBank and GISAID search engines, or search offline in the genomeset.dat and influenza_na.dat files that were retrieved from NCBI Influenza Virus Resource [65]. The sequences of the viruses, if available, were collected from GenBank [66] or GISAID [67]. When the genome of a particular virus were incomplete, the HA and/or NA of the virus were/was BLASTed against GenBank database of all influenza viruses and the top virus hit whose complete genome was available was used to extrapolate the incomplete genome (Additional file 11: Table S7). Considering the closeness between their collection year and name, the genomes of A/Turkey/15/2006(H5N1) and A/chicken/Shandong/L1/2007(H9N2) were used to represent the genomes of A/Turkey/13/2006(H5N1) and A/chicken/Shandong/lx1023/2007(H9N2), respectively, which were not available during this study. Furthermore, we extrapolated partial IAV sequences by using the closest complete IAV sequence identified by BLAST (Additional file 12: Table S8). Following the collection of IAV genomes and their extrapolation, the 12 IAV proteins were obtained by identifying their coding sequence regions using Influenza Virus Sequence Annotation Tool available at the NCBI Influenza Virus Resource [65] and then translating them into proteins according to standard genetic code. Some proteins, mainly for recombinant and/or mutant viruses, were generated from existing proteins according to the list of amino acid differences at various sites reported in the literature. Note that some IAVs were represented by different versions of genomes or sets of proteins, but the reassortant or mutant viruses were mainly reconstructed from one of the versions. The metadata for IAV nucleotide sequences used in this study, reconstructed recombinant and/or mutant IAVs generated from those sequences, and the acknowledgement of the source of the GISAID sequences are provided in Table S4-S6 (Additional files 8, 9 and 10), respectively. They were also made available in DR-NTU (Data) under the title “Virulence Information for Influenza Virus Infections (VI2VI) in Mice” [68], and further update will be available in the link: 10.21979/N9/ILQBAB.

### Machine learning approaches for IAV virulence prediction

Three rule-based machine learning approaches, i.e., OneR, JRip and PART that are available in RWeka version 0.4.39 [69], and random forest (RF) that is available in randomForest package version 4.6.14 for R software (R version 3.5.1 [70] was used for all statistical and computational works in this study) were explored to develop predictive models for IAV virulence. Various input datasets were considered (see the first section of results), but in general, the input datasets consisted of IAV proteins that have been aligned with muscle package version 3.8.425 [71] and their target virulence class. The datasets included either the concatenated alignments of all IAV proteins or individual alignment of PB2, PB1, PA, HA, NP, NA, M1, NS1, PB1-F2, PA-X, M2 or NS2 proteins. Each column in the alignment that contained more than one symbol was considered as a single feature vector – H3 and N2 numberings were used to label the position in the alignments of HA and NA, respectively. Input datasets that incorporated the host strain information, where each amino acid in the alignments was tagged with a symbol indicating associated host strain, were also considered. For each input dataset, each learning algorithm and each of the two-class and three-class datasets, rule-based and RF models were learned independently 100 times. In each iteration, the dataset was balanced by reducing the size of the bigger (biggest) class to the size of the smaller (smallest) class through sampling without replacement. Unless stated otherwise, 60% of the records (rows of the alignment) from each virulent class were used as training data for learning a model, while the rest were used as test data. Performance metrics that included accuracy, (macro-average) precision and (macro-average) recall were calculated to evaluate the models.

### Visualization, statistical analyses and site rankings

The concatenated alignments of all IAV proteins were visualized in 3D Cartesian coordinates. For this, a matrix of pairwise distances from the concatenated protein alignments was computed using Fitch similarity matrix and then the Kruskal’s non-metric multidimensional scaling available in MASS package version 7.3.50 [72] for R software was applied to place each record of the concatenated protein sequences in a 3D space.

The correlations between sites in the alignment and the target virulence class were measured using the Benjamini-Hochberg adjusted *p*-values of the chi-square test of independence. The –log (adjusted p-value) of the test over the sites of each IAV protein was visualized with a line plot.

For each of the two-class and three-class datasets, a three-way ANOVA model (with interactions) was built to identify factors that influence the accuracy of the virulence models. The factors included the dataset (with 7 levels: MIV, IV, BALB/C, C57BL/6, H1N1, H3N2 and H5N1), protein alignment (with 13 levels: all proteins, PB2, PB1, PA, HA, NP, NA, M1, NS1, PB1-F2, PA-X, M2 and NS2) and machine learning algorithm (with 3 levels: OneR, JRip and PART). The Tukey’s HSD post hoc test was then carried out to identify pairs of groups (virulence models) that were significantly different. The Wilcoxon signed-rank sum test was also used to test the null hypothesis that the median of the accuracy of PART model learned from any dataset containing the concatenated protein alignments is greater than that of the corresponding RF model. The p-values of the tests were adjusted using the Bonferroni method.

Following 100 independent learnings from the two-class and three-class IV datasets, the protein sites from models learned using each algorithm were ranked. For OneR, the sites were ranked according to their frequency of being selected for the models; for JRip and PART, the sites were ranked according to their average contribution to the accuracy of learned models; and for RF, the sites were ranked according to their contribution to the average mean decrease in accuracy. For PART models, we also ranked the site pairs according to their average contribution to the accuracy of learned models and visualized the synergistic graph arises from the top 50 site pairs using igraph package version 1.2.2 [73] for R software.

## Supplementary information

**Additional file 1: Figure S1.** Accuracy distribution of 100 OneR/JRip/PART models learned independently from two-class and three-class BALB/C, C57BL/6, H1N1, H3N2, and H5N1 datasets containing either the concatenated alignments of all IAV proteins or an individual alignment of PB2, PB1, PA, HA, NP, NA, M1, NS1, PB1-F2, PA-X, M2 or NS2 proteins.

**Additional file 2: Figure S2.** Accuracy distribution of 100 PART/random forest models learned independently from two-class and three-class datasets containing the concatenated alignments of IAV proteins.

**Additional file 3: Figure S3.** Multiple comparisons between mean accuracies of OneR, JRip and PART models for IAV virulence based on two-class and three-class MIV and IV datasets containing either the concatenated alignment of all IAV proteins or an individual alignment of PB2, PB1, PA, HA, NP, NA, M1, NS1, PB1-F2, PA-X, M2 and NS2 proteins.

**Additional file 4; Figure S4.** Multiple comparisons between mean accuracies of OneR, JRip and PART models for IAV virulence based on two-class and three-class BALB/C, C57BL/6, H1N1, H3N2 and H5N1 datasets containing either the concatenated alignment of all IAV proteins or an individual alignment of PB2, PB1, PA, HA, NP, NA, M1, NS1, PB1-F2, PA-X, M2 and NS2 proteins.

**Additional file 5: Table S1.** Initial dataset for IAV infections in mice with virulence information (with supplementary references).

**Additional file 6: Table S2.** Reduction of multiple records for infection involving specific IAV and mouse strains into a single record (with supplementary references).

**Additional file 7: Table S3.** Reduction of multiple records for infection of a specific IAV strain in different mouse strains into a single record (with supplementary references).

**Additional file 8: Table S4.** Metadata of IAV nucleotide sequences used in this study (with supplementary references).

**Additional file 9: Table S5.** Mutant and reassortant IAVs generated in this study.

**Additional file 10: Table S6.** GISAID acknowledgement table for sequences used in this study.

**Additional file 11: Table S7.** Extrapolated incomplete IAV genomes.

**Additional file 12: Table S8.** Extrapolated partial IAV segments.

**Additional file 13: Table S9.** Examples of rules generated by OneR, JRip and PART for two-class and three-class MIV datasets containing concatenated alignments of IAV proteins.

**Additional file 14: Table S10.** Examples of rules generated by OneR, JRip and PART for two-class and three-class IV datasets containing concatenated alignments of IAV proteins.

**Additional file 15: Table S11.** Examples of rules generated by OneR, JRip and PART for two-class and three-class BALB/C datasets containing concatenated alignments of IAV proteins.

**Additional file 16: Table S12.** Examples of rules generated by OneR, JRip and PART for two-class and three-class C57BL/6 datasets containing concatenated alignments of IAV proteins.

**Additional file 17: Table S13.** Examples of rules generated by OneR, JRip and PART for two-class and three-class H1N1 datasets containing concatenated alignments of IAV proteins.

**Additional file 18: Table S14.** Examples of rules generated by OneR, JRip and PART for two-class and three-class H3N2 datasets containing concatenated alignments of IAV proteins.

**Additional file 19: Table S15.** Examples of rules generated by OneR, JRip and PART for two-class and three-class H5N1 datasets containing concatenated alignments of IAV proteins.

## Data Availability

All figures and tables generated in this study are available in this article and its additional files. The sequences used in this study are available in GenBank or GISAID or can be requested from the corresponding author of related publications – the GenBank/GISAID accession number or reference for the sequences can be found in Table S4 (Additional file 8). The figures and tables, in addition to the processing and analysis scripts, are also available in DR-NTU (Data) repository 10.21979/N9/ILQBAB.

## Abbreviations

1R: OneR
avir: avirulent
CCID50: cell culture infectious dose
EID50: egg infectious dose
FFU: focus forming unit
HA: hemagglutinin
hi: high virulence
IAV: influenza A virus
int: intermediate virulence
IP: IAV protein dataset
IV: IVir ×_I_ IP dataset
IVir: IAV virulence dataset (virulence dataset whose multiple IAV infection records across different mouse strains were reduced into a single record)
JR: JRip
LD50: lethal dose 50
lo: low virulence
M1: matrix protein 1
M2: matrix protein 2
MIV: MIVir ×_I_ IP dataset
MIVir: mouse-IAV virulence dataset (virulence dataset whose multiple records involving specific IAV and mouse strain were reduced into a single record)
MLD50: mouse lethal dose 50
NA: neuraminidase
NP: nucleocapsid protein
NS1: non-structural protein 1
NS2: non-structural protein 2
PA: acidic RNA polymerase
PB1: basic RNA polymerase 1
PB1-F2: PB1 frame 2
PB2: basic RNA polymerase 2
PFU: plaque forming unit
PT: PART
RF: random forest
TCID50: tissue culture infectious dose
vir: virulent
