# Supplementary material for: Rule-based meta-analysis reveals the major role of PB2 in influencing influenza A virus virulence in mice
Source: BMC Genomics. 2019 Dec 24;20(Suppl 9):973. doi: 10.1186/s12864-019-6295-8 (PMC6929465; doi:10.1186/s12864-019-6295-8)
Supplement: Supplementary file 1 — Additional file 1: Figure S1. Accuracy distribution of 100 OneR/JRip/PART models learned independently from two-class and three-class BALB/C, C57BL/6, H1N1, H3N2, and H5N1 datasets containing either the concatenated alignments of all IAV proteins or an individual alignment of PB2, PB1, PA, HA, NP, NA, M1, NS1, PB1-F2, PA-X, M2 or NS2 proteins. [file 12864_2019_6295_MOESM1_ESM.pptx]

## Slide 1
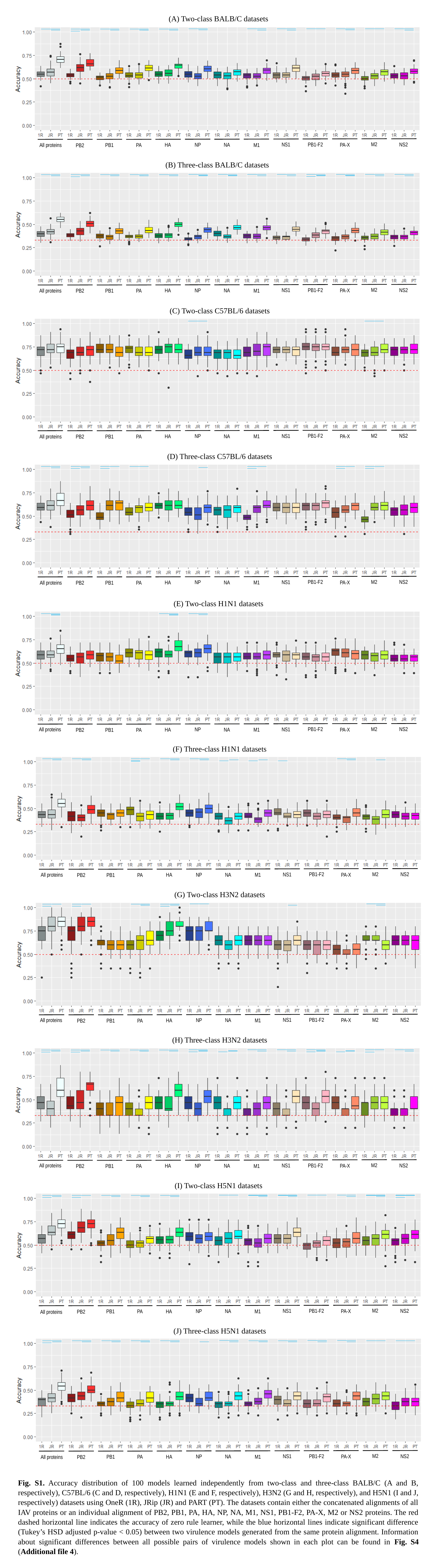

(A) Two-class BALB/C datasets
M2
PB1-F2
NS2
NS1
NP
NA
PA-X
HA
M1
PB1
PA
All proteins
PB2
(B) Three-class BALB/C datasets
M2
PB1-F2
NS2
NS1
NP
NA
PA-X
HA
M1
PB1
PA
All proteins
PB2
(C) Two-class C57BL/6 datasets
M2
PB1-F2
NS2
NS1
NP
NA
PA-X
HA
M1
PB1
PA
All proteins
PB2
(D) Three-class C57BL/6 datasets
M2
PB1-F2
NS2
NS1
NP
NA
PA-X
HA
M1
PB1
PA
All proteins
PB2
(E) Two-class H1N1 datasets
M2
PB1-F2
NS2
NS1
All proteins
NP
NA
PA-X
HA
M1
PB1
PA
PB2
(F) Three-class H1N1 datasets
M2
PB1-F2
NS2
NS1
NP
NA
PA-X
All proteins
HA
M1
PB1
PA
PB2
(G) Two-class H3N2 datasets
M2
PB1-F2
NS2
NS1
All proteins
NP
NA
PA-X
HA
M1
PB1
PA
PB2
(H) Three-class H3N2 datasets
M2
All proteins
PB1-F2
NS2
NS1
NP
NA
PA-X
HA
M1
PB1
PA
PB2
(I) Two-class H5N1 datasets
M2
PB1-F2
NS2
NS1
NP
NA
PA-X
HA
M1
PB1
PA
PB2
All proteins
(J) Three-class H5N1 datasets
M2
PB1-F2
NS2
NS1
NP
NA
PA-X
HA
M1
PB1
PA
PB2
All proteins
Fig. S1. Accuracy distribution of 100 models learned independently from two-class and three-class BALB/C (A and B, respectively), C57BL/6 (C and D, respectively), H1N1 (E and F, respectively), H3N2 (G and H, respectively), and H5N1 (I and J, respectively) datasets using OneR (1R), JRip (JR) and PART (PT). The datasets contain either the concatenated alignments of all IAV proteins or an individual alignment of PB2, PB1, PA, HA, NP, NA, M1, NS1, PB1-F2, PA-X, M2 or NS2 proteins. The red dashed horizontal line indicates the accuracy of zero rule learner, while the blue horizontal lines indicate significant difference (Tukey’s HSD adjusted p-value < 0.05) between two virulence models generated from the same protein alignment. Information about significant differences between all possible pairs of virulence models shown in each plot can be found in Fig. S4 (Additional file 4).
