# Supplementary material for: Rule-based meta-analysis reveals the major role of PB2 in influencing influenza A virus virulence in mice
Source: BMC Genomics. 2019 Dec 24;20(Suppl 9):973. doi: 10.1186/s12864-019-6295-8 (PMC6929465; doi:10.1186/s12864-019-6295-8)
Supplement: Supplementary file 2 — Additional file 2: Figure S2. Accuracy distribution of 100 PART/random forest models learned independently from two-class and three-class datasets containing the concatenated alignments of IAV proteins. [file 12864_2019_6295_MOESM2_ESM.pptx]

## Slide 1
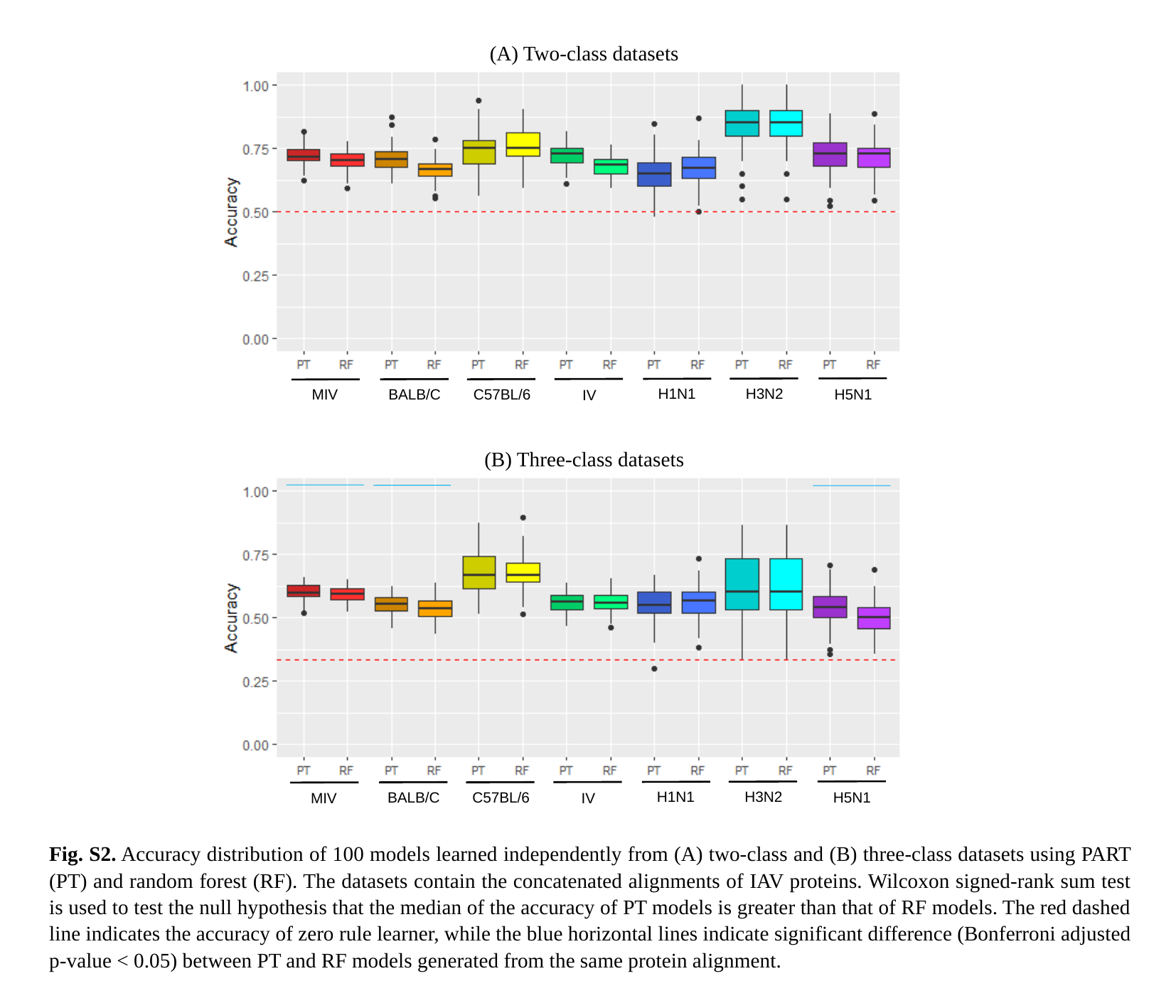

(A) Two-class datasets
H3N2
H1N1
BALB/C
C57BL/6
H5N1
MIV
IV
(B) Three-class datasets
H3N2
H1N1
BALB/C
C57BL/6
H5N1
MIV
IV
Fig. S2. Accuracy distribution of 100 models learned independently from (A) two-class and (B) three-class datasets using PART (PT) and random forest (RF). The datasets contain the concatenated alignments of IAV proteins. Wilcoxon signed-rank sum test is used to test the null hypothesis that the median of the accuracy of PT models is greater than that of RF models. The red dashed line indicates the accuracy of zero rule learner, while the blue horizontal lines indicate significant difference (Bonferroni adjusted p-value < 0.05) between PT and RF models generated from the same protein alignment.
