# Supplementary material for: Rule-based meta-analysis reveals the major role of PB2 in influencing influenza A virus virulence in mice
Source: BMC Genomics. 2019 Dec 24;20(Suppl 9):973. doi: 10.1186/s12864-019-6295-8 (PMC6929465; doi:10.1186/s12864-019-6295-8)
Supplement: Supplementary file 3 — Additional file 3: Figure S3. Multiple comparisons between mean accuracies of OneR, JRip and PART models for IAV virulence based on two-class and three-class MIV and IV datasets containing either the concatenated alignment of all IAV proteins or an individual alignment of PB2, PB1, PA, HA, NP, NA, M1, NS1, PB1-F2, PA-X, M2 and NS2 proteins. [file 12864_2019_6295_MOESM3_ESM.pptx]

## Slide 1
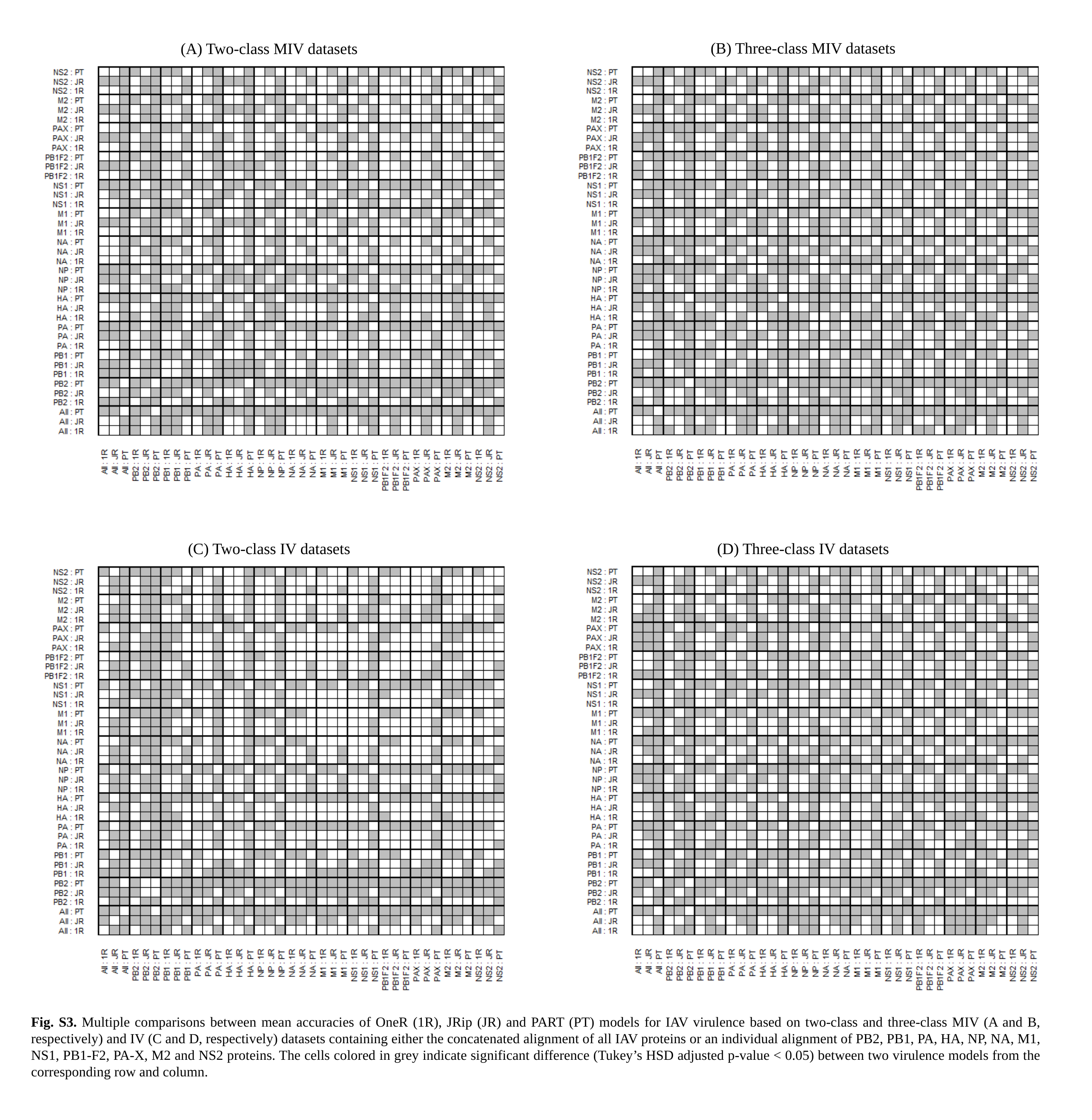

(B) Three-class MIV datasets
(A) Two-class MIV datasets
(C) Two-class IV datasets
(D) Three-class IV datasets
Fig. S3. Multiple comparisons between mean accuracies of OneR (1R), JRip (JR) and PART (PT) models for IAV virulence based on two-class and three-class MIV (A and B, respectively) and IV (C and D, respectively) datasets containing either the concatenated alignment of all IAV proteins or an individual alignment of PB2, PB1, PA, HA, NP, NA, M1, NS1, PB1-F2, PA-X, M2 and NS2 proteins. The cells colored in grey indicate significant difference (Tukey’s HSD adjusted p-value < 0.05) between two virulence models from the corresponding row and column.
