# Supplementary material for: Rule-based meta-analysis reveals the major role of PB2 in influencing influenza A virus virulence in mice
Source: BMC Genomics. 2019 Dec 24;20(Suppl 9):973. doi: 10.1186/s12864-019-6295-8 (PMC6929465; doi:10.1186/s12864-019-6295-8)
Supplement: Supplementary file 4 — Additional file 4; Figure S4. Multiple comparisons between mean accuracies of OneR, JRip and PART models for IAV virulence based on two-class and three-class BALB/C, C57BL/6, H1N1, H3N2 and H5N1 datasets containing either the concatenated alignment of all IAV proteins or an individual alignment of PB2, PB1, PA, HA, NP, NA, M1, NS1, PB1-F2, PA-X, M2 and NS2 proteins. [file 12864_2019_6295_MOESM4_ESM.pptx]

## Slide 1
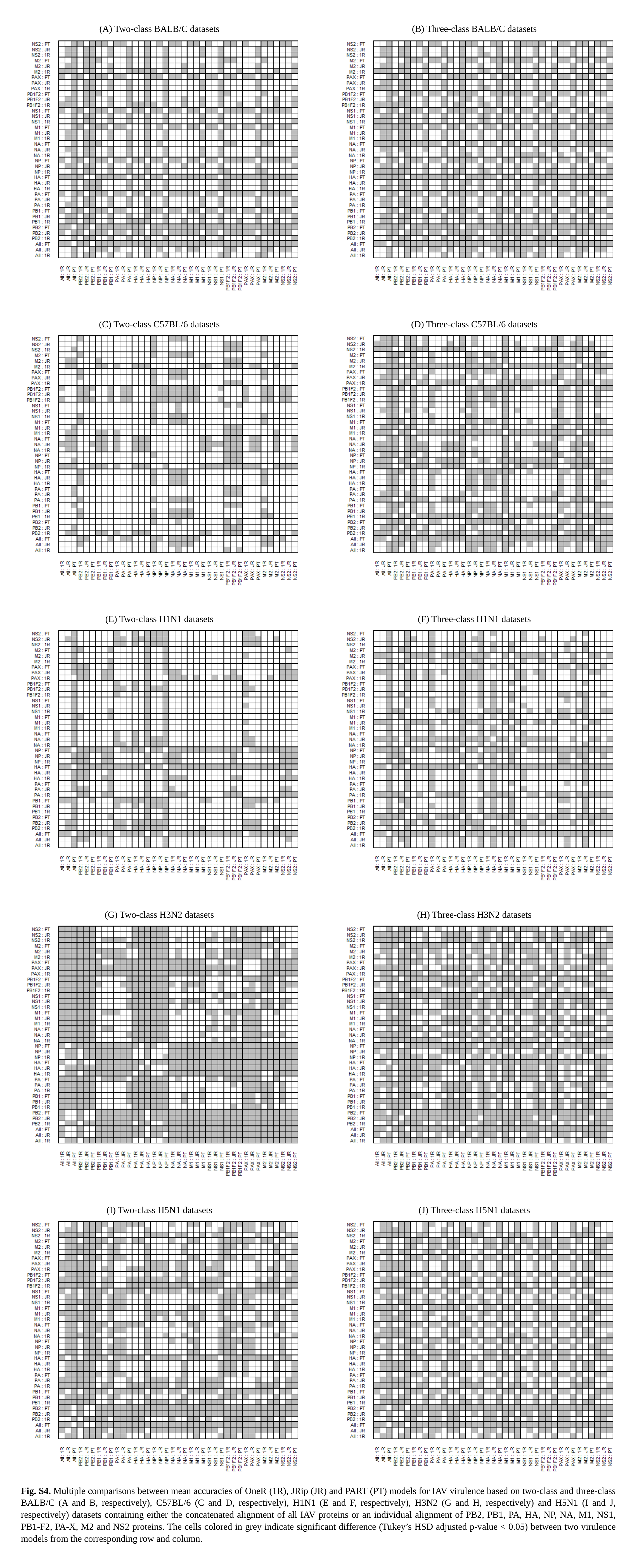

(A) Two-class BALB/C datasets
(B) Three-class BALB/C datasets
(C) Two-class C57BL/6 datasets
(D) Three-class C57BL/6 datasets
(E) Two-class H1N1 datasets
(F) Three-class H1N1 datasets
(G) Two-class H3N2 datasets
(H) Three-class H3N2 datasets
(I) Two-class H5N1 datasets
(J) Three-class H5N1 datasets
Fig. S4. Multiple comparisons between mean accuracies of OneR (1R), JRip (JR) and PART (PT) models for IAV virulence based on two-class and three-class BALB/C (A and B, respectively), C57BL/6 (C and D, respectively), H1N1 (E and F, respectively), H3N2 (G and H, respectively) and H5N1 (I and J, respectively) datasets containing either the concatenated alignment of all IAV proteins or an individual alignment of PB2, PB1, PA, HA, NP, NA, M1, NS1, PB1-F2, PA-X, M2 and NS2 proteins. The cells colored in grey indicate significant difference (Tukey’s HSD adjusted p-value < 0.05) between two virulence models from the corresponding row and column.
