# Supplementary material for: Rule-based meta-analysis reveals the major role of PB2 in influencing influenza A virus virulence in mice
Source: BMC Genomics. 2019 Dec 24;20(Suppl 9):973. doi: 10.1186/s12864-019-6295-8 (PMC6929465; doi:10.1186/s12864-019-6295-8)
Supplement: Supplementary file 7 — Additional file 7: Table S3. Reduction of multiple records for infection of a specific IAV strain in different mouse strains into a single record (with supplementary references). [file 12864_2019_6295_MOESM7_ESM.docx]

**Table S3.** Reduction of multiple records for infection of a specific IAV strain in different mouse strains into a single record. The records colored in red are the records being selected, with their LD50 values highlighted in bold if they are updated.

| **Reference** | **Host strain** | **Influenza strain** | **LD50 point estimate** | **LD50 lower bound** | **LD50 upper bound** | **Infection unit** | **Two-class virulence level** | **Three-class virulence level** |
| --- | --- | --- | --- | --- | --- | --- | --- | --- |
| Imai *et al*., 2017 (24) | BALB/C | A/Anhui/1/2013(H7N9) | 4.5 |  |  | PFU | Intermediate | Virulent |
| Zhu *et al*., 2015 (83) | C57BL/6 | A/Anhui/1/2013(H7N9) | 4.5 |  |  | TCID50 | Intermediate | Virulent |
| Pica *et al*., 2011 (54) | C57BL/6 | A/Brisbane/10/2007(H3N2) |  | 6 |  | PFU | Low | Avirulent |
| Pica *et al*., 2011 (54) | DBA/2 | A/Brisbane/10/2007(H3N2) |  | 6 |  | PFU | Low | Avirulent |
| Pica *et al*., 2011 (54) | C57BL/6 | A/Brisbane/59/2007(H1N1) |  | 6 |  | PFU | Low | Avirulent |
| Pica *et al*., 2011 (54) | DBA/2 | A/Brisbane/59/2007(H1N1) | 5 |  |  | PFU | Intermediate | Virulent |
| Manicassamy *et al*., 2010 (44) | C57BL/6 | A/California/04/2009(H1N1) | 4.7 |  |  | PFU | Intermediate | Virulent |
| Smee *et al*., 2012 (63) | BALB/C | A/California/04/2009(H1N1) | 3.5 |  |  | CCID50 | Intermediate | Virulent |
| Ye *et al*., 2010 (75) | DBA/2 | A/California/04/2009(H1N1) |  |  | 5.7 | TCID50 | High | Virulent |
| Pica *et al*., 2011 (54) | C57BL/6 | A/duck/Alberta/35/1976(H1N1) |  | 6 |  | PFU | Low | Avirulent |
| Pica *et al*., 2011 (54) | DBA/2 | A/duck/Alberta/35/1976(H1N1) |  | 6 |  | PFU | Low | Avirulent |
| Pica *et al*., 2011 (54) | C57BL/6 | A/duck/Ukraine/1/1963(H3N8) |  | 6 |  | PFU | Low | Avirulent |
| Pica *et al*., 2011 (54) | DBA/2 | A/duck/Ukraine/1/1963(H3N8) |  | 6 |  | PFU | Low | Avirulent |
| Choi *et al*., 2017 (9) | C57BL/6 | A/environment/Korea/W468/2014(H5N8) |  | 4 |  | PFU | Low | Avirulent |
| Choi *et al*., 2017 (9) | BALB/C | A/environment/Korea/W468/2014(H5N8) | 7.3 |  |  | PFU | Low | Avirulent |
| Otte *et al*., 2011 (51) | BALB/C | A/Hamburg/05/2009(H1N1) |  | 6 |  | PFU | Low | Avirulent |
| Otte *et al*., 2011 (51) | C57BL/6 | A/Hamburg/05/2009(H1N1) | 5.2 |  |  | PFU | Intermediate | Virulent |
| Otte *et al*., 2011 (51) | BALB/C | A/Hamburg/NY1580/2009(H1N1) |  | 6 |  | PFU | Low | Avirulent |
| Otte *et al*., 2015 (52) | C57BL/6 | A/Hamburg/NY1580/2009(H1N1) | 3.2 |  |  | PFU | Intermediate | Virulent |
| Pica *et al*., 2011 (54) | C57BL/6 | A/HongKong/1/1968(H3N2) |  | 6 |  | PFU | Low | Avirulent |
| Pica *et al*., 2011 (54) | DBA/2 | A/HongKong/1/1968(H3N2) | 5.5 |  |  | PFU | Intermediate | Virulent |
| Ping *et al*., 2011 (55) | CD-1 | A/HongKong/1/1968(H3N2) |  | 7.7 |  | PFU | Low | Avirulent |
| Lu *et al*., 1999 (41) | BALB/C | A/HongKong/156/1997(H5N1) | 5.9 |  |  | EID50 | Intermediate | Virulent |
| O'Neill *et al*., 2000 (50) | C57BL/6 | A/HongKong/156/1997(H5N1) |  |  | 2.8 | EID50 | High | Virulent |
| Blazejewska *et al*., 2011 (5) | C57BL/6 | A/HongKong/156/1997(H5N1) |  |  | 3.3 | PFU | High | Virulent |
| Blazejewska *et al*., 2011 (5) | DBA/2 | A/hvPR8/1934(H1N1) |  |  | 3.3 | PFU | High | Virulent |
| Kim *et al*., 2013 (33) | BALB/C | A/Korea/01/2009(H1N1) |  | 6 |  | PFU | Low | Avirulent |
| Kim *et al*., 2013 (33) | DBA/2 | A/Korea/01/2009(H1N1) | 2.83 |  |  | PFU | High | Virulent |
| Choi *et al*., 2017 (9) | C57BL/6 | A/ma452-G1-1/2014(H5N8) |  |  | 4 | PFU | High | Virulent |
| Choi *et al*., 2017 (9) | BALB/C | A/ma452-G1-1/2014(H5N8) | 1 |  |  | PFU | High | Virulent |
| Choi *et al*., 2017 (9) | C57BL/6 | A/ma452-G3-1/2014(H5N8) |  |  | 4 | PFU | High | Virulent |
| Choi *et al*., 2017 (9) | BALB/C | A/ma452-G3-1/2014(H5N8) | 2 |  |  | PFU | High | Virulent |
| Choi *et al*., 2017 (9) | C57BL/6 | A/ma452-G3-2/2014(H5N8) |  |  | 4 | PFU | High | Virulent |
| Choi *et al*., 2017 (9) | BALB/C | A/ma452-G3-2/2014(H5N8) | 0.5 |  |  | PFU | High | Virulent |
| Choi *et al*., 2017 (9) | C57BL/6 | A/ma452-G4-1/2014(H5N8) |  |  | 4 | PFU | High | Virulent |
| Choi *et al*., 2017 (9) | BALB/C | A/ma452-G4-1/2014(H5N8) | 1.3 |  |  | PFU | High | Virulent |
| Choi *et al*., 2017 (9) | C57BL/6 | A/ma468-G1-1/2014(H5N8) |  |  | 4 | PFU | High | Virulent |
| Choi *et al*., 2017 (9) | BALB/C | A/ma468-G1-1/2014(H5N8) | 1.7 |  |  | PFU | High | Virulent |
| Choi *et al*., 2017 (9) | C57BL/6 | A/ma468-G1-2/2014(H5N8) |  |  | 4 | PFU | High | Virulent |
| Choi *et al*., 2017 (9) | BALB/C | A/ma468-G1-2/2014(H5N8) | 1 |  |  | PFU | High | Virulent |
| Choi *et al*., 2017 (9) | C57BL/6 | A/ma468-G2-1/2014(H5N8) |  |  | 4 | PFU | High | Virulent |
| Choi *et al*., 2017 (9) | BALB/C | A/ma468-G2-1/2014(H5N8) | 0.7 |  |  | PFU | High | Virulent |
| Choi *et al*., 2017 (9) | C57BL/6 | A/ma468-G2-2/2014(H5N8) |  |  | 4 | PFU | High | Virulent |
| Choi *et al*., 2017 (9) | BALB/C | A/ma468-G2-2/2014(H5N8) | 1.5 |  |  | PFU | High | Virulent |
| Choi *et al*., 2017 (9) | C57BL/6 | A/ma468-G2-3/2014(H5N8) |  | 4 |  | PFU | Low | Avirulent |
| Choi *et al*., 2017 (9) | BALB/C | A/ma468-G2-3/2014(H5N8) | 4.8 |  |  | PFU | Intermediate | Virulent |
| Choi *et al*., 2017 (9) | C57BL/6 | A/ma468-G4-2/2014(H5N8) |  |  | 4 | PFU | High | Virulent |
| Choi *et al*., 2017 (9) | BALB/C | A/ma468-G4-2/2014(H5N8) | 0.5 |  |  | PFU | High | Virulent |
| Choi *et al*., 2017 (9) | C57BL/6 | A/mallard/Korea/W452/2014(H5N8) |  | 4 |  | PFU | Low | Avirulent |
| Choi *et al*., 2017 (9) | BALB/C | A/mallard/Korea/W452/2014(H5N8) | 7.5 |  |  | PFU | Low | Avirulent |
| Manicassamy *et al*., 2010 (44) | C57BL/6 | A/Netherlands/602/2009(H1N1) | 4.2 |  |  | PFU | Intermediate | Virulent |
| Pica *et al*., 2011 (54) | DBA/2 | A/Netherlands/602/2009(H1N1) | 0.5 |  |  | PFU | High | Virulent |
| Ye *et al*., 2010 (75) | BALB/C | A/Netherlands/602/2009(H1N1) | 6 |  |  | TCID50 | Intermediate | Virulent |
| Pica *et al*., 2011 (54) | C57BL/6 | A/NewCaledonia/20/1999(H1N1) | 5.9 |  |  | PFU | Intermediate | Virulent |
| Pica *et al*., 2011 (54) | DBA/2 | A/NewCaledonia/20/1999(H1N1) | 4.3 |  |  | PFU | Intermediate | Virulent |
| Ping *et al*., 2018 (56) | BALB/C | A/NewCaledonia/20/1999(H1N1) | 5.68 |  |  | PFU | Intermediate | Virulent |
| Jang *et al*., 2014 (28) | BALB/C | A/Panama/2007/1999(H3N2) |  | 6 |  | PFU | Low | Avirulent |
| Pica *et al*., 2011 (54) | C57BL/6 | A/Panama/2007/1999(H3N2) |  | 6 |  | PFU | Low | Avirulent |
| Pica *et al*., 2011 (54) | DBA/2 | A/Panama/2007/1999(H3N2) |  | 6 |  | PFU | Low | Avirulent |
| Blazejewska *et al*., 2011 (5) | C57BL/6 | A/PR8F/1934(H1N1) |  |  | 3.3 | PFU | High | Virulent |
| Blazejewska *et al*., 2011 (5) | DBA/2 | A/PR8F/1934(H1N1) |  |  | 3.3 | PFU | High | Virulent |
| Blazejewska *et al*., 2011 (5) | C57BL/6 | A/PR8M/1934(H1N1) | **3.3** | 3.3 |  | PFU | Intermediate | Virulent |
| Blazejewska *et al*., 2011 (5) | DBA/2 | A/PR8M/1934(H1N1) |  |  | 3.3 | PFU | High | Virulent |
| Pica *et al*., 2011 (54) | C57BL/6 | A/PuertoRico/8/1934(H1N1) | 1.4 |  |  | PFU | High | Virulent |
| Pica *et al*., 2011 (54) | DBA/2 | A/PuertoRico/8/1934(H1N1) | 0.4 |  |  | PFU | High | Virulent |
| Ping *et al*., 2018 (56) | BALB/C | A/PuertoRico/8/1934(H1N1) | 1.74 |  |  | PFU | High | Virulent |
| Srivastava *et al*., 2009 (66) | A/J | A/PuertoRico/8/1934(H1N1) |  |  | 3.3 | FFU | High | Virulent |
| Srivastava *et al*., 2009 (66) | CBA/J | A/PuertoRico/8/1934(H1N1) |  | 3.3 |  | FFU | Intermediate | Virulent |
| Srivastava *et al*., 2009 (66) | SJL/JOrlCrl | A/PuertoRico/8/1934(H1N1) |  | 3.3 |  | FFU | Low | Avirulent |
| Zhou *et al*., 2016 (81) | C3H | A/PuertoRico/8/1934(H1N1) |  |  | 6 | PFU | High | Virulent |
| Zhou *et al*., 2016 (81) | 129S1/SvPasCrlVr | A/PuertoRico/8/1934(H1N1) |  |  | 6 | PFU | High | Virulent |
| Zhou *et al*., 2016 (81) | FVB/NJ | A/PuertoRico/8/1934(H1N1) |  | 6 |  | PFU | Low | Avirulent |
| Gabriel *et al*., 2005 (18) | BALB/C | A/seal/Massachussetts/1-SC35M/1980(H7N7) | 2.8 |  |  | PFU | High | Virulent |
| Nurnberger *et al*., 2016 (49) | C57BL/6 | A/seal/Massachussetts/1-SC35M/1980(H7N7) |  |  | 3 | PFU | High | Virulent |
| Srivastava *et al*., 2009 (66) | DBA/2 | A/seal/Massachussetts/1-SC35M/1980(H7N7) |  |  | 3.3 | FFU | High | Virulent |
| Zhu *et al*., 2013 (82) | C57BL/6 | A/Shanghai/4664T/2013(H7N9) |  |  | 5.6 | TCID50 | Intermediate | Virulent |
| Zhu *et al*., 2013 (82) | BALB/C | A/Shanghai/4664T/2013(H7N9) |  |  | 5.6 | TCID50 | High | Virulent |
| Zhu *et al*., 2013 (82) | ICR | A/Shanghai/4664T/2013(H7N9) |  |  | 5.6 | TCID50 | Intermediate | Virulent |
| Otte *et al*., 2011 (51) | BALB/C | A/SolomonIslands/3/2006(H1N1) |  | 6 |  | PFU | Low | Avirulent |
| Otte *et al*., 2011 (51) | C57BL/6 | A/SolomonIslands/3/2006(H1N1) |  | 6 |  | PFU | Low | Avirulent |
| Pica *et al*., 2011 (54) | DBA/2 | A/SolomonIslands/3/2006(H1N1) | 3.9 |  |  | PFU | Intermediate | Virulent |
| Pica *et al*., 2011 (54) | C57BL/6 | A/swine/Kansas/77778/2007(H1N1) | 2.5 |  |  | PFU | High | Virulent |
| Pica *et al*., 2011 (54) | DBA/2 | A/swine/Kansas/77778/2007(H1N1) | 1 |  |  | PFU | High | Virulent |
| Pica *et al*., 2011 (54) | C57BL/6 | A/swine/Spain/40564/2002(H1N2) | 4.7 |  |  | PFU | Intermediate | Virulent |
| Pica *et al*., 2011 (54) | DBA/2 | A/swine/Spain/40564/2002(H1N2) | 1.3 |  |  | PFU | High | Virulent |
| Pica *et al*., 2011 (54) | C57BL/6 | A/swine/Spain/53207/2004(H1N1) | 5.7 |  |  | PFU | Intermediate | Virulent |
| Pica *et al*., 2011 (54) | DBA/2 | A/swine/Spain/53207/2004(H1N1) | 2.2 |  |  | PFU | High | Virulent |
| Pica *et al*., 2011 (54) | C57BL/6 | A/swine/Spain/54008/2004(H3N2) |  | 6 |  | PFU | Low | Avirulent |
| Pica *et al*., 2011 (54) | DBA/2 | A/swine/Spain/54008/2004(H3N2) | 5.5 |  |  | PFU | Intermediate | Virulent |
| Pica *et al*., 2011 (54) | C57BL/6 | A/swine/Texas/4199-2/1998(H3N2) | 6.2 |  |  | PFU | Low | Avirulent |
| Pica *et al*., 2011 (54) | DBA/2 | A/swine/Texas/4199-2/1998(H3N2) | 5.7 |  |  | PFU | Intermediate | Virulent |
| Otte *et al*., 2011 (51) | BALB/C | A/Thailand/KAN-1/2004(H5N1) | 0.3 |  |  | PFU | High | Virulent |
| Otte *et al*., 2011 (51) | C57BL/6 | A/Thailand/KAN-1/2004(H5N1) | 1.8 |  |  | PFU | High | Virulent |
| Pica *et al*., 2011 (54) | C57BL/6 | A/Wisconsin/67/2005(H3N2) |  | 6 |  | PFU | Low | Avirulent |
| Pica *et al*., 2011 (54) | DBA/2 | A/Wisconsin/67/2005(H3N2) |  | 6 |  | PFU | Low | Avirulent |
| Huo *et al*., 2018 (22) | C57BL/6 | A/WSN/1933(H1N1) |  |  | 2.3 | PFU | High | Virulent |
| Quan *et al*., 2008 (60) | BALB/C | A/WSN/1933(H1N1) | 2.3 |  |  | PFU | High | Virulent |
| Hatesuer *et al*., 2013 (20) | C57BL/6 | maA/HongKong/1/1968(H3N2) | **1** | 1 |  | FFU | High | Virulent |
| Leist *et al*., 2016 (37) | A/J | maA/HongKong/1/1968(H3N2) |  |  | 1 | FFU | High | Virulent |
| Leist *et al*., 2016 (37) | 129S1/SvImJ | maA/HongKong/1/1968(H3N2) |  |  | 1 | FFU | High | Virulent |
| Leist *et al*., 2016 (37) | NOD/ShiLtJ | maA/HongKong/1/1968(H3N2) | 1.38 |  |  | FFU | High | Virulent |
| Leist *et al*., 2016 (37) | NZO/HILtJ | maA/HongKong/1/1968(H3N2) |  | 5 |  | FFU | Low | Avirulent |
| Leist *et al*., 2016 (37) | CAST/EiJ | maA/HongKong/1/1968(H3N2) |  |  | 1 | FFU | High | Virulent |
| Leist *et al*., 2016 (37) | PWK/PhJ | maA/HongKong/1/1968(H3N2) |  | 5 |  | FFU | Low | Avirulent |
| Leist *et al*., 2016 (37) | WSB/EiJ | maA/HongKong/1/1968(H3N2) |  |  | 1 | FFU | High | Virulent |
| Pica *et al*., 2011 (54) | C57BL/6 | rA/PR8(123578)/ALB76(46)(H1N1) | 3.5 |  |  | PFU | Intermediate | Virulent |
| Pica *et al*., 2011 (54) | DBA/2 | rA/PR8(123578)/ALB76(46)(H1N1) | 1.2 |  |  | PFU | High | Virulent |
| Pica *et al*., 2011 (54) | C57BL/6 | rA/PR8(123578)/UKR63(46)(H3N8) |  | 6 |  | PFU | Low | Avirulent |
| Pica *et al*., 2011 (54) | DBA/2 | rA/PR8(123578)/UKR63(46)(H3N8) | 5.5 |  |  | PFU | Intermediate | Virulent |
| Lu *et al*., 1999 (41) | BALB/C | rA/X-31(H3N2) |  | 5.2 |  | EID50 | Low | Avirulent |
| Pica *et al*., 2011 (54) | C57BL/6 | rA/X-31(H3N2) | 5.3 |  |  | PFU | Intermediate | Virulent |
| Pica *et al*., 2011 (54) | DBA/2 | rA/X-31(H3N2) | 0.7 |  |  | PFU | High | Virulent |

**References**

1. Ainai A, Hasegawa H, Obuchi M, Odagiri T, Ujike M, Shirakura M, et al. Host Adaptation and the Alteration of Viral Properties of the First Influenza A/H1N1pdm09 Virus Isolated in Japan. PLoS One. 2015;10(6):e0130208.

2. Belser JA, Lu X, Maines TR, Smith C, Li Y, Donis RO, et al. Pathogenesis of avian influenza (H7) virus infection in mice and ferrets: enhanced virulence of Eurasian H7N7 viruses isolated from humans. J Virol. 2007;81(20):11139-47.

3. Belser JA, Wadford DA, Pappas C, Gustin KM, Maines TR, Pearce MB, et al. Pathogenesis of pandemic influenza A (H1N1) and triple-reassortant swine influenza A (H1) viruses in mice. J Virol. 2010;84(9):4194-203.

4. Bi Y, Xie Q, Zhang S, Li Y, Xiao H, Jin T, et al. Assessment of the internal genes of influenza A (H7N9) virus contributing to high pathogenicity in mice. J Virol. 2015;89(1):2-13.

5. Blazejewska P, Koscinski L, Viegas N, Anhlan D, Ludwig S, Schughart K. Pathogenicity of different PR8 influenza A virus variants in mice is determined by both viral and host factors. Virology. 2011;412(1):36-45.

6. Bodewes R, Geelhoed-Mieras MM, Nieuwkoop NJ, Hanson JA, David CS, Fouchier RA, et al. Redundancy of the influenza A virus-specific cytotoxic T lymphocyte response in HLA-B*2705 transgenic mice limits the impact of a mutation in the immunodominant NP(383-391) epitope on influenza pathogenesis. Virus Res. 2011;155(1):123-30.

7. Casalegno JS, Ferraris O, Escuret V, Bouscambert M, Bergeron C, Lines L, et al. Functional balance between the hemagglutinin and neuraminidase of influenza A(H1N1)pdm09 HA D222 variants. PLoS One. 2014;9(8):e104009.

8. Chen H, Bright RA, Subbarao K, Smith C, Cox NJ, Katz JM, et al. Polygenic virulence factors involved in pathogenesis of 1997 Hong Kong H5N1 influenza viruses in mice. Virus Res. 2007;128(1-2):159-63.

9. Choi WS, Baek YH, Kwon JJ, Jeong JH, Park SJ, Kim YI, et al. Rapid acquisition of polymorphic virulence markers during adaptation of highly pathogenic avian influenza H5N8 virus in the mouse. Sci Rep. 2017;7:40667.

10. Cline TD, Karlsson EA, Freiden P, Seufzer BJ, Rehg JE, Webby RJ, et al. Increased pathogenicity of a reassortant 2009 pandemic H1N1 influenza virus containing an H5N1 hemagglutinin. J Virol. 2011;85(23):12262-70.

11. Cox A, Baker SF, Nogales A, Martinez-Sobrido L, Dewhurst S. Development of a mouse-adapted live attenuated influenza virus that permits in vivo analysis of enhancements to the safety of live attenuated influenza virus vaccine. J Virol. 2015;89(6):3421-6.

12. de Jong RM, Stockhofe-Zurwieden N, Verheij ES, de Boer-Luijtze EA, Ruiter SJ, de Leeuw OS, et al. Rapid emergence of a virulent PB2 E627K variant during adaptation of highly pathogenic avian influenza H7N7 virus to mice. Virol J. 2013;10:276.

13. Driskell EA, Jones CA, Stallknecht DE, Howerth EW, Tompkins SM. Avian influenza virus isolates from wild birds replicate and cause disease in a mouse model of infection. Virology. 2010;399(2):280-9.

14. Elliott STC, Kallewaard NL, Benjamin E, Wachter-Rosati L, McAuliffe JM, Patel A, et al. DMAb inoculation of synthetic cross reactive antibodies protects against lethal influenza A and B infections. NPJ Vaccines. 2017;2:18.

15. Fan S, Deng G, Song J, Tian G, Suo Y, Jiang Y, et al. Two amino acid residues in the matrix protein M1 contribute to the virulence difference of H5N1 avian influenza viruses in mice. Virology. 2009;384(1):28-32.

16. Ferraris O, Escuret V, Bouscambert M, Casalegno JS, Jacquot F, Raoul H, et al. H1N1 influenza A virus neuraminidase modulates infectivity in mice. Antiviral Res. 2012;93(3):374-80.

17. Ferraris O, Casalegno JS, Frobert E, Bouscambert Duchamp M, Valette M, Jacquot F, et al. The NS Segment of H1N1pdm09 Enhances H5N1 Pathogenicity in a Mouse Model of Influenza Virus Infections. Viruses. 2018;10(9).

18. Gabriel G, Dauber B, Wolff T, Planz O, Klenk HD, Stech J. The viral polymerase mediates adaptation of an avian influenza virus to a mammalian host. Proc Natl Acad Sci U S A. 2005;102(51):18590-5.

19. Garigliany MM, Habyarimana A, Lambrecht B, Van de Paar E, Cornet A, van den Berg T, et al. Influenza A strain-dependent pathogenesis in fatal H1N1 and H5N1 subtype infections of mice. Emerg Infect Dis. 2010;16(4):595-603.

20. Hatesuer B, Bertram S, Mehnert N, Bahgat MM, Nelson PS, Pohlmann S, et al. Tmprss2 is essential for influenza H1N1 virus pathogenesis in mice. PLoS Pathog. 2013;9(12):e1003774.

21. Hatta M, Gao P, Halfmann P, Kawaoka Y. Molecular basis for high virulence of Hong Kong H5N1 influenza A viruses. Science (New York, NY). 2001;293(5536):1840-2.

22. Huo C, Jin Y, Zou S, Qi P, Xiao J, Tian H, et al. Lethal influenza A virus preferentially activates TLR3 and triggers a severe inflammatory response. Virus Res. 2018;257:102-12.

23. Ilyushina NA, Khalenkov AM, Seiler JP, Forrest HL, Bovin NV, Marjuki H, et al. Adaptation of pandemic H1N1 influenza viruses in mice. J Virol. 2010;84(17):8607-16.

24. Imai M, Watanabe T, Kiso M, Nakajima N, Yamayoshi S, Iwatsuki-Horimoto K, et al. A Highly Pathogenic Avian H7N9 Influenza Virus Isolated from A Human Is Lethal in Some Ferrets Infected via Respiratory Droplets. Cell Host Microbe. 2017;22(5):615-26 e8.

25. Itoh Y, Shinya K, Kiso M, Watanabe T, Sakoda Y, Hatta M, et al. In vitro and in vivo characterization of new swine-origin H1N1 influenza viruses. Nature. 2009;460(7258):1021-5.

26. Jang YH, Byun YH, Lee DH, Lee KH, Lee YJ, Lee YH, et al. Cold-adapted X-31 live attenuated 2009 pandemic H1N1 influenza vaccine elicits protective immune responses in mice and ferrets. Vaccine. 2013;31(9):1320-7.

27. Jang YH, Jung EJ, Byun YH, Lee KH, Lee EY, Lee YJ, et al. Immunogenicity and protective efficacy of cold-adapted X-31 live attenuated pre-pandemic H5N1 influenza vaccines. Vaccine. 2013;31(33):3339-46.

28. Jang YH, Lee EY, Byun YH, Jung EJ, Lee YJ, Lee YH, et al. Protective efficacy in mice of monovalent and trivalent live attenuated influenza vaccines in the background of cold-adapted A/X-31 and B/Lee/40 donor strains. Vaccine. 2014;32(5):535-43.

29. Jang YH, Kim JY, Byun YH, Son A, Lee JY, Lee YJ, et al. Pan-Influenza A Protection by Prime-Boost Vaccination with Cold-Adapted Live-Attenuated Influenza Vaccine in a Mouse Model. Front Immunol. 2018;9:116.

30. Jiao P, Tian G, Li Y, Deng G, Jiang Y, Liu C, et al. A single-amino-acid substitution in the NS1 protein changes the pathogenicity of H5N1 avian influenza viruses in mice. J Virol. 2008;82(3):1146-54.

31. Joseph T, McAuliffe J, Lu B, Jin H, Kemble G, Subbarao K. Evaluation of replication and pathogenicity of avian influenza a H7 subtype viruses in a mouse model. J Virol. 2007;81(19):10558-66.

32. Katz JM, Lu X, Tumpey TM, Smith CB, Shaw MW, Subbarao K. Molecular correlates of influenza A H5N1 virus pathogenesis in mice. J Virol. 2000;74(22):10807-10.

33. Kim JI, Park S, Lee S, Lee I, Heo J, Hwang MW, et al. DBA/2 mouse as an animal model for anti-influenza drug efficacy evaluation. J Microbiol. 2013;51(6):866-71.

34. Kobasa D, Takada A, Shinya K, Hatta M, Halfmann P, Theriault S, et al. Enhanced virulence of influenza A viruses with the haemagglutinin of the 1918 pandemic virus. Nature. 2004;431(7009):703-7.

35. Kwon HI, Kim EH, Kim YI, Park SJ, Si YJ, Lee IW, et al. Comparison of the pathogenic potential of highly pathogenic avian influenza (HPAI) H5N6, and H5N8 viruses isolated in South Korea during the 2016-2017 winter season. Emerg Microbes Infect. 2018;7(1):29.

36. Lee MS, Jang EY, Cho J, Kim K, Lee CH, Yi H. Development and comparison of two H5N8 influenza A vaccine candidate strains. Arch Virol. 2018.

37. Leist SR, Pilzner C, van den Brand JM, Dengler L, Geffers R, Kuiken T, et al. Influenza H3N2 infection of the collaborative cross founder strains reveals highly divergent host responses and identifies a unique phenotype in CAST/EiJ mice. BMC Genomics. 2016;17:143.

38. Li Z, Chen H, Jiao P, Deng G, Tian G, Li Y, et al. Molecular basis of replication of duck H5N1 influenza viruses in a mammalian mouse model. J Virol. 2005;79(18):12058-64.

39. Liedmann S, Hrincius ER, Anhlan D, McCullers JA, Ludwig S, Ehrhardt C. New virulence determinants contribute to the enhanced immune response and reduced virulence of an influenza A virus A/PR8/34 variant. The Journal of infectious diseases. 2014;209(4):532-41.

40. Long JX, Peng DX, Liu YL, Wu YT, Liu XF. Virulence of H5N1 avian influenza virus enhanced by a 15-nucleotide deletion in the viral nonstructural gene. Virus Genes. 2008;36(3):471-8.

41. Lu X, Tumpey TM, Morken T, Zaki SR, Cox NJ, Katz JM. A mouse model for the evaluation of pathogenesis and immunity to influenza A (H5N1) viruses isolated from humans. J Virol. 1999;73(7):5903-11.

42. Lu S, Zhao Z, Zhang J, Wang W, He X, Yu M, et al. Genetics, pathogenicity and transmissibility of novel reassortant H5N6 highly pathogenic avian influenza viruses first isolated from migratory birds in western China. Emerg Microbes Infect. 2018;7(1):6.

43. Maines TR, Lu XH, Erb SM, Edwards L, Guarner J, Greer PW, et al. Avian influenza (H5N1) viruses isolated from humans in Asia in 2004 exhibit increased virulence in mammals. J Virol. 2005;79(18):11788-800.

44. Manicassamy B, Medina RA, Hai R, Tsibane T, Stertz S, Nistal-Villan E, et al. Protection of mice against lethal challenge with 2009 H1N1 influenza A virus by 1918-like and classical swine H1N1 based vaccines. PLoS Pathog. 2010;6(1):e1000745.

45. Mase M, Eto M, Tanimura N, Imai K, Tsukamoto K, Horimoto T, et al. Isolation of a genotypically unique H5N1 influenza virus from duck meat imported into Japan from China. Virology. 2005;339(1):101-9.

46. Metreveli G, Gao Q, Mena I, Schmolke M, Berg M, Albrecht RA, et al. The origin of the PB1 segment of swine influenza A virus subtype H1N2 determines viral pathogenicity in mice. Virus Res. 2014;188:97-102.

47. Mifsud EJ, Tan AC, Brown LE, Chua BY, Jackson DC. Generation of Adaptive Immune Responses Following Influenza Virus Challenge is Not Compromised by Pre-Treatment with the TLR-2 Agonist Pam2Cys. Front Immunol. 2015;6:290.

48. Na W, Lyoo KS, Yoon SW, Yeom M, Kang B, Moon H, et al. Attenuation of the virulence of a recombinant influenza virus expressing the naturally truncated NS gene from an H3N8 equine influenza virus in mice. Vet Res. 2016;47(1):115.

49. Nurnberger C, Zimmermann V, Gerhardt M, Staeheli P. Influenza Virus Susceptibility of Wild-Derived CAST/EiJ Mice Results from Two Amino Acid Changes in the MX1 Restriction Factor. J Virol. 2016;90(23):10682-92.

50. O'Neill E, Krauss SL, Riberdy JM, Webster RG, Woodland DL. Heterologous protection against lethal A/HongKong/156/97 (H5N1) influenza virus infection in C57BL/6 mice. J Gen Virol. 2000;81(Pt 11):2689-96.

51. Otte A, Sauter M, Alleva L, Baumgarte S, Klingel K, Gabriel G. Differential host determinants contribute to the pathogenesis of 2009 pandemic H1N1 and human H5N1 influenza A viruses in experimental mouse models. Am J Pathol. 2011;179(1):230-9.

52. Otte A, Sauter M, Daxer MA, McHardy AC, Klingel K, Gabriel G. Adaptive Mutations That Occurred during Circulation in Humans of H1N1 Influenza Virus in the 2009 Pandemic Enhance Virulence in Mice. J Virol. 2015;89(14):7329-37.

53. Pan W, Xie H, Li X, Guan W, Chen P, Zhang B, et al. Patient-derived avian influenza A (H5N6) virus is highly pathogenic in mice but can be effectively treated by anti-influenza polyclonal antibodies. Emerg Microbes Infect. 2018;7(1):107.

54. Pica N, Iyer A, Ramos I, Bouvier NM, Fernandez-Sesma A, Garcia-Sastre A, et al. The DBA.2 mouse is susceptible to disease following infection with a broad, but limited, range of influenza A and B viruses. J Virol. 2011;85(23):12825-9.

55. Ping J, Keleta L, Forbes NE, Dankar S, Stecho W, Tyler S, et al. Genomic and protein structural maps of adaptive evolution of human influenza A virus to increased virulence in the mouse. PLoS One. 2011;6(6):e21740.

56. Ping X, Hu W, Xiong R, Zhang X, Teng Z, Ding M, et al. Generation of a broadly reactive influenza H1 antigen using a consensus HA sequence. Vaccine. 2018;36(32 Pt B):4837-45.

57. Qi L, Kash JC, Dugan VG, Wang R, Jin G, Cunningham RE, et al. Role of sialic acid binding specificity of the 1918 influenza virus hemagglutinin protein in virulence and pathogenesis for mice. J Virol. 2009;83(8):3754-61.

58. Qi L, Davis AS, Jagger BW, Schwartzman LM, Dunham EJ, Kash JC, et al. Analysis by single-gene reassortment demonstrates that the 1918 influenza virus is functionally compatible with a low-pathogenicity avian influenza virus in mice. J Virol. 2012;86(17):9211-20.

59. Qi W, Jia W, Liu D, Li J, Bi Y, Xie S, et al. Emergence and Adaptation of a Novel Highly Pathogenic H7N9 Influenza Virus in Birds and Humans from a 2013 Human-Infecting Low-Pathogenic Ancestor. J Virol. 2018;92(2).

60. Quan FS, Steinhauer D, Huang C, Ross TM, Compans RW, Kang SM. A bivalent influenza VLP vaccine confers complete inhibition of virus replication in lungs. Vaccine. 2008;26(26):3352-61.

61. Rodriguez A, Falcon A, Cuevas MT, Pozo F, Guerra S, Garcia-Barreno B, et al. Characterization in vitro and in vivo of a pandemic H1N1 influenza virus from a fatal case. PLoS One. 2013;8(1):e53515.

62. Shi J, Deng G, Kong H, Gu C, Ma S, Yin X, et al. H7N9 virulent mutants detected in chickens in China pose an increased threat to humans. Cell Res. 2017;27(12):1409-21.

63. Smee DF, von Itzstein M, Bhatt B, Tarbet EB. Exacerbation of influenza virus infections in mice by intranasal treatments and implications for evaluation of antiviral drugs. Antimicrob Agents Chemother. 2012;56(12):6328-33.

64. Song MS, Pascua PN, Lee JH, Baek YH, Lee OJ, Kim CJ, et al. The polymerase acidic protein gene of influenza a virus contributes to pathogenicity in a mouse model. J Virol. 2009;83(23):12325-35.

65. Song MS, Hee Baek Y, Kim EH, Park SJ, Kim S, Lim GJ, et al. Increased virulence of neuraminidase inhibitor-resistant pandemic H1N1 virus in mice: potential emergence of drug-resistant and virulent variants. Virulence. 2013;4(6):489-93.

66. Srivastava B, Blazejewska P, Hessmann M, Bruder D, Geffers R, Mauel S, et al. Host genetic background strongly influences the response to influenza a virus infections. PLoS One. 2009;4(3):e4857.

67. Sun H, Pu J, Wei Y, Sun Y, Hu J, Liu L, et al. Highly Pathogenic Avian Influenza H5N6 Viruses Exhibit Enhanced Affinity for Human Type Sialic Acid Receptor and In-Contact Transmission in Model Ferrets. J Virol. 2016;90(14):6235-43.

68. Sutton TC, Chakraborty S, Mallajosyula VVA, Lamirande EW, Ganti K, Bock KW, et al. Protective efficacy of influenza group 2 hemagglutinin stem-fragment immunogen vaccines. NPJ Vaccines. 2017;2:35.

69. Tate MD, Ioannidis LJ, Croker B, Brown LE, Brooks AG, Reading PC. The role of neutrophils during mild and severe influenza virus infections of mice. PLoS One. 2011;6(3):e17618.

70. Tate MD, Brooks AG, Reading PC. Specific sites of N-linked glycosylation on the hemagglutinin of H1N1 subtype influenza A virus determine sensitivity to inhibitors of the innate immune system and virulence in mice. J Immunol. 2011;187(4):1884-94.

71. Tumpey TM, Basler CF, Aguilar PV, Zeng H, Solorzano A, Swayne DE, et al. Characterization of the reconstructed 1918 Spanish influenza pandemic virus. Science (New York, NY). 2005;310(5745):77-80.

72. Vasilijevic J, Zamarreno N, Oliveros JC, Rodriguez-Frandsen A, Gomez G, Rodriguez G, et al. Reduced accumulation of defective viral genomes contributes to severe outcome in influenza virus infected patients. PLoS Pathog. 2017;13(10):e1006650.

73. Wang X, Zeng Z, Zhang Z, Zheng Y, Li B, Su G, et al. The Appropriate Combination of Hemagglutinin and Neuraminidase Prompts the Predominant H5N6 Highly Pathogenic Avian Influenza Virus in Birds. Front Microbiol. 2018;9:1088.

74. Yang W, Yin X, Guan L, Li M, Ma S, Shi J, et al. A live attenuated vaccine prevents replication and transmission of H7N9 highly pathogenic influenza viruses in mammals. Emerg Microbes Infect. 2018;7(1):153.

75. Ye J, Sorrell EM, Cai Y, Shao H, Xu K, Pena L, et al. Variations in the hemagglutinin of the 2009 H1N1 pandemic virus: potential for strains with altered virulence phenotype? PLoS Pathog. 2010;6(10):e1001145.

76. Yu Y, Zhang Z, Li H, Wang X, Li B, Ren X, et al. Biological Characterizations of H5Nx Avian Influenza Viruses Embodying Different Neuraminidases. Front Microbiol. 2017;8:1084.

77. Yu Z, Cheng K, Sun W, Zhang X, Xia X, Gao Y. PB2 and HA mutations increase the virulence of highly pathogenic H5N5 clade 2.3.4.4 avian influenza virus in mice. Arch Virol. 2018;163(2):401-10.

78. Yu Z, Cheng K, Sun W, Zhang X, Xia X, Gao Y. Multiple adaptive amino acid substitutions increase the virulence of a wild waterfowl-origin reassortant H5N8 avian influenza virus in mice. Virus Res. 2018;244:13-20.

79. Zhang Q, Shi J, Deng G, Guo J, Zeng X, He X, et al. H7N9 influenza viruses are transmissible in ferrets by respiratory droplet. Science (New York, NY). 2013;341(6144):410-4.

80. Zhou B, Pearce MB, Li Y, Wang J, Mason RJ, Tumpey TM, et al. Asparagine substitution at PB2 residue 701 enhances the replication, pathogenicity, and transmission of the 2009 pandemic H1N1 influenza A virus. PLoS One. 2013;8(6):e67616.

81. Zhou K, Wang J, Li A, Zhao W, Wang D, Zhang W, et al. Swift and Strong NK Cell Responses Protect 129 Mice against High-Dose Influenza Virus Infection. J Immunol. 2016;196(4):1842-54.

82. Zhu Z, Yang Y, Feng Y, Shi B, Chen L, Zheng Y, et al. Infection of inbred BALB/c and C57BL/6 and outbred Institute of Cancer Research mice with the emerging H7N9 avian influenza virus. Emerg Microbes Infect. 2013;2(8):e50.

83. Zhu W, Li L, Yan Z, Gan T, Li L, Chen R, et al. Dual E627K and D701N mutations in the PB2 protein of A(H7N9) influenza virus increased its virulence in mammalian models. Sci Rep. 2015;5:14170.

84. Zhu W, Zhang H, Xiang X, Zhong L, Yang L, Guo J, et al. Reassortant Eurasian Avian-Like Influenza A(H1N1) Virus from a Severely Ill Child, Hunan Province, China, 2015. Emerg Infect Dis. 2016;22(11):1930-6.
