# Supplementary material for: Rule-based meta-analysis reveals the major role of PB2 in influencing influenza A virus virulence in mice
Source: BMC Genomics. 2019 Dec 24;20(Suppl 9):973. doi: 10.1186/s12864-019-6295-8 (PMC6929465; doi:10.1186/s12864-019-6295-8)
Supplement: Supplementary file 8 — Additional file 8: Table S4. Metadata of IAV nucleotide sequences used in this study (with supplementary references). [file 12864_2019_6295_MOESM8_ESM.docx]

**Table S4.** Metadata of IAV nucleotide sequences (ordered by genome sets of IAV strains) used in this study. Information on extrapolated IAV genomes and partial sequences can be found in **Table S7** and **S8** (**Additional file 11** and **12**), respectively.

| **Genome ID** | **Host** | **Sequence ID** | **Sources** | **Influenza strain** | **Version** | **Segment** | **Extrapolation** |
| --- | --- | --- | --- | --- | --- | --- | --- |
|  |  |  |  |  |  |  |  |
| 673 | Duck | EPI1208375 | GISAID | A/duck/Guangdong/673/2014(H5N6) | 1 | 4 | No extrapolation |
| 673 | Duck | EPI980834 | GISAID | A/duck/Guangdong/673/2014(H5N6) | 1 | 6 | No extrapolation |
|  |  |  |  |  |  |  |  |
| 674 | Goose | EPI1208376 | GISAID | A/goose/Guangdong/674/2014(H5N6) | 1 | 4 | No extrapolation |
| 674 | Goose | EPI980835 | GISAID | A/goose/Guangdong/674/2014(H5N6) | 1 | 6 | No extrapolation |
|  |  |  |  |  |  |  |  |
| AH1 | Human | EPI439504 | GISAID | A/Anhui/1/2013(H7N9) | 1 | 1 | No extrapolation |
| AH1 | Human | EPI439508 | GISAID | A/Anhui/1/2013(H7N9) | 1 | 2 | No extrapolation |
| AH1 | Human | EPI439503 | GISAID | A/Anhui/1/2013(H7N9) | 1 | 3 | No extrapolation |
| AH1 | Human | EPI439507 | GISAID | A/Anhui/1/2013(H7N9) | 1 | 4 | No extrapolation |
| AH1 | Human | EPI439505 | GISAID | A/Anhui/1/2013(H7N9) | 1 | 5 | No extrapolation |
| AH1 | Human | EPI439509 | GISAID | A/Anhui/1/2013(H7N9) | 1 | 6 | No extrapolation |
| AH1 | Human | EPI439506 | GISAID | A/Anhui/1/2013(H7N9) | 1 | 7 | No extrapolation |
| AH1 | Human | EPI439510 | GISAID | A/Anhui/1/2013(H7N9) | 1 | 8 | No extrapolation |
|  |  |  |  |  |  |  |  |
| ALB76v1 | Duck | GU186639 | GenBank | A/duck/Alberta/35/1976(H1N1) | 1 | 1 | No extrapolation |
| ALB76v1 | Duck | GU186638 | GenBank | A/duck/Alberta/35/1976(H1N1) | 1 | 2 | No extrapolation |
| ALB76v1 | Duck | GU186637 | GenBank | A/duck/Alberta/35/1976(H1N1) | 1 | 3 | No extrapolation |
| ALB76v1 | Duck | GU186632 | GenBank | A/duck/Alberta/35/1976(H1N1) | 1 | 4 | No extrapolation |
| ALB76v1 | Duck | GU186635 | GenBank | A/duck/Alberta/35/1976(H1N1) | 1 | 5 | No extrapolation |
| ALB76v1 | Duck | GU186634 | GenBank | A/duck/Alberta/35/1976(H1N1) | 1 | 6 | No extrapolation |
| ALB76v1 | Duck | GU186633 | GenBank | A/duck/Alberta/35/1976(H1N1) | 1 | 7 | No extrapolation |
| ALB76v1 | Duck | GU186636 | GenBank | A/duck/Alberta/35/1976(H1N1) | 1 | 8 | No extrapolation |
|  |  |  |  |  |  |  |  |
| ALB76v2 | Duck | CY130013 | GenBank | A/duck/Alberta/35/1976(H1N1) | 2 | 1 | No extrapolation |
| ALB76v2 | Duck | CY130012 | GenBank | A/duck/Alberta/35/1976(H1N1) | 2 | 2 | No extrapolation |
| ALB76v2 | Duck | CY130011 | GenBank | A/duck/Alberta/35/1976(H1N1) | 2 | 3 | No extrapolation |
| ALB76v2 | Duck | CY130006 | GenBank | A/duck/Alberta/35/1976(H1N1) | 2 | 4 | No extrapolation |
| ALB76v2 | Duck | CY130009 | GenBank | A/duck/Alberta/35/1976(H1N1) | 2 | 5 | No extrapolation |
| ALB76v2 | Duck | CY130008 | GenBank | A/duck/Alberta/35/1976(H1N1) | 2 | 6 | No extrapolation |
| ALB76v2 | Duck | CY130007 | GenBank | A/duck/Alberta/35/1976(H1N1) | 2 | 7 | No extrapolation |
| ALB76v2 | Duck | CY130010 | GenBank | A/duck/Alberta/35/1976(H1N1) | 2 | 8 | No extrapolation |
|  |  |  |  |  |  |  |  |
| BDKB14 | Avian | KJ413839 | GenBank | A/broilerduck/Korea/Buan2/2014(H5N8) | 1 | 1 | No extrapolation |
| BDKB14 | Avian | KJ413840 | GenBank | A/broilerduck/Korea/Buan2/2014(H5N8) | 1 | 2 | No extrapolation |
| BDKB14 | Avian | KJ413841 | GenBank | A/broilerduck/Korea/Buan2/2014(H5N8) | 1 | 3 | No extrapolation |
| BDKB14 | Avian | KJ413842 | GenBank | A/broilerduck/Korea/Buan2/2014(H5N8) | 1 | 4 | No extrapolation |
| BDKB14 | Avian | KJ413843 | GenBank | A/broilerduck/Korea/Buan2/2014(H5N8) | 1 | 5 | No extrapolation |
| BDKB14 | Avian | KJ413844 | GenBank | A/broilerduck/Korea/Buan2/2014(H5N8) | 1 | 6 | No extrapolation |
| BDKB14 | Avian | KJ413845 | GenBank | A/broilerduck/Korea/Buan2/2014(H5N8) | 1 | 7 | No extrapolation |
| BDKB14 | Avian | KJ413846 | GenBank | A/broilerduck/Korea/Buan2/2014(H5N8) | 1 | 8 | No extrapolation |
|  |  |  |  |  |  |  |  |
| BEL05 | Avian | EPI229704 | GISAID | A/crestedeagle/Belgium/1/2004(H5N1) | 1 | 1 | No extrapolation |
| BEL05 | Avian | EPI229700 | GISAID | A/crestedeagle/Belgium/1/2004(H5N1) | 1 | 2 | No extrapolation |
| BEL05 | Avian | EPI229701 | GISAID | A/crestedeagle/Belgium/1/2004(H5N1) | 1 | 3 | No extrapolation |
| BEL05 | Avian | EPI229698 | GISAID | A/crestedeagle/Belgium/1/2004(H5N1) | 1 | 4 | No extrapolation |
| BEL05 | Avian | EPI229702 | GISAID | A/crestedeagle/Belgium/1/2004(H5N1) | 1 | 5 | No extrapolation |
| BEL05 | Avian | EPI229697 | GISAID | A/crestedeagle/Belgium/1/2004(H5N1) | 1 | 6 | No extrapolation |
| BEL05 | Avian | EPI229699 | GISAID | A/crestedeagle/Belgium/1/2004(H5N1) | 1 | 7 | No extrapolation |
| BEL05 | Avian | EPI229703 | GISAID | A/crestedeagle/Belgium/1/2004(H5N1) | 1 | 8 | No extrapolation |
|  |  |  |  |  |  |  |  |
| BJ89v1 | Human | DQ508830 | GenBank | A/Beijing/353/1989(H3N2) | 1 | 1 | No extrapolation |
| BJ89v1 | Human | DQ508831 | GenBank | A/Beijing/353/1989(H3N2) | 1 | 2 | No extrapolation |
| BJ89v1 | Human | DQ508832 | GenBank | A/Beijing/353/1989(H3N2) | 1 | 3 | No extrapolation |
| BJ89v1 | Human | DQ508833 | GenBank | A/Beijing/353/1989(H3N2) | 1 | 4 | No extrapolation |
| BJ89v1 | Human | DQ508834 | GenBank | A/Beijing/353/1989(H3N2) | 1 | 5 | No extrapolation |
| BJ89v1 | Human | DQ508835 | GenBank | A/Beijing/353/1989(H3N2) | 1 | 6 | No extrapolation |
| BJ89v1 | Human | DQ508836 | GenBank | A/Beijing/353/1989(H3N2) | 1 | 7 | No extrapolation |
| BJ89v1 | Human | DQ508837 | GenBank | A/Beijing/353/1989(H3N2) | 1 | 8 | No extrapolation |
|  |  |  |  |  |  |  |  |
| BJ89v2 | Human | CY121031 | GenBank | A/Beijing/353/1989(H3N2) | 2 | 1 | No extrapolation |
| BJ89v2 | Human | CY121030 | GenBank | A/Beijing/353/1989(H3N2) | 2 | 2 | No extrapolation |
| BJ89v2 | Human | CY121029 | GenBank | A/Beijing/353/1989(H3N2) | 2 | 3 | No extrapolation |
| BJ89v2 | Human | CY121024 | GenBank | A/Beijing/353/1989(H3N2) | 2 | 4 | No extrapolation |
| BJ89v2 | Human | CY121027 | GenBank | A/Beijing/353/1989(H3N2) | 2 | 5 | No extrapolation |
| BJ89v2 | Human | CY121026 | GenBank | A/Beijing/353/1989(H3N2) | 2 | 6 | No extrapolation |
| BJ89v2 | Human | CY121025 | GenBank | A/Beijing/353/1989(H3N2) | 2 | 7 | No extrapolation |
| BJ89v2 | Human | CY121028 | GenBank | A/Beijing/353/1989(H3N2) | 2 | 8 | No extrapolation |
|  |  |  |  |  |  |  |  |
| BJ89v3 | Human | CY114468 | GenBank | A/Beijing/353/1989(H3N2) | 3 | 1 | No extrapolation |
| BJ89v3 | Human | CY114467 | GenBank | A/Beijing/353/1989(H3N2) | 3 | 2 | No extrapolation |
| BJ89v3 | Human | CY114466 | GenBank | A/Beijing/353/1989(H3N2) | 3 | 3 | No extrapolation |
| BJ89v3 | Human | CY114461 | GenBank | A/Beijing/353/1989(H3N2) | 3 | 4 | No extrapolation |
| BJ89v3 | Human | CY114464 | GenBank | A/Beijing/353/1989(H3N2) | 3 | 5 | No extrapolation |
| BJ89v3 | Human | CY114463 | GenBank | A/Beijing/353/1989(H3N2) | 3 | 6 | No extrapolation |
| BJ89v3 | Human | CY114462 | GenBank | A/Beijing/353/1989(H3N2) | 3 | 7 | No extrapolation |
| BJ89v3 | Human | CY114465 | GenBank | A/Beijing/353/1989(H3N2) | 3 | 8 | No extrapolation |
|  |  |  |  |  |  |  |  |
| BRAZ78 | Human | CY020300 | GenBank | A/Brazil/11/1978(H1N1) | 1 | 1 | No extrapolation |
| BRAZ78 | Human | CY020299 | GenBank | A/Brazil/11/1978(H1N1) | 1 | 2 | No extrapolation |
| BRAZ78 | Human | CY020298 | GenBank | A/Brazil/11/1978(H1N1) | 1 | 3 | No extrapolation |
| BRAZ78 | Human | CY020293 | GenBank | A/Brazil/11/1978(H1N1) | 1 | 4 | No extrapolation |
| BRAZ78 | Human | CY020296 | GenBank | A/Brazil/11/1978(H1N1) | 1 | 5 | No extrapolation |
| BRAZ78 | Human | CY020295 | GenBank | A/Brazil/11/1978(H1N1) | 1 | 6 | No extrapolation |
| BRAZ78 | Human | CY020294 | GenBank | A/Brazil/11/1978(H1N1) | 1 | 7 | No extrapolation |
| BRAZ78 | Human | CY020297 | GenBank | A/Brazil/11/1978(H1N1) | 1 | 8 | No extrapolation |
|  |  |  |  |  |  |  |  |
| BRIS10v1 | Human | CY035029 | GenBank | A/Brisbane/10/2007(H3N2) | 1 | 1 | No extrapolation |
| BRIS10v1 | Human | CY035028 | GenBank | A/Brisbane/10/2007(H3N2) | 1 | 2 | No extrapolation |
| BRIS10v1 | Human | CY035027 | GenBank | A/Brisbane/10/2007(H3N2) | 1 | 3 | No extrapolation |
| BRIS10v1 | Human | CY035022 | GenBank | A/Brisbane/10/2007(H3N2) | 1 | 4 | No extrapolation |
| BRIS10v1 | Human | CY035025 | GenBank | A/Brisbane/10/2007(H3N2) | 1 | 5 | No extrapolation |
| BRIS10v1 | Human | CY035024 | GenBank | A/Brisbane/10/2007(H3N2) | 1 | 6 | No extrapolation |
| BRIS10v1 | Human | CY035023 | GenBank | A/Brisbane/10/2007(H3N2) | 1 | 7 | No extrapolation |
| BRIS10v1 | Human | CY035026 | GenBank | A/Brisbane/10/2007(H3N2) | 1 | 8 | No extrapolation |
|  |  |  |  |  |  |  |  |
| BRIS10v2 | Human | KJ609211 | GenBank | A/Brisbane/10/2007(H3N2) | 2 | 1 | No extrapolation |
| BRIS10v2 | Human | KJ609212 | GenBank | A/Brisbane/10/2007(H3N2) | 2 | 2 | No extrapolation |
| BRIS10v2 | Human | KJ609213 | GenBank | A/Brisbane/10/2007(H3N2) | 2 | 3 | No extrapolation |
| BRIS10v2 | Human | KJ609214 | GenBank | A/Brisbane/10/2007(H3N2) | 2 | 4 | No extrapolation |
| BRIS10v2 | Human | KJ609215 | GenBank | A/Brisbane/10/2007(H3N2) | 2 | 5 | No extrapolation |
| BRIS10v2 | Human | KJ609216 | GenBank | A/Brisbane/10/2007(H3N2) | 2 | 6 | No extrapolation |
| BRIS10v2 | Human | KJ609217 | GenBank | A/Brisbane/10/2007(H3N2) | 2 | 7 | No extrapolation |
| BRIS10v2 | Human | KJ609218 | GenBank | A/Brisbane/10/2007(H3N2) | 2 | 8 | No extrapolation |
|  |  |  |  |  |  |  |  |
| BRIS59 | Human | CY058484 | GenBank | A/Brisbane/59/2007(H1N1) | 1 | 1 | No extrapolation |
| BRIS59 | Human | CY058485 | GenBank | A/Brisbane/59/2007(H1N1) | 1 | 2 | No extrapolation |
| BRIS59 | Human | CY058486 | GenBank | A/Brisbane/59/2007(H1N1) | 1 | 3 | No extrapolation |
| BRIS59 | Human | CY058487 | GenBank | A/Brisbane/59/2007(H1N1) | 1 | 4 | No extrapolation |
| BRIS59 | Human | CY058488 | GenBank | A/Brisbane/59/2007(H1N1) | 1 | 5 | No extrapolation |
| BRIS59 | Human | CY058489 | GenBank | A/Brisbane/59/2007(H1N1) | 1 | 6 | No extrapolation |
| BRIS59 | Human | CY058490 | GenBank | A/Brisbane/59/2007(H1N1) | 1 | 7 | No extrapolation |
| BRIS59 | Human | CY058491 | GenBank | A/Brisbane/59/2007(H1N1) | 1 | 8 | No extrapolation |
|  |  |  |  |  |  |  |  |
| CA04 | Human | FJ969516 | GenBank | A/California/04/2009(H1N1) | 1 | 1 | No extrapolation |
| CA04 | Human | GQ377049 | GenBank | A/California/04/2009(H1N1) | 1 | 2 | No extrapolation |
| CA04 | Human | FJ969515 | GenBank | A/California/04/2009(H1N1) | 1 | 3 | No extrapolation |
| CA04 | Human | GQ117044 | GenBank | A/California/04/2009(H1N1) | 1 | 4 | No extrapolation |
| CA04 | Human | FJ969512 | GenBank | A/California/04/2009(H1N1) | 1 | 5 | No extrapolation |
| CA04 | Human | FJ969517 | GenBank | A/California/04/2009(H1N1) | 1 | 6 | No extrapolation |
| CA04 | Human | FJ969513 | GenBank | A/California/04/2009(H1N1) | 1 | 7 | No extrapolation |
| CA04 | Human | FJ969514 | GenBank | A/California/04/2009(H1N1) | 1 | 8 | No extrapolation |
|  |  |  |  |  |  |  |  |
| CA09v1 | Human | CY266198 | GenBank | A/California/07/2009(H1N1) | 1 | 1 | No extrapolation |
| CA09v1 | Human | CY266197 | GenBank | A/California/07/2009(H1N1) | 1 | 2 | No extrapolation |
| CA09v1 | Human | CY266196 | GenBank | A/California/07/2009(H1N1) | 1 | 3 | No extrapolation |
| CA09v1 | Human | CY266191 | GenBank | A/California/07/2009(H1N1) | 1 | 4 | No extrapolation |
| CA09v1 | Human | CY266194 | GenBank | A/California/07/2009(H1N1) | 1 | 5 | No extrapolation |
| CA09v1 | Human | CY266193 | GenBank | A/California/07/2009(H1N1) | 1 | 6 | No extrapolation |
| CA09v1 | Human | CY266192 | GenBank | A/California/07/2009(H1N1) | 1 | 7 | No extrapolation |
| CA09v1 | Human | CY266195 | GenBank | A/California/07/2009(H1N1) | 1 | 8 | No extrapolation |
|  |  |  |  |  |  |  |  |
| CA09v2 | Human | NC026438 | GenBank | A/California/07/2009(H1N1) | 2 | 1 | No extrapolation |
| CA09v2 | Human | NC026435 | GenBank | A/California/07/2009(H1N1) | 2 | 2 | No extrapolation |
| CA09v2 | Human | NC026437 | GenBank | A/California/07/2009(H1N1) | 2 | 3 | No extrapolation |
| CA09v2 | Human | NC026433 | GenBank | A/California/07/2009(H1N1) | 2 | 4 | No extrapolation |
| CA09v2 | Human | NC026436 | GenBank | A/California/07/2009(H1N1) | 2 | 5 | No extrapolation |
| CA09v2 | Human | NC026434 | GenBank | A/California/07/2009(H1N1) | 2 | 6 | No extrapolation |
| CA09v2 | Human | NC026431 | GenBank | A/California/07/2009(H1N1) | 2 | 7 | No extrapolation |
| CA09v2 | Human | NC026432 | GenBank | A/California/07/2009(H1N1) | 2 | 8 | No extrapolation |
|  |  |  |  |  |  |  |  |
| CCT03 | Avian | EU743002 | GenBank | A/chicken/CT/260413-2/2003(H7N2) | 1 | 1 | No extrapolation |
| CCT03 | Avian | EU743001 | GenBank | A/chicken/CT/260413-2/2003(H7N2) | 1 | 2 | No extrapolation |
| CCT03 | Avian | EU743000 | GenBank | A/chicken/CT/260413-2/2003(H7N2) | 1 | 3 | No extrapolation |
| CCT03 | Avian | EU742995 | GenBank | A/chicken/CT/260413-2/2003(H7N2) | 1 | 4 | No extrapolation |
| CCT03 | Avian | EU742998 | GenBank | A/chicken/CT/260413-2/2003(H7N2) | 1 | 5 | No extrapolation |
| CCT03 | Avian | EU742997 | GenBank | A/chicken/CT/260413-2/2003(H7N2) | 1 | 6 | No extrapolation |
| CCT03 | Avian | EU742996 | GenBank | A/chicken/CT/260413-2/2003(H7N2) | 1 | 7 | No extrapolation |
| CCT03 | Avian | EU742999 | GenBank | A/chicken/CT/260413-2/2003(H7N2) | 1 | 8 | No extrapolation |
|  |  |  |  |  |  |  |  |
| CG17 | Avian | MF630034 | GenBank | A/chicken/Guangdong/SD008/2017(H7N9) | 1 | 1 | No extrapolation |
| CG17 | Avian | MF630035 | GenBank | A/chicken/Guangdong/SD008/2017(H7N9) | 1 | 2 | No extrapolation |
| CG17 | Avian | MF630036 | GenBank | A/chicken/Guangdong/SD008/2017(H7N9) | 1 | 3 | No extrapolation |
| CG17 | Avian | MF630037 | GenBank | A/chicken/Guangdong/SD008/2017(H7N9) | 1 | 4 | No extrapolation |
| CG17 | Avian | MF630038 | GenBank | A/chicken/Guangdong/SD008/2017(H7N9) | 1 | 5 | No extrapolation |
| CG17 | Avian | MF630039 | GenBank | A/chicken/Guangdong/SD008/2017(H7N9) | 1 | 6 | No extrapolation |
| CG17 | Avian | MF630040 | GenBank | A/chicken/Guangdong/SD008/2017(H7N9) | 1 | 7 | No extrapolation |
| CG17 | Avian | MF630041 | GenBank | A/chicken/Guangdong/SD008/2017(H7N9) | 1 | 8 | No extrapolation |
|  |  |  |  |  |  |  |  |
| CH04 | Avian | AY950279 | GenBank | A/chicken/Henan/01/2004(H5N1) | 1 | 1 | No extrapolation |
| CH04 | Avian | AY950272 | GenBank | A/chicken/Henan/01/2004(H5N1) | 1 | 2 | No extrapolation |
| CH04 | Avian | AY950265 | GenBank | A/chicken/Henan/01/2004(H5N1) | 1 | 3 | No extrapolation |
| CH04 | Avian | AY950230 | GenBank | A/chicken/Henan/01/2004(H5N1) | 1 | 4 | No extrapolation |
| CH04 | Avian | AY950251 | GenBank | A/chicken/Henan/01/2004(H5N1) | 1 | 5 | No extrapolation |
| CH04 | Avian | AY950244 | GenBank | A/chicken/Henan/01/2004(H5N1) | 1 | 6 | No extrapolation |
| CH04 | Avian | AY950237 | GenBank | A/chicken/Henan/01/2004(H5N1) | 1 | 7 | No extrapolation |
| CH04 | Avian | AY950258 | GenBank | A/chicken/Henan/01/2004(H5N1) | 1 | 8 | No extrapolation |
|  |  |  |  |  |  |  |  |
| CH1 | Human | CY010915 | GenBank | A/Memphis/7/1980(H1N1) | 1 | 1 | No extrapolation |
| CH1 | Human | CY010914 | GenBank | A/Memphis/7/1980(H1N1) | 1 | 2 | No extrapolation |
| CH1 | Human | CY010913 | GenBank | A/Memphis/7/1980(H1N1) | 1 | 3 | No extrapolation |
| CH1 | Human | CY010908 | GenBank | A/Memphis/7/1980(H1N1) | 1 | 4 | No extrapolation |
| CH1 | Human | CY010911 | GenBank | A/Memphis/7/1980(H1N1) | 1 | 5 | No extrapolation |
| CH1 | Human | CY010910 | GenBank | A/Memphis/7/1980(H1N1) | 1 | 6 | No extrapolation |
| CH1 | Human | CY010909 | GenBank | A/Memphis/7/1980(H1N1) | 1 | 7 | No extrapolation |
| CH1 | Human | CY010912 | GenBank | A/Memphis/7/1980(H1N1) | 1 | 8 | No extrapolation |
|  |  |  |  |  |  |  |  |
| CH17 | Avian | MH209304 | GenBank | A/chicken/Hunan/S1220/2017(H7N9) | 1 | 1 | No extrapolation |
| CH17 | Avian | MH209305 | GenBank | A/chicken/Hunan/S1220/2017(H7N9) | 1 | 2 | No extrapolation |
| CH17 | Avian | MH209306 | GenBank | A/chicken/Hunan/S1220/2017(H7N9) | 1 | 3 | No extrapolation |
| CH17 | Avian | MH209307 | GenBank | A/chicken/Hunan/S1220/2017(H7N9) | 1 | 4 | No extrapolation |
| CH17 | Avian | MH209308 | GenBank | A/chicken/Hunan/S1220/2017(H7N9) | 1 | 5 | No extrapolation |
| CH17 | Avian | MH209309 | GenBank | A/chicken/Hunan/S1220/2017(H7N9) | 1 | 6 | No extrapolation |
| CH17 | Avian | MH209310 | GenBank | A/chicken/Hunan/S1220/2017(H7N9) | 1 | 7 | No extrapolation |
| CH17 | Avian | MH209311 | GenBank | A/chicken/Hunan/S1220/2017(H7N9) | 1 | 8 | No extrapolation |
|  |  |  |  |  |  |  |  |
| CHNL03 | Avian | AY342414 | GenBank | A/chicken/Netherlands/1/2003(H7N7) | 1 | 1 | No extrapolation |
| CHNL03 | Avian | AY340085 | GenBank | A/chicken/Netherlands/1/2003(H7N7) | 1 | 2 | No extrapolation |
| CHNL03 | Avian | AY342420 | GenBank | A/chicken/Netherlands/1/2003(H7N7) | 1 | 3 | No extrapolation |
| CHNL03 | Avian | AY338458 | GenBank | A/chicken/Netherlands/1/2003(H7N7) | 1 | 4 | No extrapolation |
| CHNL03 | Avian | AY342427 | GenBank | A/chicken/Netherlands/1/2003(H7N7) | 1 | 5 | No extrapolation |
| CHNL03 | Avian | AY340077 | GenBank | A/chicken/Netherlands/1/2003(H7N7) | 1 | 6 | No extrapolation |
| CHNL03 | Avian | AY340091 | GenBank | A/chicken/Netherlands/1/2003(H7N7) | 1 | 7 | No extrapolation |
| CHNL03 | Avian | AY342424 | GenBank | A/chicken/Netherlands/1/2003(H7N7) | 1 | 8 | No extrapolation |
|  |  |  |  |  |  |  |  |
| DKFJ01 | Avian | AY585504 | GenBank | A/duck/Fujian/01/2002(H5N1) | 1 | 1 | No extrapolation |
| DKFJ01 | Avian | AY585483 | GenBank | A/duck/Fujian/01/2002(H5N1) | 1 | 2 | No extrapolation |
| DKFJ01 | Avian | AY585462 | GenBank | A/duck/Fujian/01/2002(H5N1) | 1 | 3 | No extrapolation |
| DKFJ01 | Avian | AY585357 | GenBank | A/duck/Fujian/01/2002(H5N1) | 1 | 4 | No extrapolation |
| DKFJ01 | Avian | AY585420 | GenBank | A/duck/Fujian/01/2002(H5N1) | 1 | 5 | No extrapolation |
| DKFJ01 | Avian | AY585399 | GenBank | A/duck/Fujian/01/2002(H5N1) | 1 | 6 | No extrapolation |
| DKFJ01 | Avian | AY585378 | GenBank | A/duck/Fujian/01/2002(H5N1) | 1 | 7 | No extrapolation |
| DKFJ01 | Avian | AY585441 | GenBank | A/duck/Fujian/01/2002(H5N1) | 1 | 8 | Partial |
|  |  |  |  |  |  |  |  |
| DKGX12 | Avian | EU263342 | GenBank | A/duck/Guangxi/12/2003(H5N1) | 1 | 1 | No extrapolation |
| DKGX12 | Avian | EU263343 | GenBank | A/duck/Guangxi/12/2003(H5N1) | 1 | 2 | No extrapolation |
| DKGX12 | Avian | EU263344 | GenBank | A/duck/Guangxi/12/2003(H5N1) | 1 | 3 | No extrapolation |
| DKGX12 | Avian | EU263345 | GenBank | A/duck/Guangxi/12/2003(H5N1) | 1 | 4 | No extrapolation |
| DKGX12 | Avian | EU263346 | GenBank | A/duck/Guangxi/12/2003(H5N1) | 1 | 5 | No extrapolation |
| DKGX12 | Avian | EU263347 | GenBank | A/duck/Guangxi/12/2003(H5N1) | 1 | 6 | No extrapolation |
| DKGX12 | Avian | EU263348 | GenBank | A/duck/Guangxi/12/2003(H5N1) | 1 | 7 | No extrapolation |
| DKGX12 | Avian | EU263349 | GenBank | A/duck/Guangxi/12/2003(H5N1) | 1 | 8 | No extrapolation |
|  |  |  |  |  |  |  |  |
| DKGX27 | Avian | EU263350 | GenBank | A/duck/Guangxi/27/2003(H5N1) | 1 | 1 | No extrapolation |
| DKGX27 | Avian | EU263351 | GenBank | A/duck/Guangxi/27/2003(H5N1) | 1 | 2 | No extrapolation |
| DKGX27 | Avian | EU263352 | GenBank | A/duck/Guangxi/27/2003(H5N1) | 1 | 3 | No extrapolation |
| DKGX27 | Avian | EU263353 | GenBank | A/duck/Guangxi/27/2003(H5N1) | 1 | 4 | No extrapolation |
| DKGX27 | Avian | EU263354 | GenBank | A/duck/Guangxi/27/2003(H5N1) | 1 | 5 | No extrapolation |
| DKGX27 | Avian | EU263355 | GenBank | A/duck/Guangxi/27/2003(H5N1) | 1 | 6 | No extrapolation |
| DKGX27 | Avian | EU263356 | GenBank | A/duck/Guangxi/27/2003(H5N1) | 1 | 7 | No extrapolation |
| DKGX27 | Avian | EU263357 | GenBank | A/duck/Guangxi/27/2003(H5N1) | 1 | 8 | No extrapolation |
|  |  |  |  |  |  |  |  |
| DKGX35 | Avian | AY585515 | GenBank | A/duck/Guangxi/35/2001(H5N1) | 1 | 1 | No extrapolation |
| DKGX35 | Avian | AY585494 | GenBank | A/duck/Guangxi/35/2001(H5N1) | 1 | 2 | No extrapolation |
| DKGX35 | Avian | AY585473 | GenBank | A/duck/Guangxi/35/2001(H5N1) | 1 | 3 | No extrapolation |
| DKGX35 | Avian | AY585365 | GenBank | A/duck/Guangxi/35/2001(H5N1) | 1 | 4 | No extrapolation |
| DKGX35 | Avian | AY585431 | GenBank | A/duck/Guangxi/35/2001(H5N1) | 1 | 5 | No extrapolation |
| DKGX35 | Avian | AY585410 | GenBank | A/duck/Guangxi/35/2001(H5N1) | 1 | 6 | No extrapolation |
| DKGX35 | Avian | AY585389 | GenBank | A/duck/Guangxi/35/2001(H5N1) | 1 | 7 | No extrapolation |
| DKGX35 | Avian | AY585452 | GenBank | A/duck/Guangxi/35/2001(H5N1) | 1 | 8 | No extrapolation |
|  |  |  |  |  |  |  |  |
| DKGX53 | Avian | AY585517 | GenBank | A/duck/Guangxi/53/2002(H5N1) | 1 | 1 | No extrapolation |
| DKGX53 | Avian | AY585496 | GenBank | A/duck/Guangxi/53/2002(H5N1) | 1 | 2 | No extrapolation |
| DKGX53 | Avian | AY585475 | GenBank | A/duck/Guangxi/53/2002(H5N1) | 1 | 3 | No extrapolation |
| DKGX53 | Avian | AY585366 | GenBank | A/duck/Guangxi/53/2002(H5N1) | 1 | 4 | No extrapolation |
| DKGX53 | Avian | AY585433 | GenBank | A/duck/Guangxi/53/2002(H5N1) | 1 | 5 | No extrapolation |
| DKGX53 | Avian | AY585412 | GenBank | A/duck/Guangxi/53/2002(H5N1) | 1 | 6 | No extrapolation |
| DKGX53 | Avian | AY585390 | GenBank | A/duck/Guangxi/53/2002(H5N1) | 1 | 7 | No extrapolation |
| DKGX53 | Avian | AY585454 | GenBank | A/duck/Guangxi/53/2002(H5N1) | 1 | 8 | Partial |
|  |  |  |  |  |  |  |  |
| ENG63v1 | Avian | CY015072 | GenBank | A/turkey/England/1963(H7N3) | 1 | 1 | No extrapolation |
| ENG63v1 | Avian | CY015071 | GenBank | A/turkey/England/1963(H7N3) | 1 | 2 | No extrapolation |
| ENG63v1 | Avian | CY015070 | GenBank | A/turkey/England/1963(H7N3) | 1 | 3 | No extrapolation |
| ENG63v1 | Avian | CY015065 | GenBank | A/turkey/England/1963(H7N3) | 1 | 4 | No extrapolation |
| ENG63v1 | Avian | CY015068 | GenBank | A/turkey/England/1963(H7N3) | 1 | 5 | No extrapolation |
| ENG63v1 | Avian | CY015067 | GenBank | A/turkey/England/1963(H7N3) | 1 | 6 | No extrapolation |
| ENG63v1 | Avian | CY015066 | GenBank | A/turkey/England/1963(H7N3) | 1 | 7 | No extrapolation |
| ENG63v1 | Avian | CY015069 | GenBank | A/turkey/England/1963(H7N3) | 1 | 8 | No extrapolation |
|  |  |  |  |  |  |  |  |
| ENG63v2 | Avian | CY130157 | GenBank | A/turkey/England/1963(H7N3) | 2 | 1 | No extrapolation |
| ENG63v2 | Avian | CY130156 | GenBank | A/turkey/England/1963(H7N3) | 2 | 2 | No extrapolation |
| ENG63v2 | Avian | CY130155 | GenBank | A/turkey/England/1963(H7N3) | 2 | 3 | No extrapolation |
| ENG63v2 | Avian | CY130150 | GenBank | A/turkey/England/1963(H7N3) | 2 | 4 | No extrapolation |
| ENG63v2 | Avian | CY130153 | GenBank | A/turkey/England/1963(H7N3) | 2 | 5 | No extrapolation |
| ENG63v2 | Avian | CY130152 | GenBank | A/turkey/England/1963(H7N3) | 2 | 6 | No extrapolation |
| ENG63v2 | Avian | CY130151 | GenBank | A/turkey/England/1963(H7N3) | 2 | 7 | No extrapolation |
| ENG63v2 | Avian | CY130154 | GenBank | A/turkey/England/1963(H7N3) | 2 | 8 | No extrapolation |
|  |  |  |  |  |  |  |  |
| FM47v1 | Human | CY009619 | GenBank | A/FortMonmouth/1/1947(H1N1) | 1 | 1 | No extrapolation |
| FM47v1 | Human | CY009618 | GenBank | A/FortMonmouth/1/1947(H1N1) | 1 | 2 | No extrapolation |
| FM47v1 | Human | CY009617 | GenBank | A/FortMonmouth/1/1947(H1N1) | 1 | 3 | No extrapolation |
| FM47v1 | Human | CY009612 | GenBank | A/FortMonmouth/1/1947(H1N1) | 1 | 4 | No extrapolation |
| FM47v1 | Human | CY009615 | GenBank | A/FortMonmouth/1/1947(H1N1) | 1 | 5 | No extrapolation |
| FM47v1 | Human | CY009614 | GenBank | A/FortMonmouth/1/1947(H1N1) | 1 | 6 | No extrapolation |
| FM47v1 | Human | CY009613 | GenBank | A/FortMonmouth/1/1947(H1N1) | 1 | 7 | No extrapolation |
| FM47v1 | Human | CY009616 | GenBank | A/FortMonmouth/1/1947(H1N1) | 1 | 8 | No extrapolation |
|  |  |  |  |  |  |  |  |
| FM47v2 | Human | CY045787 | GenBank | A/FortMonmouth/1/1947(H1N1) | 2 | 1 | No extrapolation |
| FM47v2 | Human | CY045786 | GenBank | A/FortMonmouth/1/1947(H1N1) | 2 | 2 | No extrapolation |
| FM47v2 | Human | CY045785 | GenBank | A/FortMonmouth/1/1947(H1N1) | 2 | 3 | No extrapolation |
| FM47v2 | Human | CY045780 | GenBank | A/FortMonmouth/1/1947(H1N1) | 2 | 4 | No extrapolation |
| FM47v2 | Human | CY045783 | GenBank | A/FortMonmouth/1/1947(H1N1) | 2 | 5 | No extrapolation |
| FM47v2 | Human | CY045782 | GenBank | A/FortMonmouth/1/1947(H1N1) | 2 | 6 | No extrapolation |
| FM47v2 | Human | CY045781 | GenBank | A/FortMonmouth/1/1947(H1N1) | 2 | 7 | No extrapolation |
| FM47v2 | Human | CY045784 | GenBank | A/FortMonmouth/1/1947(H1N1) | 2 | 8 | No extrapolation |
|  |  |  |  |  |  |  |  |
| GER65 | Swan | DQ464357 | GenBank | A/swan/Germany/R65/2006(H5N1) | 1 | 1 | No extrapolation |
| GER65 | Swan | DQ464361 | GenBank | A/swan/Germany/R65/2006(H5N1) | 1 | 2 | No extrapolation |
| GER65 | Swan | DQ464360 | GenBank | A/swan/Germany/R65/2006(H5N1) | 1 | 3 | No extrapolation |
| GER65 | Swan | DQ464354 | GenBank | A/swan/Germany/R65/2006(H5N1) | 1 | 4 | No extrapolation |
| GER65 | Swan | DQ464359 | GenBank | A/swan/Germany/R65/2006(H5N1) | 1 | 5 | No extrapolation |
| GER65 | Swan | DQ464355 | GenBank | A/swan/Germany/R65/2006(H5N1) | 1 | 6 | No extrapolation |
| GER65 | Swan | DQ464356 | GenBank | A/swan/Germany/R65/2006(H5N1) | 1 | 7 | No extrapolation |
| GER65 | Swan | DQ464358 | GenBank | A/swan/Germany/R65/2006(H5N1) | 1 | 8 | No extrapolation |
|  |  |  |  |  |  |  |  |
| GSH7 | Avian | EPI1215859 | GISAID | A/goose/Guangdong/SH7/2013(H5N1) | 1 | 1 | No extrapolation |
| GSH7 | Avian | EPI1215860 | GISAID | A/goose/Guangdong/SH7/2013(H5N1) | 1 | 2 | No extrapolation |
| GSH7 | Avian | EPI1215861 | GISAID | A/goose/Guangdong/SH7/2013(H5N1) | 1 | 3 | No extrapolation |
| GSH7 | Avian | EPI1215862 | GISAID | A/goose/Guangdong/SH7/2013(H5N1) | 1 | 4 | No extrapolation |
| GSH7 | Avian | EPI1215863 | GISAID | A/goose/Guangdong/SH7/2013(H5N1) | 1 | 5 | No extrapolation |
| GSH7 | Avian | EPI980838 | GISAID | A/goose/Guangdong/SH7/2013(H5N1) | 1 | 6 | No extrapolation |
| GSH7 | Avian | EPI1215865 | GISAID | A/goose/Guangdong/SH7/2013(H5N1) | 1 | 7 | Partial |
| GSH7 | Avian | EPI1215864 | GISAID | A/goose/Guangdong/SH7/2013(H5N1) | 1 | 8 | No extrapolation |
|  |  |  |  |  |  |  |  |
| GSHK437 | Goose | GU052026 | GenBank | A/goose/HongKong/437-6/1999(H5N1) | 1 | 1 | No extrapolation |
| GSHK437 | Goose | GU052025 | GenBank | A/goose/HongKong/437-6/1999(H5N1) | 1 | 2 | No extrapolation |
| GSHK437 | Goose | GU052024 | GenBank | A/goose/HongKong/437-6/1999(H5N1) | 1 | 3 | No extrapolation |
| GSHK437 | Goose | GU052019 | GenBank | A/goose/HongKong/437-6/1999(H5N1) | 1 | 4 | No extrapolation |
| GSHK437 | Goose | GU052022 | GenBank | A/goose/HongKong/437-6/1999(H5N1) | 1 | 5 | Partial |
| GSHK437 | Goose | GU052021 | GenBank | A/goose/HongKong/437-6/1999(H5N1) | 1 | 6 | No extrapolation |
| GSHK437 | Goose | GU052020 | GenBank | A/goose/HongKong/437-6/1999(H5N1) | 1 | 7 | No extrapolation |
| GSHK437 | Goose | GU052023 | GenBank | A/goose/HongKong/437-6/1999(H5N1) | 1 | 8 | No extrapolation |
|  |  |  |  |  |  |  |  |
| GSW154 | Avian | EPI580384 | GISAID | A/chicken/Guangdong/SW154/2015(H7N9) | 1 | 1 | No extrapolation |
| GSW154 | Avian | EPI580385 | GISAID | A/chicken/Guangdong/SW154/2015(H7N9) | 1 | 2 | No extrapolation |
| GSW154 | Avian | EPI580386 | GISAID | A/chicken/Guangdong/SW154/2015(H7N9) | 1 | 3 | No extrapolation |
| GSW154 | Avian | EPI580387 | GISAID | A/chicken/Guangdong/SW154/2015(H7N9) | 1 | 4 | No extrapolation |
| GSW154 | Avian | EPI580388 | GISAID | A/chicken/Guangdong/SW154/2015(H7N9) | 1 | 5 | No extrapolation |
| GSW154 | Avian | EPI580389 | GISAID | A/chicken/Guangdong/SW154/2015(H7N9) | 1 | 6 | No extrapolation |
| GSW154 | Avian | EPI580390 | GISAID | A/chicken/Guangdong/SW154/2015(H7N9) | 1 | 7 | No extrapolation |
| GSW154 | Avian | EPI580391 | GISAID | A/chicken/Guangdong/SW154/2015(H7N9) | 1 | 8 | No extrapolation |
|  |  |  |  |  |  |  |  |
| GTH005 | Human | EPI926822 | GISAID | A/Guangdong/Th005/2017(H7N9) | 1 | 1 | No extrapolation |
| GTH005 | Human | EPI926823 | GISAID | A/Guangdong/Th005/2017(H7N9) | 1 | 2 | No extrapolation |
| GTH005 | Human | EPI926821 | GISAID | A/Guangdong/Th005/2017(H7N9) | 1 | 3 | No extrapolation |
| GTH005 | Human | EPI926825 | GISAID | A/Guangdong/Th005/2017(H7N9) | 1 | 4 | No extrapolation |
| GTH005 | Human | EPI926818 | GISAID | A/Guangdong/Th005/2017(H7N9) | 1 | 5 | No extrapolation |
| GTH005 | Human | EPI926824 | GISAID | A/Guangdong/Th005/2017(H7N9) | 1 | 6 | No extrapolation |
| GTH005 | Human | EPI926820 | GISAID | A/Guangdong/Th005/2017(H7N9) | 1 | 7 | No extrapolation |
| GTH005 | Human | EPI926819 | GISAID | A/Guangdong/Th005/2017(H7N9) | 1 | 8 | No extrapolation |
|  |  |  |  |  |  |  |  |
| GTH008 | Human | EPI926814 | GISAID | A/Guangdong/Th008/2017(H7N9) | 1 | 1 | No extrapolation |
| GTH008 | Human | EPI926815 | GISAID | A/Guangdong/Th008/2017(H7N9) | 1 | 2 | No extrapolation |
| GTH008 | Human | EPI926813 | GISAID | A/Guangdong/Th008/2017(H7N9) | 1 | 3 | No extrapolation |
| GTH008 | Human | EPI926817 | GISAID | A/Guangdong/Th008/2017(H7N9) | 1 | 4 | No extrapolation |
| GTH008 | Human | EPI926810 | GISAID | A/Guangdong/Th008/2017(H7N9) | 1 | 5 | No extrapolation |
| GTH008 | Human | EPI926816 | GISAID | A/Guangdong/Th008/2017(H7N9) | 1 | 6 | No extrapolation |
| GTH008 | Human | EPI926812 | GISAID | A/Guangdong/Th008/2017(H7N9) | 1 | 7 | No extrapolation |
| GTH008 | Human | EPI926811 | GISAID | A/Guangdong/Th008/2017(H7N9) | 1 | 8 | No extrapolation |
|  |  |  |  |  |  |  |  |
| GZ14 | Human | KP765785 | GenBank | A/Guangzhou/39715/2014(H5N6) | 1 | 1 | No extrapolation |
| GZ14 | Human | KP765786 | GenBank | A/Guangzhou/39715/2014(H5N6) | 1 | 2 | No extrapolation |
| GZ14 | Human | KP765787 | GenBank | A/Guangzhou/39715/2014(H5N6) | 1 | 3 | No extrapolation |
| GZ14 | Human | KP765788 | GenBank | A/Guangzhou/39715/2014(H5N6) | 1 | 4 | No extrapolation |
| GZ14 | Human | KP765789 | GenBank | A/Guangzhou/39715/2014(H5N6) | 1 | 5 | No extrapolation |
| GZ14 | Human | KP765790 | GenBank | A/Guangzhou/39715/2014(H5N6) | 1 | 6 | No extrapolation |
| GZ14 | Human | KP765791 | GenBank | A/Guangzhou/39715/2014(H5N6) | 1 | 7 | No extrapolation |
| GZ14 | Human | KP765792 | GenBank | A/Guangzhou/39715/2014(H5N6) | 1 | 8 | No extrapolation |
|  |  |  |  |  |  |  |  |
| H5TK13 | Human | EF620011 | GenBank | A/Turkey/13/2006(H5N1) | 1 | 1 | Full segment; Partial |
| H5TK13 | Human | EF620010 | GenBank | A/Turkey/13/2006(H5N1) | 1 | 2 | Full segment |
| H5TK13 | Human | EF620009 | GenBank | A/Turkey/13/2006(H5N1) | 1 | 3 | Full segment; Partial |
| H5TK13 | Human | EF619989 | GenBank | A/Turkey/13/2006(H5N1) | 1 | 4 | Full segment; Partial |
| H5TK13 | Human | EF620007 | GenBank | A/Turkey/13/2006(H5N1) | 1 | 5 | Full segment; Partial |
| H5TK13 | Human | EF619988 | GenBank | A/Turkey/13/2006(H5N1) | 1 | 6 | Full segment; Partial |
| H5TK13 | Human | EF620006 | GenBank | A/Turkey/13/2006(H5N1) | 1 | 7 | Full segment; Partial |
| H5TK13 | Human | EF620008 | GenBank | A/Turkey/13/2006(H5N1) | 1 | 8 | Full segment; Partial |
|  |  |  |  |  |  |  |  |
| HA4 | Human | GQ166207 | GenBank | A/Hamburg/4/2009(H1N1) | 1 | 1 | No extrapolation |
| HA4 | Human | GQ166209 | GenBank | A/Hamburg/4/2009(H1N1) | 1 | 2 | No extrapolation |
| HA4 | Human | GQ166211 | GenBank | A/Hamburg/4/2009(H1N1) | 1 | 3 | No extrapolation |
| HA4 | Human | GQ166213 | GenBank | A/Hamburg/4/2009(H1N1) | 1 | 4 | No extrapolation |
| HA4 | Human | GQ166215 | GenBank | A/Hamburg/4/2009(H1N1) | 1 | 5 | No extrapolation |
| HA4 | Human | GQ166217 | GenBank | A/Hamburg/4/2009(H1N1) | 1 | 6 | No extrapolation |
| HA4 | Human | GQ166219 | GenBank | A/Hamburg/4/2009(H1N1) | 1 | 7 | Partial |
| HA4 | Human | GQ166221 | GenBank | A/Hamburg/4/2009(H1N1) | 1 | 8 | No extrapolation |
|  |  |  |  |  |  |  |  |
| HEY16 | Avian | EPI919530 | GISAID | A/chicken/Heyuan/16876/2016(H7N9) | 1 | 1 | No extrapolation |
| HEY16 | Avian | EPI919531 | GISAID | A/chicken/Heyuan/16876/2016(H7N9) | 1 | 2 | No extrapolation |
| HEY16 | Avian | EPI919532 | GISAID | A/chicken/Heyuan/16876/2016(H7N9) | 1 | 3 | No extrapolation |
| HEY16 | Avian | EPI919533 | GISAID | A/chicken/Heyuan/16876/2016(H7N9) | 1 | 4 | Partial |
| HEY16 | Avian | EPI919534 | GISAID | A/chicken/Heyuan/16876/2016(H7N9) | 1 | 5 | No extrapolation |
| HEY16 | Avian | EPI919535 | GISAID | A/chicken/Heyuan/16876/2016(H7N9) | 1 | 6 | No extrapolation |
| HEY16 | Avian | EPI919536 | GISAID | A/chicken/Heyuan/16876/2016(H7N9) | 1 | 7 | No extrapolation |
| HEY16 | Avian | EPI919537 | GISAID | A/chicken/Heyuan/16876/2016(H7N9) | 1 | 8 | Partial |
|  |  |  |  |  |  |  |  |
| HH05 | Human | HQ111361 | GenBank | A/Hamburg/05/2009(H1N1) | 1 | 1 | No extrapolation |
| HH05 | Human | HQ111362 | GenBank | A/Hamburg/05/2009(H1N1) | 1 | 2 | No extrapolation |
| HH05 | Human | HQ111363 | GenBank | A/Hamburg/05/2009(H1N1) | 1 | 3 | No extrapolation |
| HH05 | Human | HQ111364 | GenBank | A/Hamburg/05/2009(H1N1) | 1 | 4 | No extrapolation |
| HH05 | Human | HQ111365 | GenBank | A/Hamburg/05/2009(H1N1) | 1 | 5 | No extrapolation |
| HH05 | Human | HQ111366 | GenBank | A/Hamburg/05/2009(H1N1) | 1 | 6 | No extrapolation |
| HH05 | Human | HQ111367 | GenBank | A/Hamburg/05/2009(H1N1) | 1 | 7 | Partial |
| HH05 | Human | HQ111368 | GenBank | A/Hamburg/05/2009(H1N1) | 1 | 8 | Partial |
|  |  |  |  |  |  |  |  |
| HH15 | Human | GU480807 | GenBank | A/Hamburg/NY1580/2009(H1N1) | 1 | 1 | No extrapolation |
| HH15 | Human | HQ104924 | GenBank | A/Hamburg/NY1580/2009(H1N1) | 1 | 2 | No extrapolation |
| HH15 | Human | HQ104925 | GenBank | A/Hamburg/NY1580/2009(H1N1) | 1 | 3 | No extrapolation |
| HH15 | Human | HQ104926 | GenBank | A/Hamburg/NY1580/2009(H1N1) | 1 | 4 | No extrapolation |
| HH15 | Human | HM598305 | GenBank | A/Hamburg/NY1580/2009(H1N1) | 1 | 5 | No extrapolation |
| HH15 | Human | HQ104927 | GenBank | A/Hamburg/NY1580/2009(H1N1) | 1 | 6 | No extrapolation |
| HH15 | Human | HQ104928 | GenBank | A/Hamburg/NY1580/2009(H1N1) | 1 | 7 | Partial |
| HH15 | Human | HQ104929 | GenBank | A/Hamburg/NY1580/2009(H1N1) | 1 | 8 | Partial |
|  |  |  |  |  |  |  |  |
| HK-1-1 | Human | CY033008 | GenBank | A/HongKong/1-1/1968(H3N2) | 1 | 1 | No extrapolation |
| HK-1-1 | Human | CY033007 | GenBank | A/HongKong/1-1/1968(H3N2) | 1 | 2 | No extrapolation |
| HK-1-1 | Human | CY033006 | GenBank | A/HongKong/1-1/1968(H3N2) | 1 | 3 | No extrapolation |
| HK-1-1 | Human | CY033001 | GenBank | A/HongKong/1-1/1968(H3N2) | 1 | 4 | No extrapolation |
| HK-1-1 | Human | CY033004 | GenBank | A/HongKong/1-1/1968(H3N2) | 1 | 5 | No extrapolation |
| HK-1-1 | Human | CY033003 | GenBank | A/HongKong/1-1/1968(H3N2) | 1 | 6 | No extrapolation |
| HK-1-1 | Human | CY033002 | GenBank | A/HongKong/1-1/1968(H3N2) | 1 | 7 | No extrapolation |
| HK-1-1 | Human | CY033005 | GenBank | A/HongKong/1-1/1968(H3N2) | 1 | 8 | No extrapolation |
|  |  |  |  |  |  |  |  |
| HK-1-1-MA-12 | Mouse | CY033512 | GenBank | A/HongKong/1-1-MA-12/1968(H3N2) | 1 | 1 | No extrapolation |
| HK-1-1-MA-12 | Mouse | CY033511 | GenBank | A/HongKong/1-1-MA-12/1968(H3N2) | 1 | 2 | No extrapolation |
| HK-1-1-MA-12 | Mouse | CY033510 | GenBank | A/HongKong/1-1-MA-12/1968(H3N2) | 1 | 3 | No extrapolation |
| HK-1-1-MA-12 | Mouse | CY033505 | GenBank | A/HongKong/1-1-MA-12/1968(H3N2) | 1 | 4 | No extrapolation |
| HK-1-1-MA-12 | Mouse | CY033508 | GenBank | A/HongKong/1-1-MA-12/1968(H3N2) | 1 | 5 | No extrapolation |
| HK-1-1-MA-12 | Mouse | CY033507 | GenBank | A/HongKong/1-1-MA-12/1968(H3N2) | 1 | 6 | No extrapolation |
| HK-1-1-MA-12 | Mouse | CY033506 | GenBank | A/HongKong/1-1-MA-12/1968(H3N2) | 1 | 7 | No extrapolation |
| HK-1-1-MA-12 | Mouse | CY033509 | GenBank | A/HongKong/1-1-MA-12/1968(H3N2) | 1 | 8 | No extrapolation |
|  |  |  |  |  |  |  |  |
| HK-1-1-MA-12A | Mouse | CY034949 | GenBank | A/HongKong/1-1-MA-12A/1968(H3N2) | 1 | 1 | No extrapolation |
| HK-1-1-MA-12A | Mouse | CY034948 | GenBank | A/HongKong/1-1-MA-12A/1968(H3N2) | 1 | 2 | No extrapolation |
| HK-1-1-MA-12A | Mouse | CY034947 | GenBank | A/HongKong/1-1-MA-12A/1968(H3N2) | 1 | 3 | No extrapolation |
| HK-1-1-MA-12A | Mouse | CY034942 | GenBank | A/HongKong/1-1-MA-12A/1968(H3N2) | 1 | 4 | No extrapolation |
| HK-1-1-MA-12A | Mouse | CY034945 | GenBank | A/HongKong/1-1-MA-12A/1968(H3N2) | 1 | 5 | No extrapolation |
| HK-1-1-MA-12A | Mouse | CY034944 | GenBank | A/HongKong/1-1-MA-12A/1968(H3N2) | 1 | 6 | No extrapolation |
| HK-1-1-MA-12A | Mouse | CY034943 | GenBank | A/HongKong/1-1-MA-12A/1968(H3N2) | 1 | 7 | No extrapolation |
| HK-1-1-MA-12A | Mouse | CY034946 | GenBank | A/HongKong/1-1-MA-12A/1968(H3N2) | 1 | 8 | No extrapolation |
|  |  |  |  |  |  |  |  |
| HK-1-1-MA-12B | Mouse | CY034957 | GenBank | A/HongKong/1-1-MA-12B/1968(H3N2) | 1 | 1 | No extrapolation |
| HK-1-1-MA-12B | Mouse | CY034956 | GenBank | A/HongKong/1-1-MA-12B/1968(H3N2) | 1 | 2 | No extrapolation |
| HK-1-1-MA-12B | Mouse | CY034955 | GenBank | A/HongKong/1-1-MA-12B/1968(H3N2) | 1 | 3 | No extrapolation |
| HK-1-1-MA-12B | Mouse | CY034950 | GenBank | A/HongKong/1-1-MA-12B/1968(H3N2) | 1 | 4 | No extrapolation |
| HK-1-1-MA-12B | Mouse | CY034953 | GenBank | A/HongKong/1-1-MA-12B/1968(H3N2) | 1 | 5 | No extrapolation |
| HK-1-1-MA-12B | Mouse | CY034952 | GenBank | A/HongKong/1-1-MA-12B/1968(H3N2) | 1 | 6 | No extrapolation |
| HK-1-1-MA-12B | Mouse | CY034951 | GenBank | A/HongKong/1-1-MA-12B/1968(H3N2) | 1 | 7 | No extrapolation |
| HK-1-1-MA-12B | Mouse | CY034954 | GenBank | A/HongKong/1-1-MA-12B/1968(H3N2) | 1 | 8 | No extrapolation |
|  |  |  |  |  |  |  |  |
| HK-1-1-MA-12C | Mouse | CY034965 | GenBank | A/HongKong/1-1-MA-12C/1968(H3N2) | 1 | 1 | No extrapolation |
| HK-1-1-MA-12C | Mouse | CY034964 | GenBank | A/HongKong/1-1-MA-12C/1968(H3N2) | 1 | 2 | No extrapolation |
| HK-1-1-MA-12C | Mouse | CY034963 | GenBank | A/HongKong/1-1-MA-12C/1968(H3N2) | 1 | 3 | No extrapolation |
| HK-1-1-MA-12C | Mouse | CY034958 | GenBank | A/HongKong/1-1-MA-12C/1968(H3N2) | 1 | 4 | No extrapolation |
| HK-1-1-MA-12C | Mouse | CY034961 | GenBank | A/HongKong/1-1-MA-12C/1968(H3N2) | 1 | 5 | No extrapolation |
| HK-1-1-MA-12C | Mouse | CY034960 | GenBank | A/HongKong/1-1-MA-12C/1968(H3N2) | 1 | 6 | No extrapolation |
| HK-1-1-MA-12C | Mouse | CY034959 | GenBank | A/HongKong/1-1-MA-12C/1968(H3N2) | 1 | 7 | No extrapolation |
| HK-1-1-MA-12C | Mouse | CY034962 | GenBank | A/HongKong/1-1-MA-12C/1968(H3N2) | 1 | 8 | No extrapolation |
|  |  |  |  |  |  |  |  |
| HK-1-1-MA-12D | Mouse | CY033995 | GenBank | A/HongKong/1-1-MA-12D/1968(H3N2) | 1 | 1 | No extrapolation |
| HK-1-1-MA-12D | Mouse | CY033994 | GenBank | A/HongKong/1-1-MA-12D/1968(H3N2) | 1 | 2 | No extrapolation |
| HK-1-1-MA-12D | Mouse | CY033993 | GenBank | A/HongKong/1-1-MA-12D/1968(H3N2) | 1 | 3 | No extrapolation |
| HK-1-1-MA-12D | Mouse | CY033988 | GenBank | A/HongKong/1-1-MA-12D/1968(H3N2) | 1 | 4 | No extrapolation |
| HK-1-1-MA-12D | Mouse | CY033991 | GenBank | A/HongKong/1-1-MA-12D/1968(H3N2) | 1 | 5 | No extrapolation |
| HK-1-1-MA-12D | Mouse | CY033990 | GenBank | A/HongKong/1-1-MA-12D/1968(H3N2) | 1 | 6 | No extrapolation |
| HK-1-1-MA-12D | Mouse | CY033989 | GenBank | A/HongKong/1-1-MA-12D/1968(H3N2) | 1 | 7 | No extrapolation |
| HK-1-1-MA-12D | Mouse | CY033992 | GenBank | A/HongKong/1-1-MA-12D/1968(H3N2) | 1 | 8 | No extrapolation |
|  |  |  |  |  |  |  |  |
| HK-1-1-MA-12E | Mouse | CY034973 | GenBank | A/HongKong/1-1-MA-12E/1968(H3N2) | 1 | 1 | No extrapolation |
| HK-1-1-MA-12E | Mouse | CY034972 | GenBank | A/HongKong/1-1-MA-12E/1968(H3N2) | 1 | 2 | No extrapolation |
| HK-1-1-MA-12E | Mouse | CY034971 | GenBank | A/HongKong/1-1-MA-12E/1968(H3N2) | 1 | 3 | No extrapolation |
| HK-1-1-MA-12E | Mouse | CY034966 | GenBank | A/HongKong/1-1-MA-12E/1968(H3N2) | 1 | 4 | No extrapolation |
| HK-1-1-MA-12E | Mouse | CY034969 | GenBank | A/HongKong/1-1-MA-12E/1968(H3N2) | 1 | 5 | No extrapolation |
| HK-1-1-MA-12E | Mouse | CY034968 | GenBank | A/HongKong/1-1-MA-12E/1968(H3N2) | 1 | 6 | No extrapolation |
| HK-1-1-MA-12E | Mouse | CY034967 | GenBank | A/HongKong/1-1-MA-12E/1968(H3N2) | 1 | 7 | No extrapolation |
| HK-1-1-MA-12E | Mouse | CY034970 | GenBank | A/HongKong/1-1-MA-12E/1968(H3N2) | 1 | 8 | No extrapolation |
|  |  |  |  |  |  |  |  |
| HK-1-1-MA-20 | Mouse | CY044276 | GenBank | A/HongKong/1-1-MA-20/1968(H3N2) | 1 | 1 | No extrapolation |
| HK-1-1-MA-20 | Mouse | CY044275 | GenBank | A/HongKong/1-1-MA-20/1968(H3N2) | 1 | 2 | No extrapolation |
| HK-1-1-MA-20 | Mouse | CY044274 | GenBank | A/HongKong/1-1-MA-20/1968(H3N2) | 1 | 3 | No extrapolation |
| HK-1-1-MA-20 | Mouse | CY044269 | GenBank | A/HongKong/1-1-MA-20/1968(H3N2) | 1 | 4 | No extrapolation |
| HK-1-1-MA-20 | Mouse | CY044272 | GenBank | A/HongKong/1-1-MA-20/1968(H3N2) | 1 | 5 | No extrapolation |
| HK-1-1-MA-20 | Mouse | CY044271 | GenBank | A/HongKong/1-1-MA-20/1968(H3N2) | 1 | 6 | No extrapolation |
| HK-1-1-MA-20 | Mouse | CY044270 | GenBank | A/HongKong/1-1-MA-20/1968(H3N2) | 1 | 7 | No extrapolation |
| HK-1-1-MA-20 | Mouse | CY044273 | GenBank | A/HongKong/1-1-MA-20/1968(H3N2) | 1 | 8 | No extrapolation |
|  |  |  |  |  |  |  |  |
| HK-1-1-MA-20A | Mouse | CY032984 | GenBank | A/HongKong/1-1-MA-20A/1968(H3N2) | 1 | 1 | No extrapolation |
| HK-1-1-MA-20A | Mouse | CY032983 | GenBank | A/HongKong/1-1-MA-20A/1968(H3N2) | 1 | 2 | No extrapolation |
| HK-1-1-MA-20A | Mouse | CY032982 | GenBank | A/HongKong/1-1-MA-20A/1968(H3N2) | 1 | 3 | No extrapolation |
| HK-1-1-MA-20A | Mouse | CY032977 | GenBank | A/HongKong/1-1-MA-20A/1968(H3N2) | 1 | 4 | No extrapolation |
| HK-1-1-MA-20A | Mouse | CY032980 | GenBank | A/HongKong/1-1-MA-20A/1968(H3N2) | 1 | 5 | No extrapolation |
| HK-1-1-MA-20A | Mouse | CY032979 | GenBank | A/HongKong/1-1-MA-20A/1968(H3N2) | 1 | 6 | No extrapolation |
| HK-1-1-MA-20A | Mouse | CY032978 | GenBank | A/HongKong/1-1-MA-20A/1968(H3N2) | 1 | 7 | No extrapolation |
| HK-1-1-MA-20A | Mouse | CY032981 | GenBank | A/HongKong/1-1-MA-20A/1968(H3N2) | 1 | 8 | No extrapolation |
|  |  |  |  |  |  |  |  |
| HK-1-1-MA-20B | Mouse | CY032992 | GenBank | A/HongKong/1-1-MA-20B/1968(H3N2) | 1 | 1 | No extrapolation |
| HK-1-1-MA-20B | Mouse | CY032991 | GenBank | A/HongKong/1-1-MA-20B/1968(H3N2) | 1 | 2 | No extrapolation |
| HK-1-1-MA-20B | Mouse | CY032990 | GenBank | A/HongKong/1-1-MA-20B/1968(H3N2) | 1 | 3 | No extrapolation |
| HK-1-1-MA-20B | Mouse | CY032985 | GenBank | A/HongKong/1-1-MA-20B/1968(H3N2) | 1 | 4 | No extrapolation |
| HK-1-1-MA-20B | Mouse | CY032988 | GenBank | A/HongKong/1-1-MA-20B/1968(H3N2) | 1 | 5 | No extrapolation |
| HK-1-1-MA-20B | Mouse | CY032987 | GenBank | A/HongKong/1-1-MA-20B/1968(H3N2) | 1 | 6 | No extrapolation |
| HK-1-1-MA-20B | Mouse | CY032986 | GenBank | A/HongKong/1-1-MA-20B/1968(H3N2) | 1 | 7 | No extrapolation |
| HK-1-1-MA-20B | Mouse | CY032989 | GenBank | A/HongKong/1-1-MA-20B/1968(H3N2) | 1 | 8 | No extrapolation |
|  |  |  |  |  |  |  |  |
| HK-1-1-MA-20C | Mouse | CY045723 | GenBank | A/HongKong/1-1-MA-20C/1968(H3N2) | 1 | 1 | No extrapolation |
| HK-1-1-MA-20C | Mouse | CY045722 | GenBank | A/HongKong/1-1-MA-20C/1968(H3N2) | 1 | 2 | No extrapolation |
| HK-1-1-MA-20C | Mouse | CY045721 | GenBank | A/HongKong/1-1-MA-20C/1968(H3N2) | 1 | 3 | No extrapolation |
| HK-1-1-MA-20C | Mouse | CY045716 | GenBank | A/HongKong/1-1-MA-20C/1968(H3N2) | 1 | 4 | No extrapolation |
| HK-1-1-MA-20C | Mouse | CY045719 | GenBank | A/HongKong/1-1-MA-20C/1968(H3N2) | 1 | 5 | No extrapolation |
| HK-1-1-MA-20C | Mouse | CY045718 | GenBank | A/HongKong/1-1-MA-20C/1968(H3N2) | 1 | 6 | No extrapolation |
| HK-1-1-MA-20C | Mouse | CY045717 | GenBank | A/HongKong/1-1-MA-20C/1968(H3N2) | 1 | 7 | No extrapolation |
| HK-1-1-MA-20C | Mouse | CY045720 | GenBank | A/HongKong/1-1-MA-20C/1968(H3N2) | 1 | 8 | No extrapolation |
|  |  |  |  |  |  |  |  |
| HK-1-1-MA-20D | Mouse | CY034981 | GenBank | A/HongKong/1-1-MA-20D/1968(H3N2) | 1 | 1 | No extrapolation |
| HK-1-1-MA-20D | Mouse | CY034980 | GenBank | A/HongKong/1-1-MA-20D/1968(H3N2) | 1 | 2 | No extrapolation |
| HK-1-1-MA-20D | Mouse | CY034979 | GenBank | A/HongKong/1-1-MA-20D/1968(H3N2) | 1 | 3 | No extrapolation |
| HK-1-1-MA-20D | Mouse | CY034974 | GenBank | A/HongKong/1-1-MA-20D/1968(H3N2) | 1 | 4 | No extrapolation |
| HK-1-1-MA-20D | Mouse | CY034977 | GenBank | A/HongKong/1-1-MA-20D/1968(H3N2) | 1 | 5 | No extrapolation |
| HK-1-1-MA-20D | Mouse | CY034976 | GenBank | A/HongKong/1-1-MA-20D/1968(H3N2) | 1 | 6 | No extrapolation |
| HK-1-1-MA-20D | Mouse | CY034975 | GenBank | A/HongKong/1-1-MA-20D/1968(H3N2) | 1 | 7 | No extrapolation |
| HK-1-1-MA-20D | Mouse | CY034978 | GenBank | A/HongKong/1-1-MA-20D/1968(H3N2) | 1 | 8 | No extrapolation |
|  |  |  |  |  |  |  |  |
| HK-1-1-MA-20E | Mouse | CY033000 | GenBank | A/HongKong/1-1-MA-20E/1968(H3N2) | 1 | 1 | No extrapolation |
| HK-1-1-MA-20E | Mouse | CY032999 | GenBank | A/HongKong/1-1-MA-20E/1968(H3N2) | 1 | 2 | No extrapolation |
| HK-1-1-MA-20E | Mouse | CY032998 | GenBank | A/HongKong/1-1-MA-20E/1968(H3N2) | 1 | 3 | No extrapolation |
| HK-1-1-MA-20E | Mouse | CY032993 | GenBank | A/HongKong/1-1-MA-20E/1968(H3N2) | 1 | 4 | No extrapolation |
| HK-1-1-MA-20E | Mouse | CY032996 | GenBank | A/HongKong/1-1-MA-20E/1968(H3N2) | 1 | 5 | No extrapolation |
| HK-1-1-MA-20E | Mouse | CY032995 | GenBank | A/HongKong/1-1-MA-20E/1968(H3N2) | 1 | 6 | No extrapolation |
| HK-1-1-MA-20E | Mouse | CY032994 | GenBank | A/HongKong/1-1-MA-20E/1968(H3N2) | 1 | 7 | No extrapolation |
| HK-1-1-MA-20E | Mouse | CY032997 | GenBank | A/HongKong/1-1-MA-20E/1968(H3N2) | 1 | 8 | No extrapolation |
|  |  |  |  |  |  |  |  |
| HK-1-11 | Human | CY034011 | GenBank | A/HongKong/1-11/1968(H3N2) | 1 | 1 | No extrapolation |
| HK-1-11 | Human | CY034010 | GenBank | A/HongKong/1-11/1968(H3N2) | 1 | 2 | No extrapolation |
| HK-1-11 | Human | CY034009 | GenBank | A/HongKong/1-11/1968(H3N2) | 1 | 3 | No extrapolation |
| HK-1-11 | Human | CY034004 | GenBank | A/HongKong/1-11/1968(H3N2) | 1 | 4 | No extrapolation |
| HK-1-11 | Human | CY034007 | GenBank | A/HongKong/1-11/1968(H3N2) | 1 | 5 | No extrapolation |
| HK-1-11 | Human | CY034006 | GenBank | A/HongKong/1-11/1968(H3N2) | 1 | 6 | No extrapolation |
| HK-1-11 | Human | CY034005 | GenBank | A/HongKong/1-11/1968(H3N2) | 1 | 7 | No extrapolation |
| HK-1-11 | Human | CY034008 | GenBank | A/HongKong/1-11/1968(H3N2) | 1 | 8 | No extrapolation |
|  |  |  |  |  |  |  |  |
| HK-1-11-MA21-2 | Mouse | CY033552 | GenBank | A/HongKong/1-11-MA21-2/1968(H3N2) | 1 | 1 | No extrapolation |
| HK-1-11-MA21-2 | Mouse | CY033551 | GenBank | A/HongKong/1-11-MA21-2/1968(H3N2) | 1 | 2 | No extrapolation |
| HK-1-11-MA21-2 | Mouse | CY033550 | GenBank | A/HongKong/1-11-MA21-2/1968(H3N2) | 1 | 3 | No extrapolation |
| HK-1-11-MA21-2 | Mouse | CY033545 | GenBank | A/HongKong/1-11-MA21-2/1968(H3N2) | 1 | 4 | No extrapolation |
| HK-1-11-MA21-2 | Mouse | CY033548 | GenBank | A/HongKong/1-11-MA21-2/1968(H3N2) | 1 | 5 | No extrapolation |
| HK-1-11-MA21-2 | Mouse | CY033547 | GenBank | A/HongKong/1-11-MA21-2/1968(H3N2) | 1 | 6 | No extrapolation |
| HK-1-11-MA21-2 | Mouse | CY033546 | GenBank | A/HongKong/1-11-MA21-2/1968(H3N2) | 1 | 7 | No extrapolation |
| HK-1-11-MA21-2 | Mouse | CY033549 | GenBank | A/HongKong/1-11-MA21-2/1968(H3N2) | 1 | 8 | No extrapolation |
|  |  |  |  |  |  |  |  |
| HK-1-12 | Human | CY033072 | GenBank | A/HongKong/1-12/1968(H3N2) | 1 | 1 | No extrapolation |
| HK-1-12 | Human | CY033071 | GenBank | A/HongKong/1-12/1968(H3N2) | 1 | 2 | No extrapolation |
| HK-1-12 | Human | CY033070 | GenBank | A/HongKong/1-12/1968(H3N2) | 1 | 3 | No extrapolation |
| HK-1-12 | Human | CY033065 | GenBank | A/HongKong/1-12/1968(H3N2) | 1 | 4 | No extrapolation |
| HK-1-12 | Human | CY033068 | GenBank | A/HongKong/1-12/1968(H3N2) | 1 | 5 | No extrapolation |
| HK-1-12 | Human | CY033067 | GenBank | A/HongKong/1-12/1968(H3N2) | 1 | 6 | No extrapolation |
| HK-1-12 | Human | CY033066 | GenBank | A/HongKong/1-12/1968(H3N2) | 1 | 7 | No extrapolation |
| HK-1-12 | Human | CY033069 | GenBank | A/HongKong/1-12/1968(H3N2) | 1 | 8 | No extrapolation |
|  |  |  |  |  |  |  |  |
| HK-1-2 | Human | CY033016 | GenBank | A/HongKong/1-2/1968(H3N2) | 1 | 1 | No extrapolation |
| HK-1-2 | Human | CY033015 | GenBank | A/HongKong/1-2/1968(H3N2) | 1 | 2 | No extrapolation |
| HK-1-2 | Human | CY033014 | GenBank | A/HongKong/1-2/1968(H3N2) | 1 | 3 | No extrapolation |
| HK-1-2 | Human | CY033009 | GenBank | A/HongKong/1-2/1968(H3N2) | 1 | 4 | No extrapolation |
| HK-1-2 | Human | CY033012 | GenBank | A/HongKong/1-2/1968(H3N2) | 1 | 5 | No extrapolation |
| HK-1-2 | Human | CY033011 | GenBank | A/HongKong/1-2/1968(H3N2) | 1 | 6 | No extrapolation |
| HK-1-2 | Human | CY033010 | GenBank | A/HongKong/1-2/1968(H3N2) | 1 | 7 | No extrapolation |
| HK-1-2 | Human | CY033013 | GenBank | A/HongKong/1-2/1968(H3N2) | 1 | 8 | No extrapolation |
|  |  |  |  |  |  |  |  |
| HK-1-4 | Human | CY033024 | GenBank | A/HongKong/1-4/1968(H3N2) | 1 | 1 | No extrapolation |
| HK-1-4 | Human | CY033023 | GenBank | A/HongKong/1-4/1968(H3N2) | 1 | 2 | No extrapolation |
| HK-1-4 | Human | CY033022 | GenBank | A/HongKong/1-4/1968(H3N2) | 1 | 3 | No extrapolation |
| HK-1-4 | Human | CY033017 | GenBank | A/HongKong/1-4/1968(H3N2) | 1 | 4 | No extrapolation |
| HK-1-4 | Human | CY033020 | GenBank | A/HongKong/1-4/1968(H3N2) | 1 | 5 | No extrapolation |
| HK-1-4 | Human | CY033019 | GenBank | A/HongKong/1-4/1968(H3N2) | 1 | 6 | No extrapolation |
| HK-1-4 | Human | CY033018 | GenBank | A/HongKong/1-4/1968(H3N2) | 1 | 7 | No extrapolation |
| HK-1-4 | Human | CY033021 | GenBank | A/HongKong/1-4/1968(H3N2) | 1 | 8 | No extrapolation |
|  |  |  |  |  |  |  |  |
| HK-1-4-MA21-1v1 | Mouse | CY080522 | GenBank | A/HongKong/1-4-MA21-1/1968(H3N2) | 1 | 1 | No extrapolation |
| HK-1-4-MA21-1v1 | Mouse | CY080521 | GenBank | A/HongKong/1-4-MA21-1/1968(H3N2) | 1 | 2 | No extrapolation |
| HK-1-4-MA21-1v1 | Mouse | CY080520 | GenBank | A/HongKong/1-4-MA21-1/1968(H3N2) | 1 | 3 | No extrapolation |
| HK-1-4-MA21-1v1 | Mouse | CY080515 | GenBank | A/HongKong/1-4-MA21-1/1968(H3N2) | 1 | 4 | No extrapolation |
| HK-1-4-MA21-1v1 | Mouse | CY080518 | GenBank | A/HongKong/1-4-MA21-1/1968(H3N2) | 1 | 5 | No extrapolation |
| HK-1-4-MA21-1v1 | Mouse | CY080517 | GenBank | A/HongKong/1-4-MA21-1/1968(H3N2) | 1 | 6 | No extrapolation |
| HK-1-4-MA21-1v1 | Mouse | CY080516 | GenBank | A/HongKong/1-4-MA21-1/1968(H3N2) | 1 | 7 | No extrapolation |
| HK-1-4-MA21-1v1 | Mouse | CY080519 | GenBank | A/HongKong/1-4-MA21-1/1968(H3N2) | 1 | 8 | No extrapolation |
|  |  |  |  |  |  |  |  |
| HK-1-4-MA21-1v2 | Mouse | HM641139 | GenBank | A/HongKong/1-4-MA21-1/1968(H3N2) | 2 | 1 | No extrapolation |
| HK-1-4-MA21-1v2 | Mouse | HM641150 | GenBank | A/HongKong/1-4-MA21-1/1968(H3N2) | 2 | 2 | No extrapolation |
| HK-1-4-MA21-1v2 | Mouse | HM641161 | GenBank | A/HongKong/1-4-MA21-1/1968(H3N2) | 2 | 3 | No extrapolation |
| HK-1-4-MA21-1v2 | Mouse | HM641172 | GenBank | A/HongKong/1-4-MA21-1/1968(H3N2) | 2 | 4 | No extrapolation |
| HK-1-4-MA21-1v2 | Mouse | HM641183 | GenBank | A/HongKong/1-4-MA21-1/1968(H3N2) | 2 | 5 | No extrapolation |
| HK-1-4-MA21-1v2 | Mouse | HM641194 | GenBank | A/HongKong/1-4-MA21-1/1968(H3N2) | 2 | 6 | No extrapolation |
| HK-1-4-MA21-1v2 | Mouse | HM641205 | GenBank | A/HongKong/1-4-MA21-1/1968(H3N2) | 2 | 7 | No extrapolation |
| HK-1-4-MA21-1v2 | Mouse | HM641216 | GenBank | A/HongKong/1-4-MA21-1/1968(H3N2) | 2 | 8 | No extrapolation |
|  |  |  |  |  |  |  |  |
| HK-1-4-MA21-3 | Mouse | CY033128 | GenBank | A/HongKong/1-4-MA21-3/1968(H3N2) | 1 | 1 | No extrapolation |
| HK-1-4-MA21-3 | Mouse | CY033127 | GenBank | A/HongKong/1-4-MA21-3/1968(H3N2) | 1 | 2 | No extrapolation |
| HK-1-4-MA21-3 | Mouse | CY033126 | GenBank | A/HongKong/1-4-MA21-3/1968(H3N2) | 1 | 3 | No extrapolation |
| HK-1-4-MA21-3 | Mouse | CY033121 | GenBank | A/HongKong/1-4-MA21-3/1968(H3N2) | 1 | 4 | No extrapolation |
| HK-1-4-MA21-3 | Mouse | CY033124 | GenBank | A/HongKong/1-4-MA21-3/1968(H3N2) | 1 | 5 | No extrapolation |
| HK-1-4-MA21-3 | Mouse | CY033123 | GenBank | A/HongKong/1-4-MA21-3/1968(H3N2) | 1 | 6 | No extrapolation |
| HK-1-4-MA21-3 | Mouse | CY033122 | GenBank | A/HongKong/1-4-MA21-3/1968(H3N2) | 1 | 7 | No extrapolation |
| HK-1-4-MA21-3 | Mouse | CY033125 | GenBank | A/HongKong/1-4-MA21-3/1968(H3N2) | 1 | 8 | No extrapolation |
|  |  |  |  |  |  |  |  |
| HK-1-5 | Human | CY033032 | GenBank | A/HongKong/1-5/1968(H3N2) | 1 | 1 | No extrapolation |
| HK-1-5 | Human | CY033031 | GenBank | A/HongKong/1-5/1968(H3N2) | 1 | 2 | No extrapolation |
| HK-1-5 | Human | CY033030 | GenBank | A/HongKong/1-5/1968(H3N2) | 1 | 3 | No extrapolation |
| HK-1-5 | Human | CY033025 | GenBank | A/HongKong/1-5/1968(H3N2) | 1 | 4 | No extrapolation |
| HK-1-5 | Human | CY033028 | GenBank | A/HongKong/1-5/1968(H3N2) | 1 | 5 | No extrapolation |
| HK-1-5 | Human | CY033027 | GenBank | A/HongKong/1-5/1968(H3N2) | 1 | 6 | No extrapolation |
| HK-1-5 | Human | CY033026 | GenBank | A/HongKong/1-5/1968(H3N2) | 1 | 7 | No extrapolation |
| HK-1-5 | Human | CY033029 | GenBank | A/HongKong/1-5/1968(H3N2) | 1 | 8 | No extrapolation |
|  |  |  |  |  |  |  |  |
| HK-1-5-MA21-1v1 | Mouse | CY045731 | GenBank | A/HongKong/1-5-MA21-1/1968(H3N2) | 1 | 1 | No extrapolation |
| HK-1-5-MA21-1v1 | Mouse | CY045730 | GenBank | A/HongKong/1-5-MA21-1/1968(H3N2) | 1 | 2 | No extrapolation |
| HK-1-5-MA21-1v1 | Mouse | CY045729 | GenBank | A/HongKong/1-5-MA21-1/1968(H3N2) | 1 | 3 | No extrapolation |
| HK-1-5-MA21-1v1 | Mouse | CY045724 | GenBank | A/HongKong/1-5-MA21-1/1968(H3N2) | 1 | 4 | No extrapolation |
| HK-1-5-MA21-1v1 | Mouse | CY045727 | GenBank | A/HongKong/1-5-MA21-1/1968(H3N2) | 1 | 5 | No extrapolation |
| HK-1-5-MA21-1v1 | Mouse | CY045726 | GenBank | A/HongKong/1-5-MA21-1/1968(H3N2) | 1 | 6 | No extrapolation |
| HK-1-5-MA21-1v1 | Mouse | CY045725 | GenBank | A/HongKong/1-5-MA21-1/1968(H3N2) | 1 | 7 | No extrapolation |
| HK-1-5-MA21-1v1 | Mouse | CY045728 | GenBank | A/HongKong/1-5-MA21-1/1968(H3N2) | 1 | 8 | No extrapolation |
|  |  |  |  |  |  |  |  |
| HK-1-5-MA21-1v2 | Mouse | HM641138 | GenBank | A/HongKong/1-5-MA21-1/1968(H3N2) | 2 | 1 | No extrapolation |
| HK-1-5-MA21-1v2 | Mouse | HM641149 | GenBank | A/HongKong/1-5-MA21-1/1968(H3N2) | 2 | 2 | No extrapolation |
| HK-1-5-MA21-1v2 | Mouse | HM641160 | GenBank | A/HongKong/1-5-MA21-1/1968(H3N2) | 2 | 3 | No extrapolation |
| HK-1-5-MA21-1v2 | Mouse | HM641171 | GenBank | A/HongKong/1-5-MA21-1/1968(H3N2) | 2 | 4 | No extrapolation |
| HK-1-5-MA21-1v2 | Mouse | HM641182 | GenBank | A/HongKong/1-5-MA21-1/1968(H3N2) | 2 | 5 | No extrapolation |
| HK-1-5-MA21-1v2 | Mouse | HM641193 | GenBank | A/HongKong/1-5-MA21-1/1968(H3N2) | 2 | 6 | No extrapolation |
| HK-1-5-MA21-1v2 | Mouse | HM641204 | GenBank | A/HongKong/1-5-MA21-1/1968(H3N2) | 2 | 7 | No extrapolation |
| HK-1-5-MA21-1v2 | Mouse | HM641215 | GenBank | A/HongKong/1-5-MA21-1/1968(H3N2) | 2 | 8 | No extrapolation |
|  |  |  |  |  |  |  |  |
| HK-1-5-MA21-3v1 | Mouse | CY044284 | GenBank | A/HongKong/1-5-MA21-3/1968(H3N2) | 1 | 1 | No extrapolation |
| HK-1-5-MA21-3v1 | Mouse | CY044283 | GenBank | A/HongKong/1-5-MA21-3/1968(H3N2) | 1 | 2 | No extrapolation |
| HK-1-5-MA21-3v1 | Mouse | CY044282 | GenBank | A/HongKong/1-5-MA21-3/1968(H3N2) | 1 | 3 | No extrapolation |
| HK-1-5-MA21-3v1 | Mouse | CY044277 | GenBank | A/HongKong/1-5-MA21-3/1968(H3N2) | 1 | 4 | No extrapolation |
| HK-1-5-MA21-3v1 | Mouse | CY044280 | GenBank | A/HongKong/1-5-MA21-3/1968(H3N2) | 1 | 5 | No extrapolation |
| HK-1-5-MA21-3v1 | Mouse | CY044279 | GenBank | A/HongKong/1-5-MA21-3/1968(H3N2) | 1 | 6 | No extrapolation |
| HK-1-5-MA21-3v1 | Mouse | CY044278 | GenBank | A/HongKong/1-5-MA21-3/1968(H3N2) | 1 | 7 | No extrapolation |
| HK-1-5-MA21-3v1 | Mouse | CY044281 | GenBank | A/HongKong/1-5-MA21-3/1968(H3N2) | 1 | 8 | No extrapolation |
|  |  |  |  |  |  |  |  |
| HK-1-5-MA21-3v2 | Mouse | HM641137 | GenBank | A/HongKong/1-5-MA21-3/1968(H3N2) | 2 | 1 | No extrapolation |
| HK-1-5-MA21-3v2 | Mouse | HM641148 | GenBank | A/HongKong/1-5-MA21-3/1968(H3N2) | 2 | 2 | No extrapolation |
| HK-1-5-MA21-3v2 | Mouse | HM641159 | GenBank | A/HongKong/1-5-MA21-3/1968(H3N2) | 2 | 3 | No extrapolation |
| HK-1-5-MA21-3v2 | Mouse | HM641170 | GenBank | A/HongKong/1-5-MA21-3/1968(H3N2) | 2 | 4 | No extrapolation |
| HK-1-5-MA21-3v2 | Mouse | HM641181 | GenBank | A/HongKong/1-5-MA21-3/1968(H3N2) | 2 | 5 | No extrapolation |
| HK-1-5-MA21-3v2 | Mouse | HM641192 | GenBank | A/HongKong/1-5-MA21-3/1968(H3N2) | 2 | 6 | No extrapolation |
| HK-1-5-MA21-3v2 | Mouse | HM641203 | GenBank | A/HongKong/1-5-MA21-3/1968(H3N2) | 2 | 7 | No extrapolation |
| HK-1-5-MA21-3v2 | Mouse | HM641214 | GenBank | A/HongKong/1-5-MA21-3/1968(H3N2) | 2 | 8 | No extrapolation |
|  |  |  |  |  |  |  |  |
| HK-1-6 | Human | CY033040 | GenBank | A/HongKong/1-6/1968(H3N2) | 1 | 1 | No extrapolation |
| HK-1-6 | Human | CY033039 | GenBank | A/HongKong/1-6/1968(H3N2) | 1 | 2 | No extrapolation |
| HK-1-6 | Human | CY033038 | GenBank | A/HongKong/1-6/1968(H3N2) | 1 | 3 | No extrapolation |
| HK-1-6 | Human | CY033033 | GenBank | A/HongKong/1-6/1968(H3N2) | 1 | 4 | No extrapolation |
| HK-1-6 | Human | CY033036 | GenBank | A/HongKong/1-6/1968(H3N2) | 1 | 5 | No extrapolation |
| HK-1-6 | Human | CY033035 | GenBank | A/HongKong/1-6/1968(H3N2) | 1 | 6 | No extrapolation |
| HK-1-6 | Human | CY033034 | GenBank | A/HongKong/1-6/1968(H3N2) | 1 | 7 | No extrapolation |
| HK-1-6 | Human | CY033037 | GenBank | A/HongKong/1-6/1968(H3N2) | 1 | 8 | No extrapolation |
|  |  |  |  |  |  |  |  |
| HK-1-6-MA21-3 | Mouse | CY045739 | GenBank | A/HongKong/1-6-MA21-3/1968(H3N2) | 1 | 1 | No extrapolation |
| HK-1-6-MA21-3 | Mouse | CY045738 | GenBank | A/HongKong/1-6-MA21-3/1968(H3N2) | 1 | 2 | No extrapolation |
| HK-1-6-MA21-3 | Mouse | CY045737 | GenBank | A/HongKong/1-6-MA21-3/1968(H3N2) | 1 | 3 | No extrapolation |
| HK-1-6-MA21-3 | Mouse | CY045732 | GenBank | A/HongKong/1-6-MA21-3/1968(H3N2) | 1 | 4 | No extrapolation |
| HK-1-6-MA21-3 | Mouse | CY045735 | GenBank | A/HongKong/1-6-MA21-3/1968(H3N2) | 1 | 5 | No extrapolation |
| HK-1-6-MA21-3 | Mouse | CY045734 | GenBank | A/HongKong/1-6-MA21-3/1968(H3N2) | 1 | 6 | No extrapolation |
| HK-1-6-MA21-3 | Mouse | CY045733 | GenBank | A/HongKong/1-6-MA21-3/1968(H3N2) | 1 | 7 | No extrapolation |
| HK-1-6-MA21-3 | Mouse | CY045736 | GenBank | A/HongKong/1-6-MA21-3/1968(H3N2) | 1 | 8 | No extrapolation |
|  |  |  |  |  |  |  |  |
| HK-1-8 | Human | CY034003 | GenBank | A/HongKong/1-8/1968(H3N2) | 1 | 1 | No extrapolation |
| HK-1-8 | Human | CY034002 | GenBank | A/HongKong/1-8/1968(H3N2) | 1 | 2 | No extrapolation |
| HK-1-8 | Human | CY034001 | GenBank | A/HongKong/1-8/1968(H3N2) | 1 | 3 | No extrapolation |
| HK-1-8 | Human | CY033996 | GenBank | A/HongKong/1-8/1968(H3N2) | 1 | 4 | No extrapolation |
| HK-1-8 | Human | CY033999 | GenBank | A/HongKong/1-8/1968(H3N2) | 1 | 5 | No extrapolation |
| HK-1-8 | Human | CY033998 | GenBank | A/HongKong/1-8/1968(H3N2) | 1 | 6 | No extrapolation |
| HK-1-8 | Human | CY033997 | GenBank | A/HongKong/1-8/1968(H3N2) | 1 | 7 | No extrapolation |
| HK-1-8 | Human | CY034000 | GenBank | A/HongKong/1-8/1968(H3N2) | 1 | 8 | No extrapolation |
|  |  |  |  |  |  |  |  |
| HK-1-9 | Human | CY033056 | GenBank | A/HongKong/1-9/1968(H3N2) | 1 | 1 | No extrapolation |
| HK-1-9 | Human | CY033055 | GenBank | A/HongKong/1-9/1968(H3N2) | 1 | 2 | No extrapolation |
| HK-1-9 | Human | CY033054 | GenBank | A/HongKong/1-9/1968(H3N2) | 1 | 3 | No extrapolation |
| HK-1-9 | Human | CY033049 | GenBank | A/HongKong/1-9/1968(H3N2) | 1 | 4 | No extrapolation |
| HK-1-9 | Human | CY033052 | GenBank | A/HongKong/1-9/1968(H3N2) | 1 | 5 | No extrapolation |
| HK-1-9 | Human | CY033051 | GenBank | A/HongKong/1-9/1968(H3N2) | 1 | 6 | No extrapolation |
| HK-1-9 | Human | CY033050 | GenBank | A/HongKong/1-9/1968(H3N2) | 1 | 7 | No extrapolation |
| HK-1-9 | Human | CY033053 | GenBank | A/HongKong/1-9/1968(H3N2) | 1 | 8 | No extrapolation |
|  |  |  |  |  |  |  |  |
| HK-1-9-MA21-3 | Mouse | CY033536 | GenBank | A/HongKong/1-9-MA21-3/1968(H3N2) | 1 | 1 | No extrapolation |
| HK-1-9-MA21-3 | Mouse | CY033535 | GenBank | A/HongKong/1-9-MA21-3/1968(H3N2) | 1 | 2 | No extrapolation |
| HK-1-9-MA21-3 | Mouse | CY033534 | GenBank | A/HongKong/1-9-MA21-3/1968(H3N2) | 1 | 3 | No extrapolation |
| HK-1-9-MA21-3 | Mouse | CY033529 | GenBank | A/HongKong/1-9-MA21-3/1968(H3N2) | 1 | 4 | No extrapolation |
| HK-1-9-MA21-3 | Mouse | CY033532 | GenBank | A/HongKong/1-9-MA21-3/1968(H3N2) | 1 | 5 | No extrapolation |
| HK-1-9-MA21-3 | Mouse | CY033531 | GenBank | A/HongKong/1-9-MA21-3/1968(H3N2) | 1 | 6 | No extrapolation |
| HK-1-9-MA21-3 | Mouse | CY033530 | GenBank | A/HongKong/1-9-MA21-3/1968(H3N2) | 1 | 7 | No extrapolation |
| HK-1-9-MA21-3 | Mouse | CY033533 | GenBank | A/HongKong/1-9-MA21-3/1968(H3N2) | 1 | 8 | No extrapolation |
|  |  |  |  |  |  |  |  |
| HK1073 | Human | AJ404630 | GenBank | A/HongKong/1073/1999(H9N2) | 1 | 1 | No extrapolation |
| HK1073 | Human | AJ404634 | GenBank | A/HongKong/1073/1999(H9N2) | 1 | 2 | No extrapolation |
| HK1073 | Human | AJ404637 | GenBank | A/HongKong/1073/1999(H9N2) | 1 | 3 | No extrapolation |
| HK1073 | Human | AJ404626 | GenBank | A/HongKong/1073/1999(H9N2) | 1 | 4 | No extrapolation |
| HK1073 | Human | AJ289871 | GenBank | A/HongKong/1073/1999(H9N2) | 1 | 5 | No extrapolation |
| HK1073 | Human | AJ404629 | GenBank | A/HongKong/1073/1999(H9N2) | 1 | 6 | No extrapolation |
| HK1073 | Human | AJ278646 | GenBank | A/HongKong/1073/1999(H9N2) | 1 | 7 | No extrapolation |
| HK1073 | Human | AJ278649 | GenBank | A/HongKong/1073/1999(H9N2) | 1 | 8 | No extrapolation |
|  |  |  |  |  |  |  |  |
| HK156v1 | Human | AF036363 | GenBank | A/HongKong/156/1997(H5N1) | 1 | 1 | No extrapolation |
| HK156v1 | Human | AF036362 | GenBank | A/HongKong/156/1997(H5N1) | 1 | 2 | No extrapolation |
| HK156v1 | Human | AF036361 | GenBank | A/HongKong/156/1997(H5N1) | 1 | 3 | Partial |
| HK156v1 | Human | AF036356 | GenBank | A/HongKong/156/1997(H5N1) | 1 | 4 | Partial |
| HK156v1 | Human | AF036359 | GenBank | A/HongKong/156/1997(H5N1) | 1 | 5 | No extrapolation |
| HK156v1 | Human | AF036357 | GenBank | A/HongKong/156/1997(H5N1) | 1 | 6 | No extrapolation |
| HK156v1 | Human | AF036358 | GenBank | A/HongKong/156/1997(H5N1) | 1 | 7 | No extrapolation |
| HK156v1 | Human | AF036360 | GenBank | A/HongKong/156/1997(H5N1) | 1 | 8 | No extrapolation |
|  |  |  |  |  |  |  |  |
| HK156v2 | Human | AF046093 | GenBank | A/HongKong/156/1997(H5N1) | 2 | 1 | Partial |
| HK156v2 | Human | AF046094 | GenBank | A/HongKong/156/1997(H5N1) | 2 | 2 | No extrapolation |
| HK156v2 | Human | AF046095 | GenBank | A/HongKong/156/1997(H5N1) | 2 | 3 | No extrapolation |
| HK156v2 | Human | AF046088 | GenBank | A/HongKong/156/1997(H5N1) | 2 | 4 | No extrapolation |
| HK156v2 | Human | AF046092 | GenBank | A/HongKong/156/1997(H5N1) | 2 | 5 | No extrapolation |
| HK156v2 | Human | AF046089 | GenBank | A/HongKong/156/1997(H5N1) | 2 | 6 | No extrapolation |
| HK156v2 | Human | AF046090 | GenBank | A/HongKong/156/1997(H5N1) | 2 | 7 | No extrapolation |
| HK156v2 | Human | AF046091 | GenBank | A/HongKong/156/1997(H5N1) | 2 | 8 | No extrapolation |
|  |  |  |  |  |  |  |  |
| HK481v1 | Human | AF258837 | GenBank | A/HongKong/481/1997(H5N1) | 1 | 1 | No extrapolation |
| HK481v1 | Human | AF258818 | GenBank | A/HongKong/481/1997(H5N1) | 1 | 2 | No extrapolation |
| HK481v1 | Human | AF257193 | GenBank | A/HongKong/481/1997(H5N1) | 1 | 3 | No extrapolation |
| HK481v1 | Human | AF046096 | GenBank | A/HongKong/481/1997(H5N1) | 1 | 4 | No extrapolation |
| HK481v1 | Human | AF255744 | GenBank | A/HongKong/481/1997(H5N1) | 1 | 5 | No extrapolation |
| HK481v1 | Human | AF102663 | GenBank | A/HongKong/481/1997(H5N1) | 1 | 6 | Partial |
| HK481v1 | Human | AF255365 | GenBank | A/HongKong/481/1997(H5N1) | 1 | 7 | No extrapolation |
| HK481v1 | Human | AF256178 | GenBank | A/HongKong/481/1997(H5N1) | 1 | 8 | No extrapolation |
|  |  |  |  |  |  |  |  |
| HK481v2 | Human | AF115290 | GenBank | A/HongKong/481/1997(H5N1) | 2 | 1 | No extrapolation |
| HK481v2 | Human | AF115292 | GenBank | A/HongKong/481/1997(H5N1) | 2 | 2 | No extrapolation |
| HK481v2 | Human | AF115294 | GenBank | A/HongKong/481/1997(H5N1) | 2 | 3 | No extrapolation |
| HK481v2 | Human | AF084279 | GenBank | A/HongKong/481/1997(H5N1) | 2 | 4 | Partial |
| HK481v2 | Human | AF115284 | GenBank | A/HongKong/481/1997(H5N1) | 2 | 5 | No extrapolation |
| HK481v2 | Human | AF084271 | GenBank | A/HongKong/481/1997(H5N1) | 2 | 6 | No extrapolation |
| HK481v2 | Human | AF115286 | GenBank | A/HongKong/481/1997(H5N1) | 2 | 7 | No extrapolation |
| HK481v2 | Human | AF115288 | GenBank | A/HongKong/481/1997(H5N1) | 2 | 8 | No extrapolation |
|  |  |  |  |  |  |  |  |
| HK483v1 | Human | AF258839 | GenBank | A/HongKong/483/1997(H5N1) | 2 | 1 | No extrapolation |
| HK483v1 | Human | AF258820 | GenBank | A/HongKong/483/1997(H5N1) | 2 | 2 | No extrapolation |
| HK483v1 | Human | AF257195 | GenBank | A/HongKong/483/1997(H5N1) | 2 | 3 | No extrapolation |
| HK483v1 | Human | AF046097 | GenBank | A/HongKong/483/1997(H5N1) | 2 | 4 | No extrapolation |
| HK483v1 | Human | AF255746 | GenBank | A/HongKong/483/1997(H5N1) | 2 | 5 | No extrapolation |
| HK483v1 | Human | AF102668 | GenBank | A/HongKong/483/1997(H5N1) | 2 | 6 | No extrapolation |
| HK483v1 | Human | AF255367 | GenBank | A/HongKong/483/1997(H5N1) | 2 | 7 | No extrapolation |
| HK483v1 | Human | AF256180 | GenBank | A/HongKong/483/1997(H5N1) | 2 | 8 | No extrapolation |
|  |  |  |  |  |  |  |  |
| HK483v2 | Human | AF084262 | GenBank | A/HongKong/483/1997(H5N1) | 1 | 1 | No extrapolation |
| HK483v2 | Human | AF084265 | GenBank | A/HongKong/483/1997(H5N1) | 1 | 2 | No extrapolation |
| HK483v2 | Human | AF084269 | GenBank | A/HongKong/483/1997(H5N1) | 1 | 3 | No extrapolation |
| HK483v2 | Human | AF084280 | GenBank | A/HongKong/483/1997(H5N1) | 1 | 4 | Partial |
| HK483v2 | Human | AF084277 | GenBank | A/HongKong/483/1997(H5N1) | 1 | 5 | No extrapolation |
| HK483v2 | Human | AF084273 | GenBank | A/HongKong/483/1997(H5N1) | 1 | 6 | No extrapolation |
| HK483v2 | Human | AF084283 | GenBank | A/HongKong/483/1997(H5N1) | 1 | 7 | No extrapolation |
| HK483v2 | Human | AF084286 | GenBank | A/HongKong/483/1997(H5N1) | 1 | 8 | No extrapolation |
|  |  |  |  |  |  |  |  |
| HK483v3 | Human | GU052104 | GenBank | A/HongKong/483/1997(H5N1) | 3 | 1 | No extrapolation |
| HK483v3 | Human | GU052103 | GenBank | A/HongKong/483/1997(H5N1) | 3 | 2 | Partial |
| HK483v3 | Human | GU052102 | GenBank | A/HongKong/483/1997(H5N1) | 3 | 3 | No extrapolation |
| HK483v3 | Human | GU052097 | GenBank | A/HongKong/483/1997(H5N1) | 3 | 4 | No extrapolation |
| HK483v3 | Human | GU052100 | GenBank | A/HongKong/483/1997(H5N1) | 3 | 5 | Partial |
| HK483v3 | Human | GU052099 | GenBank | A/HongKong/483/1997(H5N1) | 3 | 6 | No extrapolation |
| HK483v3 | Human | GU052098 | GenBank | A/HongKong/483/1997(H5N1) | 3 | 7 | No extrapolation |
| HK483v3 | Human | GU052101 | GenBank | A/HongKong/483/1997(H5N1) | 3 | 8 | No extrapolation |
|  |  |  |  |  |  |  |  |
| HK485v1 | Human | GU052149 | GenBank | A/HongKong/485/1997(H5N1) | 1 | 1 | No extrapolation |
| HK485v1 | Human | GU052148 | GenBank | A/HongKong/485/1997(H5N1) | 1 | 2 | No extrapolation |
| HK485v1 | Human | GU052147 | GenBank | A/HongKong/485/1997(H5N1) | 1 | 3 | No extrapolation |
| HK485v1 | Human | GU052142 | GenBank | A/HongKong/485/1997(H5N1) | 1 | 4 | No extrapolation |
| HK485v1 | Human | GU052145 | GenBank | A/HongKong/485/1997(H5N1) | 1 | 5 | No extrapolation |
| HK485v1 | Human | GU052144 | GenBank | A/HongKong/485/1997(H5N1) | 1 | 6 | No extrapolation |
| HK485v1 | Human | GU052143 | GenBank | A/HongKong/485/1997(H5N1) | 1 | 7 | Partial |
| HK485v1 | Human | GU052146 | GenBank | A/HongKong/485/1997(H5N1) | 1 | 8 | No extrapolation |
|  |  |  |  |  |  |  |  |
| HK485v2 | Human | AF084263 | GenBank | A/HongKong/485/1997(H5N1) | 2 | 1 | No extrapolation |
| HK485v2 | Human | AF084266 | GenBank | A/HongKong/485/1997(H5N1) | 2 | 2 | No extrapolation |
| HK485v2 | Human | AF084270 | GenBank | A/HongKong/485/1997(H5N1) | 2 | 3 | No extrapolation |
| HK485v2 | Human | AF084532 | GenBank | A/HongKong/485/1997(H5N1) | 2 | 4 | Partial |
| HK485v2 | Human | AF084278 | GenBank | A/HongKong/485/1997(H5N1) | 2 | 5 | No extrapolation |
| HK485v2 | Human | AF084274 | GenBank | A/HongKong/485/1997(H5N1) | 2 | 6 | No extrapolation |
| HK485v2 | Human | AF084284 | GenBank | A/HongKong/485/1997(H5N1) | 2 | 7 | No extrapolation |
| HK485v2 | Human | AF084287 | GenBank | A/HongKong/485/1997(H5N1) | 2 | 8 | No extrapolation |
|  |  |  |  |  |  |  |  |
| HK485v3 | Human | AF258847 | GenBank | A/HongKong/485/1997(H5N1) | 1 | 1 | Partial |
| HK485v3 | Human | AF258828 | GenBank | A/HongKong/485/1997(H5N1) | 1 | 2 | Partial |
| HK485v3 | Human | AF257203 | GenBank | A/HongKong/485/1997(H5N1) | 1 | 3 | Partial |
| HK485v3 | Human | AF102681 | GenBank | A/HongKong/485/1997(H5N1) | 1 | 4 | Partial |
| HK485v3 | Human | AH010699 | GenBank | A/HongKong/485/1997(H5N1) | 1 | 5 | Partial |
| HK485v3 | Human | AF102664 | GenBank | A/HongKong/485/1997(H5N1) | 1 | 6 | Partial |
| HK485v3 | Human | AH010696 | GenBank | A/HongKong/485/1997(H5N1) | 1 | 7 | Partial |
| HK485v3 | Human | AF256189 | GenBank | A/HongKong/485/1997(H5N1) | 1 | 8 | Partial |
|  |  |  |  |  |  |  |  |
| HK486v1 | Human | AF258840 | GenBank | A/HongKong/486/1997(H5N1) | 1 | 1 | No extrapolation |
| HK486v1 | Human | AF258821 | GenBank | A/HongKong/486/1997(H5N1) | 1 | 2 | No extrapolation |
| HK486v1 | Human | AF257196 | GenBank | A/HongKong/486/1997(H5N1) | 1 | 3 | No extrapolation |
| HK486v1 | Human | AF102671 | GenBank | A/HongKong/486/1997(H5N1) | 1 | 4 | Partial |
| HK486v1 | Human | AF255747 | GenBank | A/HongKong/486/1997(H5N1) | 1 | 5 | No extrapolation |
| HK486v1 | Human | AF102658 | GenBank | A/HongKong/486/1997(H5N1) | 1 | 6 | No extrapolation |
| HK486v1 | Human | AF255368 | GenBank | A/HongKong/486/1997(H5N1) | 1 | 7 | No extrapolation |
| HK486v1 | Human | AF256181 | GenBank | A/HongKong/486/1997(H5N1) | 1 | 8 | No extrapolation |
|  |  |  |  |  |  |  |  |
| HK486v2 | Human | AF115291 | GenBank | A/HongKong/486/1997(H5N1) | 2 | 1 | No extrapolation |
| HK486v2 | Human | AF115293 | GenBank | A/HongKong/486/1997(H5N1) | 2 | 2 | No extrapolation |
| HK486v2 | Human | AF115295 | GenBank | A/HongKong/486/1997(H5N1) | 2 | 3 | No extrapolation |
| HK486v2 | Human | AF084281 | GenBank | A/HongKong/486/1997(H5N1) | 2 | 4 | Partial |
| HK486v2 | Human | AF115285 | GenBank | A/HongKong/486/1997(H5N1) | 2 | 5 | No extrapolation |
| HK486v2 | Human | AF084275 | GenBank | A/HongKong/486/1997(H5N1) | 2 | 6 | No extrapolation |
| HK486v2 | Human | AF115287 | GenBank | A/HongKong/486/1997(H5N1) | 2 | 7 | No extrapolation |
| HK486v2 | Human | AF115289 | GenBank | A/HongKong/486/1997(H5N1) | 2 | 8 | No extrapolation |
|  |  |  |  |  |  |  |  |
| HK488 | Human | AF258848 | GenBank | A/HongKong/488/1997(H5N1) | 1 | 1 | Partial |
| HK488 | Human | AF258829 | GenBank | A/HongKong/488/1997(H5N1) | 1 | 2 | Partial |
| HK488 | Human | AF257204 | GenBank | A/HongKong/488/1997(H5N1) | 1 | 3 | Partial |
| HK488 | Human | AF102672 | GenBank | A/HongKong/488/1997(H5N1) | 1 | 4 | Partial |
| HK488 | Human | AH010700 | GenBank | A/HongKong/488/1997(H5N1) | 1 | 5 | Partial |
| HK488 | Human | AF102657 | GenBank | A/HongKong/488/1997(H5N1) | 1 | 6 | Partial |
| HK488 | Human | AH010697 | GenBank | A/HongKong/488/1997(H5N1) | 1 | 7 | Partial |
| HK488 | Human | AF256190 | GenBank | A/HongKong/488/1997(H5N1) | 1 | 8 | Partial |
|  |  |  |  |  |  |  |  |
| HK491 | Human | AF258849 | GenBank | A/HongKong/491/1997(H5N1) | 1 | 1 | Partial |
| HK491 | Human | AF258830 | GenBank | A/HongKong/491/1997(H5N1) | 1 | 2 | Partial |
| HK491 | Human | AF257205 | GenBank | A/HongKong/491/1997(H5N1) | 1 | 3 | Partial |
| HK491 | Human | AF102677 | GenBank | A/HongKong/491/1997(H5N1) | 1 | 4 | Partial |
| HK491 | Human | AH010701 | GenBank | A/HongKong/491/1997(H5N1) | 1 | 5 | Partial |
| HK491 | Human | AF102665 | GenBank | A/HongKong/491/1997(H5N1) | 1 | 6 | Partial |
| HK491 | Human | AH010698 | GenBank | A/HongKong/491/1997(H5N1) | 1 | 7 | Partial |
| HK491 | Human | AF256191 | GenBank | A/HongKong/491/1997(H5N1) | 1 | 8 | Partial |
|  |  |  |  |  |  |  |  |
| HK503 | Human | AF258850 | GenBank | A/HongKong/503/1997(H5N1) | 1 | 1 | Partial |
| HK503 | Human | AF258831 | GenBank | A/HongKong/503/1997(H5N1) | 1 | 2 | Partial |
| HK503 | Human | AF257206 | GenBank | A/HongKong/503/1997(H5N1) | 1 | 3 | Partial |
| HK503 | Human | AF102679 | GenBank | A/HongKong/503/1997(H5N1) | 1 | 4 | Partial |
| HK503 | Human | AH010702 | GenBank | A/HongKong/503/1997(H5N1) | 1 | 5 | Partial |
| HK503 | Human | AF102666 | GenBank | A/HongKong/503/1997(H5N1) | 1 | 6 | Partial |
| HK503 | Human | AF255381 | GenBank | A/HongKong/503/1997(H5N1) | 1 | 7 | Partial |
| HK503 | Human | AF256192 | GenBank | A/HongKong/503/1997(H5N1) | 1 | 8 | Partial |
|  |  |  |  |  |  |  |  |
| HK507 | Human | AF258851 | GenBank | A/HongKong/507/1997(H5N1) | 1 | 1 | Partial |
| HK507 | Human | AF258832 | GenBank | A/HongKong/507/1997(H5N1) | 1 | 2 | Partial |
| HK507 | Human | AF257207 | GenBank | A/HongKong/507/1997(H5N1) | 1 | 3 | Partial |
| HK507 | Human | AF102675 | GenBank | A/HongKong/507/1997(H5N1) | 1 | 4 | Partial |
| HK507 | Human | AH010703 | GenBank | A/HongKong/507/1997(H5N1) | 1 | 5 | Partial |
| HK507 | Human | AF102659 | GenBank | A/HongKong/507/1997(H5N1) | 1 | 6 | Partial |
| HK507 | Human | AF255382 | GenBank | A/HongKong/507/1997(H5N1) | 1 | 7 | Partial |
| HK507 | Human | AF256193 | GenBank | A/HongKong/507/1997(H5N1) | 1 | 8 | Partial |
|  |  |  |  |  |  |  |  |
| HK514 | Human | AF258852 | GenBank | A/HongKong/514/1997(H5N1) | 1 | 1 | Partial |
| HK514 | Human | AF258833 | GenBank | A/HongKong/514/1997(H5N1) | 1 | 2 | Partial |
| HK514 | Human | AF257208 | GenBank | A/HongKong/514/1997(H5N1) | 1 | 3 | Partial |
| HK514 | Human | AF102682 | GenBank | A/HongKong/514/1997(H5N1) | 1 | 4 | Partial |
| HK514 | Human | AH010704 | GenBank | A/HongKong/514/1997(H5N1) | 1 | 5 | Partial |
| HK514 | Human | AF102669 | GenBank | A/HongKong/514/1997(H5N1) | 1 | 6 | Partial |
| HK514 | Human | AF255383 | GenBank | A/HongKong/514/1997(H5N1) | 1 | 7 | Partial |
| HK514 | Human | AF256184 | GenBank | A/HongKong/514/1997(H5N1) | 1 | 8 | No extrapolation |
|  |  |  |  |  |  |  |  |
| HK516 | Human | AF258853 | GenBank | A/HongKong/516/1997(H5N1) | 1 | 1 | Partial |
| HK516 | Human | AF258834 | GenBank | A/HongKong/516/1997(H5N1) | 1 | 2 | Partial |
| HK516 | Human | AF257209 | GenBank | A/HongKong/516/1997(H5N1) | 1 | 3 | Partial |
| HK516 | Human | AF102673 | GenBank | A/HongKong/516/1997(H5N1) | 1 | 4 | Partial |
| HK516 | Human | AH010705 | GenBank | A/HongKong/516/1997(H5N1) | 1 | 5 | Partial |
| HK516 | Human | AF102660 | GenBank | A/HongKong/516/1997(H5N1) | 1 | 6 | Partial |
| HK516 | Human | AF255384 | GenBank | A/HongKong/516/1997(H5N1) | 1 | 7 | Partial |
| HK516 | Human | AF256194 | GenBank | A/HongKong/516/1997(H5N1) | 1 | 8 | Partial |
|  |  |  |  |  |  |  |  |
| HK532 | Human | AF258843 | GenBank | A/HongKong/532/1997(H5N1) | 1 | 1 | No extrapolation |
| HK532 | Human | AF258824 | GenBank | A/HongKong/532/1997(H5N1) | 1 | 2 | No extrapolation |
| HK532 | Human | AF257199 | GenBank | A/HongKong/532/1997(H5N1) | 1 | 3 | No extrapolation |
| HK532 | Human | AF102680 | GenBank | A/HongKong/532/1997(H5N1) | 1 | 4 | Partial |
| HK532 | Human | AF255750 | GenBank | A/HongKong/532/1997(H5N1) | 1 | 5 | No extrapolation |
| HK532 | Human | AF102667 | GenBank | A/HongKong/532/1997(H5N1) | 1 | 6 | Partial |
| HK532 | Human | AF255371 | GenBank | A/HongKong/532/1997(H5N1) | 1 | 7 | No extrapolation |
| HK532 | Human | AF256185 | GenBank | A/HongKong/532/1997(H5N1) | 1 | 8 | No extrapolation |
|  |  |  |  |  |  |  |  |
| HK538 | Human | AF258844 | GenBank | A/HongKong/538/1997(H5N1) | 1 | 1 | No extrapolation |
| HK538 | Human | AF258825 | GenBank | A/HongKong/538/1997(H5N1) | 1 | 2 | No extrapolation |
| HK538 | Human | AF257200 | GenBank | A/HongKong/538/1997(H5N1) | 1 | 3 | No extrapolation |
| HK538 | Human | AF102674 | GenBank | A/HongKong/538/1997(H5N1) | 1 | 4 | Partial |
| HK538 | Human | AF255751 | GenBank | A/HongKong/538/1997(H5N1) | 1 | 5 | No extrapolation |
| HK538 | Human | AF102662 | GenBank | A/HongKong/538/1997(H5N1) | 1 | 6 | Partial |
| HK538 | Human | AF255372 | GenBank | A/HongKong/538/1997(H5N1) | 1 | 7 | No extrapolation |
| HK538 | Human | AF256186 | GenBank | A/HongKong/538/1997(H5N1) | 1 | 8 | No extrapolation |
|  |  |  |  |  |  |  |  |
| HK542 | Human | AF258845 | GenBank | A/HongKong/542/1997(H5N1) | 1 | 1 | No extrapolation |
| HK542 | Human | AF258826 | GenBank | A/HongKong/542/1997(H5N1) | 1 | 2 | No extrapolation |
| HK542 | Human | AF257201 | GenBank | A/HongKong/542/1997(H5N1) | 1 | 3 | No extrapolation |
| HK542 | Human | AF102678 | GenBank | A/HongKong/542/1997(H5N1) | 1 | 4 | Partial |
| HK542 | Human | AF255752 | GenBank | A/HongKong/542/1997(H5N1) | 1 | 5 | No extrapolation |
| HK542 | Human | AF102670 | GenBank | A/HongKong/542/1997(H5N1) | 1 | 6 | Partial |
| HK542 | Human | AF255373 | GenBank | A/HongKong/542/1997(H5N1) | 1 | 7 | No extrapolation |
| HK542 | Human | AF256187 | GenBank | A/HongKong/542/1997(H5N1) | 1 | 8 | No extrapolation |
|  |  |  |  |  |  |  |  |
| HK68v1 | Human | AF348170 | GenBank | A/HongKong/1/1968(H3N2) | 1 | 1 | No extrapolation |
| HK68v1 | Human | AF348172 | GenBank | A/HongKong/1/1968(H3N2) | 1 | 2 | No extrapolation |
| HK68v1 | Human | AF348174 | GenBank | A/HongKong/1/1968(H3N2) | 1 | 3 | No extrapolation |
| HK68v1 | Human | AF348176 | GenBank | A/HongKong/1/1968(H3N2) | 1 | 4 | No extrapolation |
| HK68v1 | Human | AF348180 | GenBank | A/HongKong/1/1968(H3N2) | 1 | 5 | No extrapolation |
| HK68v1 | Human | AF348184 | GenBank | A/HongKong/1/1968(H3N2) | 1 | 6 | No extrapolation |
| HK68v1 | Human | AF348188 | GenBank | A/HongKong/1/1968(H3N2) | 1 | 7 | No extrapolation |
| HK68v1 | Human | AF348198 | GenBank | A/HongKong/1/1968(H3N2) | 1 | 8 | No extrapolation |
|  |  |  |  |  |  |  |  |
| HK68v2 | Human | CY112256 | GenBank | A/HongKong/1/1968(H3N2) | 2 | 1 | No extrapolation |
| HK68v2 | Human | CY112255 | GenBank | A/HongKong/1/1968(H3N2) | 2 | 2 | No extrapolation |
| HK68v2 | Human | CY112254 | GenBank | A/HongKong/1/1968(H3N2) | 2 | 3 | No extrapolation |
| HK68v2 | Human | CY112249 | GenBank | A/HongKong/1/1968(H3N2) | 2 | 4 | No extrapolation |
| HK68v2 | Human | CY112252 | GenBank | A/HongKong/1/1968(H3N2) | 2 | 5 | No extrapolation |
| HK68v2 | Human | CY112251 | GenBank | A/HongKong/1/1968(H3N2) | 2 | 6 | No extrapolation |
| HK68v2 | Human | CY112250 | GenBank | A/HongKong/1/1968(H3N2) | 2 | 7 | No extrapolation |
| HK68v2 | Human | CY112253 | GenBank | A/HongKong/1/1968(H3N2) | 2 | 8 | No extrapolation |
|  |  |  |  |  |  |  |  |
| HK68v3 | Human | CY044268 | GenBank | A/HongKong/1/1968(H3N2) | 3 | 1 | No extrapolation |
| HK68v3 | Human | CY044267 | GenBank | A/HongKong/1/1968(H3N2) | 3 | 2 | No extrapolation |
| HK68v3 | Human | CY044266 | GenBank | A/HongKong/1/1968(H3N2) | 3 | 3 | No extrapolation |
| HK68v3 | Human | CY044261 | GenBank | A/HongKong/1/1968(H3N2) | 3 | 4 | No extrapolation |
| HK68v3 | Human | CY044264 | GenBank | A/HongKong/1/1968(H3N2) | 3 | 5 | No extrapolation |
| HK68v3 | Human | CY044263 | GenBank | A/HongKong/1/1968(H3N2) | 3 | 6 | No extrapolation |
| HK68v3 | Human | CY044262 | GenBank | A/HongKong/1/1968(H3N2) | 3 | 7 | No extrapolation |
| HK68v3 | Human | CY044265 | GenBank | A/HongKong/1/1968(H3N2) | 3 | 8 | No extrapolation |
|  |  |  |  |  |  |  |  |
| HK97 | Human | AF258846 | GenBank | A/HongKong/97/1998(H5N1) | 1 | 1 | No extrapolation |
| HK97 | Human | AF258827 | GenBank | A/HongKong/97/1998(H5N1) | 1 | 2 | No extrapolation |
| HK97 | Human | AF257202 | GenBank | A/HongKong/97/1998(H5N1) | 1 | 3 | No extrapolation |
| HK97 | Human | AF102676 | GenBank | A/HongKong/97/1998(H5N1) | 1 | 4 | Partial |
| HK97 | Human | AF255753 | GenBank | A/HongKong/97/1998(H5N1) | 1 | 5 | No extrapolation |
| HK97 | Human | AF102661 | GenBank | A/HongKong/97/1998(H5N1) | 1 | 6 | Partial |
| HK97 | Human | AF255374 | GenBank | A/HongKong/97/1998(H5N1) | 1 | 7 | No extrapolation |
| HK97 | Human | AF256188 | GenBank | A/HongKong/97/1998(H5N1) | 1 | 8 | No extrapolation |
|  |  |  |  |  |  |  |  |
| HUN42 | Human | EPI691392 | GISAID | A/Hunan/42443/2015(H1N1) | 1 | 1 | No extrapolation |
| HUN42 | Human | EPI691393 | GISAID | A/Hunan/42443/2015(H1N1) | 1 | 2 | No extrapolation |
| HUN42 | Human | EPI691394 | GISAID | A/Hunan/42443/2015(H1N1) | 1 | 3 | No extrapolation |
| HUN42 | Human | EPI691395 | GISAID | A/Hunan/42443/2015(H1N1) | 1 | 4 | No extrapolation |
| HUN42 | Human | EPI691396 | GISAID | A/Hunan/42443/2015(H1N1) | 1 | 5 | No extrapolation |
| HUN42 | Human | EPI691397 | GISAID | A/Hunan/42443/2015(H1N1) | 1 | 6 | No extrapolation |
| HUN42 | Human | EPI691398 | GISAID | A/Hunan/42443/2015(H1N1) | 1 | 7 | No extrapolation |
| HUN42 | Human | EPI691399 | GISAID | A/Hunan/42443/2015(H1N1) | 1 | 8 | No extrapolation |
|  |  |  |  |  |  |  |  |
| HZ-3 | Avian | EPI917099 | GISAID | A/chicken/Huizhou/HZ-3/2016(H7N9) | 1 | 1 | No extrapolation |
| HZ-3 | Avian | EPI917100 | GISAID | A/chicken/Huizhou/HZ-3/2016(H7N9) | 1 | 2 | No extrapolation |
| HZ-3 | Avian | EPI917101 | GISAID | A/chicken/Huizhou/HZ-3/2016(H7N9) | 1 | 3 | No extrapolation |
| HZ-3 | Avian | EPI917102 | GISAID | A/chicken/Huizhou/HZ-3/2016(H7N9) | 1 | 4 | No extrapolation |
| HZ-3 | Avian | EPI917103 | GISAID | A/chicken/Huizhou/HZ-3/2016(H7N9) | 1 | 5 | No extrapolation |
| HZ-3 | Avian | EPI917104 | GISAID | A/chicken/Huizhou/HZ-3/2016(H7N9) | 1 | 6 | No extrapolation |
| HZ-3 | Avian | EPI917105 | GISAID | A/chicken/Huizhou/HZ-3/2016(H7N9) | 1 | 7 | No extrapolation |
| HZ-3 | Avian | EPI917106 | GISAID | A/chicken/Huizhou/HZ-3/2016(H7N9) | 1 | 8 | No extrapolation |
|  |  |  |  |  |  |  |  |
| INA03 | Avian | GU186744 | GenBank | A/chicken/Indonesia/7/2003(H5N1) | 1 | 1 | No extrapolation |
| INA03 | Avian | GU186743 | GenBank | A/chicken/Indonesia/7/2003(H5N1) | 1 | 2 | No extrapolation |
| INA03 | Avian | GU186742 | GenBank | A/chicken/Indonesia/7/2003(H5N1) | 1 | 3 | No extrapolation |
| INA03 | Avian | EF473080 | GenBank | A/chicken/Indonesia/7/2003(H5N1) | 1 | 4 | No extrapolation |
| INA03 | Avian | GU186740 | GenBank | A/chicken/Indonesia/7/2003(H5N1) | 1 | 5 | No extrapolation |
| INA03 | Avian | EF473082 | GenBank | A/chicken/Indonesia/7/2003(H5N1) | 1 | 6 | No extrapolation |
| INA03 | Avian | EF473079 | GenBank | A/chicken/Indonesia/7/2003(H5N1) | 1 | 7 | No extrapolation |
| INA03 | Avian | GU186741 | GenBank | A/chicken/Indonesia/7/2003(H5N1) | 1 | 8 | No extrapolation |
|  |  |  |  |  |  |  |  |
| INA5 | Human | CY116643 | GenBank | A/Indonesia/5/2005(H5N1) | 1 | 1 | No extrapolation |
| INA5 | Human | CY116644 | GenBank | A/Indonesia/5/2005(H5N1) | 1 | 2 | No extrapolation |
| INA5 | Human | CY116645 | GenBank | A/Indonesia/5/2005(H5N1) | 1 | 3 | No extrapolation |
| INA5 | Human | CY116646 | GenBank | A/Indonesia/5/2005(H5N1) | 1 | 4 | No extrapolation |
| INA5 | Human | CY116647 | GenBank | A/Indonesia/5/2005(H5N1) | 1 | 5 | No extrapolation |
| INA5 | Human | CY116648 | GenBank | A/Indonesia/5/2005(H5N1) | 1 | 6 | No extrapolation |
| INA5 | Human | CY116649 | GenBank | A/Indonesia/5/2005(H5N1) | 1 | 7 | No extrapolation |
| INA5 | Human | CY116650 | GenBank | A/Indonesia/5/2005(H5N1) | 1 | 8 | No extrapolation |
|  |  |  |  |  |  |  |  |
| JIA11v1 | Human | KF057091 | GenBank | A/Jiangsu/1/2011(H1N1) | 1 | 1 | No extrapolation |
| JIA11v1 | Human | KF057098 | GenBank | A/Jiangsu/1/2011(H1N1) | 1 | 2 | No extrapolation |
| JIA11v1 | Human | KF057105 | GenBank | A/Jiangsu/1/2011(H1N1) | 1 | 3 | No extrapolation |
| JIA11v1 | Human | KF057112 | GenBank | A/Jiangsu/1/2011(H1N1) | 1 | 4 | No extrapolation |
| JIA11v1 | Human | KF057119 | GenBank | A/Jiangsu/1/2011(H1N1) | 1 | 5 | No extrapolation |
| JIA11v1 | Human | KF057126 | GenBank | A/Jiangsu/1/2011(H1N1) | 1 | 6 | No extrapolation |
| JIA11v1 | Human | KF057133 | GenBank | A/Jiangsu/1/2011(H1N1) | 1 | 7 | No extrapolation |
| JIA11v1 | Human | KF057140 | GenBank | A/Jiangsu/1/2011(H1N1) | 1 | 8 | No extrapolation |
|  |  |  |  |  |  |  |  |
| JIA11v2 | Human | EPI301163 | GISAID | A/Jiangsu/1/2011(H1N1) | 2 | 1 | No extrapolation |
| JIA11v2 | Human | EPI301162 | GISAID | A/Jiangsu/1/2011(H1N1) | 2 | 2 | No extrapolation |
| JIA11v2 | Human | EPI301161 | GISAID | A/Jiangsu/1/2011(H1N1) | 2 | 3 | No extrapolation |
| JIA11v2 | Human | EPI301156 | GISAID | A/Jiangsu/1/2011(H1N1) | 2 | 4 | No extrapolation |
| JIA11v2 | Human | EPI301159 | GISAID | A/Jiangsu/1/2011(H1N1) | 2 | 5 | No extrapolation |
| JIA11v2 | Human | EPI301158 | GISAID | A/Jiangsu/1/2011(H1N1) | 2 | 6 | No extrapolation |
| JIA11v2 | Human | EPI301157 | GISAID | A/Jiangsu/1/2011(H1N1) | 2 | 7 | No extrapolation |
| JIA11v2 | Human | EPI301160 | GISAID | A/Jiangsu/1/2011(H1N1) | 2 | 8 | No extrapolation |
|  |  |  |  |  |  |  |  |
| JS1306 | Goose | EPI980837 | GISAID | A/goose/Jiangsu/1306/2014(H5N8) | 1 | 6 | No extrapolation |
|  |  |  |  |  |  |  |  |
| K173 | Human | CY003031 | GenBank | A/Kawasaki/173/2001(H1N1) | 1 | 1 | Full segment |
| K173 | Human | CY003030 | GenBank | A/Kawasaki/173/2001(H1N1) | 1 | 2 | Full segment |
| K173 | Human | CY003029 | GenBank | A/Kawasaki/173/2001(H1N1) | 1 | 3 | Full segment |
| K173 | Human | AB671296 | GenBank | A/Kawasaki/173/2001(H1N1) | 1 | 4 | No extrapolation |
| K173 | Human | CY003027 | GenBank | A/Kawasaki/173/2001(H1N1) | 1 | 5 | Full segment |
| K173 | Human | AB671297 | GenBank | A/Kawasaki/173/2001(H1N1) | 1 | 6 | No extrapolation |
| K173 | Human | CY003025 | GenBank | A/Kawasaki/173/2001(H1N1) | 1 | 7 | Full segment |
| K173 | Human | CY003028 | GenBank | A/Kawasaki/173/2001(H1N1) | 1 | 8 | Full segment |
|  |  |  |  |  |  |  |  |
| KANS07 | Swine | GQ484358 | GenBank | A/swine/Kansas/77778/2007(H1N1) | 1 | 1 | No extrapolation |
| KANS07 | Swine | GQ484357 | GenBank | A/swine/Kansas/77778/2007(H1N1) | 1 | 2 | No extrapolation |
| KANS07 | Swine | GQ484359 | GenBank | A/swine/Kansas/77778/2007(H1N1) | 1 | 3 | No extrapolation |
| KANS07 | Swine | GQ484355 | GenBank | A/swine/Kansas/77778/2007(H1N1) | 1 | 4 | No extrapolation |
| KANS07 | Swine | GQ484360 | GenBank | A/swine/Kansas/77778/2007(H1N1) | 1 | 5 | No extrapolation |
| KANS07 | Swine | GQ484356 | GenBank | A/swine/Kansas/77778/2007(H1N1) | 1 | 6 | No extrapolation |
| KANS07 | Swine | GQ484361 | GenBank | A/swine/Kansas/77778/2007(H1N1) | 1 | 7 | No extrapolation |
| KANS07 | Swine | GQ484362 | GenBank | A/swine/Kansas/77778/2007(H1N1) | 1 | 8 | No extrapolation |
|  |  |  |  |  |  |  |  |
| KOR03 | Avian | AY676023 | GenBank | A/chicken/Korea/es/2003(H5N1) | 1 | 1 | No extrapolation |
| KOR03 | Avian | AY676027 | GenBank | A/chicken/Korea/es/2003(H5N1) | 1 | 2 | No extrapolation |
| KOR03 | Avian | AY676031 | GenBank | A/chicken/Korea/es/2003(H5N1) | 1 | 3 | No extrapolation |
| KOR03 | Avian | AY676035 | GenBank | A/chicken/Korea/es/2003(H5N1) | 1 | 4 | No extrapolation |
| KOR03 | Avian | AY676039 | GenBank | A/chicken/Korea/es/2003(H5N1) | 1 | 5 | No extrapolation |
| KOR03 | Avian | AY676043 | GenBank | A/chicken/Korea/es/2003(H5N1) | 1 | 6 | No extrapolation |
| KOR03 | Avian | AY676047 | GenBank | A/chicken/Korea/es/2003(H5N1) | 1 | 7 | No extrapolation |
| KOR03 | Avian | AY676051 | GenBank | A/chicken/Korea/es/2003(H5N1) | 1 | 8 | No extrapolation |
|  |  |  |  |  |  |  |  |
| KOR09 | Human | GQ160811 | GenBank | A/Korea/01/2009(H1N1) | 1 | 1 | No extrapolation |
| KOR09 | Human | GQ160813 | GenBank | A/Korea/01/2009(H1N1) | 1 | 2 | No extrapolation |
| KOR09 | Human | GQ160812 | GenBank | A/Korea/01/2009(H1N1) | 1 | 3 | No extrapolation |
| KOR09 | Human | GQ131023 | GenBank | A/Korea/01/2009(H1N1) | 1 | 4 | No extrapolation |
| KOR09 | Human | GQ131024 | GenBank | A/Korea/01/2009(H1N1) | 1 | 5 | No extrapolation |
| KOR09 | Human | GQ132185 | GenBank | A/Korea/01/2009(H1N1) | 1 | 6 | No extrapolation |
| KOR09 | Human | GQ131025 | GenBank | A/Korea/01/2009(H1N1) | 1 | 7 | No extrapolation |
| KOR09 | Human | GQ131026 | GenBank | A/Korea/01/2009(H1N1) | 1 | 8 | No extrapolation |
|  |  |  |  |  |  |  |  |
| KW541 | Environment | KY273025 | GenBank | A/environment/Korea/W541/2016(H5N6) | 1 | 1 | No extrapolation |
| KW541 | Environment | KY273021 | GenBank | A/environment/Korea/W541/2016(H5N6) | 1 | 2 | No extrapolation |
| KW541 | Environment | KY273017 | GenBank | A/environment/Korea/W541/2016(H5N6) | 1 | 3 | No extrapolation |
| KW541 | Environment | KY272997 | GenBank | A/environment/Korea/W541/2016(H5N6) | 1 | 4 | No extrapolation |
| KW541 | Environment | KY273009 | GenBank | A/environment/Korea/W541/2016(H5N6) | 1 | 5 | No extrapolation |
| KW541 | Environment | KY273005 | GenBank | A/environment/Korea/W541/2016(H5N6) | 1 | 6 | No extrapolation |
| KW541 | Environment | KY273001 | GenBank | A/environment/Korea/W541/2016(H5N6) | 1 | 7 | No extrapolation |
| KW541 | Environment | KY273013 | GenBank | A/environment/Korea/W541/2016(H5N6) | 1 | 8 | No extrapolation |
|  |  |  |  |  |  |  |  |
| KW555 | Avian | KY576115 | GenBank | A/commonteal/Korea/W555/2017(H5N8) | 1 | 1 | No extrapolation |
| KW555 | Avian | KY576110 | GenBank | A/commonteal/Korea/W555/2017(H5N8) | 1 | 2 | No extrapolation |
| KW555 | Avian | KY576105 | GenBank | A/commonteal/Korea/W555/2017(H5N8) | 1 | 3 | No extrapolation |
| KW555 | Avian | KY576080 | GenBank | A/commonteal/Korea/W555/2017(H5N8) | 1 | 4 | No extrapolation |
| KW555 | Avian | KY576095 | GenBank | A/commonteal/Korea/W555/2017(H5N8) | 1 | 5 | No extrapolation |
| KW555 | Avian | KY576090 | GenBank | A/commonteal/Korea/W555/2017(H5N8) | 1 | 6 | No extrapolation |
| KW555 | Avian | KY576085 | GenBank | A/commonteal/Korea/W555/2017(H5N8) | 1 | 7 | No extrapolation |
| KW555 | Avian | KY576100 | GenBank | A/commonteal/Korea/W555/2017(H5N8) | 1 | 8 | No extrapolation |
|  |  |  |  |  |  |  |  |
| KYG11 | Equine | JX844143 | GenBank | A/equine/Kyonggi/SA1/2011(H3N8) | 1 | 1 | No extrapolation |
| KYG11 | Equine | JX844144 | GenBank | A/equine/Kyonggi/SA1/2011(H3N8) | 1 | 2 | No extrapolation |
| KYG11 | Equine | JX844145 | GenBank | A/equine/Kyonggi/SA1/2011(H3N8) | 1 | 3 | No extrapolation |
| KYG11 | Equine | JX844146 | GenBank | A/equine/Kyonggi/SA1/2011(H3N8) | 1 | 4 | No extrapolation |
| KYG11 | Equine | JX844147 | GenBank | A/equine/Kyonggi/SA1/2011(H3N8) | 1 | 5 | No extrapolation |
| KYG11 | Equine | JX844148 | GenBank | A/equine/Kyonggi/SA1/2011(H3N8) | 1 | 6 | No extrapolation |
| KYG11 | Equine | JX844149 | GenBank | A/equine/Kyonggi/SA1/2011(H3N8) | 1 | 7 | No extrapolation |
| KYG11 | Equine | JX844150 | GenBank | A/equine/Kyonggi/SA1/2011(H3N8) | 1 | 8 | No extrapolation |
|  |  |  |  |  |  |  |  |
| L1.12 | Human | KF897806 | GenBank | A/Lyon/1.12/2011(H1N1) | 1 | 1 | No extrapolation |
| L1.12 | Human | KF897807 | GenBank | A/Lyon/1.12/2011(H1N1) | 1 | 2 | No extrapolation |
| L1.12 | Human | KF897808 | GenBank | A/Lyon/1.12/2011(H1N1) | 1 | 3 | No extrapolation |
| L1.12 | Human | KF897809 | GenBank | A/Lyon/1.12/2011(H1N1) | 1 | 4 | No extrapolation |
| L1.12 | Human | KF897810 | GenBank | A/Lyon/1.12/2011(H1N1) | 1 | 5 | No extrapolation |
| L1.12 | Human | KF897811 | GenBank | A/Lyon/1.12/2011(H1N1) | 1 | 6 | No extrapolation |
| L1.12 | Human | KF897812 | GenBank | A/Lyon/1.12/2011(H1N1) | 1 | 7 | Partial |
| L1.12 | Human | KF897813 | GenBank | A/Lyon/1.12/2011(H1N1) | 1 | 8 | No extrapolation |
|  |  |  |  |  |  |  |  |
| L1337 | Human | CY043558 | GenBank | A/Lyon/1337/2007(H1N1) | 1 | 1 | Full segment |
| L1337 | Human | CY043559 | GenBank | A/Lyon/1337/2007(H1N1) | 1 | 2 | Full segment |
| L1337 | Human | CY043560 | GenBank | A/Lyon/1337/2007(H1N1) | 1 | 3 | Full segment |
| L1337 | Human | KP459007 | GenBank | A/Lyon/1337/2007(H1N1) | 1 | 4 | Partial |
| L1337 | Human | CY043562 | GenBank | A/Lyon/1337/2007(H1N1) | 1 | 5 | Full segment |
| L1337 | Human | HQ658485 | GenBank | A/Lyon/1337/2007(H1N1) | 1 | 6 | No extrapolation |
| L1337 | Human | CY031385 | GenBank | A/Lyon/1337/2007(H1N1) | 1 | 7 | No extrapolation |
| L1337 | Human | CY043565 | GenBank | A/Lyon/1337/2007(H1N1) | 1 | 8 | Full segment |
|  |  |  |  |  |  |  |  |
| L48.425 | Human | KY926041 | GenBank | A/Lyon/48.425/2009(H1N1) | 1 | 1 | Full segment |
| L48.425 | Human | KY925290 | GenBank | A/Lyon/48.425/2009(H1N1) | 1 | 2 | Full segment |
| L48.425 | Human | KY926136 | GenBank | A/Lyon/48.425/2009(H1N1) | 1 | 3 | Full segment |
| L48.425 | Human | JF429393 | GenBank | A/Lyon/48.425/2009(H1N1) | 1 | 4 | No extrapolation |
| L48.425 | Human | KY925820 | GenBank | A/Lyon/48.425/2009(H1N1) | 1 | 5 | Full segment |
| L48.425 | Human | JF429398 | GenBank | A/Lyon/48.425/2009(H1N1) | 1 | 6 | No extrapolation |
| L48.425 | Human | KY925621 | GenBank | A/Lyon/48.425/2009(H1N1) | 1 | 7 | Full segment |
| L48.425 | Human | KY926168 | GenBank | A/Lyon/48.425/2009(H1N1) | 1 | 8 | Full segment |
|  |  |  |  |  |  |  |  |
| L969 | Human | KC800977 | GenBank | A/Lyon/969/2009(H1N1) | 1 | 1 | No extrapolation |
| L969 | Human | KC800978 | GenBank | A/Lyon/969/2009(H1N1) | 1 | 2 | No extrapolation |
| L969 | Human | KC800979 | GenBank | A/Lyon/969/2009(H1N1) | 1 | 3 | No extrapolation |
| L969 | Human | JF429402 | GenBank | A/Lyon/969/2009(H1N1) | 1 | 4 | No extrapolation |
| L969 | Human | KC800980 | GenBank | A/Lyon/969/2009(H1N1) | 1 | 5 | No extrapolation |
| L969 | Human | JF429403 | GenBank | A/Lyon/969/2009(H1N1) | 1 | 6 | No extrapolation |
| L969 | Human | KC800981 | GenBank | A/Lyon/969/2009(H1N1) | 1 | 7 | No extrapolation |
| L969 | Human | KC800982 | GenBank | A/Lyon/969/2009(H1N1) | 1 | 8 | No extrapolation |
|  |  |  |  |  |  |  |  |
| LIMO10 | Human | KF897798 | GenBank | A/Limoges/1159/2010(H1N1) | 1 | 1 | No extrapolation |
| LIMO10 | Human | KF897799 | GenBank | A/Limoges/1159/2010(H1N1) | 1 | 2 | No extrapolation |
| LIMO10 | Human | KF897800 | GenBank | A/Limoges/1159/2010(H1N1) | 1 | 3 | No extrapolation |
| LIMO10 | Human | KF897801 | GenBank | A/Limoges/1159/2010(H1N1) | 1 | 4 | No extrapolation |
| LIMO10 | Human | KF897802 | GenBank | A/Limoges/1159/2010(H1N1) | 1 | 5 | No extrapolation |
| LIMO10 | Human | KF897803 | GenBank | A/Limoges/1159/2010(H1N1) | 1 | 6 | No extrapolation |
| LIMO10 | Human | KF897804 | GenBank | A/Limoges/1159/2010(H1N1) | 1 | 7 | Partial |
| LIMO10 | Human | KF897805 | GenBank | A/Limoges/1159/2010(H1N1) | 1 | 8 | No extrapolation |
|  |  |  |  |  |  |  |  |
| LN11 | Avian | KJ907644 | GenBank | A/duck/Liaoning/LN/2011(H5N5) | 1 | 1 | No extrapolation |
| LN11 | Avian | KJ907645 | GenBank | A/duck/Liaoning/LN/2011(H5N5) | 1 | 2 | No extrapolation |
| LN11 | Avian | KJ907646 | GenBank | A/duck/Liaoning/LN/2011(H5N5) | 1 | 3 | No extrapolation |
| LN11 | Avian | KJ907647 | GenBank | A/duck/Liaoning/LN/2011(H5N5) | 1 | 4 | No extrapolation |
| LN11 | Avian | KJ907648 | GenBank | A/duck/Liaoning/LN/2011(H5N5) | 1 | 5 | No extrapolation |
| LN11 | Avian | KJ907649 | GenBank | A/duck/Liaoning/LN/2011(H5N5) | 1 | 6 | No extrapolation |
| LN11 | Avian | KJ907650 | GenBank | A/duck/Liaoning/LN/2011(H5N5) | 1 | 7 | No extrapolation |
| LN11 | Avian | KJ907651 | GenBank | A/duck/Liaoning/LN/2011(H5N5) | 1 | 8 | No extrapolation |
|  |  |  |  |  |  |  |  |
| LR10 | Human | JX309663 | GenBank | A/LaReunion/803/2010(H1N1) | 1 | 1 | Full segment |
| LR10 | Human | KF897775 | GenBank | A/LaReunion/803/2010(H1N1) | 1 | 2 | No extrapolation |
| LR10 | Human | KF897776 | GenBank | A/LaReunion/803/2010(H1N1) | 1 | 3 | No extrapolation |
| LR10 | Human | KF897777 | GenBank | A/LaReunion/803/2010(H1N1) | 1 | 4 | No extrapolation |
| LR10 | Human | KF897778 | GenBank | A/LaReunion/803/2010(H1N1) | 1 | 5 | No extrapolation |
| LR10 | Human | KF897779 | GenBank | A/LaReunion/803/2010(H1N1) | 1 | 6 | No extrapolation |
| LR10 | Human | KF897780 | GenBank | A/LaReunion/803/2010(H1N1) | 1 | 7 | No extrapolation |
| LR10 | Human | KF897781 | GenBank | A/LaReunion/803/2010(H1N1) | 1 | 8 | No extrapolation |
|  |  |  |  |  |  |  |  |
| LYON10 | Human | KF897782 | GenBank | A/Lyon/52.16/2010(H1N1) | 1 | 1 | No extrapolation |
| LYON10 | Human | KF897783 | GenBank | A/Lyon/52.16/2010(H1N1) | 1 | 2 | No extrapolation |
| LYON10 | Human | KF897784 | GenBank | A/Lyon/52.16/2010(H1N1) | 1 | 3 | No extrapolation |
| LYON10 | Human | KF897785 | GenBank | A/Lyon/52.16/2010(H1N1) | 1 | 4 | No extrapolation |
| LYON10 | Human | KF897786 | GenBank | A/Lyon/52.16/2010(H1N1) | 1 | 5 | No extrapolation |
| LYON10 | Human | KF897787 | GenBank | A/Lyon/52.16/2010(H1N1) | 1 | 6 | No extrapolation |
| LYON10 | Human | KF897788 | GenBank | A/Lyon/52.16/2010(H1N1) | 1 | 7 | Partial |
| LYON10 | Human | KF897789 | GenBank | A/Lyon/52.16/2010(H1N1) | 1 | 8 | No extrapolation |
|  |  |  |  |  |  |  |  |
| LZF | Chicken | EPI980836 | GISAID | A/chicken/Hebei/LZF/2014(H5N2) | 1 | 6 | No extrapolation |
|  |  |  |  |  |  |  |  |
| M88 | Human | CY010763 | GenBank | A/Memphis/8/1988(H3N2) | 1 | 1 | No extrapolation |
| M88 | Human | CY010762 | GenBank | A/Memphis/8/1988(H3N2) | 1 | 2 | No extrapolation |
| M88 | Human | CY010761 | GenBank | A/Memphis/8/1988(H3N2) | 1 | 3 | No extrapolation |
| M88 | Human | CY010756 | GenBank | A/Memphis/8/1988(H3N2) | 1 | 4 | No extrapolation |
| M88 | Human | CY010759 | GenBank | A/Memphis/8/1988(H3N2) | 1 | 5 | No extrapolation |
| M88 | Human | CY010758 | GenBank | A/Memphis/8/1988(H3N2) | 1 | 6 | No extrapolation |
| M88 | Human | CY010757 | GenBank | A/Memphis/8/1988(H3N2) | 1 | 7 | No extrapolation |
| M88 | Human | CY010760 | GenBank | A/Memphis/8/1988(H3N2) | 1 | 8 | No extrapolation |
|  |  |  |  |  |  |  |  |
| MEMP71 | Human | CY002503 | GenBank | A/Memphis/1/1971(H3N2) | 1 | 1 | No extrapolation |
| MEMP71 | Human | CY002502 | GenBank | A/Memphis/1/1971(H3N2) | 1 | 2 | No extrapolation |
| MEMP71 | Human | CY002501 | GenBank | A/Memphis/1/1971(H3N2) | 1 | 3 | No extrapolation |
| MEMP71 | Human | CY002496 | GenBank | A/Memphis/1/1971(H3N2) | 1 | 4 | No extrapolation |
| MEMP71 | Human | CY002499 | GenBank | A/Memphis/1/1971(H3N2) | 1 | 5 | No extrapolation |
| MEMP71 | Human | CY002498 | GenBank | A/Memphis/1/1971(H3N2) | 1 | 6 | No extrapolation |
| MEMP71 | Human | CY002497 | GenBank | A/Memphis/1/1971(H3N2) | 1 | 7 | No extrapolation |
| MEMP71 | Human | CY002500 | GenBank | A/Memphis/1/1971(H3N2) | 1 | 8 | No extrapolation |
|  |  |  |  |  |  |  |  |
| MEX4108v1 | Human | GQ162180 | GenBank | A/Mexico/4108/2009(H1N1) | 1 | 1 | No extrapolation |
| MEX4108v1 | Human | GQ339879 | GenBank | A/Mexico/4108/2009(H1N1) | 1 | 2 | No extrapolation |
| MEX4108v1 | Human | GQ149661 | GenBank | A/Mexico/4108/2009(H1N1) | 1 | 3 | No extrapolation |
| MEX4108v1 | Human | GQ149662 | GenBank | A/Mexico/4108/2009(H1N1) | 1 | 4 | No extrapolation |
| MEX4108v1 | Human | GQ149660 | GenBank | A/Mexico/4108/2009(H1N1) | 1 | 5 | No extrapolation |
| MEX4108v1 | Human | GQ149659 | GenBank | A/Mexico/4108/2009(H1N1) | 1 | 6 | No extrapolation |
| MEX4108v1 | Human | GQ149657 | GenBank | A/Mexico/4108/2009(H1N1) | 1 | 7 | No extrapolation |
| MEX4108v1 | Human | GQ149658 | GenBank | A/Mexico/4108/2009(H1N1) | 1 | 8 | No extrapolation |
|  |  |  |  |  |  |  |  |
| MEX4108v2 | Human | GQ379815 | GenBank | A/Mexico/4108/2009(H1N1) | 2 | 1 | No extrapolation |
| MEX4108v2 | Human | GQ149652 | GenBank | A/Mexico/4108/2009(H1N1) | 2 | 2 | No extrapolation |
| MEX4108v2 | Human | GQ149653 | GenBank | A/Mexico/4108/2009(H1N1) | 2 | 3 | No extrapolation |
| MEX4108v2 | Human | GQ149654 | GenBank | A/Mexico/4108/2009(H1N1) | 2 | 4 | No extrapolation |
| MEX4108v2 | Human | GQ149655 | GenBank | A/Mexico/4108/2009(H1N1) | 2 | 5 | No extrapolation |
| MEX4108v2 | Human | GQ149656 | GenBank | A/Mexico/4108/2009(H1N1) | 2 | 6 | No extrapolation |
| MEX4108v2 | Human | GQ162179 | GenBank | A/Mexico/4108/2009(H1N1) | 2 | 7 | No extrapolation |
| MEX4108v2 | Human | GQ379814 | GenBank | A/Mexico/4108/2009(H1N1) | 2 | 8 | No extrapolation |
|  |  |  |  |  |  |  |  |
| MEX4482 | Human | CY098505 | GenBank | A/Mexico/4482/2009(H1N1) | 1 | 1 | No extrapolation |
| MEX4482 | Human | GQ149675 | GenBank | A/Mexico/4482/2009(H1N1) | 1 | 2 | No extrapolation |
| MEX4482 | Human | GQ149676 | GenBank | A/Mexico/4482/2009(H1N1) | 1 | 3 | No extrapolation |
| MEX4482 | Human | GQ149677 | GenBank | A/Mexico/4482/2009(H1N1) | 1 | 4 | No extrapolation |
| MEX4482 | Human | GQ149678 | GenBank | A/Mexico/4482/2009(H1N1) | 1 | 5 | No extrapolation |
| MEX4482 | Human | GQ379820 | GenBank | A/Mexico/4482/2009(H1N1) | 1 | 6 | No extrapolation |
| MEX4482 | Human | GQ162192 | GenBank | A/Mexico/4482/2009(H1N1) | 1 | 7 | No extrapolation |
| MEX4482 | Human | GQ379818 | GenBank | A/Mexico/4482/2009(H1N1) | 1 | 8 | No extrapolation |
|  |  |  |  |  |  |  |  |
| MEX4487 | Human | FJ998206 | GenBank | A/Mexico/InDRE4487/2009(H1N1) | 1 | 1 | No extrapolation |
| MEX4487 | Human | FJ998226 | GenBank | A/Mexico/InDRE4487/2009(H1N1) | 1 | 2 | No extrapolation |
| MEX4487 | Human | FJ998223 | GenBank | A/Mexico/InDRE4487/2009(H1N1) | 1 | 3 | No extrapolation |
| MEX4487 | Human | FJ998208 | GenBank | A/Mexico/InDRE4487/2009(H1N1) | 1 | 4 | No extrapolation |
| MEX4487 | Human | FJ998217 | GenBank | A/Mexico/InDRE4487/2009(H1N1) | 1 | 5 | No extrapolation |
| MEX4487 | Human | FJ998214 | GenBank | A/Mexico/InDRE4487/2009(H1N1) | 1 | 6 | No extrapolation |
| MEX4487 | Human | FJ998211 | GenBank | A/Mexico/InDRE4487/2009(H1N1) | 1 | 7 | No extrapolation |
| MEX4487 | Human | FJ998220 | GenBank | A/Mexico/InDRE4487/2009(H1N1) | 1 | 8 | No extrapolation |
|  |  |  |  |  |  |  |  |
| MI63 | Equine | M29257 | GenBank | A/equine/2/Miami/1963(H3N8) | 1 | 4 | No extrapolation |
| MI63 | Equine | AF001674 | GenBank | A/equine/2/Miami/1963(H3N8) | 1 | 7 | No extrapolation |
| MI63 | Equine | KJ664226 | GenBank | A/equine/2/Miami/1963(H3N8) | 1 | 8 | No extrapolation |
|  |  |  |  |  |  |  |  |
| MN81 | Avian | CY014732 | GenBank | A/duck/Minnesota/1525/1981(H5N1) | 1 | 1 | No extrapolation |
| MN81 | Avian | CY014731 | GenBank | A/duck/Minnesota/1525/1981(H5N1) | 1 | 2 | No extrapolation |
| MN81 | Avian | CY014730 | GenBank | A/duck/Minnesota/1525/1981(H5N1) | 1 | 3 | No extrapolation |
| MN81 | Avian | CY014726 | GenBank | A/duck/Minnesota/1525/1981(H5N1) | 1 | 4 | No extrapolation |
| MN81 | Avian | CY014728 | GenBank | A/duck/Minnesota/1525/1981(H5N1) | 1 | 5 | No extrapolation |
| MN81 | Avian | CY179413 | GenBank | A/duck/Minnesota/1525/1981(H5N1) | 1 | 6 | Full segment |
| MN81 | Avian | CY014727 | GenBank | A/duck/Minnesota/1525/1981(H5N1) | 1 | 7 | No extrapolation |
| MN81 | Avian | CY014729 | GenBank | A/duck/Minnesota/1525/1981(H5N1) | 1 | 8 | No extrapolation |
|  |  |  |  |  |  |  |  |
| NAR1 | Human | GQ169307 | GenBank | A/Narita/1/2009(H1N1) | 1 | 1 | No extrapolation |
| NAR1 | Human | GQ169306 | GenBank | A/Narita/1/2009(H1N1) | 1 | 2 | No extrapolation |
| NAR1 | Human | GQ169305 | GenBank | A/Narita/1/2009(H1N1) | 1 | 3 | No extrapolation |
| NAR1 | Human | GQ165814 | GenBank | A/Narita/1/2009(H1N1) | 1 | 4 | No extrapolation |
| NAR1 | Human | GQ169303 | GenBank | A/Narita/1/2009(H1N1) | 1 | 5 | No extrapolation |
| NAR1 | Human | GQ166204 | GenBank | A/Narita/1/2009(H1N1) | 1 | 6 | No extrapolation |
| NAR1 | Human | GQ169302 | GenBank | A/Narita/1/2009(H1N1) | 1 | 7 | No extrapolation |
| NAR1 | Human | GQ169304 | GenBank | A/Narita/1/2009(H1N1) | 1 | 8 | No extrapolation |
|  |  |  |  |  |  |  |  |
| NC99v1 | Human | DQ508854 | GenBank | A/NewCaledonia/20/1999(H1N1) | 1 | 1 | No extrapolation |
| NC99v1 | Human | DQ508855 | GenBank | A/NewCaledonia/20/1999(H1N1) | 1 | 2 | No extrapolation |
| NC99v1 | Human | DQ508856 | GenBank | A/NewCaledonia/20/1999(H1N1) | 1 | 3 | No extrapolation |
| NC99v1 | Human | DQ508857 | GenBank | A/NewCaledonia/20/1999(H1N1) | 1 | 4 | No extrapolation |
| NC99v1 | Human | DQ508858 | GenBank | A/NewCaledonia/20/1999(H1N1) | 1 | 5 | No extrapolation |
| NC99v1 | Human | DQ508859 | GenBank | A/NewCaledonia/20/1999(H1N1) | 1 | 6 | No extrapolation |
| NC99v1 | Human | DQ508860 | GenBank | A/NewCaledonia/20/1999(H1N1) | 1 | 7 | No extrapolation |
| NC99v1 | Human | DQ508861 | GenBank | A/NewCaledonia/20/1999(H1N1) | 1 | 8 | No extrapolation |
|  |  |  |  |  |  |  |  |
| NC99v2 | Human | CY033629 | GenBank | A/NewCaledonia/20/1999(H1N1) | 2 | 1 | No extrapolation |
| NC99v2 | Human | CY033628 | GenBank | A/NewCaledonia/20/1999(H1N1) | 2 | 2 | No extrapolation |
| NC99v2 | Human | CY033627 | GenBank | A/NewCaledonia/20/1999(H1N1) | 2 | 3 | No extrapolation |
| NC99v2 | Human | CY033622 | GenBank | A/NewCaledonia/20/1999(H1N1) | 2 | 4 | No extrapolation |
| NC99v2 | Human | CY033625 | GenBank | A/NewCaledonia/20/1999(H1N1) | 2 | 5 | No extrapolation |
| NC99v2 | Human | CY033624 | GenBank | A/NewCaledonia/20/1999(H1N1) | 2 | 6 | No extrapolation |
| NC99v2 | Human | CY033623 | GenBank | A/NewCaledonia/20/1999(H1N1) | 2 | 7 | No extrapolation |
| NC99v2 | Human | CY033626 | GenBank | A/NewCaledonia/20/1999(H1N1) | 2 | 8 | No extrapolation |
|  |  |  |  |  |  |  |  |
| NC99v3 | Human | CY125107 | GenBank | A/NewCaledonia/20/1999(H1N1) | 2 | 1 | No extrapolation |
| NC99v3 | Human | CY125106 | GenBank | A/NewCaledonia/20/1999(H1N1) | 2 | 2 | No extrapolation |
| NC99v3 | Human | CY125105 | GenBank | A/NewCaledonia/20/1999(H1N1) | 2 | 3 | No extrapolation |
| NC99v3 | Human | CY125100 | GenBank | A/NewCaledonia/20/1999(H1N1) | 2 | 4 | No extrapolation |
| NC99v3 | Human | CY125103 | GenBank | A/NewCaledonia/20/1999(H1N1) | 2 | 5 | No extrapolation |
| NC99v3 | Human | CY125102 | GenBank | A/NewCaledonia/20/1999(H1N1) | 2 | 6 | No extrapolation |
| NC99v3 | Human | CY125101 | GenBank | A/NewCaledonia/20/1999(H1N1) | 2 | 7 | No extrapolation |
| NC99v3 | Human | CY125104 | GenBank | A/NewCaledonia/20/1999(H1N1) | 2 | 8 | No extrapolation |
|  |  |  |  |  |  |  |  |
| NJ76 | Human | CY130125 | GenBank | A/NewJersey/8/1976(H1N1) | 1 | 1 | No extrapolation |
| NJ76 | Human | CY130124 | GenBank | A/NewJersey/8/1976(H1N1) | 1 | 2 | No extrapolation |
| NJ76 | Human | CY130123 | GenBank | A/NewJersey/8/1976(H1N1) | 1 | 3 | No extrapolation |
| NJ76 | Human | CY130118 | GenBank | A/NewJersey/8/1976(H1N1) | 1 | 4 | No extrapolation |
| NJ76 | Human | CY130121 | GenBank | A/NewJersey/8/1976(H1N1) | 1 | 5 | No extrapolation |
| NJ76 | Human | CY130120 | GenBank | A/NewJersey/8/1976(H1N1) | 1 | 6 | No extrapolation |
| NJ76 | Human | CY130119 | GenBank | A/NewJersey/8/1976(H1N1) | 1 | 7 | No extrapolation |
| NJ76 | Human | CY130122 | GenBank | A/NewJersey/8/1976(H1N1) | 1 | 8 | No extrapolation |
|  |  |  |  |  |  |  |  |
| NL03 | Human | AY342413 | GenBank | A/Netherlands/219/2003(H7N7) | 1 | 1 | No extrapolation |
| NL03 | Human | AY340083 | GenBank | A/Netherlands/219/2003(H7N7) | 1 | 2 | No extrapolation |
| NL03 | Human | AY342418 | GenBank | A/Netherlands/219/2003(H7N7) | 1 | 3 | No extrapolation |
| NL03 | Human | AY338459 | GenBank | A/Netherlands/219/2003(H7N7) | 1 | 4 | No extrapolation |
| NL03 | Human | AY342425 | GenBank | A/Netherlands/219/2003(H7N7) | 1 | 5 | No extrapolation |
| NL03 | Human | AY340079 | GenBank | A/Netherlands/219/2003(H7N7) | 1 | 6 | No extrapolation |
| NL03 | Human | AY340089 | GenBank | A/Netherlands/219/2003(H7N7) | 1 | 7 | No extrapolation |
| NL03 | Human | AY342422 | GenBank | A/Netherlands/219/2003(H7N7) | 1 | 8 | No extrapolation |
|  |  |  |  |  |  |  |  |
| NL230 | Human | EPI319935 | GISAID | A/Netherlands/230/2003(H7N7) | 1 | 1 | Partial |
| NL230 | Human | AB438939 | GenBank | A/Netherlands/230/2003(H7N7) | 1 | 2 | Full segment |
| NL230 | Human | AB438940 | GenBank | A/Netherlands/230/2003(H7N7) | 1 | 3 | Full segment |
| NL230 | Human | EPI319937 | GISAID | A/Netherlands/230/2003(H7N7) | 1 | 4 | Partial |
| NL230 | Human | AB438942 | GenBank | A/Netherlands/230/2003(H7N7) | 1 | 5 | Full segment |
| NL230 | Human | EPI319936 | GISAID | A/Netherlands/230/2003(H7N7) | 1 | 6 | Partial |
| NL230 | Human | AB438944 | GenBank | A/Netherlands/230/2003(H7N7) | 1 | 7 | Full segment |
| NL230 | Human | AB438945 | GenBank | A/Netherlands/230/2003(H7N7) | 1 | 8 | Full segment |
|  |  |  |  |  |  |  |  |
| NL602 | Human | CY046940 | GenBank | A/Netherlands/602/2009(H1N1) | 1 | 1 | No extrapolation |
| NL602 | Human | CY046941 | GenBank | A/Netherlands/602/2009(H1N1) | 1 | 2 | No extrapolation |
| NL602 | Human | CY046942 | GenBank | A/Netherlands/602/2009(H1N1) | 1 | 3 | No extrapolation |
| NL602 | Human | CY039527 | GenBank | A/Netherlands/602/2009(H1N1) | 1 | 4 | No extrapolation |
| NL602 | Human | CY046943 | GenBank | A/Netherlands/602/2009(H1N1) | 1 | 5 | No extrapolation |
| NL602 | Human | CY039528 | GenBank | A/Netherlands/602/2009(H1N1) | 1 | 6 | No extrapolation |
| NL602 | Human | CY046944 | GenBank | A/Netherlands/602/2009(H1N1) | 1 | 7 | No extrapolation |
| NL602 | Human | CY046945 | GenBank | A/Netherlands/602/2009(H1N1) | 1 | 8 | No extrapolation |
|  |  |  |  |  |  |  |  |
| NL94 | Human | CY114164 | GenBank | A/Netherlands/18/1994(H3N2) | 1 | 1 | No extrapolation |
| NL94 | Human | CY114163 | GenBank | A/Netherlands/18/1994(H3N2) | 1 | 2 | No extrapolation |
| NL94 | Human | CY114162 | GenBank | A/Netherlands/18/1994(H3N2) | 1 | 3 | No extrapolation |
| NL94 | Human | CY114157 | GenBank | A/Netherlands/18/1994(H3N2) | 1 | 4 | No extrapolation |
| NL94 | Human | CY114160 | GenBank | A/Netherlands/18/1994(H3N2) | 1 | 5 | No extrapolation |
| NL94 | Human | CY114159 | GenBank | A/Netherlands/18/1994(H3N2) | 1 | 6 | No extrapolation |
| NL94 | Human | CY114158 | GenBank | A/Netherlands/18/1994(H3N2) | 1 | 7 | No extrapolation |
| NL94 | Human | CY114161 | GenBank | A/Netherlands/18/1994(H3N2) | 1 | 8 | No extrapolation |
|  |  |  |  |  |  |  |  |
| NWS33 | Human | CY120991 | GenBank | A/NWS/1933(H1N1) | 1 | 1 | Full segment |
| NWS33 | Human | CY120990 | GenBank | A/NWS/1933(H1N1) | 1 | 2 | Full segment |
| NWS33 | Human | CY120989 | GenBank | A/NWS/1933(H1N1) | 1 | 3 | Full segment |
| NWS33 | Human | U08903 | GenBank | A/NWS/1933(H1N1) | 1 | 4 | No extrapolation |
| NWS33 | Human | CY120987 | GenBank | A/NWS/1933(H1N1) | 1 | 5 | Full segment |
| NWS33 | Human | L25815 | GenBank | A/NWS/1933(H1N1) | 1 | 6 | No extrapolation |
| NWS33 | Human | L25814 | GenBank | A/NWS/1933(H1N1) | 1 | 7 | No extrapolation |
| NWS33 | Human | L25720 | GenBank | A/NWS/1933(H1N1) | 1 | 8 | No extrapolation |
|  |  |  |  |  |  |  |  |
| NX488-53 | Avian | MF399665 | GenBank | A/NorthernShoveler/Ningxia/488-53/2015(H5N6) | 1 | 1 | No extrapolation |
| NX488-53 | Avian | MF399666 | GenBank | A/NorthernShoveler/Ningxia/488-53/2015(H5N6) | 1 | 2 | No extrapolation |
| NX488-53 | Avian | MF399667 | GenBank | A/NorthernShoveler/Ningxia/488-53/2015(H5N6) | 1 | 3 | No extrapolation |
| NX488-53 | Avian | MF399668 | GenBank | A/NorthernShoveler/Ningxia/488-53/2015(H5N6) | 1 | 4 | No extrapolation |
| NX488-53 | Avian | MF399669 | GenBank | A/NorthernShoveler/Ningxia/488-53/2015(H5N6) | 1 | 5 | No extrapolation |
| NX488-53 | Avian | MF399670 | GenBank | A/NorthernShoveler/Ningxia/488-53/2015(H5N6) | 1 | 6 | No extrapolation |
| NX488-53 | Avian | MF399671 | GenBank | A/NorthernShoveler/Ningxia/488-53/2015(H5N6) | 1 | 7 | No extrapolation |
| NX488-53 | Avian | MF399672 | GenBank | A/NorthernShoveler/Ningxia/488-53/2015(H5N6) | 1 | 8 | No extrapolation |
|  |  |  |  |  |  |  |  |
| NY107 | Human | EU783920 | GenBank | A/NewYork/107/2003(H7N2) | 1 | 1 | No extrapolation |
| NY107 | Human | EU587369 | GenBank | A/NewYork/107/2003(H7N2) | 1 | 2 | No extrapolation |
| NY107 | Human | EU587370 | GenBank | A/NewYork/107/2003(H7N2) | 1 | 3 | No extrapolation |
| NY107 | Human | EU587368 | GenBank | A/NewYork/107/2003(H7N2) | 1 | 4 | No extrapolation |
| NY107 | Human | EU587371 | GenBank | A/NewYork/107/2003(H7N2) | 1 | 5 | Partial |
| NY107 | Human | EU587372 | GenBank | A/NewYork/107/2003(H7N2) | 1 | 6 | No extrapolation |
| NY107 | Human | EU587373 | GenBank | A/NewYork/107/2003(H7N2) | 1 | 7 | No extrapolation |
| NY107 | Human | EU587374 | GenBank | A/NewYork/107/2003(H7N2) | 1 | 8 | No extrapolation |
|  |  |  |  |  |  |  |  |
| NY1682 | Human | CY039908 | GenBank | A/NewYork/1682/2009(H1N1) | 1 | 1 | No extrapolation |
| NY1682 | Human | CY039907 | GenBank | A/NewYork/1682/2009(H1N1) | 1 | 2 | No extrapolation |
| NY1682 | Human | CY039906 | GenBank | A/NewYork/1682/2009(H1N1) | 1 | 3 | No extrapolation |
| NY1682 | Human | CY039901 | GenBank | A/NewYork/1682/2009(H1N1) | 1 | 4 | No extrapolation |
| NY1682 | Human | CY039904 | GenBank | A/NewYork/1682/2009(H1N1) | 1 | 5 | No extrapolation |
| NY1682 | Human | CY039903 | GenBank | A/NewYork/1682/2009(H1N1) | 1 | 6 | No extrapolation |
| NY1682 | Human | CY039902 | GenBank | A/NewYork/1682/2009(H1N1) | 1 | 7 | No extrapolation |
| NY1682 | Human | CY039905 | GenBank | A/NewYork/1682/2009(H1N1) | 1 | 8 | No extrapolation |
|  |  |  |  |  |  |  |  |
| NY18 | Human | FJ984351 | GenBank | A/NewYork/18/2009(H1N1) | 1 | 1 | No extrapolation |
| NY18 | Human | FJ984353 | GenBank | A/NewYork/18/2009(H1N1) | 1 | 2 | No extrapolation |
| NY18 | Human | FJ984354 | GenBank | A/NewYork/18/2009(H1N1) | 1 | 3 | No extrapolation |
| NY18 | Human | FJ984355 | GenBank | A/NewYork/18/2009(H1N1) | 1 | 4 | No extrapolation |
| NY18 | Human | FJ984352 | GenBank | A/NewYork/18/2009(H1N1) | 1 | 5 | No extrapolation |
| NY18 | Human | FJ984350 | GenBank | A/NewYork/18/2009(H1N1) | 1 | 6 | No extrapolation |
| NY18 | Human | FJ984348 | GenBank | A/NewYork/18/2009(H1N1) | 1 | 7 | No extrapolation |
| NY18 | Human | FJ984349 | GenBank | A/NewYork/18/2009(H1N1) | 1 | 8 | No extrapolation |
|  |  |  |  |  |  |  |  |
| NY312 | Human | CY002703 | GenBank | A/NewYork/312/2001(H1N1) | 1 | 1 | No extrapolation |
| NY312 | Human | CY002702 | GenBank | A/NewYork/312/2001(H1N1) | 1 | 2 | No extrapolation |
| NY312 | Human | CY002701 | GenBank | A/NewYork/312/2001(H1N1) | 1 | 3 | No extrapolation |
| NY312 | Human | CY002696 | GenBank | A/NewYork/312/2001(H1N1) | 1 | 4 | No extrapolation |
| NY312 | Human | CY002699 | GenBank | A/NewYork/312/2001(H1N1) | 1 | 5 | No extrapolation |
| NY312 | Human | CY002698 | GenBank | A/NewYork/312/2001(H1N1) | 1 | 6 | No extrapolation |
| NY312 | Human | CY002697 | GenBank | A/NewYork/312/2001(H1N1) | 1 | 7 | No extrapolation |
| NY312 | Human | CY002700 | GenBank | A/NewYork/312/2001(H1N1) | 1 | 8 | No extrapolation |
|  |  |  |  |  |  |  |  |
| OH02 | Human | EPI291908 | GISAID | A/Ohio/02/2007(H1N1) | 1 | 1 | Partial |
| OH02 | Human | EPI291907 | GISAID | A/Ohio/02/2007(H1N1) | 1 | 2 | No extrapolation |
| OH02 | Human | EPI291906 | GISAID | A/Ohio/02/2007(H1N1) | 1 | 3 | No extrapolation |
| OH02 | Human | EPI178949 | GISAID | A/Ohio/02/2007(H1N1) | 1 | 4 | No extrapolation |
| OH02 | Human | EPI291905 | GISAID | A/Ohio/02/2007(H1N1) | 1 | 5 | No extrapolation |
| OH02 | Human | EPI338828 | GISAID | A/Ohio/02/2007(H1N1) | 1 | 6 | Partial |
| OH02 | Human | EPI291904 | GISAID | A/Ohio/02/2007(H1N1) | 1 | 7 | No extrapolation |
| OH02 | Human | EPI291903 | GISAID | A/Ohio/02/2007(H1N1) | 1 | 8 | No extrapolation |
|  |  |  |  |  |  |  |  |
| OH175 | Avian | CY018884 | GenBank | A/green-wingedteal/Ohio/175/1986(H2N1) | 1 | 1 | No extrapolation |
| OH175 | Avian | CY018883 | GenBank | A/green-wingedteal/Ohio/175/1986(H2N1) | 1 | 2 | No extrapolation |
| OH175 | Avian | CY018882 | GenBank | A/green-wingedteal/Ohio/175/1986(H2N1) | 1 | 3 | No extrapolation |
| OH175 | Avian | CY018877 | GenBank | A/green-wingedteal/Ohio/175/1986(H2N1) | 1 | 4 | No extrapolation |
| OH175 | Avian | CY018880 | GenBank | A/green-wingedteal/Ohio/175/1986(H2N1) | 1 | 5 | No extrapolation |
| OH175 | Avian | CY018879 | GenBank | A/green-wingedteal/Ohio/175/1986(H2N1) | 1 | 6 | No extrapolation |
| OH175 | Avian | CY018878 | GenBank | A/green-wingedteal/Ohio/175/1986(H2N1) | 1 | 7 | No extrapolation |
| OH175 | Avian | CY018881 | GenBank | A/green-wingedteal/Ohio/175/1986(H2N1) | 1 | 8 | No extrapolation |
|  |  |  |  |  |  |  |  |
| OH265 | Avian | CY017282 | GenBank | A/mallard/Ohio/265/1987(H1N9) | 1 | 1 | No extrapolation |
| OH265 | Avian | CY017281 | GenBank | A/mallard/Ohio/265/1987(H1N9) | 1 | 2 | No extrapolation |
| OH265 | Avian | CY017280 | GenBank | A/mallard/Ohio/265/1987(H1N9) | 1 | 3 | No extrapolation |
| OH265 | Avian | CY017275 | GenBank | A/mallard/Ohio/265/1987(H1N9) | 1 | 4 | No extrapolation |
| OH265 | Avian | CY017278 | GenBank | A/mallard/Ohio/265/1987(H1N9) | 1 | 5 | No extrapolation |
| OH265 | Avian | CY017277 | GenBank | A/mallard/Ohio/265/1987(H1N9) | 1 | 6 | No extrapolation |
| OH265 | Avian | CY017276 | GenBank | A/mallard/Ohio/265/1987(H1N9) | 1 | 7 | No extrapolation |
| OH265 | Avian | CY017279 | GenBank | A/mallard/Ohio/265/1987(H1N9) | 1 | 8 | No extrapolation |
|  |  |  |  |  |  |  |  |
| PAN99v1 | Human | DQ508862 | GenBank | A/Panama/2007/1999(H3N2) | 1 | 1 | No extrapolation |
| PAN99v1 | Human | DQ508863 | GenBank | A/Panama/2007/1999(H3N2) | 1 | 2 | No extrapolation |
| PAN99v1 | Human | DQ508864 | GenBank | A/Panama/2007/1999(H3N2) | 1 | 3 | No extrapolation |
| PAN99v1 | Human | DQ508865 | GenBank | A/Panama/2007/1999(H3N2) | 1 | 4 | No extrapolation |
| PAN99v1 | Human | DQ508866 | GenBank | A/Panama/2007/1999(H3N2) | 1 | 5 | No extrapolation |
| PAN99v1 | Human | DQ508867 | GenBank | A/Panama/2007/1999(H3N2) | 1 | 6 | No extrapolation |
| PAN99v1 | Human | DQ508868 | GenBank | A/Panama/2007/1999(H3N2) | 1 | 7 | No extrapolation |
| PAN99v1 | Human | DQ508869 | GenBank | A/Panama/2007/1999(H3N2) | 1 | 8 | No extrapolation |
|  |  |  |  |  |  |  |  |
| PAN99v2 | Human | DQ487334 | GenBank | A/Panama/2007/1999(H3N2) | 2 | 1 | No extrapolation |
| PAN99v2 | Human | DQ487333 | GenBank | A/Panama/2007/1999(H3N2) | 2 | 2 | No extrapolation |
| PAN99v2 | Human | DQ487335 | GenBank | A/Panama/2007/1999(H3N2) | 2 | 3 | No extrapolation |
| PAN99v2 | Human | DQ487340 | GenBank | A/Panama/2007/1999(H3N2) | 2 | 4 | No extrapolation |
| PAN99v2 | Human | DQ487339 | GenBank | A/Panama/2007/1999(H3N2) | 2 | 5 | No extrapolation |
| PAN99v2 | Human | DQ487337 | GenBank | A/Panama/2007/1999(H3N2) | 2 | 6 | No extrapolation |
| PAN99v2 | Human | DQ487338 | GenBank | A/Panama/2007/1999(H3N2) | 2 | 7 | No extrapolation |
| PAN99v2 | Human | DQ487336 | GenBank | A/Panama/2007/1999(H3N2) | 2 | 8 | No extrapolation |
|  |  |  |  |  |  |  |  |
| PAN99v3 | Human | CY034107 | GenBank | A/Panama/2007/1999(H3N2) | 3 | 1 | No extrapolation |
| PAN99v3 | Human | CY034106 | GenBank | A/Panama/2007/1999(H3N2) | 3 | 2 | No extrapolation |
| PAN99v3 | Human | CY034105 | GenBank | A/Panama/2007/1999(H3N2) | 3 | 3 | No extrapolation |
| PAN99v3 | Human | CY034100 | GenBank | A/Panama/2007/1999(H3N2) | 3 | 4 | No extrapolation |
| PAN99v3 | Human | CY034103 | GenBank | A/Panama/2007/1999(H3N2) | 3 | 5 | No extrapolation |
| PAN99v3 | Human | CY034102 | GenBank | A/Panama/2007/1999(H3N2) | 3 | 6 | No extrapolation |
| PAN99v3 | Human | CY034101 | GenBank | A/Panama/2007/1999(H3N2) | 3 | 7 | No extrapolation |
| PAN99v3 | Human | CY034104 | GenBank | A/Panama/2007/1999(H3N2) | 3 | 8 | No extrapolation |
|  |  |  |  |  |  |  |  |
| PAN99v4 | Human | CY112924 | GenBank | A/Panama/2007/1999(H3N2) | 4 | 1 | No extrapolation |
| PAN99v4 | Human | CY112923 | GenBank | A/Panama/2007/1999(H3N2) | 4 | 2 | No extrapolation |
| PAN99v4 | Human | CY112922 | GenBank | A/Panama/2007/1999(H3N2) | 4 | 3 | No extrapolation |
| PAN99v4 | Human | CY112917 | GenBank | A/Panama/2007/1999(H3N2) | 4 | 4 | No extrapolation |
| PAN99v4 | Human | CY112920 | GenBank | A/Panama/2007/1999(H3N2) | 4 | 5 | No extrapolation |
| PAN99v4 | Human | CY112919 | GenBank | A/Panama/2007/1999(H3N2) | 4 | 6 | No extrapolation |
| PAN99v4 | Human | CY112918 | GenBank | A/Panama/2007/1999(H3N2) | 4 | 7 | No extrapolation |
| PAN99v4 | Human | CY112921 | GenBank | A/Panama/2007/1999(H3N2) | 4 | 8 | No extrapolation |
|  |  |  |  |  |  |  |  |
| PHIL82v1 | Human | CY065975 | GenBank | A/Philippines/2/1982(H3N2) | 1 | 1 | No extrapolation |
| PHIL82v1 | Human | CY065974 | GenBank | A/Philippines/2/1982(H3N2) | 1 | 2 | No extrapolation |
| PHIL82v1 | Human | CY065973 | GenBank | A/Philippines/2/1982(H3N2) | 1 | 3 | No extrapolation |
| PHIL82v1 | Human | CY065968 | GenBank | A/Philippines/2/1982(H3N2) | 1 | 4 | No extrapolation |
| PHIL82v1 | Human | CY065971 | GenBank | A/Philippines/2/1982(H3N2) | 1 | 5 | No extrapolation |
| PHIL82v1 | Human | CY065970 | GenBank | A/Philippines/2/1982(H3N2) | 1 | 6 | No extrapolation |
| PHIL82v1 | Human | CY065969 | GenBank | A/Philippines/2/1982(H3N2) | 1 | 7 | No extrapolation |
| PHIL82v1 | Human | CY065972 | GenBank | A/Philippines/2/1982(H3N2) | 1 | 8 | No extrapolation |
|  |  |  |  |  |  |  |  |
| PHIL82v2 | Human | CY121252 | GenBank | A/Philippines/2/1982(H3N2) | 2 | 1 | No extrapolation |
| PHIL82v2 | Human | CY121251 | GenBank | A/Philippines/2/1982(H3N2) | 2 | 2 | No extrapolation |
| PHIL82v2 | Human | CY121250 | GenBank | A/Philippines/2/1982(H3N2) | 2 | 3 | No extrapolation |
| PHIL82v2 | Human | CY121245 | GenBank | A/Philippines/2/1982(H3N2) | 2 | 4 | No extrapolation |
| PHIL82v2 | Human | CY121248 | GenBank | A/Philippines/2/1982(H3N2) | 2 | 5 | No extrapolation |
| PHIL82v2 | Human | CY121247 | GenBank | A/Philippines/2/1982(H3N2) | 2 | 6 | No extrapolation |
| PHIL82v2 | Human | CY121246 | GenBank | A/Philippines/2/1982(H3N2) | 2 | 7 | No extrapolation |
| PHIL82v2 | Human | CY121249 | GenBank | A/Philippines/2/1982(H3N2) | 2 | 8 | No extrapolation |
|  |  |  |  |  |  |  |  |
| PHIL82v3 | Human | CY113308 | GenBank | A/Philippines/2/1982(H3N2) | 3 | 1 | No extrapolation |
| PHIL82v3 | Human | CY113307 | GenBank | A/Philippines/2/1982(H3N2) | 3 | 2 | No extrapolation |
| PHIL82v3 | Human | CY113306 | GenBank | A/Philippines/2/1982(H3N2) | 3 | 3 | No extrapolation |
| PHIL82v3 | Human | CY113301 | GenBank | A/Philippines/2/1982(H3N2) | 3 | 4 | No extrapolation |
| PHIL82v3 | Human | CY113304 | GenBank | A/Philippines/2/1982(H3N2) | 3 | 5 | No extrapolation |
| PHIL82v3 | Human | CY113303 | GenBank | A/Philippines/2/1982(H3N2) | 3 | 6 | No extrapolation |
| PHIL82v3 | Human | CY113302 | GenBank | A/Philippines/2/1982(H3N2) | 3 | 7 | No extrapolation |
| PHIL82v3 | Human | CY113305 | GenBank | A/Philippines/2/1982(H3N2) | 3 | 8 | No extrapolation |
|  |  |  |  |  |  |  |  |
| PR8F | Mouse | ID0081 | Corresponding author (1, 2) | A/PR8F/1934(H1N1) | 1 | 1 | No extrapolation |
| PR8F | Mouse | ID0082 | Corresponding author (1, 2) | A/PR8F/1934(H1N1) | 1 | 2 | No extrapolation |
| PR8F | Mouse | ID0083 | Corresponding author (1, 2) | A/PR8F/1934(H1N1) | 1 | 3 | No extrapolation |
| PR8F | Mouse | ID0084 | Corresponding author (1, 2) | A/PR8F/1934(H1N1) | 1 | 4 | No extrapolation |
| PR8F | Mouse | ID0085 | Corresponding author (1, 2) | A/PR8F/1934(H1N1) | 1 | 5 | No extrapolation |
| PR8F | Mouse | ID0086 | Corresponding author (1, 2) | A/PR8F/1934(H1N1) | 1 | 6 | No extrapolation |
| PR8F | Mouse | ID0087 | Corresponding author (1, 2) | A/PR8F/1934(H1N1) | 1 | 7 | No extrapolation |
| PR8F | Mouse | ID0088 | Corresponding author (1, 2) | A/PR8F/1934(H1N1) | 1 | 8 | No extrapolation |
|  |  |  |  |  |  |  |  |
| PR8M | Mouse | ID0089 | Corresponding author (1, 2) | A/PR8M/1934(H1N1) | 1 | 1 | No extrapolation |
| PR8M | Mouse | ID0090 | Corresponding author (1, 2) | A/PR8M/1934(H1N1) | 1 | 2 | No extrapolation |
| PR8M | Mouse | ID0091 | Corresponding author (1, 2) | A/PR8M/1934(H1N1) | 1 | 3 | No extrapolation |
| PR8M | Mouse | ID0092 | Corresponding author (1, 2) | A/PR8M/1934(H1N1) | 1 | 4 | No extrapolation |
| PR8M | Mouse | ID0093 | Corresponding author (1, 2) | A/PR8M/1934(H1N1) | 1 | 5 | No extrapolation |
| PR8M | Mouse | ID0094 | Corresponding author (1, 2) | A/PR8M/1934(H1N1) | 1 | 6 | No extrapolation |
| PR8M | Mouse | ID0095 | Corresponding author (1, 2) | A/PR8M/1934(H1N1) | 1 | 7 | No extrapolation |
| PR8M | Mouse | ID0096 | Corresponding author (1, 2) | A/PR8M/1934(H1N1) | 1 | 8 | No extrapolation |
|  |  |  |  |  |  |  |  |
| PR8v1 | Human | V00603 | GenBank | A/PuertoRico/8/1934(H1N1) | 1 | 1 | No extrapolation |
| PR8v1 | Human | J02151 | GenBank | A/PuertoRico/8/1934(H1N1) | 1 | 2 | No extrapolation |
| PR8v1 | Human | V01106 | GenBank | A/PuertoRico/8/1934(H1N1) | 1 | 3 | No extrapolation |
| PR8v1 | Human | V01088 | GenBank | A/PuertoRico/8/1934(H1N1) | 1 | 4 | No extrapolation |
| PR8v1 | Human | J02147 | GenBank | A/PuertoRico/8/1934(H1N1) | 1 | 5 | No extrapolation |
| PR8v1 | Human | J02146 | GenBank | A/PuertoRico/8/1934(H1N1) | 1 | 6 | No extrapolation |
| PR8v1 | Human | V01099 | GenBank | A/PuertoRico/8/1934(H1N1) | 1 | 7 | No extrapolation |
| PR8v1 | Human | J02150 | GenBank | A/PuertoRico/8/1934(H1N1) | 1 | 8 | No extrapolation |
|  |  |  |  |  |  |  |  |
| PR8v2 | Human | EF467818 | GenBank | A/PuertoRico/8/1934(H1N1) | 2 | 1 | No extrapolation |
| PR8v2 | Human | EF467819 | GenBank | A/PuertoRico/8/1934(H1N1) | 2 | 2 | No extrapolation |
| PR8v2 | Human | EF467820 | GenBank | A/PuertoRico/8/1934(H1N1) | 2 | 3 | No extrapolation |
| PR8v2 | Human | EF467821 | GenBank | A/PuertoRico/8/1934(H1N1) | 2 | 4 | No extrapolation |
| PR8v2 | Human | EF467822 | GenBank | A/PuertoRico/8/1934(H1N1) | 2 | 5 | No extrapolation |
| PR8v2 | Human | EF467823 | GenBank | A/PuertoRico/8/1934(H1N1) | 2 | 6 | No extrapolation |
| PR8v2 | Human | EF467824 | GenBank | A/PuertoRico/8/1934(H1N1) | 2 | 7 | No extrapolation |
| PR8v2 | Human | EF467817 | GenBank | A/PuertoRico/8/1934(H1N1) | 2 | 8 | No extrapolation |
|  |  |  |  |  |  |  |  |
| PR8v3 | Human | LC120388 | GenBank | A/PuertoRico/8/1934(H1N1) | 3 | 1 | No extrapolation |
| PR8v3 | Human | LC120389 | GenBank | A/PuertoRico/8/1934(H1N1) | 3 | 2 | No extrapolation |
| PR8v3 | Human | LC120390 | GenBank | A/PuertoRico/8/1934(H1N1) | 3 | 3 | No extrapolation |
| PR8v3 | Human | LC120391 | GenBank | A/PuertoRico/8/1934(H1N1) | 3 | 4 | No extrapolation |
| PR8v3 | Human | LC120392 | GenBank | A/PuertoRico/8/1934(H1N1) | 3 | 5 | No extrapolation |
| PR8v3 | Human | LC120393 | GenBank | A/PuertoRico/8/1934(H1N1) | 3 | 6 | No extrapolation |
| PR8v3 | Human | LC120394 | GenBank | A/PuertoRico/8/1934(H1N1) | 3 | 7 | No extrapolation |
| PR8v3 | Human | LC120395 | GenBank | A/PuertoRico/8/1934(H1N1) | 3 | 8 | No extrapolation |
|  |  |  |  |  |  |  |  |
| PR8vMountSinai | Human | AF389115 | GenBank | A/PuertoRico/8/1934/MountSinai(H1N1) | 1 | 1 | No extrapolation |
| PR8vMountSinai | Human | AF389116 | GenBank | A/PuertoRico/8/1934/MountSinai(H1N1) | 1 | 2 | No extrapolation |
| PR8vMountSinai | Human | AF389117 | GenBank | A/PuertoRico/8/1934/MountSinai(H1N1) | 1 | 3 | No extrapolation |
| PR8vMountSinai | Human | AF389118 | GenBank | A/PuertoRico/8/1934/MountSinai(H1N1) | 1 | 4 | No extrapolation |
| PR8vMountSinai | Human | AF389119 | GenBank | A/PuertoRico/8/1934/MountSinai(H1N1) | 1 | 5 | No extrapolation |
| PR8vMountSinai | Human | AF389120 | GenBank | A/PuertoRico/8/1934/MountSinai(H1N1) | 1 | 6 | No extrapolation |
| PR8vMountSinai | Human | AF389121 | GenBank | A/PuertoRico/8/1934/MountSinai(H1N1) | 1 | 7 | No extrapolation |
| PR8vMountSinai | Human | AF389122 | GenBank | A/PuertoRico/8/1934/MountSinai(H1N1) | 1 | 8 | No extrapolation |
|  |  |  |  |  |  |  |  |
| QHKG1 | Quail | KY785900 | GenBank | A/quail/HongKong/G1/1997(H9N2) | 1 | 1 | No extrapolation |
| QHKG1 | Quail | KY785901 | GenBank | A/quail/HongKong/G1/1997(H9N2) | 1 | 2 | No extrapolation |
| QHKG1 | Quail | KY785899 | GenBank | A/quail/HongKong/G1/1997(H9N2) | 1 | 3 | No extrapolation |
| QHKG1 | Quail | KY785896 | GenBank | A/quail/HongKong/G1/1997(H9N2) | 1 | 4 | No extrapolation |
| QHKG1 | Quail | KY785898 | GenBank | A/quail/HongKong/G1/1997(H9N2) | 1 | 5 | No extrapolation |
| QHKG1 | Quail | KY785903 | GenBank | A/quail/HongKong/G1/1997(H9N2) | 1 | 6 | No extrapolation |
| QHKG1 | Quail | KY785902 | GenBank | A/quail/HongKong/G1/1997(H9N2) | 1 | 7 | No extrapolation |
| QHKG1 | Quail | KY785897 | GenBank | A/quail/HongKong/G1/1997(H9N2) | 1 | 8 | No extrapolation |
|  |  |  |  |  |  |  |  |
| RT625 | Avian | CY144420 | GenBank | A/ruddyturnstone/Delaware/650625/2002(H6N1) | 1 | 1 | No extrapolation |
| RT625 | Avian | CY144419 | GenBank | A/ruddyturnstone/Delaware/650625/2002(H6N1) | 1 | 2 | No extrapolation |
| RT625 | Avian | CY144418 | GenBank | A/ruddyturnstone/Delaware/650625/2002(H6N1) | 1 | 3 | No extrapolation |
| RT625 | Avian | CY144413 | GenBank | A/ruddyturnstone/Delaware/650625/2002(H6N1) | 1 | 4 | No extrapolation |
| RT625 | Avian | CY144416 | GenBank | A/ruddyturnstone/Delaware/650625/2002(H6N1) | 1 | 5 | No extrapolation |
| RT625 | Avian | CY144415 | GenBank | A/ruddyturnstone/Delaware/650625/2002(H6N1) | 1 | 6 | No extrapolation |
| RT625 | Avian | CY144414 | GenBank | A/ruddyturnstone/Delaware/650625/2002(H6N1) | 1 | 7 | No extrapolation |
| RT625 | Avian | CY144417 | GenBank | A/ruddyturnstone/Delaware/650625/2002(H6N1) | 1 | 8 | No extrapolation |
|  |  |  |  |  |  |  |  |
| S0322 | Avian | KP732584 | GenBank | A/duck/EasternChina/S0322/2014(H5N6) | 1 | 1 | No extrapolation |
| S0322 | Avian | KP732604 | GenBank | A/duck/EasternChina/S0322/2014(H5N6) | 1 | 2 | No extrapolation |
| S0322 | Avian | KP732624 | GenBank | A/duck/EasternChina/S0322/2014(H5N6) | 1 | 3 | No extrapolation |
| S0322 | Avian | KP732644 | GenBank | A/duck/EasternChina/S0322/2014(H5N6) | 1 | 4 | No extrapolation |
| S0322 | Avian | KP732664 | GenBank | A/duck/EasternChina/S0322/2014(H5N6) | 1 | 5 | No extrapolation |
| S0322 | Avian | KP732684 | GenBank | A/duck/EasternChina/S0322/2014(H5N6) | 1 | 6 | No extrapolation |
| S0322 | Avian | KP732704 | GenBank | A/duck/EasternChina/S0322/2014(H5N6) | 1 | 7 | No extrapolation |
| S0322 | Avian | KP732724 | GenBank | A/duck/EasternChina/S0322/2014(H5N6) | 1 | 8 | No extrapolation |
|  |  |  |  |  |  |  |  |
| S0513 | Avian | KP732578 | GenBank | A/goose/EasternChina/S0513/2013(H5N6) | 1 | 1 | No extrapolation |
| S0513 | Avian | KP732598 | GenBank | A/goose/EasternChina/S0513/2013(H5N6) | 1 | 2 | No extrapolation |
| S0513 | Avian | KP732618 | GenBank | A/goose/EasternChina/S0513/2013(H5N6) | 1 | 3 | No extrapolation |
| S0513 | Avian | KP732638 | GenBank | A/goose/EasternChina/S0513/2013(H5N6) | 1 | 4 | No extrapolation |
| S0513 | Avian | KP732658 | GenBank | A/goose/EasternChina/S0513/2013(H5N6) | 1 | 5 | No extrapolation |
| S0513 | Avian | KP732679 | GenBank | A/goose/EasternChina/S0513/2013(H5N6) | 1 | 6 | No extrapolation |
| S0513 | Avian | KP732698 | GenBank | A/goose/EasternChina/S0513/2013(H5N6) | 1 | 7 | No extrapolation |
| S0513 | Avian | KP732718 | GenBank | A/goose/EasternChina/S0513/2013(H5N6) | 1 | 8 | No extrapolation |
|  |  |  |  |  |  |  |  |
| S0711 | Avian | KP732581 | GenBank | A/duck/EasternChina/S0711/2014(H5N6) | 1 | 1 | No extrapolation |
| S0711 | Avian | KP732601 | GenBank | A/duck/EasternChina/S0711/2014(H5N6) | 1 | 2 | No extrapolation |
| S0711 | Avian | KP732621 | GenBank | A/duck/EasternChina/S0711/2014(H5N6) | 1 | 3 | No extrapolation |
| S0711 | Avian | KP732641 | GenBank | A/duck/EasternChina/S0711/2014(H5N6) | 1 | 4 | No extrapolation |
| S0711 | Avian | KP732661 | GenBank | A/duck/EasternChina/S0711/2014(H5N6) | 1 | 5 | No extrapolation |
| S0711 | Avian | KP732681 | GenBank | A/duck/EasternChina/S0711/2014(H5N6) | 1 | 6 | No extrapolation |
| S0711 | Avian | KP732701 | GenBank | A/duck/EasternChina/S0711/2014(H5N6) | 1 | 7 | No extrapolation |
| S0711 | Avian | KP732721 | GenBank | A/duck/EasternChina/S0711/2014(H5N6) | 1 | 8 | No extrapolation |
|  |  |  |  |  |  |  |  |
| S0908 | Avian | KP732583 | GenBank | A/duck/EasternChina/S0908/2014(H5N6) | 1 | 1 | No extrapolation |
| S0908 | Avian | KP732603 | GenBank | A/duck/EasternChina/S0908/2014(H5N6) | 1 | 2 | No extrapolation |
| S0908 | Avian | KP732623 | GenBank | A/duck/EasternChina/S0908/2014(H5N6) | 1 | 3 | No extrapolation |
| S0908 | Avian | KP732643 | GenBank | A/duck/EasternChina/S0908/2014(H5N6) | 1 | 4 | No extrapolation |
| S0908 | Avian | KP732663 | GenBank | A/duck/EasternChina/S0908/2014(H5N6) | 1 | 5 | No extrapolation |
| S0908 | Avian | KP732683 | GenBank | A/duck/EasternChina/S0908/2014(H5N6) | 1 | 6 | No extrapolation |
| S0908 | Avian | KP732703 | GenBank | A/duck/EasternChina/S0908/2014(H5N6) | 1 | 7 | No extrapolation |
| S0908 | Avian | KP732723 | GenBank | A/duck/EasternChina/S0908/2014(H5N6) | 1 | 8 | No extrapolation |
|  |  |  |  |  |  |  |  |
| SC09 | Human | GQ166228 | GenBank | A/Sichuan/1/2009(H1N1) | 1 | 1 | No extrapolation |
| SC09 | Human | GQ166227 | GenBank | A/Sichuan/1/2009(H1N1) | 1 | 2 | No extrapolation |
| SC09 | Human | GQ166226 | GenBank | A/Sichuan/1/2009(H1N1) | 1 | 3 | No extrapolation |
| SC09 | Human | GQ166223 | GenBank | A/Sichuan/1/2009(H1N1) | 1 | 4 | No extrapolation |
| SC09 | Human | GQ166225 | GenBank | A/Sichuan/1/2009(H1N1) | 1 | 5 | No extrapolation |
| SC09 | Human | GQ166224 | GenBank | A/Sichuan/1/2009(H1N1) | 1 | 6 | No extrapolation |
| SC09 | Human | GQ166229 | GenBank | A/Sichuan/1/2009(H1N1) | 1 | 7 | No extrapolation |
| SC09 | Human | GQ166230 | GenBank | A/Sichuan/1/2009(H1N1) | 1 | 8 | No extrapolation |
|  |  |  |  |  |  |  |  |
| SC18 | Human | DQ208309 | GenBank | A/SouthCarolina/1/1918(H1N1) | 1 | 1 | Full segment |
| SC18 | Human | DQ208310 | GenBank | A/SouthCarolina/1/1918(H1N1) | 1 | 2 | Full segment |
| SC18 | Human | DQ208311 | GenBank | A/SouthCarolina/1/1918(H1N1) | 1 | 3 | Full segment |
| SC18 | Human | AF117241 | GenBank | A/SouthCarolina/1/1918(H1N1) | 1 | 4 | No extrapolation |
| SC18 | Human | AY744935 | GenBank | A/SouthCarolina/1/1918(H1N1) | 1 | 5 | Full segment |
| SC18 | Human | AF250356 | GenBank | A/SouthCarolina/1/1918(H1N1) | 1 | 6 | Full segment |
| SC18 | Human | AY130766 | GenBank | A/SouthCarolina/1/1918(H1N1) | 1 | 7 | Full segment |
| SC18 | Human | AF333238 | GenBank | A/SouthCarolina/1/1918(H1N1) | 1 | 8 | Full segment |
|  |  |  |  |  |  |  |  |
| SC35M | Seal | DQ266094 | GenBank | A/seal/Massachussetts/1-SC35M/1980(H7N7) | 1 | 1 | No extrapolation |
| SC35M | Seal | DQ266095 | GenBank | A/seal/Massachussetts/1-SC35M/1980(H7N7) | 1 | 2 | No extrapolation |
| SC35M | Seal | DQ266096 | GenBank | A/seal/Massachussetts/1-SC35M/1980(H7N7) | 1 | 3 | No extrapolation |
| SC35M | Seal | DQ266097 | GenBank | A/seal/Massachussetts/1-SC35M/1980(H7N7) | 1 | 4 | No extrapolation |
| SC35M | Seal | DQ266098 | GenBank | A/seal/Massachussetts/1-SC35M/1980(H7N7) | 1 | 5 | No extrapolation |
| SC35M | Seal | DQ266099 | GenBank | A/seal/Massachussetts/1-SC35M/1980(H7N7) | 1 | 6 | No extrapolation |
| SC35M | Seal | DQ266100 | GenBank | A/seal/Massachussetts/1-SC35M/1980(H7N7) | 1 | 7 | No extrapolation |
| SC35M | Seal | DQ266101 | GenBank | A/seal/Massachussetts/1-SC35M/1980(H7N7) | 1 | 8 | No extrapolation |
|  |  |  |  |  |  |  |  |
| SD093 | Avian | AY856861 | GenBank | A/duck/Shandong/093/2004(H5N1) | 1 | 1 | No extrapolation |
| SD093 | Avian | AY856862 | GenBank | A/duck/Shandong/093/2004(H5N1) | 1 | 2 | No extrapolation |
| SD093 | Avian | AY856863 | GenBank | A/duck/Shandong/093/2004(H5N1) | 1 | 3 | No extrapolation |
| SD093 | Avian | AY854190 | GenBank | A/duck/Shandong/093/2004(H5N1) | 1 | 4 | No extrapolation |
| SD093 | Avian | AY856864 | GenBank | A/duck/Shandong/093/2004(H5N1) | 1 | 5 | No extrapolation |
| SD093 | Avian | AY854191 | GenBank | A/duck/Shandong/093/2004(H5N1) | 1 | 6 | No extrapolation |
| SD093 | Avian | AY856865 | GenBank | A/duck/Shandong/093/2004(H5N1) | 1 | 7 | No extrapolation |
| SD093 | Avian | AY856866 | GenBank | A/duck/Shandong/093/2004(H5N1) | 1 | 8 | No extrapolation |
|  |  |  |  |  |  |  |  |
| SH-9 | Avian | KJ476654 | GenBank | A/mallard/Shanghai/SH-9/2013(H5N8) | 1 | 1 | No extrapolation |
| SH-9 | Avian | KJ476655 | GenBank | A/mallard/Shanghai/SH-9/2013(H5N8) | 1 | 2 | No extrapolation |
| SH-9 | Avian | KJ476656 | GenBank | A/mallard/Shanghai/SH-9/2013(H5N8) | 1 | 3 | No extrapolation |
| SH-9 | Avian | KJ476657 | GenBank | A/mallard/Shanghai/SH-9/2013(H5N8) | 1 | 4 | No extrapolation |
| SH-9 | Avian | KJ476658 | GenBank | A/mallard/Shanghai/SH-9/2013(H5N8) | 1 | 5 | No extrapolation |
| SH-9 | Avian | KJ476659 | GenBank | A/mallard/Shanghai/SH-9/2013(H5N8) | 1 | 6 | No extrapolation |
| SH-9 | Avian | KJ476660 | GenBank | A/mallard/Shanghai/SH-9/2013(H5N8) | 1 | 7 | No extrapolation |
| SH-9 | Avian | KJ476661 | GenBank | A/mallard/Shanghai/SH-9/2013(H5N8) | 1 | 8 | No extrapolation |
|  |  |  |  |  |  |  |  |
| SH02v1 | Human | KF021594 | GenBank | A/Shanghai/02/2013(H7N9) | 1 | 1 | No extrapolation |
| SH02v1 | Human | KF021595 | GenBank | A/Shanghai/02/2013(H7N9) | 1 | 2 | No extrapolation |
| SH02v1 | Human | KF021596 | GenBank | A/Shanghai/02/2013(H7N9) | 1 | 3 | No extrapolation |
| SH02v1 | Human | KF021597 | GenBank | A/Shanghai/02/2013(H7N9) | 1 | 4 | No extrapolation |
| SH02v1 | Human | KF021598 | GenBank | A/Shanghai/02/2013(H7N9) | 1 | 5 | No extrapolation |
| SH02v1 | Human | KF021599 | GenBank | A/Shanghai/02/2013(H7N9) | 1 | 6 | No extrapolation |
| SH02v1 | Human | KF021600 | GenBank | A/Shanghai/02/2013(H7N9) | 1 | 7 | No extrapolation |
| SH02v1 | Human | KF021601 | GenBank | A/Shanghai/02/2013(H7N9) | 1 | 8 | No extrapolation |
|  |  |  |  |  |  |  |  |
| SH02v2 | Human | NC026422 | GenBank | A/Shanghai/02/2013(H7N9) | 2 | 1 | No extrapolation |
| SH02v2 | Human | NC026423 | GenBank | A/Shanghai/02/2013(H7N9) | 2 | 2 | No extrapolation |
| SH02v2 | Human | NC026424 | GenBank | A/Shanghai/02/2013(H7N9) | 2 | 3 | No extrapolation |
| SH02v2 | Human | NC026425 | GenBank | A/Shanghai/02/2013(H7N9) | 2 | 4 | No extrapolation |
| SH02v2 | Human | NC026426 | GenBank | A/Shanghai/02/2013(H7N9) | 2 | 5 | No extrapolation |
| SH02v2 | Human | NC026429 | GenBank | A/Shanghai/02/2013(H7N9) | 2 | 6 | No extrapolation |
| SH02v2 | Human | NC026427 | GenBank | A/Shanghai/02/2013(H7N9) | 2 | 7 | No extrapolation |
| SH02v2 | Human | NC026428 | GenBank | A/Shanghai/02/2013(H7N9) | 2 | 8 | No extrapolation |
|  |  |  |  |  |  |  |  |
| SH1 | Human | EPI439488 | GISAID | A/Shanghai/1/2013(H7N9) | 1 | 1 | No extrapolation |
| SH1 | Human | EPI439489 | GISAID | A/Shanghai/1/2013(H7N9) | 1 | 2 | No extrapolation |
| SH1 | Human | EPI439490 | GISAID | A/Shanghai/1/2013(H7N9) | 1 | 3 | No extrapolation |
| SH1 | Human | EPI439486 | GISAID | A/Shanghai/1/2013(H7N9) | 1 | 4 | No extrapolation |
| SH1 | Human | EPI439491 | GISAID | A/Shanghai/1/2013(H7N9) | 1 | 5 | No extrapolation |
| SH1 | Human | EPI439487 | GISAID | A/Shanghai/1/2013(H7N9) | 1 | 6 | No extrapolation |
| SH1 | Human | EPI439493 | GISAID | A/Shanghai/1/2013(H7N9) | 1 | 7 | No extrapolation |
| SH1 | Human | EPI439494 | GISAID | A/Shanghai/1/2013(H7N9) | 1 | 8 | No extrapolation |
|  |  |  |  |  |  |  |  |
| SH1023 | Avian | JF795121 | GenBank | A/chicken/Shandong/lx1023/2007(H9N2) | 1 | 1 | No extrapolation |
| SH1023 | Avian | JF795122 | GenBank | A/chicken/Shandong/lx1023/2007(H9N2) | 1 | 2 | No extrapolation |
| SH1023 | Avian | JF795123 | GenBank | A/chicken/Shandong/lx1023/2007(H9N2) | 1 | 3 | No extrapolation |
| SH1023 | Avian | FJ190142 | GenBank | A/chicken/Shandong/lx1023/2007(H9N2) | 1 | 4 | No extrapolation |
| SH1023 | Avian | JF795124 | GenBank | A/chicken/Shandong/lx1023/2007(H9N2) | 1 | 5 | No extrapolation |
| SH1023 | Avian | EU939162 | GenBank | A/chicken/Shandong/lx1023/2007(H9N2) | 1 | 6 | Full segment |
| SH1023 | Avian | JF795125 | GenBank | A/chicken/Shandong/lx1023/2007(H9N2) | 1 | 7 | No extrapolation |
| SH1023 | Avian | JF795126 | GenBank | A/chicken/Shandong/lx1023/2007(H9N2) | 1 | 8 | No extrapolation |
|  |  |  |  |  |  |  |  |
| SH1053 | Avian | CY146953 | GenBank | A/chicken/Shanghai/S1053/2013(H7N9) | 1 | 1 | No extrapolation |
| SH1053 | Avian | CY146954 | GenBank | A/chicken/Shanghai/S1053/2013(H7N9) | 1 | 2 | No extrapolation |
| SH1053 | Avian | CY146955 | GenBank | A/chicken/Shanghai/S1053/2013(H7N9) | 1 | 3 | No extrapolation |
| SH1053 | Avian | CY146956 | GenBank | A/chicken/Shanghai/S1053/2013(H7N9) | 1 | 4 | No extrapolation |
| SH1053 | Avian | CY146957 | GenBank | A/chicken/Shanghai/S1053/2013(H7N9) | 1 | 5 | No extrapolation |
| SH1053 | Avian | CY146958 | GenBank | A/chicken/Shanghai/S1053/2013(H7N9) | 1 | 6 | No extrapolation |
| SH1053 | Avian | CY146959 | GenBank | A/chicken/Shanghai/S1053/2013(H7N9) | 1 | 7 | No extrapolation |
| SH1053 | Avian | CY146960 | GenBank | A/chicken/Shanghai/S1053/2013(H7N9) | 1 | 8 | No extrapolation |
|  |  |  |  |  |  |  |  |
| SH1069 | Avian | CY147169 | GenBank | A/pigeon/Shanghai/S1069/2013(H7N9) | 1 | 1 | No extrapolation |
| SH1069 | Avian | CY147170 | GenBank | A/pigeon/Shanghai/S1069/2013(H7N9) | 1 | 2 | No extrapolation |
| SH1069 | Avian | CY147171 | GenBank | A/pigeon/Shanghai/S1069/2013(H7N9) | 1 | 3 | No extrapolation |
| SH1069 | Avian | CY147172 | GenBank | A/pigeon/Shanghai/S1069/2013(H7N9) | 1 | 4 | No extrapolation |
| SH1069 | Avian | CY147173 | GenBank | A/pigeon/Shanghai/S1069/2013(H7N9) | 1 | 5 | No extrapolation |
| SH1069 | Avian | CY147174 | GenBank | A/pigeon/Shanghai/S1069/2013(H7N9) | 1 | 6 | No extrapolation |
| SH1069 | Avian | CY147175 | GenBank | A/pigeon/Shanghai/S1069/2013(H7N9) | 1 | 7 | No extrapolation |
| SH1069 | Avian | CY147176 | GenBank | A/pigeon/Shanghai/S1069/2013(H7N9) | 1 | 8 | No extrapolation |
|  |  |  |  |  |  |  |  |
| SH1421 | Avian | CY147177 | GenBank | A/pigeon/Shanghai/S1421/2013(H7N9) | 1 | 1 | No extrapolation |
| SH1421 | Avian | CY147178 | GenBank | A/pigeon/Shanghai/S1421/2013(H7N9) | 1 | 2 | No extrapolation |
| SH1421 | Avian | CY147179 | GenBank | A/pigeon/Shanghai/S1421/2013(H7N9) | 1 | 3 | No extrapolation |
| SH1421 | Avian | CY147180 | GenBank | A/pigeon/Shanghai/S1421/2013(H7N9) | 1 | 4 | No extrapolation |
| SH1421 | Avian | CY147181 | GenBank | A/pigeon/Shanghai/S1421/2013(H7N9) | 1 | 5 | No extrapolation |
| SH1421 | Avian | CY147182 | GenBank | A/pigeon/Shanghai/S1421/2013(H7N9) | 1 | 6 | No extrapolation |
| SH1421 | Avian | CY147183 | GenBank | A/pigeon/Shanghai/S1421/2013(H7N9) | 1 | 7 | No extrapolation |
| SH1421 | Avian | CY147184 | GenBank | A/pigeon/Shanghai/S1421/2013(H7N9) | 1 | 8 | No extrapolation |
|  |  |  |  |  |  |  |  |
| SHANG13 | Human | KC853229 | GenBank | A/Shanghai/4664T/2013(H7N9) | 1 | 1 | No extrapolation |
| SHANG13 | Human | KC853230 | GenBank | A/Shanghai/4664T/2013(H7N9) | 1 | 2 | No extrapolation |
| SHANG13 | Human | KC853226 | GenBank | A/Shanghai/4664T/2013(H7N9) | 1 | 3 | No extrapolation |
| SHANG13 | Human | KC853228 | GenBank | A/Shanghai/4664T/2013(H7N9) | 1 | 4 | No extrapolation |
| SHANG13 | Human | KC853225 | GenBank | A/Shanghai/4664T/2013(H7N9) | 1 | 5 | Partial |
| SHANG13 | Human | KC853231 | GenBank | A/Shanghai/4664T/2013(H7N9) | 1 | 6 | No extrapolation |
| SHANG13 | Human | KC853227 | GenBank | A/Shanghai/4664T/2013(H7N9) | 1 | 7 | No extrapolation |
| SHANG13 | Human | KC853232 | GenBank | A/Shanghai/4664T/2013(H7N9) | 1 | 8 | No extrapolation |
|  |  |  |  |  |  |  |  |
| SI06 | Human | CY119233 | GenBank | A/SolomonIslands/3/2006(H1N1) | 1 | 1 | Full segment |
| SI06 | Human | CY119232 | GenBank | A/SolomonIslands/3/2006(H1N1) | 1 | 2 | Full segment |
| SI06 | Human | CY047425 | GenBank | A/SolomonIslands/3/2006(H1N1) | 1 | 3 | Partial |
| SI06 | Human | EU124177 | GenBank | A/SolomonIslands/3/2006(H1N1) | 1 | 4 | No extrapolation |
| SI06 | Human | CY047398 | GenBank | A/SolomonIslands/3/2006(H1N1) | 1 | 5 | No extrapolation |
| SI06 | Human | EU124136 | GenBank | A/SolomonIslands/3/2006(H1N1) | 1 | 6 | Partial |
| SI06 | Human | CY031341 | GenBank | A/SolomonIslands/3/2006(H1N1) | 1 | 7 | No extrapolation |
| SI06 | Human | CY119230 | GenBank | A/SolomonIslands/3/2006(H1N1) | 1 | 8 | Full segment |
|  |  |  |  |  |  |  |  |
| SI76 | Swine | CY022108 | GenBank | A/swine/Iowa/4/1976(H1N1) | 1 | 1 | No extrapolation |
| SI76 | Swine | CY022107 | GenBank | A/swine/Iowa/4/1976(H1N1) | 1 | 2 | No extrapolation |
| SI76 | Swine | CY022106 | GenBank | A/swine/Iowa/4/1976(H1N1) | 1 | 3 | No extrapolation |
| SI76 | Swine | CY022101 | GenBank | A/swine/Iowa/4/1976(H1N1) | 1 | 4 | No extrapolation |
| SI76 | Swine | CY022104 | GenBank | A/swine/Iowa/4/1976(H1N1) | 1 | 5 | No extrapolation |
| SI76 | Swine | CY022103 | GenBank | A/swine/Iowa/4/1976(H1N1) | 1 | 6 | No extrapolation |
| SI76 | Swine | CY022102 | GenBank | A/swine/Iowa/4/1976(H1N1) | 1 | 7 | No extrapolation |
| SI76 | Swine | CY022105 | GenBank | A/swine/Iowa/4/1976(H1N1) | 1 | 8 | No extrapolation |
|  |  |  |  |  |  |  |  |
| SPN40 | Swine | CY116547 | GenBank | A/swine/Spain/40564/2002(H1N2) | 1 | 1 | No extrapolation |
| SPN40 | Swine | CY116548 | GenBank | A/swine/Spain/40564/2002(H1N2) | 1 | 2 | No extrapolation |
| SPN40 | Swine | CY116549 | GenBank | A/swine/Spain/40564/2002(H1N2) | 1 | 3 | No extrapolation |
| SPN40 | Swine | CY116550 | GenBank | A/swine/Spain/40564/2002(H1N2) | 1 | 4 | No extrapolation |
| SPN40 | Swine | CY116551 | GenBank | A/swine/Spain/40564/2002(H1N2) | 1 | 5 | No extrapolation |
| SPN40 | Swine | CY116552 | GenBank | A/swine/Spain/40564/2002(H1N2) | 1 | 6 | No extrapolation |
| SPN40 | Swine | CY116553 | GenBank | A/swine/Spain/40564/2002(H1N2) | 1 | 7 | No extrapolation |
| SPN40 | Swine | CY116554 | GenBank | A/swine/Spain/40564/2002(H1N2) | 1 | 8 | No extrapolation |
|  |  |  |  |  |  |  |  |
| SPN53 | Swine | CY010587 | GenBank | A/swine/Spain/53207/2004(H1N1) | 1 | 1 | No extrapolation |
| SPN53 | Swine | CY010586 | GenBank | A/swine/Spain/53207/2004(H1N1) | 1 | 2 | No extrapolation |
| SPN53 | Swine | CY010585 | GenBank | A/swine/Spain/53207/2004(H1N1) | 1 | 3 | No extrapolation |
| SPN53 | Swine | CY010580 | GenBank | A/swine/Spain/53207/2004(H1N1) | 1 | 4 | No extrapolation |
| SPN53 | Swine | CY010583 | GenBank | A/swine/Spain/53207/2004(H1N1) | 1 | 5 | No extrapolation |
| SPN53 | Swine | CY010582 | GenBank | A/swine/Spain/53207/2004(H1N1) | 1 | 6 | No extrapolation |
| SPN53 | Swine | CY010581 | GenBank | A/swine/Spain/53207/2004(H1N1) | 1 | 7 | No extrapolation |
| SPN53 | Swine | CY010584 | GenBank | A/swine/Spain/53207/2004(H1N1) | 1 | 8 | No extrapolation |
|  |  |  |  |  |  |  |  |
| SPN54 | Swine | CY010571 | GenBank | A/swine/Spain/54008/2004(H3N2) | 1 | 1 | No extrapolation |
| SPN54 | Swine | CY010570 | GenBank | A/swine/Spain/54008/2004(H3N2) | 1 | 2 | No extrapolation |
| SPN54 | Swine | CY010569 | GenBank | A/swine/Spain/54008/2004(H3N2) | 1 | 3 | No extrapolation |
| SPN54 | Swine | CY010564 | GenBank | A/swine/Spain/54008/2004(H3N2) | 1 | 4 | No extrapolation |
| SPN54 | Swine | CY010567 | GenBank | A/swine/Spain/54008/2004(H3N2) | 1 | 5 | No extrapolation |
| SPN54 | Swine | CY010566 | GenBank | A/swine/Spain/54008/2004(H3N2) | 1 | 6 | No extrapolation |
| SPN54 | Swine | CY010565 | GenBank | A/swine/Spain/54008/2004(H3N2) | 1 | 7 | No extrapolation |
| SPN54 | Swine | CY010568 | GenBank | A/swine/Spain/54008/2004(H3N2) | 1 | 8 | No extrapolation |
|  |  |  |  |  |  |  |  |
| STE09 | Human | KF897769 | GenBank | A/StEtienne/1691/2009(H1N1) | 1 | 1 | No extrapolation |
| STE09 | Human | KF897770 | GenBank | A/StEtienne/1691/2009(H1N1) | 1 | 2 | No extrapolation |
| STE09 | Human | KF897771 | GenBank | A/StEtienne/1691/2009(H1N1) | 1 | 3 | No extrapolation |
| STE09 | Human | JF429396 | GenBank | A/StEtienne/1691/2009(H1N1) | 1 | 4 | No extrapolation |
| STE09 | Human | KF897772 | GenBank | A/StEtienne/1691/2009(H1N1) | 1 | 5 | No extrapolation |
| STE09 | Human | JF429401 | GenBank | A/StEtienne/1691/2009(H1N1) | 1 | 6 | No extrapolation |
| STE09 | Human | KF897773 | GenBank | A/StEtienne/1691/2009(H1N1) | 1 | 7 | No extrapolation |
| STE09 | Human | KF897774 | GenBank | A/StEtienne/1691/2009(H1N1) | 1 | 8 | No extrapolation |
|  |  |  |  |  |  |  |  |
| STE10 | Human | KF897790 | GenBank | A/StEtienne/1139/2010(H1N1) | 1 | 1 | No extrapolation |
| STE10 | Human | KF897791 | GenBank | A/StEtienne/1139/2010(H1N1) | 1 | 2 | No extrapolation |
| STE10 | Human | KF897792 | GenBank | A/StEtienne/1139/2010(H1N1) | 1 | 3 | No extrapolation |
| STE10 | Human | KF897793 | GenBank | A/StEtienne/1139/2010(H1N1) | 1 | 4 | No extrapolation |
| STE10 | Human | KF897794 | GenBank | A/StEtienne/1139/2010(H1N1) | 1 | 5 | No extrapolation |
| STE10 | Human | KF897795 | GenBank | A/StEtienne/1139/2010(H1N1) | 1 | 6 | No extrapolation |
| STE10 | Human | KF897796 | GenBank | A/StEtienne/1139/2010(H1N1) | 1 | 7 | Partial |
| STE10 | Human | KF897797 | GenBank | A/StEtienne/1139/2010(H1N1) | 1 | 8 | No extrapolation |
|  |  |  |  |  |  |  |  |
| SWE1021 | Swine | GQ495129 | GenBank | A/swine/Sweden/1021/2009(H1N2) | 1 | 1 | No extrapolation |
| SWE1021 | Swine | GQ495130 | GenBank | A/swine/Sweden/1021/2009(H1N2) | 1 | 2 | No extrapolation |
| SWE1021 | Swine | GQ495131 | GenBank | A/swine/Sweden/1021/2009(H1N2) | 1 | 3 | No extrapolation |
| SWE1021 | Swine | GQ495132 | GenBank | A/swine/Sweden/1021/2009(H1N2) | 1 | 4 | No extrapolation |
| SWE1021 | Swine | GQ495133 | GenBank | A/swine/Sweden/1021/2009(H1N2) | 1 | 5 | No extrapolation |
| SWE1021 | Swine | GQ495134 | GenBank | A/swine/Sweden/1021/2009(H1N2) | 1 | 6 | Partial |
| SWE1021 | Swine | GQ495135 | GenBank | A/swine/Sweden/1021/2009(H1N2) | 1 | 7 | Partial |
| SWE1021 | Swine | GQ495136 | GenBank | A/swine/Sweden/1021/2009(H1N2) | 1 | 8 | No extrapolation |
|  |  |  |  |  |  |  |  |
| SWE9706 | Swine | HM626479 | GenBank | A/swine/Sweden/9706/2010(H1N2) | 1 | 1 | No extrapolation |
| SWE9706 | Swine | HM626480 | GenBank | A/swine/Sweden/9706/2010(H1N2) | 1 | 2 | No extrapolation |
| SWE9706 | Swine | HM626481 | GenBank | A/swine/Sweden/9706/2010(H1N2) | 1 | 3 | No extrapolation |
| SWE9706 | Swine | HM626482 | GenBank | A/swine/Sweden/9706/2010(H1N2) | 1 | 4 | No extrapolation |
| SWE9706 | Swine | HM626483 | GenBank | A/swine/Sweden/9706/2010(H1N2) | 1 | 5 | No extrapolation |
| SWE9706 | Swine | HM626484 | GenBank | A/swine/Sweden/9706/2010(H1N2) | 1 | 6 | Partial |
| SWE9706 | Swine | HM626485 | GenBank | A/swine/Sweden/9706/2010(H1N2) | 1 | 7 | Partial |
| SWE9706 | Swine | HM626486 | GenBank | A/swine/Sweden/9706/2010(H1N2) | 1 | 8 | No extrapolation |
|  |  |  |  |  |  |  |  |
| SY01 | Human | CY128218 | GenBank | A/Seoul/Y-01/2009(H1N1) | 1 | 1 | Full segment |
| SY01 | Human | CY128217 | GenBank | A/Seoul/Y-01/2009(H1N1) | 1 | 2 | Full segment |
| SY01 | Human | CY128216 | GenBank | A/Seoul/Y-01/2009(H1N1) | 1 | 3 | Full segment |
| SY01 | Human | EPI272297 | GISAID | A/Seoul/Y-01/2009(H1N1) | 1 | 4 | No extrapolation |
| SY01 | Human | CY128214 | GenBank | A/Seoul/Y-01/2009(H1N1) | 1 | 5 | Full segment |
| SY01 | Human | EPI272298 | GISAID | A/Seoul/Y-01/2009(H1N1) | 1 | 6 | No extrapolation |
| SY01 | Human | CY128212 | GenBank | A/Seoul/Y-01/2009(H1N1) | 1 | 7 | Full segment |
| SY01 | Human | CY128215 | GenBank | A/Seoul/Y-01/2009(H1N1) | 1 | 8 | Full segment |
|  |  |  |  |  |  |  |  |
| THAI04v1 | Human | AY626149 | GenBank | A/Thailand/KAN-1/2004(H5N1) | 1 | 1 | No extrapolation |
| THAI04v1 | Human | AY626148 | GenBank | A/Thailand/KAN-1/2004(H5N1) | 1 | 2 | No extrapolation |
| THAI04v1 | Human | AY626147 | GenBank | A/Thailand/KAN-1/2004(H5N1) | 1 | 3 | No extrapolation |
| THAI04v1 | Human | AY555150 | GenBank | A/Thailand/KAN-1/2004(H5N1) | 1 | 4 | No extrapolation |
| THAI04v1 | Human | AY626145 | GenBank | A/Thailand/KAN-1/2004(H5N1) | 1 | 5 | No extrapolation |
| THAI04v1 | Human | AY555151 | GenBank | A/Thailand/KAN-1/2004(H5N1) | 1 | 6 | No extrapolation |
| THAI04v1 | Human | AY626144 | GenBank | A/Thailand/KAN-1/2004(H5N1) | 1 | 7 | No extrapolation |
| THAI04v1 | Human | AY626146 | GenBank | A/Thailand/KAN-1/2004(H5N1) | 1 | 8 | No extrapolation |
|  |  |  |  |  |  |  |  |
| THAI04v2 | Human | CY111595 | GenBank | A/Thailand/KAN-1/2004(H5N1) | 2 | 1 | No extrapolation |
| THAI04v2 | Human | CY111596 | GenBank | A/Thailand/KAN-1/2004(H5N1) | 2 | 2 | No extrapolation |
| THAI04v2 | Human | CY111597 | GenBank | A/Thailand/KAN-1/2004(H5N1) | 2 | 3 | No extrapolation |
| THAI04v2 | Human | CY111598 | GenBank | A/Thailand/KAN-1/2004(H5N1) | 2 | 4 | No extrapolation |
| THAI04v2 | Human | CY111599 | GenBank | A/Thailand/KAN-1/2004(H5N1) | 2 | 5 | No extrapolation |
| THAI04v2 | Human | CY111600 | GenBank | A/Thailand/KAN-1/2004(H5N1) | 2 | 6 | No extrapolation |
| THAI04v2 | Human | CY111601 | GenBank | A/Thailand/KAN-1/2004(H5N1) | 2 | 7 | No extrapolation |
| THAI04v2 | Human | CY111602 | GenBank | A/Thailand/KAN-1/2004(H5N1) | 2 | 8 | No extrapolation |
|  |  |  |  |  |  |  |  |
| THAI16v1 | Human | EU268216 | GenBank | A/Thailand/16/2004(H5N1) | 1 | 1 | No extrapolation |
| THAI16v1 | Human | EU268217 | GenBank | A/Thailand/16/2004(H5N1) | 1 | 2 | No extrapolation |
| THAI16v1 | Human | EU268218 | GenBank | A/Thailand/16/2004(H5N1) | 1 | 3 | No extrapolation |
| THAI16v1 | Human | EU268219 | GenBank | A/Thailand/16/2004(H5N1) | 1 | 4 | No extrapolation |
| THAI16v1 | Human | EU268220 | GenBank | A/Thailand/16/2004(H5N1) | 1 | 5 | No extrapolation |
| THAI16v1 | Human | EU268221 | GenBank | A/Thailand/16/2004(H5N1) | 1 | 6 | No extrapolation |
| THAI16v1 | Human | EU268222 | GenBank | A/Thailand/16/2004(H5N1) | 1 | 7 | No extrapolation |
| THAI16v1 | Human | EU268223 | GenBank | A/Thailand/16/2004(H5N1) | 1 | 8 | No extrapolation |
|  |  |  |  |  |  |  |  |
| THAI16v2 | Human | EF467807 | GenBank | A/Thailand/16/2004(H5N1) | 2 | 1 | No extrapolation |
| THAI16v2 | Human | EF467810 | GenBank | A/Thailand/16/2004(H5N1) | 2 | 2 | No extrapolation |
| THAI16v2 | Human | EF473503 | GenBank | A/Thailand/16/2004(H5N1) | 2 | 3 | Partial |
| THAI16v2 | Human | EF541408 | GenBank | A/Thailand/16/2004(H5N1) | 2 | 4 | No extrapolation |
| THAI16v2 | Human | EF541459 | GenBank | A/Thailand/16/2004(H5N1) | 2 | 5 | No extrapolation |
| THAI16v2 | Human | EF541470 | GenBank | A/Thailand/16/2004(H5N1) | 2 | 6 | No extrapolation |
| THAI16v2 | Human | EF541448 | GenBank | A/Thailand/16/2004(H5N1) | 2 | 7 | No extrapolation |
| THAI16v2 | Human | EF541458 | GenBank | A/Thailand/16/2004(H5N1) | 2 | 8 | No extrapolation |
|  |  |  |  |  |  |  |  |
| TN560 | Human | CY041538 | GenBank | A/Tennessee/1-560/2009(H1N1) | 1 | 1 | No extrapolation |
| TN560 | Human | CY041539 | GenBank | A/Tennessee/1-560/2009(H1N1) | 1 | 2 | No extrapolation |
| TN560 | Human | CY041540 | GenBank | A/Tennessee/1-560/2009(H1N1) | 1 | 3 | No extrapolation |
| TN560 | Human | CY040457 | GenBank | A/Tennessee/1-560/2009(H1N1) | 1 | 4 | Partial |
| TN560 | Human | KY926173 | GenBank | A/Tennessee/1-560/2009(H1N1) | 1 | 5 | Full segment |
| TN560 | Human | CY040458 | GenBank | A/Tennessee/1-560/2009(H1N1) | 1 | 6 | Partial |
| TN560 | Human | KY926049 | GenBank | A/Tennessee/1-560/2009(H1N1) | 1 | 7 | Full segment |
| TN560 | Human | KY925946 | GenBank | A/Tennessee/1-560/2009(H1N1) | 1 | 8 | Full segment |
|  |  |  |  |  |  |  |  |
| TX09 | Human | GQ200256 | GenBank | A/Texas/15/2009(H1N1) | 1 | 1 | No extrapolation |
| TX09 | Human | GQ122094 | GenBank | A/Texas/15/2009(H1N1) | 1 | 2 | No extrapolation |
| TX09 | Human | GQ200257 | GenBank | A/Texas/15/2009(H1N1) | 1 | 3 | No extrapolation |
| TX09 | Human | GQ122097 | GenBank | A/Texas/15/2009(H1N1) | 1 | 4 | No extrapolation |
| TX09 | Human | GQ122092 | GenBank | A/Texas/15/2009(H1N1) | 1 | 5 | No extrapolation |
| TX09 | Human | GQ122096 | GenBank | A/Texas/15/2009(H1N1) | 1 | 6 | No extrapolation |
| TX09 | Human | GQ122095 | GenBank | A/Texas/15/2009(H1N1) | 1 | 7 | No extrapolation |
| TX09 | Human | GQ122093 | GenBank | A/Texas/15/2009(H1N1) | 1 | 8 | No extrapolation |
|  |  |  |  |  |  |  |  |
| TX91v1 | Human | CY009323 | GenBank | A/Texas/36/1991(H1N1) | 1 | 1 | No extrapolation |
| TX91v1 | Human | CY009322 | GenBank | A/Texas/36/1991(H1N1) | 1 | 2 | No extrapolation |
| TX91v1 | Human | CY009321 | GenBank | A/Texas/36/1991(H1N1) | 1 | 3 | No extrapolation |
| TX91v1 | Human | CY009316 | GenBank | A/Texas/36/1991(H1N1) | 1 | 4 | No extrapolation |
| TX91v1 | Human | CY009319 | GenBank | A/Texas/36/1991(H1N1) | 1 | 5 | No extrapolation |
| TX91v1 | Human | CY009318 | GenBank | A/Texas/36/1991(H1N1) | 1 | 6 | No extrapolation |
| TX91v1 | Human | CY009317 | GenBank | A/Texas/36/1991(H1N1) | 1 | 7 | No extrapolation |
| TX91v1 | Human | CY009320 | GenBank | A/Texas/36/1991(H1N1) | 1 | 8 | No extrapolation |
|  |  |  |  |  |  |  |  |
| TX91v2 | Human | CY033605 | GenBank | A/Texas/36/1991(H1N1) | 2 | 1 | No extrapolation |
| TX91v2 | Human | CY033604 | GenBank | A/Texas/36/1991(H1N1) | 2 | 2 | No extrapolation |
| TX91v2 | Human | CY033603 | GenBank | A/Texas/36/1991(H1N1) | 2 | 3 | No extrapolation |
| TX91v2 | Human | CY033655 | GenBank | A/Texas/36/1991(H1N1) | 2 | 4 | No extrapolation |
| TX91v2 | Human | CY033601 | GenBank | A/Texas/36/1991(H1N1) | 2 | 5 | No extrapolation |
| TX91v2 | Human | CY033600 | GenBank | A/Texas/36/1991(H1N1) | 2 | 6 | No extrapolation |
| TX91v2 | Human | CY033656 | GenBank | A/Texas/36/1991(H1N1) | 2 | 7 | No extrapolation |
| TX91v2 | Human | CY033602 | GenBank | A/Texas/36/1991(H1N1) | 2 | 8 | No extrapolation |
|  |  |  |  |  |  |  |  |
| TX91v3 | Human | DQ508886 | GenBank | A/Texas/36/1991(H1N1) | 3 | 1 | No extrapolation |
| TX91v3 | Human | DQ508887 | GenBank | A/Texas/36/1991(H1N1) | 3 | 2 | No extrapolation |
| TX91v3 | Human | DQ508888 | GenBank | A/Texas/36/1991(H1N1) | 3 | 3 | No extrapolation |
| TX91v3 | Human | DQ508889 | GenBank | A/Texas/36/1991(H1N1) | 3 | 4 | No extrapolation |
| TX91v3 | Human | DQ508890 | GenBank | A/Texas/36/1991(H1N1) | 3 | 5 | No extrapolation |
| TX91v3 | Human | DQ508891 | GenBank | A/Texas/36/1991(H1N1) | 3 | 6 | No extrapolation |
| TX91v3 | Human | DQ508892 | GenBank | A/Texas/36/1991(H1N1) | 3 | 7 | No extrapolation |
| TX91v3 | Human | DQ508893 | GenBank | A/Texas/36/1991(H1N1) | 3 | 8 | No extrapolation |
|  |  |  |  |  |  |  |  |
| TX98 | Swine | CY095672 | GenBank | A/swine/Texas/4199-2/1998(H3N2) | 1 | 1 | No extrapolation |
| TX98 | Swine | CY095673 | GenBank | A/swine/Texas/4199-2/1998(H3N2) | 1 | 2 | No extrapolation |
| TX98 | Swine | CY095674 | GenBank | A/swine/Texas/4199-2/1998(H3N2) | 1 | 3 | No extrapolation |
| TX98 | Swine | CY095675 | GenBank | A/swine/Texas/4199-2/1998(H3N2) | 1 | 4 | No extrapolation |
| TX98 | Swine | CY095676 | GenBank | A/swine/Texas/4199-2/1998(H3N2) | 1 | 5 | No extrapolation |
| TX98 | Swine | CY095677 | GenBank | A/swine/Texas/4199-2/1998(H3N2) | 1 | 6 | No extrapolation |
| TX98 | Swine | CY095678 | GenBank | A/swine/Texas/4199-2/1998(H3N2) | 1 | 7 | No extrapolation |
| TX98 | Swine | CY095679 | GenBank | A/swine/Texas/4199-2/1998(H3N2) | 1 | 8 | No extrapolation |
|  |  |  |  |  |  |  |  |
| UKR63v1 | Duck | CY005819 | GenBank | A/duck/Ukraine/1/1963(H3N8) | 1 | 1 | No extrapolation |
| UKR63v1 | Duck | CY005818 | GenBank | A/duck/Ukraine/1/1963(H3N8) | 1 | 2 | No extrapolation |
| UKR63v1 | Duck | CY005817 | GenBank | A/duck/Ukraine/1/1963(H3N8) | 1 | 3 | No extrapolation |
| UKR63v1 | Duck | CY006038 | GenBank | A/duck/Ukraine/1/1963(H3N8) | 1 | 4 | No extrapolation |
| UKR63v1 | Duck | CY005815 | GenBank | A/duck/Ukraine/1/1963(H3N8) | 1 | 5 | No extrapolation |
| UKR63v1 | Duck | CY014648 | GenBank | A/duck/Ukraine/1/1963(H3N8) | 1 | 6 | No extrapolation |
| UKR63v1 | Duck | CY005814 | GenBank | A/duck/Ukraine/1/1963(H3N8) | 1 | 7 | No extrapolation |
| UKR63v1 | Duck | CY005816 | GenBank | A/duck/Ukraine/1/1963(H3N8) | 1 | 8 | No extrapolation |
|  |  |  |  |  |  |  |  |
| UKR63v2 | Duck | CY130021 | GenBank | A/duck/Ukraine/1/1963(H3N8) | 2 | 1 | No extrapolation |
| UKR63v2 | Duck | CY130020 | GenBank | A/duck/Ukraine/1/1963(H3N8) | 2 | 2 | No extrapolation |
| UKR63v2 | Duck | CY130019 | GenBank | A/duck/Ukraine/1/1963(H3N8) | 2 | 3 | No extrapolation |
| UKR63v2 | Duck | CY130014 | GenBank | A/duck/Ukraine/1/1963(H3N8) | 2 | 4 | No extrapolation |
| UKR63v2 | Duck | CY130017 | GenBank | A/duck/Ukraine/1/1963(H3N8) | 2 | 5 | No extrapolation |
| UKR63v2 | Duck | CY130016 | GenBank | A/duck/Ukraine/1/1963(H3N8) | 2 | 6 | No extrapolation |
| UKR63v2 | Duck | CY130015 | GenBank | A/duck/Ukraine/1/1963(H3N8) | 2 | 7 | No extrapolation |
| UKR63v2 | Duck | CY130018 | GenBank | A/duck/Ukraine/1/1963(H3N8) | 2 | 8 | No extrapolation |
|  |  |  |  |  |  |  |  |
| UT95 | Avian | EU980473 | GenBank | A/turkey/Utah/24721-10/1995(H7N3) | 1 | 1 | No extrapolation |
| UT95 | Avian | EU980472 | GenBank | A/turkey/Utah/24721-10/1995(H7N3) | 1 | 2 | No extrapolation |
| UT95 | Avian | EU980471 | GenBank | A/turkey/Utah/24721-10/1995(H7N3) | 1 | 3 | No extrapolation |
| UT95 | Avian | EF470585 | GenBank | A/turkey/Utah/24721-10/1995(H7N3) | 1 | 4 | No extrapolation |
| UT95 | Avian | EU980470 | GenBank | A/turkey/Utah/24721-10/1995(H7N3) | 1 | 5 | No extrapolation |
| UT95 | Avian | EU980469 | GenBank | A/turkey/Utah/24721-10/1995(H7N3) | 1 | 6 | No extrapolation |
| UT95 | Avian | AF073201 | GenBank | A/turkey/Utah/24721-10/1995(H7N3) | 1 | 7 | No extrapolation |
| UT95 | Avian | AF074284 | GenBank | A/turkey/Utah/24721-10/1995(H7N3) | 1 | 8 | No extrapolation |
|  |  |  |  |  |  |  |  |
| UTK09 | Human | CY043494 | GenBank | A/Kawasaki/UTK-4/2009(H1N1) | 1 | 1 | Full segment |
| UTK09 | Human | CY043495 | GenBank | A/Kawasaki/UTK-4/2009(H1N1) | 1 | 2 | Full segment |
| UTK09 | Human | CY043496 | GenBank | A/Kawasaki/UTK-4/2009(H1N1) | 1 | 3 | Full segment |
| UTK09 | Human | AB671291 | GenBank | A/Kawasaki/UTK-4/2009(H1N1) | 1 | 4 | No extrapolation |
| UTK09 | Human | CY043498 | GenBank | A/Kawasaki/UTK-4/2009(H1N1) | 1 | 5 | Full segment |
| UTK09 | Human | AB671292 | GenBank | A/Kawasaki/UTK-4/2009(H1N1) | 1 | 6 | No extrapolation |
| UTK09 | Human | CY043500 | GenBank | A/Kawasaki/UTK-4/2009(H1N1) | 1 | 7 | Full segment |
| UTK09 | Human | CY043501 | GenBank | A/Kawasaki/UTK-4/2009(H1N1) | 1 | 8 | Full segment |
|  |  |  |  |  |  |  |  |
| VA02 | Avian | EU982307 | GenBank | A/turkey/VA/55/2002(H7N2) | 1 | 1 | No extrapolation |
| VA02 | Avian | EU982306 | GenBank | A/turkey/VA/55/2002(H7N2) | 1 | 2 | No extrapolation |
| VA02 | Avian | EU982305 | GenBank | A/turkey/VA/55/2002(H7N2) | 1 | 3 | No extrapolation |
| VA02 | Avian | AY240912 | GenBank | A/turkey/VA/55/2002(H7N2) | 1 | 4 | No extrapolation |
| VA02 | Avian | EU982304 | GenBank | A/turkey/VA/55/2002(H7N2) | 1 | 5 | No extrapolation |
| VA02 | Avian | AY254146 | GenBank | A/turkey/VA/55/2002(H7N2) | 1 | 6 | No extrapolation |
| VA02 | Avian | AY241623 | GenBank | A/turkey/VA/55/2002(H7N2) | 1 | 7 | No extrapolation |
| VA02 | Avian | AY241660 | GenBank | A/turkey/VA/55/2002(H7N2) | 1 | 8 | No extrapolation |
|  |  |  |  |  |  |  |  |
| VIC75v1 | Human | CY121204 | GenBank | A/Victoria/3/1975(H3N2) | 1 | 1 | No extrapolation |
| VIC75v1 | Human | CY121203 | GenBank | A/Victoria/3/1975(H3N2) | 1 | 2 | No extrapolation |
| VIC75v1 | Human | CY121202 | GenBank | A/Victoria/3/1975(H3N2) | 1 | 3 | No extrapolation |
| VIC75v1 | Human | CY121197 | GenBank | A/Victoria/3/1975(H3N2) | 1 | 4 | No extrapolation |
| VIC75v1 | Human | CY121200 | GenBank | A/Victoria/3/1975(H3N2) | 1 | 5 | No extrapolation |
| VIC75v1 | Human | CY121199 | GenBank | A/Victoria/3/1975(H3N2) | 1 | 6 | No extrapolation |
| VIC75v1 | Human | CY121198 | GenBank | A/Victoria/3/1975(H3N2) | 1 | 7 | No extrapolation |
| VIC75v1 | Human | CY121201 | GenBank | A/Victoria/3/1975(H3N2) | 1 | 8 | No extrapolation |
|  |  |  |  |  |  |  |  |
| VIC75v2 | Human | CY113188 | GenBank | A/Victoria/3/1975(H3N2) | 2 | 1 | No extrapolation |
| VIC75v2 | Human | CY113187 | GenBank | A/Victoria/3/1975(H3N2) | 2 | 2 | No extrapolation |
| VIC75v2 | Human | CY113186 | GenBank | A/Victoria/3/1975(H3N2) | 2 | 3 | No extrapolation |
| VIC75v2 | Human | CY113181 | GenBank | A/Victoria/3/1975(H3N2) | 2 | 4 | No extrapolation |
| VIC75v2 | Human | CY113184 | GenBank | A/Victoria/3/1975(H3N2) | 2 | 5 | No extrapolation |
| VIC75v2 | Human | CY113183 | GenBank | A/Victoria/3/1975(H3N2) | 2 | 6 | No extrapolation |
| VIC75v2 | Human | CY113182 | GenBank | A/Victoria/3/1975(H3N2) | 2 | 7 | No extrapolation |
| VIC75v2 | Human | CY113185 | GenBank | A/Victoria/3/1975(H3N2) | 2 | 8 | No extrapolation |
|  |  |  |  |  |  |  |  |
| VN03 | Avian | DQ492869 | GenBank | A/chicken/VietNam/8/2003(H5N1) | 1 | 1 | Partial |
| VN03 | Avian | DQ493393 | GenBank | A/chicken/VietNam/8/2003(H5N1) | 1 | 2 | Partial |
| VN03 | Avian | DQ493306 | GenBank | A/chicken/VietNam/8/2003(H5N1) | 1 | 3 | Partial |
| VN03 | Avian | DQ497693 | GenBank | A/chicken/VietNam/8/2003(H5N1) | 1 | 4 | Partial |
| VN03 | Avian | DQ493131 | GenBank | A/chicken/VietNam/8/2003(H5N1) | 1 | 5 | Partial |
| VN03 | Avian | DQ493042 | GenBank | A/chicken/VietNam/8/2003(H5N1) | 1 | 6 | No extrapolation |
| VN03 | Avian | DQ492954 | GenBank | A/chicken/VietNam/8/2003(H5N1) | 1 | 7 | Partial |
| VN03 | Avian | DQ493218 | GenBank | A/chicken/VietNam/8/2003(H5N1) | 1 | 8 | Partial |
|  |  |  |  |  |  |  |  |
| VN1203v1 | Human | AY818126 | GenBank | A/VietNam/1203/2004(H5N1) | 1 | 1 | No extrapolation |
| VN1203v1 | Human | AY818129 | GenBank | A/VietNam/1203/2004(H5N1) | 1 | 2 | No extrapolation |
| VN1203v1 | Human | AY818132 | GenBank | A/VietNam/1203/2004(H5N1) | 1 | 3 | No extrapolation |
| VN1203v1 | Human | AY818135 | GenBank | A/VietNam/1203/2004(H5N1) | 1 | 4 | No extrapolation |
| VN1203v1 | Human | AY818138 | GenBank | A/VietNam/1203/2004(H5N1) | 1 | 5 | No extrapolation |
| VN1203v1 | Human | AY818141 | GenBank | A/VietNam/1203/2004(H5N1) | 1 | 6 | No extrapolation |
| VN1203v1 | Human | AY818144 | GenBank | A/VietNam/1203/2004(H5N1) | 1 | 7 | No extrapolation |
| VN1203v1 | Human | AY818147 | GenBank | A/VietNam/1203/2004(H5N1) | 1 | 8 | No extrapolation |
|  |  |  |  |  |  |  |  |
| VN1203v2 | Human | HM006756 | GenBank | A/VietNam/1203/2004(H5N1) | 2 | 1 | No extrapolation |
| VN1203v2 | Human | HM006757 | GenBank | A/VietNam/1203/2004(H5N1) | 2 | 2 | No extrapolation |
| VN1203v2 | Human | HM006758 | GenBank | A/VietNam/1203/2004(H5N1) | 2 | 3 | No extrapolation |
| VN1203v2 | Human | HM006759 | GenBank | A/VietNam/1203/2004(H5N1) | 2 | 4 | No extrapolation |
| VN1203v2 | Human | HM006760 | GenBank | A/VietNam/1203/2004(H5N1) | 2 | 5 | No extrapolation |
| VN1203v2 | Human | HM006761 | GenBank | A/VietNam/1203/2004(H5N1) | 2 | 6 | No extrapolation |
| VN1203v2 | Human | HM006762 | GenBank | A/VietNam/1203/2004(H5N1) | 2 | 7 | No extrapolation |
| VN1203v2 | Human | HM006763 | GenBank | A/VietNam/1203/2004(H5N1) | 2 | 8 | No extrapolation |
|  |  |  |  |  |  |  |  |
| VN1204 | Human | EF467806 | GenBank | A/VietNam/1204/2004(H5N1) | 1 | 1 | No extrapolation |
| VN1204 | Human | EF467809 | GenBank | A/VietNam/1204/2004(H5N1) | 1 | 2 | No extrapolation |
| VN1204 | Human | EF473407 | GenBank | A/VietNam/1204/2004(H5N1) | 1 | 3 | Partial |
| VN1204 | Human | EF541404 | GenBank | A/VietNam/1204/2004(H5N1) | 1 | 4 | No extrapolation |
| VN1204 | Human | HM006760 | GenBank | A/VietNam/1204/2004(H5N1) | 1 | 5 | Full segment |
| VN1204 | Human | AB745467 | GenBank | A/VietNam/1204/2004(H5N1) | 1 | 6 | No extrapolation |
| VN1204 | Human | HM006762 | GenBank | A/VietNam/1204/2004(H5N1) | 1 | 7 | Full segment |
| VN1204 | Human | EF541457 | GenBank | A/VietNam/1204/2004(H5N1) | 1 | 8 | No extrapolation |
|  |  |  |  |  |  |  |  |
| W452 | Mallard | KJ746108 | GenBank | A/mallard/Korea/W452/2014(H5N8) | 1 | 1 | No extrapolation |
| W452 | Mallard | KJ746109 | GenBank | A/mallard/Korea/W452/2014(H5N8) | 1 | 2 | No extrapolation |
| W452 | Mallard | KJ746110 | GenBank | A/mallard/Korea/W452/2014(H5N8) | 1 | 3 | No extrapolation |
| W452 | Mallard | KJ746111 | GenBank | A/mallard/Korea/W452/2014(H5N8) | 1 | 4 | No extrapolation |
| W452 | Mallard | KJ746112 | GenBank | A/mallard/Korea/W452/2014(H5N8) | 1 | 5 | No extrapolation |
| W452 | Mallard | KJ746113 | GenBank | A/mallard/Korea/W452/2014(H5N8) | 1 | 6 | No extrapolation |
| W452 | Mallard | KJ746114 | GenBank | A/mallard/Korea/W452/2014(H5N8) | 1 | 7 | No extrapolation |
| W452 | Mallard | KJ746115 | GenBank | A/mallard/Korea/W452/2014(H5N8) | 1 | 8 | No extrapolation |
|  |  |  |  |  |  |  |  |
| W468 | Environment | KX298045 | GenBank | A/environment/Korea/W468/2014(H5N8) | 1 | 1 | No extrapolation |
| W468 | Environment | KX298020 | GenBank | A/environment/Korea/W468/2014(H5N8) | 1 | 2 | No extrapolation |
| W468 | Environment | KX297995 | GenBank | A/environment/Korea/W468/2014(H5N8) | 1 | 3 | No extrapolation |
| W468 | Environment | KX297870 | GenBank | A/environment/Korea/W468/2014(H5N8) | 1 | 4 | No extrapolation |
| W468 | Environment | KX297945 | GenBank | A/environment/Korea/W468/2014(H5N8) | 1 | 5 | No extrapolation |
| W468 | Environment | KX297920 | GenBank | A/environment/Korea/W468/2014(H5N8) | 1 | 6 | No extrapolation |
| W468 | Environment | KX297895 | GenBank | A/environment/Korea/W468/2014(H5N8) | 1 | 7 | No extrapolation |
| W468 | Environment | KX297970 | GenBank | A/environment/Korea/W468/2014(H5N8) | 1 | 8 | No extrapolation |
|  |  |  |  |  |  |  |  |
| WISC67v1 | Human | CY034123 | GenBank | A/Wisconsin/67/2005(H3N2) | 1 | 1 | No extrapolation |
| WISC67v1 | Human | CY034122 | GenBank | A/Wisconsin/67/2005(H3N2) | 1 | 2 | No extrapolation |
| WISC67v1 | Human | CY034121 | GenBank | A/Wisconsin/67/2005(H3N2) | 1 | 3 | No extrapolation |
| WISC67v1 | Human | CY034116 | GenBank | A/Wisconsin/67/2005(H3N2) | 1 | 4 | No extrapolation |
| WISC67v1 | Human | CY034119 | GenBank | A/Wisconsin/67/2005(H3N2) | 1 | 5 | No extrapolation |
| WISC67v1 | Human | CY034118 | GenBank | A/Wisconsin/67/2005(H3N2) | 1 | 6 | No extrapolation |
| WISC67v1 | Human | CY034117 | GenBank | A/Wisconsin/67/2005(H3N2) | 1 | 7 | No extrapolation |
| WISC67v1 | Human | CY034120 | GenBank | A/Wisconsin/67/2005(H3N2) | 1 | 8 | No extrapolation |
|  |  |  |  |  |  |  |  |
| WISC67v2 | Human | CY114388 | GenBank | A/Wisconsin/67/2005(H3N2) | 2 | 1 | No extrapolation |
| WISC67v2 | Human | CY114387 | GenBank | A/Wisconsin/67/2005(H3N2) | 2 | 2 | No extrapolation |
| WISC67v2 | Human | CY114386 | GenBank | A/Wisconsin/67/2005(H3N2) | 2 | 3 | No extrapolation |
| WISC67v2 | Human | CY114381 | GenBank | A/Wisconsin/67/2005(H3N2) | 2 | 4 | No extrapolation |
| WISC67v2 | Human | CY114384 | GenBank | A/Wisconsin/67/2005(H3N2) | 2 | 5 | No extrapolation |
| WISC67v2 | Human | CY114383 | GenBank | A/Wisconsin/67/2005(H3N2) | 2 | 6 | No extrapolation |
| WISC67v2 | Human | CY114382 | GenBank | A/Wisconsin/67/2005(H3N2) | 2 | 7 | No extrapolation |
| WISC67v2 | Human | CY114385 | GenBank | A/Wisconsin/67/2005(H3N2) | 2 | 8 | No extrapolation |
|  |  |  |  |  |  |  |  |
| WSN33 | Human | CY034139 | GenBank | A/WSN/1933(H1N1) | 1 | 1 | No extrapolation |
| WSN33 | Human | CY034138 | GenBank | A/WSN/1933(H1N1) | 1 | 2 | No extrapolation |
| WSN33 | Human | CY034137 | GenBank | A/WSN/1933(H1N1) | 1 | 3 | No extrapolation |
| WSN33 | Human | CY034132 | GenBank | A/WSN/1933(H1N1) | 1 | 4 | No extrapolation |
| WSN33 | Human | CY034135 | GenBank | A/WSN/1933(H1N1) | 1 | 5 | No extrapolation |
| WSN33 | Human | CY034134 | GenBank | A/WSN/1933(H1N1) | 1 | 6 | No extrapolation |
| WSN33 | Human | CY034133 | GenBank | A/WSN/1933(H1N1) | 1 | 7 | No extrapolation |
| WSN33 | Human | CY034136 | GenBank | A/WSN/1933(H1N1) | 1 | 8 | No extrapolation |
|  |  |  |  |  |  |  |  |
| X-31 | Human | DQ874873 | GenBank | rA/X-31(H3N2) | 1 | 1 | No extrapolation |
| X-31 | Human | DQ874874 | GenBank | rA/X-31(H3N2) | 1 | 2 | No extrapolation |
| X-31 | Human | DQ874875 | GenBank | rA/X-31(H3N2) | 1 | 3 | No extrapolation |
| X-31 | Human | DQ874876 | GenBank | rA/X-31(H3N2) | 1 | 4 | No extrapolation |
| X-31 | Human | DQ874877 | GenBank | rA/X-31(H3N2) | 1 | 5 | No extrapolation |
| X-31 | Human | DQ874878 | GenBank | rA/X-31(H3N2) | 1 | 6 | No extrapolation |
| X-31 | Human | DQ874879 | GenBank | rA/X-31(H3N2) | 1 | 7 | No extrapolation |
| X-31 | Human | DQ874880 | GenBank | rA/X-31(H3N2) | 1 | 8 | No extrapolation |
|  |  |  |  |  |  |  |  |
| X-31B | Human | CY044308 | GenBank | rA/X-31(H3N2) | 2 | 1 | No extrapolation |
| X-31B | Human | CY044307 | GenBank | rA/X-31(H3N2) | 2 | 2 | No extrapolation |
| X-31B | Human | CY044306 | GenBank | rA/X-31(H3N2) | 2 | 3 | No extrapolation |
| X-31B | Human | CY044301 | GenBank | rA/X-31(H3N2) | 2 | 4 | No extrapolation |
| X-31B | Human | CY044304 | GenBank | rA/X-31(H3N2) | 2 | 5 | No extrapolation |
| X-31B | Human | CY044303 | GenBank | rA/X-31(H3N2) | 2 | 6 | No extrapolation |
| X-31B | Human | CY044302 | GenBank | rA/X-31(H3N2) | 2 | 7 | No extrapolation |
| X-31B | Human | CY044305 | GenBank | rA/X-31(H3N2) | 2 | 8 | No extrapolation |
|  |  |  |  |  |  |  |  |
| X-79 | Human | CY121260 | GenBank | rA/X-79(H3N2) | 1 | 1 | No extrapolation |
| X-79 | Human | CY121259 | GenBank | rA/X-79(H3N2) | 1 | 2 | No extrapolation |
| X-79 | Human | CY121258 | GenBank | rA/X-79(H3N2) | 1 | 3 | No extrapolation |
| X-79 | Human | CY121253 | GenBank | rA/X-79(H3N2) | 1 | 4 | No extrapolation |
| X-79 | Human | CY121256 | GenBank | rA/X-79(H3N2) | 1 | 5 | No extrapolation |
| X-79 | Human | CY121255 | GenBank | rA/X-79(H3N2) | 1 | 6 | No extrapolation |
| X-79 | Human | CY121254 | GenBank | rA/X-79(H3N2) | 1 | 7 | No extrapolation |
| X-79 | Human | CY121257 | GenBank | rA/X-79(H3N2) | 1 | 8 | No extrapolation |
|  |  |  |  |  |  |  |  |
| YOKO03 | Avian | AB212277 | GenBank | A/duck/Yokohama/aq10/2003(H5N1) | 1 | 1 | No extrapolation |
| YOKO03 | Avian | AB212278 | GenBank | A/duck/Yokohama/aq10/2003(H5N1) | 1 | 2 | No extrapolation |
| YOKO03 | Avian | AB212279 | GenBank | A/duck/Yokohama/aq10/2003(H5N1) | 1 | 3 | No extrapolation |
| YOKO03 | Avian | AB212280 | GenBank | A/duck/Yokohama/aq10/2003(H5N1) | 1 | 4 | No extrapolation |
| YOKO03 | Avian | AB212281 | GenBank | A/duck/Yokohama/aq10/2003(H5N1) | 1 | 5 | No extrapolation |
| YOKO03 | Avian | AB212282 | GenBank | A/duck/Yokohama/aq10/2003(H5N1) | 1 | 6 | No extrapolation |
| YOKO03 | Avian | AB212283 | GenBank | A/duck/Yokohama/aq10/2003(H5N1) | 1 | 7 | No extrapolation |
| YOKO03 | Avian | AB212284 | GenBank | A/duck/Yokohama/aq10/2003(H5N1) | 1 | 8 | No extrapolation |
|  |  |  |  |  |  |  |  |
| YZ232 | Duck | DQ354060 | GenBank | A/duck/Yangzhou/232/2004(H5N1) | 1 | 8 | No extrapolation |
|  |  |  |  |  |  |  |  |
| hvPR8 | Mouse | EF190971 | GenBank | A/hvPR8/1934(H1N1) | 1 | 1 | No extrapolation |
| hvPR8 | Mouse | EF190972 | GenBank | A/hvPR8/1934(H1N1) | 1 | 2 | No extrapolation |
| hvPR8 | Mouse | EF190973 | GenBank | A/hvPR8/1934(H1N1) | 1 | 3 | No extrapolation |
| hvPR8 | Mouse | EF190974 | GenBank | A/hvPR8/1934(H1N1) | 1 | 4 | No extrapolation |
| hvPR8 | Mouse | EF190975 | GenBank | A/hvPR8/1934(H1N1) | 1 | 5 | No extrapolation |
| hvPR8 | Mouse | EF190976 | GenBank | A/hvPR8/1934(H1N1) | 1 | 6 | No extrapolation |
| hvPR8 | Mouse | EF190977 | GenBank | A/hvPR8/1934(H1N1) | 1 | 7 | No extrapolation |
| hvPR8 | Mouse | EF190978 | GenBank | A/hvPR8/1934(H1N1) | 1 | 8 | No extrapolation |
|  |  |  |  |  |  |  |  |
| ma452-G1-1 | Mouse | ID0001 | Corresponding author (3) | A/ma452-G1-1/2014(H5N8) | 1 | 1 | No extrapolation |
| ma452-G1-1 | Mouse | ID0002 | Corresponding author (3) | A/ma452-G1-1/2014(H5N8) | 1 | 2 | No extrapolation |
| ma452-G1-1 | Mouse | ID0003 | Corresponding author (3) | A/ma452-G1-1/2014(H5N8) | 1 | 3 | No extrapolation |
| ma452-G1-1 | Mouse | ID0004 | Corresponding author (3) | A/ma452-G1-1/2014(H5N8) | 1 | 4 | No extrapolation |
| ma452-G1-1 | Mouse | ID0005 | Corresponding author (3) | A/ma452-G1-1/2014(H5N8) | 1 | 5 | No extrapolation |
| ma452-G1-1 | Mouse | ID0006 | Corresponding author (3) | A/ma452-G1-1/2014(H5N8) | 1 | 6 | No extrapolation |
| ma452-G1-1 | Mouse | ID0007 | Corresponding author (3) | A/ma452-G1-1/2014(H5N8) | 1 | 7 | Partial |
| ma452-G1-1 | Mouse | ID0008 | Corresponding author (3) | A/ma452-G1-1/2014(H5N8) | 1 | 8 | Partial |
|  |  |  |  |  |  |  |  |
| ma452-G3-1 | Mouse | ID0009 | Corresponding author (3) | A/ma452-G3-1/2014(H5N8) | 1 | 1 | No extrapolation |
| ma452-G3-1 | Mouse | ID0010 | Corresponding author (3) | A/ma452-G3-1/2014(H5N8) | 1 | 2 | No extrapolation |
| ma452-G3-1 | Mouse | ID0011 | Corresponding author (3) | A/ma452-G3-1/2014(H5N8) | 1 | 3 | No extrapolation |
| ma452-G3-1 | Mouse | ID0012 | Corresponding author (3) | A/ma452-G3-1/2014(H5N8) | 1 | 4 | No extrapolation |
| ma452-G3-1 | Mouse | ID0013 | Corresponding author (3) | A/ma452-G3-1/2014(H5N8) | 1 | 5 | No extrapolation |
| ma452-G3-1 | Mouse | ID0014 | Corresponding author (3) | A/ma452-G3-1/2014(H5N8) | 1 | 6 | No extrapolation |
| ma452-G3-1 | Mouse | ID0015 | Corresponding author (3) | A/ma452-G3-1/2014(H5N8) | 1 | 7 | Partial |
| ma452-G3-1 | Mouse | ID0016 | Corresponding author (3) | A/ma452-G3-1/2014(H5N8) | 1 | 8 | Partial |
|  |  |  |  |  |  |  |  |
| ma452-G3-2 | Mouse | ID0017 | Corresponding author (3) | A/ma452-G3-2/2014(H5N8) | 1 | 1 | No extrapolation |
| ma452-G3-2 | Mouse | ID0018 | Corresponding author (3) | A/ma452-G3-2/2014(H5N8) | 1 | 2 | No extrapolation |
| ma452-G3-2 | Mouse | ID0019 | Corresponding author (3) | A/ma452-G3-2/2014(H5N8) | 1 | 3 | No extrapolation |
| ma452-G3-2 | Mouse | ID0020 | Corresponding author (3) | A/ma452-G3-2/2014(H5N8) | 1 | 4 | No extrapolation |
| ma452-G3-2 | Mouse | ID0021 | Corresponding author (3) | A/ma452-G3-2/2014(H5N8) | 1 | 5 | No extrapolation |
| ma452-G3-2 | Mouse | ID0022 | Corresponding author (3) | A/ma452-G3-2/2014(H5N8) | 1 | 6 | No extrapolation |
| ma452-G3-2 | Mouse | ID0023 | Corresponding author (3) | A/ma452-G3-2/2014(H5N8) | 1 | 7 | Partial |
| ma452-G3-2 | Mouse | ID0024 | Corresponding author (3) | A/ma452-G3-2/2014(H5N8) | 1 | 8 | Partial |
|  |  |  |  |  |  |  |  |
| ma452-G4-1 | Mouse | ID0025 | Corresponding author (3) | A/ma452-G4-1/2014(H5N8) | 1 | 1 | No extrapolation |
| ma452-G4-1 | Mouse | ID0026 | Corresponding author (3) | A/ma452-G4-1/2014(H5N8) | 1 | 2 | No extrapolation |
| ma452-G4-1 | Mouse | ID0027 | Corresponding author (3) | A/ma452-G4-1/2014(H5N8) | 1 | 3 | No extrapolation |
| ma452-G4-1 | Mouse | ID0028 | Corresponding author (3) | A/ma452-G4-1/2014(H5N8) | 1 | 4 | No extrapolation |
| ma452-G4-1 | Mouse | ID0029 | Corresponding author (3) | A/ma452-G4-1/2014(H5N8) | 1 | 5 | No extrapolation |
| ma452-G4-1 | Mouse | ID0030 | Corresponding author (3) | A/ma452-G4-1/2014(H5N8) | 1 | 6 | No extrapolation |
| ma452-G4-1 | Mouse | ID0031 | Corresponding author (3) | A/ma452-G4-1/2014(H5N8) | 1 | 7 | Partial |
| ma452-G4-1 | Mouse | ID0032 | Corresponding author (3) | A/ma452-G4-1/2014(H5N8) | 1 | 8 | Partial |
|  |  |  |  |  |  |  |  |
| ma468-G1-1 | Mouse | ID0033 | Corresponding author (3) | A/ma468-G1-1/2014(H5N8) | 1 | 1 | No extrapolation |
| ma468-G1-1 | Mouse | ID0034 | Corresponding author (3) | A/ma468-G1-1/2014(H5N8) | 1 | 2 | No extrapolation |
| ma468-G1-1 | Mouse | ID0035 | Corresponding author (3) | A/ma468-G1-1/2014(H5N8) | 1 | 3 | No extrapolation |
| ma468-G1-1 | Mouse | ID0036 | Corresponding author (3) | A/ma468-G1-1/2014(H5N8) | 1 | 4 | No extrapolation |
| ma468-G1-1 | Mouse | ID0037 | Corresponding author (3) | A/ma468-G1-1/2014(H5N8) | 1 | 5 | No extrapolation |
| ma468-G1-1 | Mouse | ID0038 | Corresponding author (3) | A/ma468-G1-1/2014(H5N8) | 1 | 6 | No extrapolation |
| ma468-G1-1 | Mouse | ID0039 | Corresponding author (3) | A/ma468-G1-1/2014(H5N8) | 1 | 7 | Partial |
| ma468-G1-1 | Mouse | ID0040 | Corresponding author (3) | A/ma468-G1-1/2014(H5N8) | 1 | 8 | Partial |
|  |  |  |  |  |  |  |  |
| ma468-G1-2 | Mouse | ID0041 | Corresponding author (3) | A/ma468-G1-2/2014(H5N8) | 1 | 1 | No extrapolation |
| ma468-G1-2 | Mouse | ID0042 | Corresponding author (3) | A/ma468-G1-2/2014(H5N8) | 1 | 2 | No extrapolation |
| ma468-G1-2 | Mouse | ID0043 | Corresponding author (3) | A/ma468-G1-2/2014(H5N8) | 1 | 3 | No extrapolation |
| ma468-G1-2 | Mouse | ID0044 | Corresponding author (3) | A/ma468-G1-2/2014(H5N8) | 1 | 4 | No extrapolation |
| ma468-G1-2 | Mouse | ID0045 | Corresponding author (3) | A/ma468-G1-2/2014(H5N8) | 1 | 5 | No extrapolation |
| ma468-G1-2 | Mouse | ID0046 | Corresponding author (3) | A/ma468-G1-2/2014(H5N8) | 1 | 6 | No extrapolation |
| ma468-G1-2 | Mouse | ID0047 | Corresponding author (3) | A/ma468-G1-2/2014(H5N8) | 1 | 7 | Partial |
| ma468-G1-2 | Mouse | ID0048 | Corresponding author (3) | A/ma468-G1-2/2014(H5N8) | 1 | 8 | Partial |
|  |  |  |  |  |  |  |  |
| ma468-G2-1 | Mouse | ID0049 | Corresponding author (3) | A/ma468-G2-1/2014(H5N8) | 1 | 1 | No extrapolation |
| ma468-G2-1 | Mouse | ID0050 | Corresponding author (3) | A/ma468-G2-1/2014(H5N8) | 1 | 2 | No extrapolation |
| ma468-G2-1 | Mouse | ID0051 | Corresponding author (3) | A/ma468-G2-1/2014(H5N8) | 1 | 3 | No extrapolation |
| ma468-G2-1 | Mouse | ID0052 | Corresponding author (3) | A/ma468-G2-1/2014(H5N8) | 1 | 4 | No extrapolation |
| ma468-G2-1 | Mouse | ID0053 | Corresponding author (3) | A/ma468-G2-1/2014(H5N8) | 1 | 5 | No extrapolation |
| ma468-G2-1 | Mouse | ID0054 | Corresponding author (3) | A/ma468-G2-1/2014(H5N8) | 1 | 6 | No extrapolation |
| ma468-G2-1 | Mouse | ID0055 | Corresponding author (3) | A/ma468-G2-1/2014(H5N8) | 1 | 7 | Partial |
| ma468-G2-1 | Mouse | ID0056 | Corresponding author (3) | A/ma468-G2-1/2014(H5N8) | 1 | 8 | Partial |
|  |  |  |  |  |  |  |  |
| ma468-G2-2 | Mouse | ID0057 | Corresponding author (3) | A/ma468-G2-2/2014(H5N8) | 1 | 1 | No extrapolation |
| ma468-G2-2 | Mouse | ID0058 | Corresponding author (3) | A/ma468-G2-2/2014(H5N8) | 1 | 2 | No extrapolation |
| ma468-G2-2 | Mouse | ID0059 | Corresponding author (3) | A/ma468-G2-2/2014(H5N8) | 1 | 3 | No extrapolation |
| ma468-G2-2 | Mouse | ID0060 | Corresponding author (3) | A/ma468-G2-2/2014(H5N8) | 1 | 4 | No extrapolation |
| ma468-G2-2 | Mouse | ID0061 | Corresponding author (3) | A/ma468-G2-2/2014(H5N8) | 1 | 5 | No extrapolation |
| ma468-G2-2 | Mouse | ID0062 | Corresponding author (3) | A/ma468-G2-2/2014(H5N8) | 1 | 6 | No extrapolation |
| ma468-G2-2 | Mouse | ID0063 | Corresponding author (3) | A/ma468-G2-2/2014(H5N8) | 1 | 7 | Partial |
| ma468-G2-2 | Mouse | ID0064 | Corresponding author (3) | A/ma468-G2-2/2014(H5N8) | 1 | 8 | Partial |
|  |  |  |  |  |  |  |  |
| ma468-G2-3 | Mouse | ID0065 | Corresponding author (3) | A/ma468-G2-3/2014(H5N8) | 1 | 1 | No extrapolation |
| ma468-G2-3 | Mouse | ID0066 | Corresponding author (3) | A/ma468-G2-3/2014(H5N8) | 1 | 2 | No extrapolation |
| ma468-G2-3 | Mouse | ID0067 | Corresponding author (3) | A/ma468-G2-3/2014(H5N8) | 1 | 3 | No extrapolation |
| ma468-G2-3 | Mouse | ID0068 | Corresponding author (3) | A/ma468-G2-3/2014(H5N8) | 1 | 4 | No extrapolation |
| ma468-G2-3 | Mouse | ID0069 | Corresponding author (3) | A/ma468-G2-3/2014(H5N8) | 1 | 5 | No extrapolation |
| ma468-G2-3 | Mouse | ID0070 | Corresponding author (3) | A/ma468-G2-3/2014(H5N8) | 1 | 6 | No extrapolation |
| ma468-G2-3 | Mouse | ID0071 | Corresponding author (3) | A/ma468-G2-3/2014(H5N8) | 1 | 7 | Partial |
| ma468-G2-3 | Mouse | ID0072 | Corresponding author (3) | A/ma468-G2-3/2014(H5N8) | 1 | 8 | Partial |
|  |  |  |  |  |  |  |  |
| ma468-G4-2 | Mouse | ID0073 | Corresponding author (3) | A/ma468-G4-2/2014(H5N8) | 1 | 1 | No extrapolation |
| ma468-G4-2 | Mouse | ID0074 | Corresponding author (3) | A/ma468-G4-2/2014(H5N8) | 1 | 2 | No extrapolation |
| ma468-G4-2 | Mouse | ID0075 | Corresponding author (3) | A/ma468-G4-2/2014(H5N8) | 1 | 3 | No extrapolation |
| ma468-G4-2 | Mouse | ID0076 | Corresponding author (3) | A/ma468-G4-2/2014(H5N8) | 1 | 4 | Partial |
| ma468-G4-2 | Mouse | ID0077 | Corresponding author (3) | A/ma468-G4-2/2014(H5N8) | 1 | 5 | No extrapolation |
| ma468-G4-2 | Mouse | ID0078 | Corresponding author (3) | A/ma468-G4-2/2014(H5N8) | 1 | 6 | No extrapolation |
| ma468-G4-2 | Mouse | ID0079 | Corresponding author (3) | A/ma468-G4-2/2014(H5N8) | 1 | 7 | Partial |
| ma468-G4-2 | Mouse | ID0080 | Corresponding author (3) | A/ma468-G4-2/2014(H5N8) | 1 | 8 | Partial |
|  |  |  |  |  |  |  |  |
| maHK68 | Mouse | KY348528 | GenBank | maA/HongKong/1/1968(H3N2) | 1 | 1 | No extrapolation |
| maHK68 | Mouse | KY348529 | GenBank | maA/HongKong/1/1968(H3N2) | 1 | 2 | No extrapolation |
| maHK68 | Mouse | KY348530 | GenBank | maA/HongKong/1/1968(H3N2) | 1 | 3 | No extrapolation |
| maHK68 | Mouse | KY348531 | GenBank | maA/HongKong/1/1968(H3N2) | 1 | 4 | No extrapolation |
| maHK68 | Mouse | KY348532 | GenBank | maA/HongKong/1/1968(H3N2) | 1 | 5 | No extrapolation |
| maHK68 | Mouse | KY348533 | GenBank | maA/HongKong/1/1968(H3N2) | 1 | 6 | No extrapolation |
| maHK68 | Mouse | KY348534 | GenBank | maA/HongKong/1/1968(H3N2) | 1 | 7 | No extrapolation |
| maHK68 | Mouse | KY348535 | GenBank | maA/HongKong/1/1968(H3N2) | 1 | 8 | No extrapolation |
|  |  |  |  |  |  |  |  |
| w81 | Avian | GU361390 | GenBank | A/aquaticbird/Korea/w81/2005(H5N2) | 1 | 1 | No extrapolation |
| w81 | Avian | GU361308 | GenBank | A/aquaticbird/Korea/w81/2005(H5N2) | 1 | 2 | No extrapolation |
| w81 | Avian | GU361350 | GenBank | A/aquaticbird/Korea/w81/2005(H5N2) | 1 | 3 | No extrapolation |
| w81 | Avian | GU361196 | GenBank | A/aquaticbird/Korea/w81/2005(H5N2) | 1 | 4 | No extrapolation |
| w81 | Avian | GU361156 | GenBank | A/aquaticbird/Korea/w81/2005(H5N2) | 1 | 5 | Partial |
| w81 | Avian | GU361235 | GenBank | A/aquaticbird/Korea/w81/2005(H5N2) | 1 | 6 | No extrapolation |
| w81 | Avian | EU819138 | GenBank | A/aquaticbird/Korea/w81/2005(H5N2) | 1 | 7 | No extrapolation |
| w81 | Avian | GU361430 | GenBank | A/aquaticbird/Korea/w81/2005(H5N2) | 1 | 8 | No extrapolation |

**References**

1. Blazejewska P, Koscinski L, Viegas N, Anhlan D, Ludwig S, Schughart K. Pathogenicity of different PR8 influenza A virus variants in mice is determined by both viral and host factors. Virology. 2011;412(1):36-45.

2. Hatesuer B, Bertram S, Mehnert N, Bahgat MM, Nelson PS, Pohlmann S, et al. Tmprss2 is essential for influenza H1N1 virus pathogenesis in mice. PLoS Pathog. 2013;9(12):e1003774.

3. Choi WS, Baek YH, Kwon JJ, Jeong JH, Park SJ, Kim YI, et al. Rapid acquisition of polymorphic virulence markers during adaptation of highly pathogenic avian influenza H5N8 virus in the mouse. Sci Rep. 2017;7:40667.
