# Supplementary material for: Rule-based meta-analysis reveals the major role of PB2 in influencing influenza A virus virulence in mice
Source: BMC Genomics. 2019 Dec 24;20(Suppl 9):973. doi: 10.1186/s12864-019-6295-8 (PMC6929465; doi:10.1186/s12864-019-6295-8)
Supplement: Supplementary file 9 — Additional file 9: Table S5. Mutant and reassortant IAVs generated in this study. [file 12864_2019_6295_MOESM9_ESM.docx]

**Table S5.** Mutant and reassortant IAVs generated in this study. Further information about point mutations being introduced to the IAV genomes can be found in related publications shown in **Table S1** (**Additional file 5**).

| **Genome ID** | **Influenza strain** | **Method for genome derivation** | **Genome sources** |
| --- | --- | --- | --- |
|  |  |  |  |
| CA04MA1Y | A/California/04/MA1/Y/2009(H1N1) | Point mutations | CA04 |
| MA-w81 | mA/aquaticbird/Korea/w81/2005(H5N2) | Point mutations | w81 |
| DKGX22 | A/duck/Guangxi/22/2001(H5N1) | Point mutations | DKGX35 |
| HK486I | rA/HK486/HA-227I(H5N1) | Point mutations | HK486v1 |
| SC35 | A/seal/Massachussetts/1/1980(H7N7) | Point mutations | SC35M |
| laivPR8v1 | A/PR8/LAIV/1934(H1N1) | Point mutations | PR8v1 |
| laivPR8v2 | A/PR8/LAIV/1934(H1N1) | Point mutations | PR8v2 |
| laivPR8v3 | A/PR8/LAIV/1934(H1N1) | Point mutations | PR8v3 |
| PR811Cv1 | A/PR8/11C/1934(H1N1) | Point mutations | PR8v1 |
| PR811Cv2 | A/PR8/11C/1934(H1N1) | Point mutations | PR8v2 |
| PR811Cv3 | A/PR8/11C/1934(H1N1) | Point mutations | PR8v3 |
| laivPR811Cv1 | A/PR8/LAIV11C/1934(H1N1) | Point mutations | PR8v1 |
| laivPR811Cv2 | A/PR8/LAIV11C/1934(H1N1) | Point mutations | PR8v2 |
| laivPR811Cv3 | A/PR8/LAIV11C/1934(H1N1) | Point mutations | PR8v3 |
| PR8MUTv1 | A/PR8/Mutant(H1N1) | Point mutations | PR8v1 |
| PR8MUTv2 | A/PR8/Mutant(H1N1) | Point mutations | PR8v2 |
| PR8MUTv3 | A/PR8/Mutant(H1N1) | Point mutations | PR8v3 |
| CA04MA1I | A/California/04/MA1/I/2009(H1N1) | Point mutations | CA04 |
| CA04MA2I | A/California/04/MA2/I/2009(H1N1) | Point mutations | CA04 |
| TN560MA1I | A/Tennessee/1-560/MA1/I/2009(H1N1) | Point mutations | TN560 |
| TN560MA2I | A/Tennessee/1-560/MA2/I/2009(H1N1) | Point mutations | TN560 |
| NAR1CK1 | A/Narita/1/2009/MDCK(H1N1) | Point mutations | NAR1 |
| NAR1CK15 | A/Narita/1/2009/MDCK15(H1N1) | Point mutations | NAR1 |
| NAR1E1 | A/Narita/1/2009/Egg(H1N1) | Point mutations | NAR1 |
| NAR1E15 | A/Narita/1/2009/Egg15(H1N1) | Point mutations | NAR1 |
| NAR1M15 | A/Narita/1/2009/Mouse15(H1N1) | Point mutations | NAR1 |
| HH05NP100I | A/Hamburg/05/NP100I/2009(H1N1) | Point mutations | HH05 |
| HH05NP133L | A/Hamburg/05/NP133L/2009(H1N1) | Point mutations | HH05 |
| HH05NP373T | A/Hamburg/05/NP373T/2009(H1N1) | Point mutations | HH05 |
| AH1KN | A/AH1/KN(H7N9) | Point mutations | AH1 |
| AH1EN | A/AH1/EN(H7N9) | Point mutations | AH1 |
| CG17PB2-627K | A/chicken/Guangdong/SD008/PB2-627K/2017(H7N9) | Point mutations | CG17 |
| CG2PB2-701N | A/chicken/Guangdong/SD008/PB2-701N/2017(H7N9) | Point mutations | CG17 |
| LN11P1 | mA/duck/Liaoning/LNP1/2011(H5N5) | Point mutations | LN11 |
| LN11P2 | mA/duck/Liaoning/LNP2/2011(H5N5) | Point mutations | LN11 |
| BDKB14QRET | rA/broilerduck/Korea/Buan2-QRET/2014(H5N8) | Point mutations | BDKB14 |
| BDKB14LRET | rA/broilerduck/Korea/Buan2-LRET/2014(H5N8) | Point mutations | BDKB14 |
| SH9L1P5 | mA/Shanghai/SH-9/L1P5/2013(H5N8) | Point mutations | SH-9 |
| SH9L2P5 | mA/Shanghai/SH-9/L2P5/2013(H5N8) | Point mutations | SH-9 |
| SH9L1P2 | mA/Shanghai/SH-9/L1P2/2013(H5N8) | Point mutations | SH-9 |
| SH9L1P4 | mA/Shanghai/SH-9/L1P4/2013(H5N8) | Point mutations | SH-9 |
| CMRR5661 | A/CastillaLaMancha/RR5661/2009(H1N1) | Point mutations | CA04 |
| CMRR5911 | A/CastillaLaMancha/RR5911/2009(H1N1) | Point mutations | CA04 |
| GTH008-NA294R | A/Guangdong/Th008/NA294R/2017(H7N9) | Point mutations | GTH008 |
| GTH008-NA294K | A/Guangdong/Th008/NA294K/2017(H7N9) | Point mutations | GTH008 |
| MA-w81-PB2-627K | rA/MA-w81/PB2-627K(H5N2) | Point mutations | MA-w81 |
| MA-w81-PA-22K | rA/MA-w81/PA-22K(H5N2) | Point mutations | MA-w81 |
| MA-w81-PA-97T | rA/MA-w81/PA-97T(H5N2) | Point mutations | MA-w81 |
| MA-w81-PA-155M | rA/MA-w81/PA-155M(H5N2) | Point mutations | MA-w81 |
| MA-w81-PA-216D | rA/MA-w81/PA-216D(H5N2) | Point mutations | MA-w81 |
| MA-w81-PA-22K-97T | rA/MA-w81/PA-22K-97T(H5N2) | Point mutations | MA-w81 |
| MA-w81-PA-97T-155M | rA/MA-w81/PA-97T-155M(H5N2) | Point mutations | MA-w81 |
| MA-w81-PA-97T-216D | rA/MA-w81/PA-97T-216D(H5N2) | Point mutations | MA-w81 |
| w81-PA-97I | rA/w81/PA-97I(H5N2) | Point mutations | w81 |
| w81-NA-106V-PA-97I | rA/w81/NA-106V/PA-97I(H5N2) | Point mutations | w81 |
| w81-NA-316Y-PA-97I | rA/w81/NA-316Y/PA-97I(H5N2) | Point mutations | w81 |
| w81-NA-436A-PA-97I | rA/w81/NA-436A/PA-97I(H5N2) | Point mutations | w81 |
| w81-PB2-627K | rA/w81/PB2-627K(H5N2) | Point mutations | w81 |
| w81-PB2-627K/PA-97I | rA/w81/PB2-627K/PA-97I(H5N2) | Point mutations | w81 |
| MA-w81-PB2-627K-PA-97T | rA/MA-w81/PB2-627K/PA-97T(H5N2) | Point mutations | MA-w81 |
| CA04-PB2mut | rA/CA04/PB2mut(H1N1) | Point mutations | CA04 |
| CA04-PAmut | rA/CA04/PAmut(H1N1) | Point mutations | CA04 |
| CA04-PB2PAmut | rA/CA04/PB2-PAmut(H1N1) | Point mutations | CA04 |
| CA04-Mmut | rA/CA04/Mmut(H1N1) | Point mutations | CA04 |
| CA04-MPAmut | rA/CA04/M-PAmut(H1N1) | Point mutations | CA04 |
| AH1-PB2-627E | rA/Anhui/1/PB2-627E/2013(H7N9) | Point mutations | AH1 |
| HK486-15mts | rA/HK486/15mts(H5N1) | Point mutations | HK486v1 |
| HK486-5mts | rA/HK486/5mts(H5N1) | Point mutations | HK486v1 |
| HK486-PB2-6mts | rA/HK486/PB2-6mts(H5N1) | Point mutations | HK486v1 |
| HK486-M-3mts | rA/HK486/M-3mts(H5N1) | Point mutations | HK486v1 |
| HK486-NA-3mts | rA/HK486/NA-3mts(H5N1) | Point mutations | HK486v1 |
| HK486-PB1-3mts | rA/HK486/PB1-3mts(H5N1) | Point mutations | HK486v1 |
| HK486-PB2-627K | rA/HK486/PB2-627K(H5N1) | Point mutations | HK486v1 |
| cNL621557 | A/chicken/Netherlands/621557/2003(H7N7) | Point mutations | NL03 |
| maNL621557 | maA/chicken/Netherlands/621557/2003(H7N7) | Point mutations | NL03 |
| DKGX53-M1N30D | A/DKGX53/M1N30D(H5N1) | Point mutations | DKGX53 |
| DKGX53-M1S126G | A/DKGX53/M1S126G(H5N1) | Point mutations | DKGX53 |
| DKGX53-M1T215A | A/DKGX53/M1T215A(H5N1) | Point mutations | DKGX53 |
| DKGX53-M1N30DT215A | A/DKGX53/M1N30DT215A(H5N1) | Point mutations | DKGX53 |
| DKGX53-M1S126GT215A | A/DKGX53/M1S126GT215A(H5N1) | Point mutations | DKGX53 |
| DKFJ01-M1D30NA215T | A/DKFJ01/M1D30NA215T(H5N1) | Point mutations | DKFJ01 |
| DKFJ01-M1D30N | A/DKFJ01/M1D30N(H5N1) | Point mutations | DKFJ01 |
| DKFJ01-M1A215T | A/DKFJ01/M1A215T(H5N1) | Point mutations | DKFJ01 |
| SC35-PB1-13P | rA/SC35/PB1-13P(H7N7) | Point mutations | SC35 |
| SC35-PB1-678N | rA/SC35/PB1-678N(H7N7) | Point mutations | SC35 |
| SC35-PB2-701N | rA/SC35/PB2-701N(H7N7) | Point mutations | SC35 |
| SC35-PB2-714R | rA/SC35/PB2-714R(H7N7) | Point mutations | SC35 |
| SC35-PB2-701N-714R | rA/SC35/PB2-701N-714R(H7N7) | Point mutations | SC35 |
| SC35M-PB1-13L | rA/SC35M/PB1-13L(H7N7) | Point mutations | SC35M |
| SC35M-PB1-678S | rA/SC35M/PB1-678S(H7N7) | Point mutations | SC35M |
| SC35M-PB2-333T | rA/SC35M/PB2-333T(H7N7) | Point mutations | SC35M |
| SC35M-PB2-701D | rA/SC35M/PB2-701D(H7N7) | Point mutations | SC35M |
| SC35M-PB2-714S | rA/SC35M/PB2-714S(H7N7) | Point mutations | SC35M |
| SC18-PB2-E627 | rA/SC18/PB2-E627(H1N1) | Point mutations | SC18 |
| HK483-PB2-675I | rA/HK483/PB2-675I(H5N1) | Point mutations | HK483v1 |
| HK486-PB2-675L | rA/HK486/PB2-675L(H5N1) | Point mutations | HK486v1 |
| HK483-PB2-627E | rA/HK483/PB2-627E(H5N1) | Point mutations | HK483v1 |
| NY1682-PB2-E158G | rA/NY1682/PB2-E158G(H1N1) | Point mutations | NY1682 |
| NY1682-PB2-E627K | rA/NY1682/PB2-E627K(H1N1) | Point mutations | NY1682 |
| NY1682-PB2-D701N | rA/NY1682/PB2-D701N(H1N1) | Point mutations | NY1682 |
| maCA04A | maA/California/04A/2009(H1N1) | Point mutations | CA04 |
| maCA04B | maA/California/04B/2009(H1N1) | Point mutations | CA04 |
| maCA04C | maA/California/04C/2009(H1N1) | Point mutations | CA04 |
| maCA04D | maA/California/04D/2009(H1N1) | Point mutations | CA04 |
| CA04-H274Y | A/California/04/H274Y/2009(H1N1) | Point mutations | CA04 |
| maCA04A-H274Y | maA/California/04A/H274Y/2009(H1N1) | Point mutations | CA04 |
| maCA04B-H274Y | maA/California/04B/H274Y/2009(H1N1) | Point mutations | CA04 |
| maCA04C-H274Y | maA/California/04C/H274Y/2009(H1N1) | Point mutations | CA04 |
| maCA04D-H274Y | maA/California/04D/H274Y/2009(H1N1) | Point mutations | CA04 |
| HK483-HA-cleaved | rA/HK483/HA-cleaved(H5N1) | Point mutations | HK483v1 |
| YOKO03-VAR1 | A/duck/Yokohama/aq10/VAR1/2003(H5N1) | Point mutations | YOKO03 |
| YOKO03-VAR2 | A/duck/Yokohama/aq10/VAR2/2003(H5N1) | Point mutations | YOKO03 |
| TSP83v1 | A/Thailand/SP/83/2004(H5N1) | Point mutations | THAI16v1 |
| TSP83v2 | A/Thailand/SP/83/2004(H5N1) | Point mutations | THAI16v2 |
| DKGX12-NS1-P42S | rA/DKGX12/NS1-P42S(H5N1) | Point mutations | DKGX12 |
| DKGX12-NS1-N48S | rA/DKGX12/NS1-N48S(H5N1) | Point mutations | DKGX12 |
| DKGX12-NS1-S42P | rA/DKGX27/NS1-S42P(H5N1) | Point mutations | DKGX27 |
| DKGX12-NS1-S48N | rA/DKGX27/NS1-S48N(H5N1) | Point mutations | DKGX27 |
| DKGX12-NS1-R38A | rA/DKGX27/NS1-R38A(H5N1) | Point mutations | DKGX27 |
| DKGX12-NS1-K41A | rA/DKGX27/NS1-K41A(H5N1) | Point mutations | DKGX27 |
| DKGX12-NS1-R38A-K41A | rA/DKGX27/NS1-R38A-K41A(H5N1) | Point mutations | DKGX27 |
| REC0001 | rA/SWE1021(1345678)/SWE9706(2)(H1N2) | Recombination | SWE1021 and SWE9706 |
| REC0002 | rA/SWE1021(2)/SWE9706(1345678)(H1N2) | Recombination | SWE1021 and SWE9706 |
| REC0003 | rA/BJ89(123578)/PR8(46)(H1N1) | Recombination | BJ89v1 and PR8v1 |
| REC0004 | rA/BJ89(123578)/PR8(46)(H1N1) | Recombination | BJ89v2 and PR8v1 |
| REC0005 | rA/BJ89(123578)/PR8(46)(H1N1) | Recombination | BJ89v3 and PR8v1 |
| REC0006 | rA/PR8M(1235678)/PR8F(4)(H1N1) | Recombination | PR8M and PR8F |
| REC0007 | rA/PR8vMountSinai(1235678)/BRAZ78(4)(H1N1) | Recombination | PR8vMountSinai and BRAZ78 |
| REC0008 | rA/PR8(1234567)/MI63(8)(H1N1) | Recombination | PR8v1 and MI63 |
| REC0009 | rA/PR8(1234567)/KYG11(8)(H1N1) | Recombination | PR8v1 and KYG11 |
| REC0010 | rA/PR8(123578)/ALB76(46)(H1N1) | Recombination | PR8v1 and ALB76v1 |
| REC0011 | rA/PR8(123578)/ALB76(46)(H1N1) | Recombination | PR8v1 and ALB76v2 |
| REC0012 | rA/PR8(123578)/UKR63(46)(H3N8) | Recombination | PR8v1 and UKR63v1 |
| REC0013 | rA/PR8(123578)/UKR63(46)(H3N8) | Recombination | PR8v1 and UKR63v2 |
| REC0014 | rA/HH05(24678)/HH15(135)(H1N1) | Recombination | HH05 and HH15 |
| REC0015 | rA/HH05(123578)/HH15(46)(H1N1) | Recombination | HH05 and HH15 |
| REC0016 | rA/HH05(1234567)/HH15(8)(H1N1) | Recombination | HH05 and HH15 |
| REC0017 | rA/HH05(1234578)/HH15(6)(H1N1) | Recombination | HH05 and HH15 |
| REC0018 | rA/HH05(1234678)/HH15(5)(H1N1) | Recombination | HH05 and HH15 |
| REC0019 | rA/HH05(1235678)/HH15(4)(H1N1) | Recombination | HH05 and HH15 |
| REC0020 | rA/HH05(1345678)/HH15(3)(H1N1) | Recombination | HH05 and HH15 |
| REC0021 | rA/HH05(2345678)/HH15(1)(H1N1) | Recombination | HH05 and HH15 |
| REC0022 | rA/WSN33(12357)/SD093(468)(H5N1) | Recombination | WSN33 and SD093 |
| REC0023 | rA/WSN33(12357)/SD093(46)/YZ232(8)(H5N1) | Recombination | WSN33, SD093 and YZ232 |
| REC0024 | rA/WSN33(12357)/SD093(468)/NSdel(H5N1) | Recombination | WSN33 and SD093 |
| REC0025 | rA/WSN33(12357)/SD093(46)/YZ232(8)/NSins(H5N1) | Recombination | WSN33, SD093 and YZ232 |
| REC0026 | rA/HK1073(15)/GSHK437(234678)(H5N1) | Recombination | HK1073 and GSHK437 |
| REC0027 | rA/NY312(123578)/ALB76(46)(H1N1) | Recombination | NY312 and ALB76v1 |
| REC0028 | rA/NY312(123578)/SC18(46)(H1N1) | Recombination | NY312 and SC18 |
| REC0029 | rA/PR8(2345678)/PR8M(1)(H1N1) | Recombination | PR8v1 amd PR8M |
| REC0030 | rA/PR8(1234678)/PR8M(5)(H1N1) | Recombination | PR8v1 amd PR8M |
| REC0031 | rA/PR8(1234578)/PR8M(6)(H1N1) | Recombination | PR8v1 amd PR8M |
| REC0032 | rA/PR8(1234567)/PR8M(8)(H1N1) | Recombination | PR8v1 amd PR8M |
| REC0033 | rA/PR8(4678)/PR8M(1235)(H1N1) | Recombination | PR8v1 amd PR8M |
| REC0034 | rA/SC18(45678)/TX91(123)(H1N1) | Recombination | SC18 and TX91v1 |
| REC0035 | rA/SC18(1235678)/TX91(4)(H1N1) | Recombination | SC18 and TX91v1 |
| REC0036 | rA/WSN33(123578)/SC18(46)(H1N1) | Recombination | WSN33 and SC18 |
| REC0037 | rA/M88(123578)/SC18(46)(H1N1) | Recombination | M88 and SC18 |
| REC0038 | rA/M88(123578)/SC18(4)/K173(6)(H1N1) | Recombination | M88 and SC18 |
| REC0039 | rA/M88(123578)/WSN33(46)(H1N1) | Recombination | M88 and WSN33 |
| REC0040 | rA/K173(123578)/SC18(46)(H1N1) | Recombination | K173 and SC18 |
| REC0041 | rA/K173(1235678)/SC18(4)(H1N1) | Recombination | K173 and SC18 |
| REC0042 | rA/K173(123578)/WSN33(46)(H1N1) | Recombination | K173 and WSN33 |
| REC0043 | rA/PR8(1234678)/NL94(5)(H1N1) | Recombination | PR8v1 and NL94 |
| REC0044 | rA/NL602(1235678)/NY18(4)(H1N1) | Recombination | NL602 and NY18 |
| REC0045 | rA/PR8(1235678)/NL03(4)(H7N1) | Recombination | PR8v1 and HA from NL03 |
| REC0046 | rA/H5TK13(1345678)/L969(2)(H5N1) | Recombination | H5TK13 and L969 |
| REC0047 | rA/H5TK13(1234568)/L969(7)(H5N1) | Recombination | H5TK13 and L969 |
| REC0048 | rA/H5TK13(123458)/L969(67)(H5N1) | Recombination | H5TK13 and L969 |
| REC0049 | rA/H5TK13(1234567)/L969(8)(H5N1) | Recombination | H5TK13 and L969 |
| REC0050 | rA/H5TK13(124567)/L969(38)(H5N1) | Recombination | H5TK13 and L969 |
| REC0051 | rA/H5TK13(123457)/L969(68)(H5N1) | Recombination | H5TK13 and L969 |
| REC0052 | rA/CA04(2345678)/HK483(1)(H1N1) | Recombination | CA04 and HK483v1 |
| REC0053 | rA/CA04(1345678)/HK483(2)(H1N1) | Recombination | CA04 and HK483v1 |
| REC0054 | rA/CA04(1245678)/HK483(3)(H1N1) | Recombination | CA04 and HK483v1 |
| REC0055 | rA/CA04(1235678)/HK483(4)(H5N1) | Recombination | CA04 and HK483v1 |
| REC0056 | rA/CA04(1234678)/HK483(5)(H1N1) | Recombination | CA04 and HK483v1 |
| REC0057 | rA/CA04(1234578)/HK483(6)(H1N1) | Recombination | CA04 and HK483v1 |
| REC0058 | rA/CA04(1234568)/HK483(7)(H1N1) | Recombination | CA04 and HK483v1 |
| REC0059 | rA/CA04(1234567)/HK483(8)(H1N1) | Recombination | CA04 and HK483v1 |
| REC0060 | rA/GSH7/673HA-673NA(H5N6) | Recombination | GSH7 and 673 |
| REC0061 | rA/GSH7/673HA-674NA(H5N6) | Recombination | GSH7, 673 and 674 |
| REC0062 | rA/GSH7/674HA-673NA(H5N6) | Recombination | GSH7, 673 and 674 |
| REC0063 | rA/GSH7/674HA-674NA(H5N6) | Recombination | GSH7 and 674 |
| REC0064 | rA/GSH7/LZFNA(H5N2) | Recombination | GSH7 and LZF |
| REC0065 | rA/GSH7/673NA(H5N6) | Recombination | GSH7 and 673 |
| REC0066 | rA/GSH7/674NA(H5N6) | Recombination | GSH7 and 674 |
| REC0067 | rA/GSH7/JS1306NA(H5N8) | Recombination | GSH7 and JS1306 |
| REC0068 | rA/L969(1234578)/L1337(6)(H1N1) | Recombination | L969 and L1337 |
| REC0069 | rA/L969(123478)/L1337(56)(H1N1) | Recombination | L969 and L1337 |
| REC0070 | rA/L969(123578)/L1337(46)(H1N1) | Recombination | L969 and L1337 |
| REC0071 | rA/X-31(123578)/NC99(46)(H1N1) | Recombination | X-31 and NC99v1 |
| REC0072 | rA/X-31(123578)/PAN99(46)(H3N2) | Recombination | X-31 and PAN99v1 |
| REC0073 | rA/X-31(123578)/INA5(46)(H5N1) | Recombination | X-31 and INA5 |
| REC0074 | rA/X-31(123578)/KOR03(46)(H5N1) | Recombination | X-31 and KOR03 |
| REC0075 | rA/X-31(123578)/KOR09(46)(H1N1) | Recombination | X-31 and KOR09 |
| REC0076 | rA/HK483/HK486-PA(H5N1) | Recombination | HK483v1 and HK486v1 |
| REC0077 | rA/HK483/HK486-PB1(H5N1) | Recombination | HK483v1 and HK486v1 |
| REC0078 | rA/HK483/HK486-PB2(H5N1) | Recombination | HK483v1 and HK486v1 |
| REC0079 | rA/HK483/HK486-HA227S(H5N1) | Recombination | HK483v1 and HK486v1 |
| REC0080 | rA/HK483/HK486-NP(H5N1) | Recombination | HK483v1 and HK486v1 |
| REC0081 | rA/HK483/HK486-NA(H5N1) | Recombination | HK483v1 and HK486v1 |
| REC0082 | rA/HK483/HK486-M(H5N1) | Recombination | HK483v1 and HK486v1 |
| REC0083 | rA/HK483/HK486-NS(H5N1) | Recombination | HK483v1 and HK486v1 |
| REC0084 | rA/HK486-HA227S/HK483-PA(H5N1) | Recombination | HK483v1 and HK486v1 |
| REC0085 | rA/HK486-HA227S/HK483-PB1(H5N1) | Recombination | HK483v1 and HK486v1 |
| REC0086 | rA/HK486-HA227S/HK483-PB2(H5N1) | Recombination | HK483v1 and HK486v1 |
| REC0087 | rA/HK486-HA227S/HK483-NP(H5N1) | Recombination | HK483v1 and HK486v1 |
| REC0088 | rA/HK486-HA227S/HK483-NA(H5N1) | Recombination | HK483v1 and HK486v1 |
| REC0089 | rA/HK486-HA227S/HK483-M(H5N1) | Recombination | HK483v1 and HK486v1 |
| REC0090 | rA/HK486-HA227S/HK483-NS(H5N1) | Recombination | HK483v1 and HK486v1 |
| REC0091 | rA/HK486/HK483-HA(H5N1) | Recombination | HK483v1 and HK486v1 |
| REC0092 | rA/HK486(123578)/HK483(46)(H5N1) | Recombination | HK483v1 and HK486v1 |
| REC0093 | rA/HK486(1235678)/HK483(4)(H5N1) | Recombination | HK483v1 and HK486v1 |
| REC0094 | rA/HK486(1234578)/HK483(6)(H5N1) | Recombination | HK483v1 and HK486v1 |
| REC0095 | rA/HK483(2345678)/HK486(1)(H5N1) | Recombination | HK483v1 and HK486v1 |
| REC0096 | rA/DKGX53(2345678)/DKFJ01(1)(H5N1) | Recombination | DKGX53 and DKFJ01 |
| REC0097 | rA/DKGX53(1345678)/DKFJ01(2)(H5N1) | Recombination | DKGX53 and DKFJ01 |
| REC0098 | rA/DKGX53(1245678)/DKFJ01(3)(H5N1) | Recombination | DKGX53 and DKFJ01 |
| REC0099 | rA/DKGX53(1235678)/DKFJ01(4)(H5N1) | Recombination | DKGX53 and DKFJ01 |
| REC0100 | rA/DKGX53(1234678)/DKFJ01(5)(H5N1) | Recombination | DKGX53 and DKFJ01 |
| REC0101 | rA/DKGX53(1234578)/DKFJ01(6)(H5N1) | Recombination | DKGX53 and DKFJ01 |
| REC0102 | rA/DKGX53(1234568)/DKFJ01(7)(H5N1) | Recombination | DKGX53 and DKFJ01 |
| REC0103 | rA/DKGX53(1234567)/DKFJ01(8)(H5N1) | Recombination | DKGX53 and DKFJ01 |
| REC0104 | rA/DKFJ01(2345678)/DKGX53(1)(H5N1) | Recombination | DKGX53 and DKFJ01 |
| REC0105 | rA/DKFJ01(1345678)/DKGX53(2)(H5N1) | Recombination | DKGX53 and DKFJ01 |
| REC0106 | rA/DKFJ01(1245678)/DKGX53(3)(H5N1) | Recombination | DKGX53 and DKFJ01 |
| REC0107 | rA/DKFJ01(1235678)/DKGX53(4)(H5N1) | Recombination | DKGX53 and DKFJ01 |
| REC0108 | rA/DKFJ01(1234678)/DKGX53(5)(H5N1) | Recombination | DKGX53 and DKFJ01 |
| REC0109 | rA/DKFJ01(1234578)/DKGX53(6)(H5N1) | Recombination | DKGX53 and DKFJ01 |
| REC0110 | rA/DKFJ01(1234568)/DKGX53(7)(H5N1) | Recombination | DKGX53 and DKFJ01 |
| REC0111 | rA/DKFJ01(1234567)/DKGX53(8)(H5N1) | Recombination | DKGX53 and DKFJ01 |
| REC0112 | rA/SH1023(2345678)/AH1(1)(H9N2) | Recombination | SH1023 and AH1 |
| REC0113 | rA/SH1023(1345678)/AH1(2)(H9N2) | Recombination | SH1023 and AH1 |
| REC0114 | rA/SH1023(1245678)/AH1(3)(H9N2) | Recombination | SH1023 and AH1 |
| REC0115 | rA/SH1023(1234678)/AH1(5)(H9N2) | Recombination | SH1023 and AH1 |
| REC0116 | rA/SH1023(1234568)/AH1(7)(H9N2) | Recombination | SH1023 and AH1 |
| REC0117 | rA/SH1023(1234567)/AH1(8)(H9N2) | Recombination | SH1023 and AH1 |
| REC0118 | rA/DKGX12(2345678)/DKGX27(1)(H5N1) | Recombination | DKGX12 and DKGX27 |
| REC0119 | rA/DKGX12(1245678)/DKGX27(3)(H5N1) | Recombination | DKGX12 and DKGX27 |
| REC0120 | rA/DKGX12(1235678)/DKGX27(4)(H5N1) | Recombination | DKGX12 and DKGX27 |
| REC0121 | rA/DKGX12(1234678)/DKGX27(5)(H5N1) | Recombination | DKGX12 and DKGX27 |
| REC0122 | rA/DKGX12(1234567)/DKGX27(8)(H5N1) | Recombination | DKGX12 and DKGX27 |
| REC0123 | rA/DKGX27(2345678)/DKGX12(1)(H5N1) | Recombination | DKGX12 and DKGX27 |
| REC0124 | rA/DKGX27(1245678)/DKGX12(3)(H5N1) | Recombination | DKGX12 and DKGX27 |
| REC0125 | rA/DKGX27(1235678)/DKGX12(4)(H5N1) | Recombination | DKGX12 and DKGX27 |
| REC0126 | rA/DKGX27(1234678)/DKGX12(5)(H5N1) | Recombination | DKGX12 and DKGX27 |
| REC0127 | rA/DKGX27(1234567)/DKGX12(8)(H5N1) | Recombination | DKGX12 and DKGX27 |
| REC0128 | rA/SC18(2345678)/OH175(1)(H1N1) | Recombination | SC18 and OH175 |
| REC0129 | rA/SC18(1345678)/OH175(2)(H1N1) | Recombination | SC18 and OH175 |
| REC0130 | rA/SC18(1245678)/OH175(3)(H1N1) | Recombination | SC18 and OH175 |
| REC0131 | rA/SC18(1235678)/OH265(4)(H1N1) | Recombination | SC18 and OH265 |
| REC0132 | rA/SC18(1234678)/OH175(5)(H1N1) | Recombination | SC18 and OH175 |
| REC0133 | rA/SC18(1234578)/OH175(6)(H1N1) | Recombination | SC18 and OH175 |
| REC0134 | rA/SC18(1234568)/OH175(7)(H1N1) | Recombination | SC18 and OH175 |
| REC0135 | rA/SC18(1234567)/OH175(8)(H1N1) | Recombination | SC18 and OH175 |
| REC0136 | rA/OH175(1235678)/OH265(4)(H1N1) | Recombination | OH175 and OH265 |
| REC0137 | rA/CA04(1235678)/CA04MA1Y(4)(H1N1) | Recombination | CA04 and CA04MA1Y |
| REC0138 | rA/CA04(125678)/CA04MA1Y(34)(H1N1) | Recombination | CA04 and CA04MA1Y |
| REC0139 | rA/CA04(123678)/CA04MA1Y(45)(H1N1) | Recombination | CA04 and CA04MA1Y |
| REC0140 | rA/CA04(12678)/CA04MA1Y(345)(H1N1) | Recombination | CA04 and CA04MA1Y |
| REC0141 | rA/NL602(1235678)/CA04MA1Y(4)(H1N1) | Recombination | CA04 and CA04MA1Y |
| REC0142 | rA/MA-w81(H5N2) | Point mutations | MA-w81 |
| REC0143 | rA/MA-w81(2345678)/w81(1)(H5N2) | Recombination | w81 and MA-w81 |
| REC0144 | rA/MA-w81(2345678)/w81(1)(H5N2) | Recombination | w81 and MA-w81 |
| REC0145 | rA/MA-w81(1345678)/w81(2)(H5N2) | Recombination | w81 and MA-w81 |
| REC0146 | rA/MA-w81(1245678)/w81(3)(H5N2) | Recombination | w81 and MA-w81 |
| REC0147 | rA/MA-w81(1235678)/w81(4)(H5N2) | Recombination | w81 and MA-w81 |
| REC0148 | rA/MA-w81(1234578)/w81(6)(H5N2) | Recombination | w81 and MA-w81 |
| REC0149 | rA/MA-w81(1234568)/w81(7)(H5N2) | Recombination | w81 and MA-w81 |
| REC0150 | rA/w81(2345678)/MA-w81(1)(H5N2) | Recombination | w81 and MA-w81 |
| REC0151 | rA/w81(1345678)/MA-w81(2)(H5N2) | Recombination | w81 and MA-w81 |
| REC0152 | rA/w81(1245678)/MA-w81(3)(H5N2) | Recombination | w81 and MA-w81 |
| REC0153 | rA/w81(1235678)/MA-w81(4)(H5N2) | Recombination | w81 and MA-w81 |
| REC0154 | rA/w81(1234578)/MA-w81(6)(H5N2) | Recombination | w81 and MA-w81 |
| REC0155 | rA/w81(1234568)/MA-w81(7)(H5N2) | Recombination | w81 and MA-w81 |
| REC0156 | rA/w81(124578)/MA-w81(36)(H5N2) | Recombination | w81 and MA-w81 |
| REC0157 | rA/SC35(2345678)/SC35M(1)(H7N7) | Recombination | SC35 and SC35M |
| REC0158 | rA/SC35(1345678)/SC35M(2)(H7N7) | Recombination | SC35 and SC35M |
| REC0159 | rA/SC35(1245678)/SC35M(3)(H7N7) | Recombination | SC35 and SC35M |
| REC0160 | rA/SC35(1235678)/SC35M(4)(H7N7) | Recombination | SC35 and SC35M |
| REC0161 | rA/SC35(1234678)/SC35M(5)(H7N7) | Recombination | SC35 and SC35M |
| REC0162 | rA/SC35(1234578)/SC35M(6)(H7N7) | Recombination | SC35 and SC35M |
| REC0163 | rA/DKGX22(2345678)/DKGX35(1)(H5N1) | Recombination | DKGX22 and DKGX35 |
| REC0164 | rA/DKGX22(1345678)/DKGX35(2)(H5N1) | Recombination | DKGX22 and DKGX35 |
| REC0165 | rA/DKGX22(1245678)/DKGX35(3)(H5N1) | Recombination | DKGX22 and DKGX35 |
| REC0166 | rA/DKGX22(1235678)/DKGX35(4)(H5N1) | Recombination | DKGX22 and DKGX35 |
| REC0167 | rA/DKGX22(1234678)/DKGX35(5)(H5N1) | Recombination | DKGX22 and DKGX35 |
| REC0168 | rA/DKGX22(1234578)/DKGX35(6)(H5N1) | Recombination | DKGX22 and DKGX35 |
| REC0169 | rA/DKGX22(1234568)/DKGX35(7)(H5N1) | Recombination | DKGX22 and DKGX35 |
| REC0170 | rA/DKGX22(1234567)/DKGX35(8)(H5N1) | Recombination | DKGX22 and DKGX35 |
| REC0171 | rA/DKGX35(2345678)/DKGX22(1)(H5N1) | Recombination | DKGX22 and DKGX35 |
| REC0172 | rA/DKGX35(1345678)/DKGX22(2)(H5N1) | Recombination | DKGX22 and DKGX35 |
| REC0173 | rA/DKGX35(1245678)/DKGX22(3)(H5N1) | Recombination | DKGX22 and DKGX35 |
| REC0174 | rA/DKGX35(1235678)/DKGX22(4)(H5N1) | Recombination | DKGX22 and DKGX35 |
| REC0175 | rA/DKGX35(1234678)/DKGX22(5)(H5N1) | Recombination | DKGX22 and DKGX35 |
| REC0176 | rA/DKGX35(1234578)/DKGX22(6)(H5N1) | Recombination | DKGX22 and DKGX35 |
| REC0177 | rA/DKGX35(1234568)/DKGX22(7)(H5N1) | Recombination | DKGX22 and DKGX35 |
| REC0178 | rA/DKGX35(1234567)/DKGX22(8)(H5N1) | Recombination | DKGX22 and DKGX35 |
| REC0179 | rA/HK483/HK486-HA227I(H5N1) | Recombination | HK483v1 and HK486I |
| REC0180 | rA/HK486-HA227I/HK483-PA(H5N1) | Recombination | HK483v1 and HK486I |
| REC0181 | rA/HK486-HA227I/HK483-PB1(H5N1) | Recombination | HK483v1 and HK486I |
| REC0182 | rA/HK486-HA227I/HK483-PB2(H5N1) | Recombination | HK483v1 and HK486I |
| REC0183 | rA/HK486-HA227I/HK483-NP(H5N1) | Recombination | HK483v1 and HK486I |
| REC0184 | rA/HK486-HA227I/HK483-NA(H5N1) | Recombination | HK483v1 and HK486I |
| REC0185 | rA/HK486-HA227I/HK483-M(H5N1) | Recombination | HK483v1 and HK486I |
| REC0186 | rA/HK486-HA227I/HK483-NS(H5N1) | Recombination | HK483v1 and HK486I |
| RECMUT0001 | rA/MA-w81/w81PA-97I(H5N2) | Recombination with (additional) point mutations | w81 and MA-w81 |
| RECMUT0002 | rA/w81/PA-97I/MA-w81NA(H5N2) | Recombination with (additional) point mutations | w81 and MA-w81 |
| RECMUT0003 | rA/PR8(1234678)/NL94(5)/G384R(H1N1) | Recombination with (additional) point mutations | PR8v1 and NL94 |
| RECMUT0004 | rA/PR8(1235678)/CH1(4)(H1N1) | Recombination with (additional) point mutations | PR8v1 and CH1 (consensus H1) |
| RECMUT0005 | rA/NY312(123578)/mutALB76(46)(H1N1) | Recombination with (additional) point mutations | NY312 and ALB76v1 |
| RECMUT0006 | rA/NY312(123578)/SC18(46)/HA-D225G(H1N1) | Recombination with (additional) point mutations | NY312 and SC18 |
| RECMUT0007 | rA/NY312(123578)/SC18(46)/HA-D190E-D225G(H1N1) | Recombination with (additional) point mutations | NY312 and SC18 |
| RECMUT0008 | rA/SH1023(2345678)/AH1(1)-627E(H9N2) | Recombination with (additional) point mutations | SH1023 and AH1 |
| RECMUT0009 | rA/WSN33(12357)/SD093(468)/NS1-E97D(H5N1) | Recombination with (additional) point mutations | WSN33 and SD093 |
| RECMUT0010 | rA/WSN33(12357)/SD093(468)/NSdel/NS1-E92D(H5N1) | Recombination with (additional) point mutations | WSN33 and SD093 |
| RECMUT0011 | rA/SC18/OH175-PB2-K627(H1N1) | Recombination with (additional) point mutations | SC18 and OH175 |
| RECMUT0012 | rA/SC35/PB2-701N/SC35M(5)(H7N7) | Recombination with (additional) point mutations | SC35 and SC35M |
| RECMUT0013 | rA/SC35/PB2-714R/SC35M(5)(H7N7) | Recombination with (additional) point mutations | SC35 and SC35M |
| RECMUT0014 | rA/SC35/PB2-701N-714R/SC35M(5)(H7N7) | Recombination with (additional) point mutations | SC35 and SC35M |
