# Supplementary material for: Rule-based meta-analysis reveals the major role of PB2 in influencing influenza A virus virulence in mice
Source: BMC Genomics. 2019 Dec 24;20(Suppl 9):973. doi: 10.1186/s12864-019-6295-8 (PMC6929465; doi:10.1186/s12864-019-6295-8)
Supplement: Supplementary file 10 — Additional file 10: Table S6. GISAID acknowledgement table for sequences used in this study. [file 12864_2019_6295_MOESM10_ESM.docx]

**Table S6.** GISAID acknowledgement table for sequences used in this study.

| *We acknowledge the authors, originating and submitting laboratories of the sequences from GISAID’s EpiFlu*™ Database on which this research is based. The list is detailed below. | | | | | | | |
| --- | --- | --- | --- | --- | --- | --- | --- |
| *All submitters of data may be contacted directly via the GISAID website* ***www.gisaid.org*** | | | | | | | |
| **Segment ID** | **Segment** | **Country** | **Collection date** | **Isolate name** | **Originating Lab** | **Submitting Lab** | **Authors** |
|  |  |  |  |  |  |  |  |
| EPI228459 | HA | Belgium | 2004-Jan-01 | A/crested eagle/Belgium/01/2004 |  | Import from public-domain | Van Borm,S.; Lambrecht,B.; Boschmans,M.; van den Berg,T. |
| EPI229697 | NA | Belgium | 2004-Jan-01 | A/crested eagle/Belgium/01/2004 |  | Import from public-domain | Van Borm,S.; Lambrecht,B.; Boschmans,M.; van den Berg,T. |
| EPI229698 | HA | Belgium | 2004-Jan-01 | A/crested eagle/Belgium/01/2004 |  | Import from public-domain | Van Borm,S.; Lambrecht,B.; Boschmans,M.; van den Berg,T. |
| EPI229699 | MP | Belgium | 2004-Jan-01 | A/crested eagle/Belgium/01/2004 |  | Import from public-domain | Van Borm,S.; Lambrecht,B.; Boschmans,M.; van den Berg,T. |
| EPI229700 | PB1 | Belgium | 2004-Jan-01 | A/crested eagle/Belgium/01/2004 |  | Import from public-domain | Van Borm,S.; Lambrecht,B.; Boschmans,M.; van den Berg,T. |
| EPI229701 | PA | Belgium | 2004-Jan-01 | A/crested eagle/Belgium/01/2004 |  | Import from public-domain | Van Borm,S.; Lambrecht,B.; Boschmans,M.; van den Berg,T. |
| EPI229702 | NP | Belgium | 2004-Jan-01 | A/crested eagle/Belgium/01/2004 |  | Import from public-domain | Van Borm,S.; Lambrecht,B.; Boschmans,M.; van den Berg,T. |
| EPI229703 | NS | Belgium | 2004-Jan-01 | A/crested eagle/Belgium/01/2004 |  | Import from public-domain | Van Borm,S.; Lambrecht,B.; Boschmans,M.; van den Berg,T. |
| EPI229704 | PB2 | Belgium | 2004-Jan-01 | A/crested eagle/Belgium/01/2004 |  | Import from public-domain | Van Borm,S.; Lambrecht,B.; Boschmans,M.; van den Berg,T. |
| EPI229712 | HA | Belgium | 2004-Jan-01 | A/crested eagle/Belgium/01/2004 |  | Import from public-domain | Van Borm,S.; Lambrecht,B.; Boschmans,M.; van den Berg,T. |
| EPI229713 | HA | Belgium | 2004-Jan-01 | A/crested eagle/Belgium/01/2004 |  | Import from public-domain | Van Borm,S.; Lambrecht,B.; Boschmans,M.; van den Berg,T. |
| EPI1215861 | PA | China | 2013-Sep-20 | A/goose/Guangdong/SH7/2013(H5N1) | South China Agricultural University | South China Agricultural University | Yuandi,Yu;Zaoyue,Zhang;Wenbao,Qi;Ming,Liao |
| EPI1215864 | NS | China | 2013-Sep-20 | A/goose/Guangdong/SH7/2013(H5N1) | South China Agricultural University | South China Agricultural University | Yuandi,Yu;Zaoyue,Zhang;Wenbao,Qi;Ming,Liao |
| EPI1215859 | PB2 | China | 2013-Sep-20 | A/goose/Guangdong/SH7/2013(H5N1) | South China Agricultural University | South China Agricultural University | Yuandi,Yu;Zaoyue,Zhang;Wenbao,Qi;Ming,Liao |
| EPI1215860 | PB1 | China | 2013-Sep-20 | A/goose/Guangdong/SH7/2013(H5N1) | South China Agricultural University | South China Agricultural University | Yuandi,Yu;Zaoyue,Zhang;Wenbao,Qi;Ming,Liao |
| EPI1215865 | MP | China | 2013-Sep-20 | A/goose/Guangdong/SH7/2013(H5N1) | South China Agricultural University | South China Agricultural University | Yuandi,Yu;Zaoyue,Zhang;Wenbao,Qi;Ming,Liao |
| EPI1215862 | HA | China | 2013-Sep-20 | A/goose/Guangdong/SH7/2013(H5N1) | South China Agricultural University | South China Agricultural University | Yuandi,Yu;Zaoyue,Zhang;Wenbao,Qi;Ming,Liao |
| EPI1215863 | NP | China | 2013-Sep-20 | A/goose/Guangdong/SH7/2013(H5N1) | South China Agricultural University | South China Agricultural University | Yuandi,Yu;Zaoyue,Zhang;Wenbao,Qi;Ming,Liao |
| EPI980838 | NA | China | 2013-Sep-20 | A/goose/Guangdong/SH7/2013(H5N1) | South China Agricultural University | South China Agricultural University | Yuandi,Yu;Zaoyue,Zhang;Wenbao,Qi;Ming,Liao |
| EPI1208376 | HA | China | 2014-Mar-30 | A/goose/Guangdong/674/2014(H5N6) | South China Agricultural University | South China Agricultural University | Yuandi,Yu;Zaoyue,Zhang;Wenbao,Qi;Ming,Liao |
| EPI980835 | NA | China | 2014-Mar-30 | A/goose/Guangdong/674/2014(H5N6) | South China Agricultural University | South China Agricultural University | Yuandi,Yu;Zaoyue,Zhang;Wenbao,Qi;Ming,Liao |
| EPI319936 | NA | Netherlands | 2003-Jan-01 | A/Netherlands/230/03 | National Institute for Public Health and the Environment (RIVM) | National Institute for Public Health and the Environment (RIVM) | Meijer,A; Jonges,M; Fouchier,RAM |
| EPI319937 | HA | Netherlands | 2003-Jan-01 | A/Netherlands/230/03 | National Institute for Public Health and the Environment (RIVM) | National Institute for Public Health and the Environment (RIVM) | Meijer,A; Jonges,M; Fouchier,RAM |
| EPI319935 | PB2 | Netherlands | 2003-Jan-01 | A/Netherlands/230/03 | National Institute for Public Health and the Environment (RIVM) | National Institute for Public Health and the Environment (RIVM) | Meijer,A; Jonges,M; Fouchier,RAM |
| EPI926821 | PA | China | 2017-Feb-01 | A/Guangdong/Th005/2017_H7N9 | CAS Key Laboratory of Pathogenic Microbiology and Immunology, Institute of Microbiology, Chinese Academy of Sciences | Institute of Microbiology, Chinese Academy of Sciences | Yuhai, Bi |
| EPI926822 | PB2 | China | 2017-Feb-01 | A/Guangdong/Th005/2017_H7N9 | CAS Key Laboratory of Pathogenic Microbiology and Immunology, Institute of Microbiology, Chinese Academy of Sciences | Institute of Microbiology, Chinese Academy of Sciences | Yuhai, Bi |
| EPI926823 | PB1 | China | 2017-Feb-01 | A/Guangdong/Th005/2017_H7N9 | CAS Key Laboratory of Pathogenic Microbiology and Immunology, Institute of Microbiology, Chinese Academy of Sciences | Institute of Microbiology, Chinese Academy of Sciences | Yuhai, Bi |
| EPI926818 | NP | China | 2017-Feb-01 | A/Guangdong/Th005/2017_H7N9 | CAS Key Laboratory of Pathogenic Microbiology and Immunology, Institute of Microbiology, Chinese Academy of Sciences | Institute of Microbiology, Chinese Academy of Sciences | Yuhai, Bi |
| EPI926819 | NS | China | 2017-Feb-01 | A/Guangdong/Th005/2017_H7N9 | CAS Key Laboratory of Pathogenic Microbiology and Immunology, Institute of Microbiology, Chinese Academy of Sciences | Institute of Microbiology, Chinese Academy of Sciences | Yuhai, Bi |
| EPI926820 | MP | China | 2017-Feb-01 | A/Guangdong/Th005/2017_H7N9 | CAS Key Laboratory of Pathogenic Microbiology and Immunology, Institute of Microbiology, Chinese Academy of Sciences | Institute of Microbiology, Chinese Academy of Sciences | Yuhai, Bi |
| EPI926825 | HA | China | 2017-Feb-01 | A/Guangdong/Th005/2017_H7N9 | CAS Key Laboratory of Pathogenic Microbiology and Immunology, Institute of Microbiology, Chinese Academy of Sciences | Institute of Microbiology, Chinese Academy of Sciences | Yuhai, Bi |
| EPI926824 | NA | China | 2017-Feb-01 | A/Guangdong/Th005/2017_H7N9 | CAS Key Laboratory of Pathogenic Microbiology and Immunology, Institute of Microbiology, Chinese Academy of Sciences | Institute of Microbiology, Chinese Academy of Sciences | Yuhai, Bi |
| EPI926810 | NP | China | 2017-Jan-04 | A/Guangdong/Th008/2017_H7N9 | CAS Key Laboratory of Pathogenic Microbiology and Immunology, Institute of Microbiology, Chinese Academy of Sciences | Institute of Microbiology, Chinese Academy of Sciences | Yuhai, Bi |
| EPI926811 | NS | China | 2017-Jan-04 | A/Guangdong/Th008/2017_H7N9 | CAS Key Laboratory of Pathogenic Microbiology and Immunology, Institute of Microbiology, Chinese Academy of Sciences | Institute of Microbiology, Chinese Academy of Sciences | Yuhai, Bi |
| EPI926812 | MP | China | 2017-Jan-04 | A/Guangdong/Th008/2017_H7N9 | CAS Key Laboratory of Pathogenic Microbiology and Immunology, Institute of Microbiology, Chinese Academy of Sciences | Institute of Microbiology, Chinese Academy of Sciences | Yuhai, Bi |
| EPI926813 | PA | China | 2017-Jan-04 | A/Guangdong/Th008/2017_H7N9 | CAS Key Laboratory of Pathogenic Microbiology and Immunology, Institute of Microbiology, Chinese Academy of Sciences | Institute of Microbiology, Chinese Academy of Sciences | Yuhai, Bi |
| EPI926814 | PB2 | China | 2017-Jan-04 | A/Guangdong/Th008/2017_H7N9 | CAS Key Laboratory of Pathogenic Microbiology and Immunology, Institute of Microbiology, Chinese Academy of Sciences | Institute of Microbiology, Chinese Academy of Sciences | Yuhai, Bi |
| EPI926815 | PB1 | China | 2017-Jan-04 | A/Guangdong/Th008/2017_H7N9 | CAS Key Laboratory of Pathogenic Microbiology and Immunology, Institute of Microbiology, Chinese Academy of Sciences | Institute of Microbiology, Chinese Academy of Sciences | Yuhai, Bi |
| EPI926816 | NA | China | 2017-Jan-04 | A/Guangdong/Th008/2017_H7N9 | CAS Key Laboratory of Pathogenic Microbiology and Immunology, Institute of Microbiology, Chinese Academy of Sciences | Institute of Microbiology, Chinese Academy of Sciences | Yuhai, Bi |
| EPI926817 | HA | China | 2017-Jan-04 | A/Guangdong/Th008/2017_H7N9 | CAS Key Laboratory of Pathogenic Microbiology and Immunology, Institute of Microbiology, Chinese Academy of Sciences | Institute of Microbiology, Chinese Academy of Sciences | Yuhai, Bi |
| EPI272297 | HA | Korea, Republic of | 2009-Oct-06 | A/Seoul/Y-01/2009 |  | Import from public-domain | Byun,Y.; Lee,K.; Seong,B. |
| EPI272298 | NA | Korea, Republic of | 2009-Oct-06 | A/Seoul/Y-01/2009 |  | Import from public-domain | Byun,Y.; Lee,K.; Seong,B. |
| EPI919592 | NP | China | 2017-Jan-12 | A/Guangdong/17SF006/2017 |  | WHO Chinese National Influenza Center |  |
| EPI919593 | NS | China | 2017-Jan-12 | A/Guangdong/17SF006/2017 |  | WHO Chinese National Influenza Center |  |
| EPI919594 | MP | China | 2017-Jan-12 | A/Guangdong/17SF006/2017 |  | WHO Chinese National Influenza Center |  |
| EPI919595 | PA | China | 2017-Jan-12 | A/Guangdong/17SF006/2017 |  | WHO Chinese National Influenza Center |  |
| EPI919596 | PB2 | China | 2017-Jan-12 | A/Guangdong/17SF006/2017 |  | WHO Chinese National Influenza Center |  |
| EPI919597 | PB1 | China | 2017-Jan-12 | A/Guangdong/17SF006/2017 |  | WHO Chinese National Influenza Center |  |
| EPI919598 | NA | China | 2017-Jan-12 | A/Guangdong/17SF006/2017 |  | WHO Chinese National Influenza Center |  |
| EPI919599 | HA | China | 2017-Jan-12 | A/Guangdong/17SF006/2017 |  | WHO Chinese National Influenza Center |  |
| EPI691394 | PA | China | 2015-Jul-02 | A/Hunan/42443/2015 |  | WHO Chinese National Influenza Center | Tan, Minju; Li, Xiaodan; Chen, Wenbing; Wang, Dayan |
| EPI691392 | PB2 | China | 2015-Jul-02 | A/Hunan/42443/2015 |  | WHO Chinese National Influenza Center | Tan, Minju; Li, Xiaodan; Chen, Wenbing; Wang, Dayan |
| EPI691397 | NA | China | 2015-Jul-02 | A/Hunan/42443/2015 |  | WHO Chinese National Influenza Center | Tan, Minju; Li, Xiaodan; Chen, Wenbing; Wang, Dayan |
| EPI691399 | NS | China | 2015-Jul-02 | A/Hunan/42443/2015 |  | WHO Chinese National Influenza Center | Tan, Minju; Li, Xiaodan; Chen, Wenbing; Wang, Dayan |
| EPI691393 | PB1 | China | 2015-Jul-02 | A/Hunan/42443/2015 |  | WHO Chinese National Influenza Center | Tan, Minju; Li, Xiaodan; Chen, Wenbing; Wang, Dayan |
| EPI691396 | NP | China | 2015-Jul-02 | A/Hunan/42443/2015 |  | WHO Chinese National Influenza Center | Tan, Minju; Li, Xiaodan; Chen, Wenbing; Wang, Dayan |
| EPI691395 | HA | China | 2015-Jul-02 | A/Hunan/42443/2015 |  | WHO Chinese National Influenza Center | Tan, Minju; Li, Xiaodan; Chen, Wenbing; Wang, Dayan |
| EPI691398 | MP | China | 2015-Jul-02 | A/Hunan/42443/2015 |  | WHO Chinese National Influenza Center | Tan, Minju; Li, Xiaodan; Chen, Wenbing; Wang, Dayan |
| EPI439503 | PA | China | 2013-Mar-20 | A/Anhui/1/2013 |  | WHO Chinese National Influenza Center |  |
| EPI439504 | PB2 | China | 2013-Mar-20 | A/Anhui/1/2013 |  | WHO Chinese National Influenza Center |  |
| EPI439505 | NP | China | 2013-Mar-20 | A/Anhui/1/2013 |  | WHO Chinese National Influenza Center |  |
| EPI439508 | PB1 | China | 2013-Mar-20 | A/Anhui/1/2013 |  | WHO Chinese National Influenza Center |  |
| EPI439506 | MP | China | 2013-Mar-20 | A/Anhui/1/2013 |  | WHO Chinese National Influenza Center |  |
| EPI439507 | HA | China | 2013-Mar-20 | A/Anhui/1/2013 |  | WHO Chinese National Influenza Center |  |
| EPI439509 | NA | China | 2013-Mar-20 | A/Anhui/1/2013 |  | WHO Chinese National Influenza Center |  |
| EPI439510 | NS | China | 2013-Mar-20 | A/Anhui/1/2013 |  | WHO Chinese National Influenza Center |  |
| EPI439488 | PB2 | China | 2013-Feb-26 | A/Shanghai/1/2013 |  | WHO Chinese National Influenza Center |  |
| EPI439493 | MP | China | 2013-Feb-26 | A/Shanghai/1/2013 |  | WHO Chinese National Influenza Center |  |
| EPI439489 | PB1 | China | 2013-Feb-26 | A/Shanghai/1/2013 |  | WHO Chinese National Influenza Center |  |
| EPI439490 | PA | China | 2013-Feb-26 | A/Shanghai/1/2013 |  | WHO Chinese National Influenza Center |  |
| EPI439486 | HA | China | 2013-Feb-26 | A/Shanghai/1/2013 |  | WHO Chinese National Influenza Center |  |
| EPI439487 | NA | China | 2013-Feb-26 | A/Shanghai/1/2013 |  | WHO Chinese National Influenza Center |  |
| EPI439491 | NP | China | 2013-Feb-26 | A/Shanghai/1/2013 |  | WHO Chinese National Influenza Center |  |
| EPI439494 | NS | China | 2013-Feb-26 | A/Shanghai/1/2013 |  | WHO Chinese National Influenza Center |  |
| EPI301157 | MP | China | 2011-Jan-04 | A/Jiangsu/1/2011 |  | WHO Chinese National Influenza Center | Yuelong,Shu |
| EPI301159 | NP | China | 2011-Jan-04 | A/Jiangsu/1/2011 |  | WHO Chinese National Influenza Center | Yuelong,Shu |
| EPI301158 | NA | China | 2011-Jan-04 | A/Jiangsu/1/2011 |  | WHO Chinese National Influenza Center | Yuelong,Shu |
| EPI301163 | PB2 | China | 2011-Jan-04 | A/Jiangsu/1/2011 |  | WHO Chinese National Influenza Center | Yuelong,Shu |
| EPI301161 | PA | China | 2011-Jan-04 | A/Jiangsu/1/2011 |  | WHO Chinese National Influenza Center | Yuelong,Shu |
| EPI301156 | HA | China | 2011-Jan-04 | A/Jiangsu/1/2011 |  | WHO Chinese National Influenza Center | Yuelong,Shu |
| EPI301160 | NS | China | 2011-Jan-04 | A/Jiangsu/1/2011 |  | WHO Chinese National Influenza Center | Yuelong,Shu |
| EPI301162 | PB1 | China | 2011-Jan-04 | A/Jiangsu/1/2011 |  | WHO Chinese National Influenza Center | Yuelong,Shu |
| EPI291905 | NP | United States | 2007-Aug-17 | A/Ohio/02/2007 | Ohio Department of Health Laboratories | Centers for Disease Control and Prevention |  |
| EPI291906 | PA | United States | 2007-Aug-17 | A/Ohio/02/2007 | Ohio Department of Health Laboratories | Centers for Disease Control and Prevention |  |
| EPI291907 | PB1 | United States | 2007-Aug-17 | A/Ohio/02/2007 | Ohio Department of Health Laboratories | Centers for Disease Control and Prevention |  |
| EPI291908 | PB2 | United States | 2007-Aug-17 | A/Ohio/02/2007 | Ohio Department of Health Laboratories | Centers for Disease Control and Prevention |  |
| EPI178949 | HA | United States | 2007-Aug-17 | A/Ohio/02/2007 | Ohio Department of Health Laboratories | Centers for Disease Control and Prevention |  |
| EPI291903 | NS | United States | 2007-Aug-17 | A/Ohio/02/2007 | Ohio Department of Health Laboratories | Centers for Disease Control and Prevention |  |
| EPI291904 | MP | United States | 2007-Aug-17 | A/Ohio/02/2007 | Ohio Department of Health Laboratories | Centers for Disease Control and Prevention |  |
| EPI338828 | NA | United States | 2007-Aug-17 | A/Ohio/02/2007 | Ohio Department of Health Laboratories | Centers for Disease Control and Prevention |  |
| EPI980836 | NA | China | 2014-Sep-15 | A/chicken/Hebei/LZF/2014(H5N2) | South China Agricultural University | South China Agricultural University | Yuandi,Yu;Zaoyue,Zhang;Wenbao,Qi;Ming,Liao |
| EPI919532 | PA | China | 2016-Jul-24 | A/Chicken/Heyuan/16876/2016(H7N9) | South China Agricultural University | South China Agricultural University | Weixin,Jia;Li,Xing;Zhixian,Li;Wenbao,Qi;Ming,Liao |
| EPI919533 | HA | China | 2016-Jul-24 | A/Chicken/Heyuan/16876/2016(H7N9) | South China Agricultural University | South China Agricultural University | Weixin,Jia;Li,Xing;Zhixian,Li;Wenbao,Qi;Ming,Liao |
| EPI919531 | PB1 | China | 2016-Jul-24 | A/Chicken/Heyuan/16876/2016(H7N9) | South China Agricultural University | South China Agricultural University | Weixin,Jia;Li,Xing;Zhixian,Li;Wenbao,Qi;Ming,Liao |
| EPI919536 | MP | China | 2016-Jul-24 | A/Chicken/Heyuan/16876/2016(H7N9) | South China Agricultural University | South China Agricultural University | Weixin,Jia;Li,Xing;Zhixian,Li;Wenbao,Qi;Ming,Liao |
| EPI919537 | NS | China | 2016-Jul-24 | A/Chicken/Heyuan/16876/2016(H7N9) | South China Agricultural University | South China Agricultural University | Weixin,Jia;Li,Xing;Zhixian,Li;Wenbao,Qi;Ming,Liao |
| EPI919530 | PB2 | China | 2016-Jul-24 | A/Chicken/Heyuan/16876/2016(H7N9) | South China Agricultural University | South China Agricultural University | Weixin,Jia;Li,Xing;Zhixian,Li;Wenbao,Qi;Ming,Liao |
| EPI919534 | NP | China | 2016-Jul-24 | A/Chicken/Heyuan/16876/2016(H7N9) | South China Agricultural University | South China Agricultural University | Weixin,Jia;Li,Xing;Zhixian,Li;Wenbao,Qi;Ming,Liao |
| EPI919535 | NA | China | 2016-Jul-24 | A/Chicken/Heyuan/16876/2016(H7N9) | South China Agricultural University | South China Agricultural University | Weixin,Jia;Li,Xing;Zhixian,Li;Wenbao,Qi;Ming,Liao |
| EPI917099 | PB2 | China | 2016-Dec-28 | A/Chicken/Huizhou/HZ-3/2016(H7N9) | South China Agricultural University | South China Agricultural University | Wenbao,Qi;Bo,Li;Jiahao,Zhang;Guangjie,Lao;Guanming,Su;Weixin,Jia;Ming,Liao |
| EPI917100 | PB1 | China | 2016-Dec-28 | A/Chicken/Huizhou/HZ-3/2016(H7N9) | South China Agricultural University | South China Agricultural University | Wenbao,Qi;Bo,Li;Jiahao,Zhang;Guangjie,Lao;Guanming,Su;Weixin,Jia;Ming,Liao |
| EPI917101 | PA | China | 2016-Dec-28 | A/Chicken/Huizhou/HZ-3/2016(H7N9) | South China Agricultural University | South China Agricultural University | Wenbao,Qi;Bo,Li;Jiahao,Zhang;Guangjie,Lao;Guanming,Su;Weixin,Jia;Ming,Liao |
| EPI917106 | NS | China | 2016-Dec-28 | A/Chicken/Huizhou/HZ-3/2016(H7N9) | South China Agricultural University | South China Agricultural University | Wenbao,Qi;Bo,Li;Jiahao,Zhang;Guangjie,Lao;Guanming,Su;Weixin,Jia;Ming,Liao |
| EPI917102 | HA | China | 2016-Dec-28 | A/Chicken/Huizhou/HZ-3/2016(H7N9) | South China Agricultural University | South China Agricultural University | Wenbao,Qi;Bo,Li;Jiahao,Zhang;Guangjie,Lao;Guanming,Su;Weixin,Jia;Ming,Liao |
| EPI917104 | NA | China | 2016-Dec-28 | A/Chicken/Huizhou/HZ-3/2016(H7N9) | South China Agricultural University | South China Agricultural University | Wenbao,Qi;Bo,Li;Jiahao,Zhang;Guangjie,Lao;Guanming,Su;Weixin,Jia;Ming,Liao |
| EPI917105 | MP | China | 2016-Dec-28 | A/Chicken/Huizhou/HZ-3/2016(H7N9) | South China Agricultural University | South China Agricultural University | Wenbao,Qi;Bo,Li;Jiahao,Zhang;Guangjie,Lao;Guanming,Su;Weixin,Jia;Ming,Liao |
| EPI917103 | NP | China | 2016-Dec-28 | A/Chicken/Huizhou/HZ-3/2016(H7N9) | South China Agricultural University | South China Agricultural University | Wenbao,Qi;Bo,Li;Jiahao,Zhang;Guangjie,Lao;Guanming,Su;Weixin,Jia;Ming,Liao |
| EPI580385 | PB1 | China | 2015-Feb-10 | A/Chicken/Guangdong/SW154/2015 | South China Agricultural University | South China Agricultural University | Shumin,Xie;Weixin,Jia;Yicun,Lin;Kaixiang,Xing;Xingxing,Ren;Wenbao,Qi;Ming,Liao |
| EPI580387 | HA | China | 2015-Feb-10 | A/Chicken/Guangdong/SW154/2015 | South China Agricultural University | South China Agricultural University | Shumin,Xie;Weixin,Jia;Yicun,Lin;Kaixiang,Xing;Xingxing,Ren;Wenbao,Qi;Ming,Liao |
| EPI580389 | NA | China | 2015-Feb-10 | A/Chicken/Guangdong/SW154/2015 | South China Agricultural University | South China Agricultural University | Shumin,Xie;Weixin,Jia;Yicun,Lin;Kaixiang,Xing;Xingxing,Ren;Wenbao,Qi;Ming,Liao |
| EPI580390 | MP | China | 2015-Feb-10 | A/Chicken/Guangdong/SW154/2015 | South China Agricultural University | South China Agricultural University | Shumin,Xie;Weixin,Jia;Yicun,Lin;Kaixiang,Xing;Xingxing,Ren;Wenbao,Qi;Ming,Liao |
| EPI580384 | PB2 | China | 2015-Feb-10 | A/Chicken/Guangdong/SW154/2015 | South China Agricultural University | South China Agricultural University | Shumin,Xie;Weixin,Jia;Yicun,Lin;Kaixiang,Xing;Xingxing,Ren;Wenbao,Qi;Ming,Liao |
| EPI580386 | PA | China | 2015-Feb-10 | A/Chicken/Guangdong/SW154/2015 | South China Agricultural University | South China Agricultural University | Shumin,Xie;Weixin,Jia;Yicun,Lin;Kaixiang,Xing;Xingxing,Ren;Wenbao,Qi;Ming,Liao |
| EPI580391 | NS | China | 2015-Feb-10 | A/Chicken/Guangdong/SW154/2015 | South China Agricultural University | South China Agricultural University | Shumin,Xie;Weixin,Jia;Yicun,Lin;Kaixiang,Xing;Xingxing,Ren;Wenbao,Qi;Ming,Liao |
| EPI580388 | NP | China | 2015-Feb-10 | A/Chicken/Guangdong/SW154/2015 | South China Agricultural University | South China Agricultural University | Shumin,Xie;Weixin,Jia;Yicun,Lin;Kaixiang,Xing;Xingxing,Ren;Wenbao,Qi;Ming,Liao |
| EPI1208375 | HA | China | 2014-Mar-30 | A/duck/Guangdong/673/2014(H5N6) | South China Agricultural University | South China Agricultural University | Yuandi,Yu;Zaoyue,Zhang;Wenbao,Qi;Ming,Liao |
| EPI980834 | NA | China | 2014-Mar-30 | A/duck/Guangdong/673/2014(H5N6) | South China Agricultural University | South China Agricultural University | Yuandi,Yu;Zaoyue,Zhang;Wenbao,Qi;Ming,Liao |
