# Supplementary material for: Rule-based meta-analysis reveals the major role of PB2 in influencing influenza A virus virulence in mice
Source: BMC Genomics. 2019 Dec 24;20(Suppl 9):973. doi: 10.1186/s12864-019-6295-8 (PMC6929465; doi:10.1186/s12864-019-6295-8)
Supplement: Supplementary file 11 — Additional file 11: Table S7. Extrapolated incomplete IAV genomes. [file 12864_2019_6295_MOESM11_ESM.docx]

**Table S7.** Extrapolated incomplete IAV genomes.

| **No.** | **Query** | | | | | **Selected influenza strain and sequences for extrapolation** | | | |
| --- | --- | --- | --- | --- | --- | --- | --- | --- | --- |
|  | **Genome ID** | **Sequence ID** | **IAV strain** | **Segment** | **Methods** | **Sequence ID** | **IAV strain** | **Query cover** | **Identity** |
| 1 | H5TK13 | ?????? | A/Turkey/13/2006(H5N1) | 1 | Based on the closseness of the collection year and name | EF620011 | A/Turkey/15/2006(H5N1) |  |  |
|  | H5TK13 | ?????? | A/Turkey/13/2006(H5N1) | 2 | Based on the closseness of the collection year and name | EF620010 | A/Turkey/15/2006(H5N1) |  |  |
|  | H5TK13 | ?????? | A/Turkey/13/2006(H5N1) | 3 | Based on the closseness of the collection year and name | EF620009 | A/Turkey/15/2006(H5N1) |  |  |
|  | H5TK13 | ?????? | A/Turkey/13/2006(H5N1) | 4 | Based on the closseness of the collection year and name | EF619989 | A/Turkey/15/2006(H5N1) |  |  |
|  | H5TK13 | ?????? | A/Turkey/13/2006(H5N1) | 5 | Based on the closseness of the collection year and name | EF620007 | A/Turkey/15/2006(H5N1) |  |  |
|  | H5TK13 | ?????? | A/Turkey/13/2006(H5N1) | 6 | Based on the closseness of the collection year and name | EF619988 | A/Turkey/15/2006(H5N1) |  |  |
|  | H5TK13 | ?????? | A/Turkey/13/2006(H5N1) | 7 | Based on the closseness of the collection year and name | EF620006 | A/Turkey/15/2006(H5N1) |  |  |
|  | H5TK13 | ?????? | A/Turkey/13/2006(H5N1) | 8 | Based on the closseness of the collection year and name | EF620008 | A/Turkey/15/2006(H5N1) |  |  |
| 2 | K173 | AB671296 | A/Kawasaki/173/2001(H1N1) | 4 |  | CY003024 | A/New York/341/2001(H1N1) | 98% | 99.72% |
|  | K173 | AB671297 | A/Kawasaki/173/2001(H1N1) | 6 |  | CY003026 | A/New York/341/2001(H1N1) | 100% | 99.93% |
|  | K173 | ?????? | A/Kawasaki/173/2001(H1N1) | 1 | BLAST the HA and NA | CY003031 | A/New York/341/2001(H1N1) |  |  |
|  | K173 | ?????? | A/Kawasaki/173/2001(H1N1) | 2 | BLAST the HA and NA | CY003030 | A/New York/341/2001(H1N1) |  |  |
|  | K173 | ?????? | A/Kawasaki/173/2001(H1N1) | 3 | BLAST the HA and NA | CY003029 | A/New York/341/2001(H1N1) |  |  |
|  | K173 | ?????? | A/Kawasaki/173/2001(H1N1) | 5 | BLAST the HA and NA | CY003027 | A/New York/341/2001(H1N1) |  |  |
|  | K173 | ?????? | A/Kawasaki/173/2001(H1N1) | 7 | BLAST the HA and NA | CY003025 | A/New York/341/2001(H1N1) |  |  |
|  | K173 | ?????? | A/Kawasaki/173/2001(H1N1) | 8 | BLAST the HA and NA | CY003028 | A/New York/341/2001(H1N1) |  |  |
| 3 | L1337 | KP459007 | A/Lyon/1337/2007(H1N1) | 4 |  | KP459227 | A/Yokohama/78/2008(H1N1) | 100 | 99.88 |
|  | L1337 | HQ658485 | A/Lyon/1337/2007(H1N1) | 6 |  | CY043563 | A/Yokohama/78/2008(H1N1) | 99 | 99.93 |
|  | L1337 | ?????? | A/Lyon/1337/2007(H1N1) | 1 | BLAST the HA and NA | CY043558 | A/Yokohama/78/2008(H1N1) |  |  |
|  | L1337 | ?????? | A/Lyon/1337/2007(H1N1) | 2 | BLAST the HA and NA | CY043559 | A/Yokohama/78/2008(H1N1) |  |  |
|  | L1337 | ?????? | A/Lyon/1337/2007(H1N1) | 3 | BLAST the HA and NA | CY043560 | A/Yokohama/78/2008(H1N1) |  |  |
|  | L1337 | ?????? | A/Lyon/1337/2007(H1N1) | 5 | BLAST the HA and NA | CY043562 | A/Yokohama/78/2008(H1N1) |  |  |
|  | L1337 | ?????? | A/Lyon/1337/2007(H1N1) | 8 | BLAST the HA and NA | CY043565 | A/Yokohama/78/2008(H1N1) |  |  |
| 4 | L48.425 | JF429393 | A/Lyon/48.425/2009(H1N1) | 4 |  | KY926119 | A/Teutonia/LACENRS-711/2009(H1N1) | 100 | 99.82 |
|  | L48.425 | ?????? | A/Lyon/48.425/2009(H1N1) | 1 | BLAST the HA | KY926041 | A/Teutonia/LACENRS-711/2009(H1N1) |  |  |
|  | L48.425 | ?????? | A/Lyon/48.425/2009(H1N1) | 2 | BLAST the HA | KY925290 | A/Teutonia/LACENRS-711/2009(H1N1) |  |  |
|  | L48.425 | ?????? | A/Lyon/48.425/2009(H1N1) | 3 | BLAST the HA | KY926136 | A/Teutonia/LACENRS-711/2009(H1N1) |  |  |
|  | L48.425 | ?????? | A/Lyon/48.425/2009(H1N1) | 5 | BLAST the HA | KY925820 | A/Teutonia/LACENRS-711/2009(H1N1) |  |  |
|  | L48.425 | ?????? | A/Lyon/48.425/2009(H1N1) | 7 | BLAST the HA | KY925621 | A/Teutonia/LACENRS-711/2009(H1N1) |  |  |
|  | L48.425 | ?????? | A/Lyon/48.425/2009(H1N1) | 8 | BLAST the HA | KY926168 | A/Teutonia/LACENRS-711/2009(H1N1) |  |  |
| 5 | LR10 | KF897777 | A/LaReunion/803/2010(H1N1) | 4 |  | JX309590 | A/Singapore/GP2892/2010(H1N1) | 100 | 99.89 |
|  | LR10 | KF897779 | A/LaReunion/803/2010(H1N1) | 6 |  | JX309592 | A/Singapore/GP2892/2010(H1N1) | 100 | 99.52 |
|  | LR10 | ?????? | A/LaReunion/803/2010(H1N1) | 1 | BLAST the HA and NA | JX309587 | A/Singapore/GP2892/2010(H1N1) |  |  |
| 6 | MN81 | CY014726 | A/duck/Minnesota/1525/1981(H5N1) | 4 |  | CY179411 | A/mallard/Wisconsin/568/1982(H5N1) | 98 | 99.6 |
|  | MN81 | ?????? | A/duck/Minnesota/1525/1981(H5N1) | 6 | BLAST the HA | CY179413 | A/mallard/Wisconsin/568/1982(H5N1) |  |  |
| 7 | NL230 | EPI319937 | A/Netherlands/230/2003(H7N7) | 4 |  | AB438941 | A/chicken/Netherlands/2586/2003(H7N7) | 100 | 99.94 |
|  | NL230 | EPI319936 | A/Netherlands/230/2003(H7N7) | 6 |  | AB438943 | A/chicken/Netherlands/2586/2003(H7N7) | 100 | 99.93 |
|  | NL230 | ?????? | A/Netherlands/230/2003(H7N7) | 2 | BLAST the HA and NA | AB438939 | A/chicken/Netherlands/2586/2003(H7N7) |  |  |
|  | NL230 | ?????? | A/Netherlands/230/2003(H7N7) | 3 | BLAST the HA and NA | AB438940 | A/chicken/Netherlands/2586/2003(H7N7) |  |  |
|  | NL230 | ?????? | A/Netherlands/230/2003(H7N7) | 5 | BLAST the HA and NA | AB438942 | A/chicken/Netherlands/2586/2003(H7N7) |  |  |
|  | NL230 | ?????? | A/Netherlands/230/2003(H7N7) | 7 | BLAST the HA and NA | AB438944 | A/chicken/Netherlands/2586/2003(H7N7) |  |  |
|  | NL230 | ?????? | A/Netherlands/230/2003(H7N7) | 8 | BLAST the HA and NA | AB438945 | A/chicken/Netherlands/2586/2003(H7N7) |  |  |
| 8 | NWS33 | U08903 | A/NWS/1933(H1N1) | 4 |  | CY120984 | A/NWS/1934(H1N1) | 99 | 99.77 |
|  | NWS33 | L25815 | A/NWS/1933(H1N1) | 6 |  | CY120986 | A/NWS/1934(H1N1) | 98 | 99.86 |
|  | NWS33 | ?????? | A/NWS/1933(H1N1) | 1 | BLAST the HA and NA | CY120991 | A/NWS/1934(H1N1) |  |  |
|  | NWS33 | ?????? | A/NWS/1933(H1N1) | 2 | BLAST the HA and NA | CY120990 | A/NWS/1934(H1N1) |  |  |
|  | NWS33 | ?????? | A/NWS/1933(H1N1) | 3 | BLAST the HA and NA | CY120989 | A/NWS/1934(H1N1) |  |  |
|  | NWS33 | ?????? | A/NWS/1933(H1N1) | 5 | BLAST the HA and NA | CY120987 | A/NWS/1934(H1N1) |  |  |
| 9 | SC18 | ?????? | A/SouthCarolina/1/18 (H1N1) | 1 | Following related publications | DQ208309 | A/BrevigMission/1/1918(H1N1) |  |  |
|  | SC18 | ?????? | A/SouthCarolina/1/18 (H1N1) | 2 | Following related publications | DQ208310 | A/BrevigMission/1/1918(H1N1) |  |  |
|  | SC18 | ?????? | A/SouthCarolina/1/18 (H1N1) | 3 | Following related publications | DQ208311 | A/BrevigMission/1/1918(H1N1) |  |  |
|  | SC18 | ?????? | A/SouthCarolina/1/18 (H1N1) | 5 | Following related publications | AY744935 | A/BrevigMission/1/1918(H1N1) |  |  |
|  | SC18 | ?????? | A/SouthCarolina/1/18 (H1N1) | 6 | Following related publications | AF250356 | A/BrevigMission/1/1918(H1N1) |  |  |
|  | SC18 | ?????? | A/SouthCarolina/1/18 (H1N1) | 7 | Following related publications | AY130766 | A/BrevigMission/1/1918(H1N1) |  |  |
|  | SC18 | ?????? | A/SouthCarolina/1/18 (H1N1) | 8 | Following related publications | AF333238 | A/BrevigMission/1/1918(H1N1) |  |  |
| 10 | SH1023 | ?????? | A/chicken/Shandong/lx1023/2007(H9N2) | 6 | Based on the closseness of the collection year and name |  | A/chicken/Shandong/L1/2007(H9N2) |  |  |
| 11 | SY01 | EPI272297 | A/Seoul/Y-01/2009(H1N1) | 4 |  | CY128211 | A/Viet Nam/13032036/2009(H1N1) | 100 | 99.82 |
|  | SY01 | EPI272298 | A/Seoul/Y-01/2009(H1N1) | 6 |  | CY128213 | A/Viet Nam/13032036/2009(H1N1) | 99 | 100 |
|  | SY01 | ?????? | A/Seoul/Y-01/2009(H1N1) | 1 | BLAST the HA and NA | CY128218 | A/Viet Nam/13032036/2009(H1N1) |  |  |
|  | SY01 | ?????? | A/Seoul/Y-01/2009(H1N1) | 2 | BLAST the HA and NA | CY128217 | A/Viet Nam/13032036/2009(H1N1) |  |  |
|  | SY01 | ?????? | A/Seoul/Y-01/2009(H1N1) | 3 | BLAST the HA and NA | CY128216 | A/Viet Nam/13032036/2009(H1N1) |  |  |
|  | SY01 | ?????? | A/Seoul/Y-01/2009(H1N1) | 5 | BLAST the HA and NA | CY128214 | A/Viet Nam/13032036/2009(H1N1) |  |  |
|  | SY01 | ?????? | A/Seoul/Y-01/2009(H1N1) | 7 | BLAST the HA and NA | CY128212 | A/Viet Nam/13032036/2009(H1N1) |  |  |
|  | SY01 | ?????? | A/Seoul/Y-01/2009(H1N1) | 8 | BLAST the HA and NA | CY128215 | A/Viet Nam/13032036/2009(H1N1) |  |  |
| 12 | TN560 | CY040457 | A/Tennessee/1-560/2009(H1N1) | 4 |  | KY925045 | A/Sao Gabriel/LACENRS-1626/2009(H1N1) | 100 | 99.94 |
|  | TN560 | CY040458 | A/Tennessee/1-560/2009(H1N1) | 6 |  | KY925348 | A/Sao Gabriel/LACENRS-1626/2009(H1N1) | 100 | 99.93 |
|  | TN560 | ?????? | A/Tennessee/1-560/2009(H1N1) | 5 | BLAST the HA and NA | KY926173 | A/Sao Gabriel/LACENRS-1626/2009(H1N1) |  |  |
|  | TN560 | ?????? | A/Tennessee/1-560/2009(H1N1) | 7 | BLAST the HA and NA | KY926049 | A/Sao Gabriel/LACENRS-1626/2009(H1N1) |  |  |
|  | TN560 | ?????? | A/Tennessee/1-560/2009(H1N1) | 8 | BLAST the HA and NA | KY925946 | A/Sao Gabriel/LACENRS-1626/2009(H1N1) |  |  |
| 13 | UTK09 | AB671291 | A/Kawasaki/UTK-4/2009(H1N1) | 4 |  | CY043497 | A/Niigata/08F031/2009(H1N1) | 100 | 99.89 |
|  | UTK09 | AB671292 | A/Kawasaki/UTK-4/2009(H1N1) | 6 |  | CY043499 | A/Niigata/08F031/2009(H1N1) | 100 | 99.59 |
|  | UTK09 | ?????? | A/Kawasaki/UTK-4/2009(H1N1) | 1 | BLAST the HA and NA | CY043494 | A/Niigata/08F031/2009(H1N1) |  |  |
|  | UTK09 | ?????? | A/Kawasaki/UTK-4/2009(H1N1) | 2 | BLAST the HA and NA | CY043495 | A/Niigata/08F031/2009(H1N1) |  |  |
|  | UTK09 | ?????? | A/Kawasaki/UTK-4/2009(H1N1) | 3 | BLAST the HA and NA | CY043496 | A/Niigata/08F031/2009(H1N1) |  |  |
|  | UTK09 | ?????? | A/Kawasaki/UTK-4/2009(H1N1) | 5 | BLAST the HA and NA | CY043498 | A/Niigata/08F031/2009(H1N1) |  |  |
|  | UTK09 | ?????? | A/Kawasaki/UTK-4/2009(H1N1) | 7 | BLAST the HA and NA | CY043500 | A/Niigata/08F031/2009(H1N1) |  |  |
|  | UTK09 | ?????? | A/Kawasaki/UTK-4/2009(H1N1) | 8 | BLAST the HA and NA | CY043501 | A/Niigata/08F031/2009(H1N1) |  |  |
| 14 | VN1204 | EF541404 | A/VietNam/1204/2004(H5N1) | 4 |  | HM006759 | A/Viet Nam/1203/2004(H5N1) | 100 | 100 |
|  | VN1204 | AB745467 | A/VietNam/1204/2004(H5N1) | 6 |  | HM006761 | A/Viet Nam/1203/2004(H5N1) | 100 | 100 |
|  | VN1204 | ?????? | A/VietNam/1204/2004(H5N1) | 5 | BLAST the HA and NA | HM006760 | A/Viet Nam/1203/2004(H5N1) |  |  |
|  | VN1204 | ?????? | A/VietNam/1204/2004(H5N1) | 7 | BLAST the HA and NA | HM006762 | A/Viet Nam/1203/2004(H5N1) |  |  |
