# Supplementary material for: Rule-based meta-analysis reveals the major role of PB2 in influencing influenza A virus virulence in mice
Source: BMC Genomics. 2019 Dec 24;20(Suppl 9):973. doi: 10.1186/s12864-019-6295-8 (PMC6929465; doi:10.1186/s12864-019-6295-8)
Supplement: Supplementary file 12 — Additional file 12: Table S8. Extrapolated partial IAV segments. [file 12864_2019_6295_MOESM12_ESM.docx]

**Table S8.** Extrapolated partial IAV segments.

| **No.** | **Query** | | | | | **BLAST hit for extrapolation** | | | |
| --- | --- | --- | --- | --- | --- | --- | --- | --- | --- |
|  | **Genome ID** | **Sequence ID** | **IAV strain** | **Segment** | **Notes** | **Sequence ID** | **IAV strain** | **Query cover** | **Percent Identity** |
| 1 | DKFJ01 | AY585441 | A/duck/Fujian/01/2002(H5N1) | 8 |  | HQ259228 | A/mallard/Bavaria/185-8/2008(H1N1) | 100% | 93.88% |
| 2 | DKGX53 | AY585454 | A/duck/Guangxi/53/2002(H5N1) | 8 |  | DQ997525 | A/goose/Guangdong/xb/2001(H5N1) | 100% | 99.42% |
| 3 | GSH7 | EPI1215865 | A/goose/Guangdong/SH7/2013(H5N1) | 7 |  | KP732557 | A/chicken/AnNing/1/2014(H5N1) | 100% | 100.00% |
| 4 | GSHK437 | GU052022 | A/goose/HongKong/437-6/1999(H5N1) | 5 |  | AF216720 | A/environment/Hong Kong/437-6/99 (H5N1) | 99% | 99.92% |
| 5 | H5TK13 | EF620011 | A/Turkey/13/2006(H5N1) | 1 | including insertion at position 2079 | EU146846 | A/Iraq/1/2006(H5N1) | 100% | 99.63% |
|  | H5TK13 | EF620009 | A/Turkey/13/2006(H5N1) | 3 |  | EF619995 | A/Turkey/651242/2006(H5N1) | 100% | 100.00% |
|  | H5TK13 | EF619989 | A/Turkey/13/2006(H5N1) | 4 |  | EF446779 | A/goose/Hungary/3413/2007(H5N1) | 100% | 99.46% |
|  | H5TK13 | EF620007 | A/Turkey/13/2006(H5N1) | 5 |  | DQ323677 | A/chicken/Kurgan/3/2005(H5N1) | 100% | 99.65% |
|  | H5TK13 | EF619988 | A/Turkey/13/2006(H5N1) | 6 |  | EF620000 | A/Turkey/65596/2006(H5N1) | 100% | 100.00% |
|  | H5TK13 | EF620006 | A/Turkey/13/2006(H5N1) | 7 |  | DQ234077 | A/grebe/Novosibirsk/29/2005(H5N1) | 100% | 99.79% |
|  | H5TK13 | EF620008 | A/Turkey/13/2006(H5N1) | 8 |  | EU599286 | A/chicken/Iraq/900845/2006(H5N1) | 100% | 99.75% |
| 6 | HA4 | GQ166219 | A/Hamburg/4/2009(H1N1) | 7 |  | KX571118 | A/swine/Quebec/1279825/2011(H3N2) | 100% | 100.00% |
| 7 | HEY16 | EPI919533 | A/chicken/Heyuan/16876/2016(H7N9) | 4 |  | KY751065 | A/chicken/Jiangxi/JX4/2017(H7N9) | 100% | 99.94% |
|  | HEY16 | EPI919537 | A/chicken/Heyuan/16876/2016(H7N9) | 8 |  | MH209551 | A/environment/Guangdong/S12706/2017(H7N9) | 100% | 100.00% |
| 8 | HH05 | HQ111367 | A/Hamburg/05/2009(H1N1) | 7 |  | CY266344 | A/California/07-00019/2009(H1N1) | 100% | 100.00% |
|  | HH05 | HQ111368 | A/Hamburg/05/2009(H1N1) | 8 |  | CY266347 | A/California/07-00019/2009(H1N1) | 100% | 100.00% |
| 9 | HH15 | HQ104928 | A/Hamburg/NY1580/2009(H1N1) | 7 |  | KP412332 | A/swine/Illinois/21-1230/2009(H1N1) | 100% | 100.00% |
|  | HH15 | HQ104929 | A/Hamburg/NY1580/2009(H1N1) | 8 |  | JX625532 | A/Northern Ireland/94480397/2009(H1N1) | 100% | 100.00% |
| 10 | HK156v1 | AF036361 | A/HongKong/156/1997(H5N1) | 3 |  | AJ289874 | A/Hong Kong/156/97(H5N1) | 100% | 99.83% |
|  | HK156v1 | AF036356 | A/HongKong/156/1997(H5N1) | 4 |  | GU052127 | A/environment/Hong Kong/156/1997(H5N1) | 100% | 99.94% |
| 11 | HK156v2 | AF046093 | A/HongKong/156/1997(H5N1) | 1 |  | GU052134 | A/environment/Hong Kong/156/1997(H5N1) | 100% | 100.00% |
| 12 | HK481v1 | AF102663 | A/HongKong/481/1997(H5N1) | 6 |  | AF084271 | A/HongKong/481/1997(H5N1) | 100% | 99.78% |
| 13 | HK481v2 | AF084279 | A/HongKong/481/1997(H5N1) | 4 |  | AF046096 | A/HongKong/481/1997(H5N1) | 100% | 99.94% |
| 14 | HK483v2 | AF084280 | A/HongKong/483/1997(H5N1) | 4 |  | AF046097 | A/Hong Kong/483/97(H5N1) | 100% | 99.94% |
| 15 | HK483v3 | GU052103 | A/HongKong/483/1997(H5N1) | 2 |  | GU052182 | A/environment/Hong Kong/258/1997(H5) | 100% | 99.85% |
|  | HK483v3 | GU052100 | A/HongKong/483/1997(H5N1) | 5 |  | AF255746 | A/Hong Kong/483/97(H5N1) | 100% | 100.00% |
| 16 | HK485v1 | AF258847 | A/HongKong/485/1997(H5N1) | 1 |  | GU052149 | A/Hong Kong/485/1997(H5N1) | 100% | 99.06% |
|  | HK485v1 | AF258828 | A/HongKong/485/1997(H5N1) | 2 |  | GU052182 | A/environment/Hong Kong/258/1997(H5) | 100% | 99.68% |
|  | HK485v1 | AF257203 | A/HongKong/485/1997(H5N1) | 3 |  | AF046087 | A/Chicken/Hong Kong/220/97(H5N1) | 100% | 99.68% |
|  | HK485v1 | AF102681 | A/HongKong/485/1997(H5N1) | 4 |  | GU052142 | A/Hong Kong/485/1997(H5N1) | 100% | 100.00% |
|  | HK485v1 | AH010699 | A/HongKong/485/1997(H5N1) | 5 |  | GU052145 | A/Hong Kong/485/1997(H5N1) | 91% | 99.72% |
|  | HK485v1 | AF102664 | A/HongKong/485/1997(H5N1) | 6 |  | GU052144 | A/Hong Kong/485/1997(H5N1) | 100% | 99.85% |
| 17 | HK485v1 | GU052143 | A/HongKong/485/1997(H5N1) | 7 |  | AF046082 | A/Chicken/Hong Kong/220/97 (H5N1) | 100% | 99.72% |
|  | HK485v1 | AH010696 | A/HongKong/485/1997(H5N1) | 7 |  | JX465629 | A/chicken/Iran/ZMT-101/1998(H9N2) | 86% | 99.41% |
|  | HK485v1 | AF256189 | A/HongKong/485/1997(H5N1) | 8 |  | GU052146 | A/Hong Kong/485/1997(H5N1) | 100% | 100.00% |
| 18 | HK485v2 | AF084532 | A/HongKong/485/1997(H5N1) | 4 |  | GU052142 | A/Hong Kong/485/1997(H5N1) | 100% | 100.00% |
| 19 | HK486v1 | AF102671 | A/HongKong/486/1997(H5N1) | 4 |  | AF046098 | A/Hong Kong/482/97(H5N1) | 100% | 100.00% |
| 20 | HK486v2 | AF084281 | A/HongKong/486/1997(H5N1) | 4 |  | AF046098 | A/Hong Kong/482/97(H5N1) | 100% | 100.00% |
| 21 | HK488 | AF258848 | A/HongKong/488/1997(H5N1) | 1 |  | GU052041 | A/environment/Hong Kong/486/1997(H5N1) | 100% | 99.38% |
|  | HK488 | AF258829 | A/HongKong/488/1997(H5N1) | 2 |  | GU052040 | A/environment/Hong Kong/486/1997(H5N1) | 100% | 99.68% |
|  | HK488 | AF257204 | A/HongKong/488/1997(H5N1) | 3 |  | AF046087 | A/Chicken/Hong Kong/220/97(H5N1) | 100% | 99.68% |
|  | HK488 | AF102672 | A/HongKong/488/1997(H5N1) | 4 |  | AF046098 | A/Hong Kong/482/97(H5N1) | 100% | 99.94% |
|  | HK488 | AH010700 | A/HongKong/488/1997(H5N1) | 5 |  | GU052037 | A/environment/Hong Kong/486/1997(H5N1) | 90% | 100.00% |
|  | HK488 | AF102657 | A/HongKong/488/1997(H5N1) | 6 |  | GU052036 | A/environment/Hong Kong/486/1997(H5N1) | 100% | 99.70% |
|  | HK488 | AH010697 | A/HongKong/488/1997(H5N1) | 7 | Replace Ns at the centre | AF255373 | A/Hong Kong/542/97(H5N1) | 86% | 99.71% |
|  | HK488 | AF256190 | A/HongKong/488/1997(H5N1) | 8 |  | AF084285 | A/HongKong/482/97(H5N1) | 100% | 100.00% |
| 22 | HK491 | AF258849 | A/HongKong/491/1997(H5N1) | 1 |  | AF258845 | A/Hong Kong/542/97(H5N1) | 100% | 99.69% |
|  | HK491 | AF258830 | A/HongKong/491/1997(H5N1) | 2 |  | AF258826 | A/Hong Kong/542/97(H5N1) | 100% | 100.00% |
|  | HK491 | AF257205 | A/HongKong/491/1997(H5N1) | 3 |  | AF046087 | A/Chicken/Hong Kong/220/97 (H5N1) | 100% | 99.70% |
|  | HK491 | AF102677 | A/HongKong/491/1997(H5N1) | 4 |  | AF082034 | A/Chicken/Hong Kong/728/97 (H5N1) | 100% | 99.82% |
|  | HK491 | AH010701 | A/HongKong/491/1997(H5N1) | 5 |  | AF098619 | A/Chicken/Hong Kong/786/97 (H5N1) | 90% | 99.69% |
|  | HK491 | AF102665 | A/HongKong/491/1997(H5N1) | 6 |  | GU052122 | A/chicken/China/27402/1997(H5N1) | 100% | 99.41% |
|  | HK491 | AH010698 | A/HongKong/491/1997(H5N1) | 7 |  | AF255365 | A/Hong Kong/481/97(H5N1) | 87% | 99.25% |
|  | HK491 | AF256191 | A/HongKong/491/1997(H5N1) | 8 |  | GU052138 | A/chicken/Hong Kong/786-2/1997(H5N1) | 100% | 99.69% |
| 23 | HK503 | AF258850 | A/HongKong/503/1997(H5N1) | 1 |  | AF258845 | A/Hong Kong/542/97(H5N1) | 100% | 99.67% |
|  | HK503 | AF258831 | A/HongKong/503/1997(H5N1) | 2 |  | AF258826 | A/Hong Kong/542/97(H5N1) | 100% | 99.74% |
|  | HK503 | AF257206 | A/HongKong/503/1997(H5N1) | 3 |  | AF046087 | A/Chicken/Hong Kong/220/97(H5N1) | 100% | 100.00% |
|  | HK503 | AF102679 | A/HongKong/503/1997(H5N1) | 4 |  | AF082034 | A/Chicken/Hong Kong/728/97(H5N1) | 100% | 99.21% |
|  | HK503 | AH010702 | A/HongKong/503/1997(H5N1) | 5 |  | AF098619 | A/Chicken/Hong Kong/786/97(H5N1) | 89% | 100.00% |
|  | HK503 | AF102666 | A/HongKong/503/1997(H5N1) | 6 |  | GU052122 | A/chicken/China/27402/1997(H5N1) | 100% | 99.11% |
|  | HK503 | AF255381 | A/HongKong/503/1997(H5N1) | 7 |  | AF255373 | A/Hong Kong/542/97(H5N1) | 100% | 99.71% |
|  | HK503 | AF256192 | A/HongKong/503/1997(H5N1) | 8 |  | GU052138 | A/chicken/Hong Kong/786-2/1997(H5N1) | 100% | 98.28% |
| 24 | HK507 | AF258851 | A/HongKong/507/1997(H5N1) | 1 |  | GU052041 | A/environment/Hong Kong/486/1997(H5N1) | 100% | 100.00% |
|  | HK507 | AF258832 | A/HongKong/507/1997(H5N1) | 2 |  | GU052040 | A/environment/Hong Kong/486/1997(H5N1) | 100% | 99.31% |
|  | HK507 | AF257207 | A/HongKong/507/1997(H5N1) | 3 |  | AF046087 | A/Chicken/Hong Kong/220/97(H5N1) | 100% | 99.67% |
|  | HK507 | AF102675 | A/HongKong/507/1997(H5N1) | 4 |  | AF046098 | A/Hong Kong/482/97(H5N1) | 100% | 99.64% |
|  | HK507 | AH010703 | A/HongKong/507/1997(H5N1) | 5 |  | GU052037 | A/environment/Hong Kong/486/1997(H5N1) | 85% | 100.00% |
|  | HK507 | AF102659 | A/HongKong/507/1997(H5N1) | 6 |  | GU052036 | A/environment/Hong Kong/486/1997(H5N1) | 100% | 99.56% |
|  | HK507 | AF255382 | A/HongKong/507/1997(H5N1) | 7 |  | AF255372 | A/Hong Kong/538/97(H5N1) | 100% | 100.00% |
|  | HK507 | AF256193 | A/HongKong/507/1997(H5N1) | 8 |  | GU052038 | A/environment/Hong Kong/486/1997(H5N1) | 100% | 98.87% |
| 25 | HK514 | AF258852 | A/HongKong/514/1997(H5N1) | 1 |  | AB586776 | A/quail/Hong Kong/NT342/2001(H6N1) | 100% | 99.64% |
|  | HK514 | AF258833 | A/HongKong/514/1997(H5N1) | 2 |  | GU052182 | A/environment/Hong Kong/258/1997(H5) | 100% | 99.73% |
|  | HK514 | AF257208 | A/HongKong/514/1997(H5N1) | 3 |  | AF046087 | A/Chicken/Hong Kong/220/97(H5N1) | 100% | 98.90% |
|  | HK514 | AF102682 | A/HongKong/514/1997(H5N1) | 4 |  | GU052142 | A/Hong Kong/485/1997(H5N1) | 100% | 99.03% |
|  | HK514 | AH010704 | A/HongKong/514/1997(H5N1) | 5 |  | AF046084 | A/Chicken/Hong Kong/220/97 (H5N1) | 90% | 99.86% |
|  | HK514 | AF102669 | A/HongKong/514/1997(H5N1) | 6 |  | GU052122 | A/chicken/China/27402/1997(H5N1) | 100% | 98.74% |
|  | HK514 | AF255383 | A/HongKong/514/1997(H5N1) | 7 |  | KY785902 | A/quail/Hong Kong/G1/1997(H9N2) | 100% | 100.00% |
| 26 | HK516 | AF258853 | A/HongKong/516/1997(H5N1) | 1 |  | GU052041 | A/environment/Hong Kong/486/1997(H5N1) | 100% | 99.64% |
|  | HK516 | AF258834 | A/HongKong/516/1997(H5N1) | 2 |  | GU052040 | A/environment/Hong Kong/486/1997(H5N1) | 100% | 100.00% |
|  | HK516 | AF257209 | A/HongKong/516/1997(H5N1) | 3 |  | GU052039 | A/environment/Hong Kong/486/1997(H5N1) | 100% | 100.00% |
|  | HK516 | AF102673 | A/HongKong/516/1997(H5N1) | 4 |  | AF046098 | A/Hong Kong/482/97(H5N1) | 100% | 99.94% |
|  | HK516 | AH010705 | A/HongKong/516/1997(H5N1) | 5 |  | GU052037 | A/environment/Hong Kong/486/1997(H5N1) | 89% | 100.00% |
|  | HK516 | AF102660 | A/HongKong/516/1997(H5N1) | 6 |  | AF084272 | A/HongKong/482/97(H5N1) | 100% | 99.85% |
|  | HK516 | AF255384 | A/HongKong/516/1997(H5N1) | 7 |  | AF255372 | A/Hong Kong/538/97(H5N1) | 100% | 100.00% |
|  | HK516 | AF256194 | A/HongKong/516/1997(H5N1) | 8 |  | GU052038 | A/environment/Hong Kong/486/1997(H5N1) | 100% | 99.66% |
| 27 | HK532 | AF102680 | A/HongKong/532/1997(H5N1) | 4 |  | GU052135 | A/chicken/Hong Kong/786-2/1997(H5N1) | 100% | 99.40% |
|  | HK532 | AF102667 | A/HongKong/532/1997(H5N1) | 6 |  | GU052122 | A/chicken/China/27402/1997(H5N1) | 100% | 99.19% |
| 28 | HK538 | AF102674 | A/HongKong/538/1997(H5N1) | 4 |  | AF046098 | A/Hong Kong/482/97(H5N1) | 100% | 99.82% |
|  | HK538 | AF102662 | A/HongKong/538/1997(H5N1) | 6 |  | AF084272 | A/HongKong/482/97(H5N1) | 100% | 99.33% |
| 29 | HK542 | AF102678 | A/HongKong/542/1997(H5N1) | 4 |  | AF082034 | A/Chicken/Hong Kong/728/97(H5N1) | 100% | 99.70% |
|  | HK542 | AF102670 | A/HongKong/542/1997(H5N1) | 6 |  | GU052122 | A/chicken/China/27402/1997(H5N1) | 100% | 98.67% |
| 30 | HK97 | AF102676 | A/HongKong/97/1998(H5N1) | 4 |  | AF046098 | A/Hong Kong/482/97(H5N1) | 99% | 99.52% |
|  | HK97 | AF102661 | A/HongKong/97/1998(H5N1) | 6 |  | AF084272 | A/HongKong/482/97(H5N1) | 100% | 99.56% |
| 31 | L1.12 | KF897812 | A/Lyon/1.12/2011(H1N1) | 7 |  | KY925952 | A/Viamao/LACENRS-1400/2011(H1N1) | 100% | 100.00% |
| 32 | L1337 | KP459007 | A/Lyon/1337/2007(H1N1) | 4 |  | CY105086 | A/KhanhHoa/KH161/2008(H1N1) | 100% | 99.88% |
| 33 | LIMO10 | KF897804 | A/Limoges/1159/2010(H1N1) | 7 |  | JX625745 | A/England/182/2010(H1N1) | 100% | 100.00% |
| 34 | LYON10 | KF897788 | A/Lyon/52.16/2010(H1N1) | 7 |  | KC488882 | A/Chita/RII08/2012(H1N1) | 100% | 100.00% |
| 35 | ma452-G1-1 | ID0007 | A/ma452-G1-1/2014(H5N8) | 7 |  | MG965867 | A/turkey/Wisconsin/15-014298-1/2015(H5N2) | 100% | 100.00% |
|  | ma452-G1-1 | ID0008 | A/ma452-G1-1/2014(H5N8) | 8 |  | MG964812 | A/red-tailed hawk/Washington/15-002551-2/2015(H5N2) | 100% | 100.00% |
| 36 | ma452-G3-1 | ID0015 | A/ma452-G3-1/2014(H5N8) | 7 |  | MG965867 | A/turkey/Wisconsin/15-014298-1/2015(H5N2) | 100% | 100.00% |
|  | ma452-G3-1 | ID0016 | A/ma452-G3-1/2014(H5N8) | 8 |  | MG964812 | A/red-tailed hawk/Washington/15-002551-2/2015(H5N2) | 100% | 100.00% |
| 37 | ma452-G3-2 | ID0023 | A/ma452-G3-2/2014(H5N8) | 7 |  | MG965867 | A/turkey/Wisconsin/15-014298-1/2015(H5N2) | 100% | 100.00% |
|  | ma452-G3-2 | ID0024 | A/ma452-G3-2/2014(H5N8) | 8 |  | MG964812 | A/red-tailed hawk/Washington/15-002551-2/2015(H5N2) | 100% | 100.00% |
| 38 | ma452-G4-1 | ID0031 | A/ma452-G4-1/2014(H5N8) | 7 |  | MG965867 | A/turkey/Wisconsin/15-014298-1/2015(H5N2) | 100% | 100.00% |
|  | ma452-G4-1 | ID0032 | A/ma452-G4-1/2014(H5N8) | 8 |  | MG964812 | A/red-tailed hawk/Washington/15-002551-2/2015(H5N2) | 100% | 100.00% |
| 39 | ma468-G1-1 | ID0039 | A/ma468-G1-1/2014(H5N8) | 7 |  | KX297904 | A/environment/Korea/W477/2014(H5N8) | 100% | 100.00% |
|  | ma468-G1-1 | ID0040 | A/ma468-G1-1/2014(H5N8) | 8 |  | KX297970 | A/environment/Korea/W468/2014(H5N8) | 100% | 100.00% |
| 40 | ma468-G1-2 | ID0047 | A/ma468-G1-2/2014(H5N8) | 7 |  | KX297904 | A/environment/Korea/W477/2014(H5N8) | 100% | 100.00% |
|  | ma468-G1-2 | ID0048 | A/ma468-G1-2/2014(H5N8) | 8 |  | KX297970 | A/environment/Korea/W468/2014(H5N8) | 100% | 100.00% |
| 41 | ma468-G2-1 | ID0055 | A/ma468-G2-1/2014(H5N8) | 7 |  | KX297904 | A/environment/Korea/W477/2014(H5N8) | 100% | 100.00% |
|  | ma468-G2-1 | ID0056 | A/ma468-G2-1/2014(H5N8) | 8 |  | KX297970 | A/environment/Korea/W468/2014(H5N8) | 100% | 100.00% |
| 42 | ma468-G2-2 | ID0063 | A/ma468-G2-2/2014(H5N8) | 7 |  | KX297904 | A/environment/Korea/W477/2014(H5N8) | 100% | 100.00% |
|  | ma468-G2-2 | ID0064 | A/ma468-G2-2/2014(H5N8) | 8 |  | KX297970 | A/environment/Korea/W468/2014(H5N8) | 100% | 100.00% |
| 43 | ma468-G2-3 | ID0071 | A/ma468-G2-3/2014(H5N8) | 7 |  | KX297904 | A/environment/Korea/W477/2014(H5N8) | 100% | 100.00% |
|  | ma468-G2-3 | ID0072 | A/ma468-G2-3/2014(H5N8) | 8 |  | KX297970 | A/environment/Korea/W468/2014(H5N8) | 100% | 100.00% |
| 44 | ma468-G4-2 | ID0076 | A/ma468-G4-2/2014(H5N8) | 4 |  | KX297879 | A/environment/Korea/W477/2014(H5N8) | 100% | 99.88% |
|  | ma468-G4-2 | ID0079 | A/ma468-G4-2/2014(H5N8) | 7 |  | KX297904 | A/environment/Korea/W477/2014(H5N8) | 100% | 100.00% |
|  | ma468-G4-2 | ID0080 | A/ma468-G4-2/2014(H5N8) | 8 |  | KX297970 | A/environment/Korea/W468/2014(H5N8) | 100% | 100.00% |
| 45 | NL230 | EPI319935 | A/Netherlands/230/2003(H7N7) | 1 |  | EF015558 | A/chicken/Netherlands/03010132/03(H7N7) | 100% | 99.69% |
|  | NL230 | EPI319937 | A/Netherlands/230/2003(H7N7) | 4 |  | AB438941 | A/chicken/Netherlands/2586/2003(H7N7) | 100% | 99.94% |
|  | NL230 | EPI319936 | A/Netherlands/230/2003(H7N7) | 6 |  | AB438943 | A/chicken/Netherlands/2586/2003(H7N7) | 100% | 99.93% |
| 46 | NY107 | EU587371 | A/NewYork/107/2003(H7N2) | 5 |  | CY031654 | A/guinea fowl/New York/22071/2005(H7N2) | 100% | 99.59% |
| 47 | OH02 | EPI291908 | A/Ohio/02/2007(H1N1) | 1 |  | EU604691 | A/swine/OH/511445/2007(H1N1) | 100% | 99.96% |
|  | OH02 | EPI338828 | A/Ohio/02/2007(H1N1) | 6 |  | EU604690 | A/swine/OH/511445/2007(H1N1) | 100% | 100.00% |
| 48 | SHANG13 | KC853225 | A/Shanghai/4664T/2013(H7N9) | 5 |  | MF988739 | A/duck/Anhui/S702/2013(H7N9) | 100% | 100.00% |
| 49 | STE10 | KF897796 | A/StEtienne/1139/2010(H1N1) | 7 |  | JX625745 | A/England/182/2010(H1N1) | 100% | 100.00% |
| 50 | SWE1021 | GQ495134 | A/swine/Sweden/1021/2009(H1N2) | 6 |  | KR066629 | A/swine/Denmark/10832-1/2009(H1N2) | 100% | 99.98% |
|  | SWE1021 | GQ495135 | A/swine/Sweden/1021/2009(H1N2) | 7 |  | KR700052 | A/swine/Denmark/10-1725-1/2011(H1N2) | 100% | 98.47% |
| 51 | SWE9706 | HM626484 | A/swine/Sweden/9706/2010(H1N2) | 6 |  | KR066629 | A/swine/Denmark/10832-1/2009(H1N2) | 100% | 98.02% |
|  | SWE9706 | HM626485 | A/swine/Sweden/9706/2010(H1N2) | 7 |  | KR700052 | A/swine/Denmark/10-1725-1/2011(H1N2) | 100% | 98.47% |
| 52 | THAI16v2 | EF473503 | A/Thailand/16/2004(H5N1) | 3 |  | EU268218 | A/Thailand/16/2004(H5N1) | 100% | 99.91% |
| 53 | TN560 | CY040457 | A/Tennessee/1-560/2009(H1N1) | 4 |  | KY926119 | A/Teutonia/LACENRS-711/2009(H1N1) | 100% | 99.94% |
|  | TN560 | CY040458 | A/Tennessee/1-560/2009(H1N1) | 6 |  | KY925637 | A/Porto Alegre/LACENRS-1786/2009(H1N1) | 100% | 100.00% |
| 54 | VN03 | DQ492869 | A/chicken/VietNam/8/2003(H5N1) | 1 |  | CY034205 | A/chicken/Vietnam/18/2004(H5N1) | 100% | 99.71% |
|  | VN03 | DQ493393 | A/chicken/VietNam/8/2003(H5N1) | 2 |  | CY028706 | A/chicken/Vietnam/10/2004(H5N1) | 100% | 99.73% |
|  | VN03 | DQ493306 | A/chicken/VietNam/8/2003(H5N1) | 3 | Remove the first 24 nts (low quality) | HM627920 | A/openbill stork/Thailand/VSMU-5-NSN/2004(H5N1) | 100% | 99.51% |
|  | VN03 | DQ497693 | A/chicken/VietNam/8/2003(H5N1) | 4 |  | AB440326 | A/quail/Angthong/71/2004(H5N1) | 100% | 99.41% |
|  | VN03 | DQ493131 | A/chicken/VietNam/8/2003(H5N1) | 5 |  | AY627895 | A/Thailand/2(SP-33)/2004(H5N1) | 100% | 100.00% |
|  | VN03 | DQ492954 | A/chicken/VietNam/8/2003(H5N1) | 7 |  | EF541449 | A/chicken/Viet Nam/1/2004(H5N1) | 100% | 100.00% |
|  | VN03 | DQ493218 | A/chicken/VietNam/8/2003(H5N1) | 8 |  | AY651555 | A/Viet Nam/3062/2004(H5N1) | 100% | 99.63% |
| 55 | VN1204 | EF473407 | A/VietNam/1204/2004(H5N1) | 3 |  | HM006758 | A/Viet Nam/1203/2004(H5N1) | 100% | 99.91% |
| 56 | w81 | GU361156 | A/aquaticbird/Korea/w81/2005(H5N2) | 5 |  | HM145070 | A/mallard/Jiangxi/10071/2005(H6N1) | 100% | 99.66% |
