# Supplementary material for: Rule-based meta-analysis reveals the major role of PB2 in influencing influenza A virus virulence in mice
Source: BMC Genomics. 2019 Dec 24;20(Suppl 9):973. doi: 10.1186/s12864-019-6295-8 (PMC6929465; doi:10.1186/s12864-019-6295-8)
Supplement: Supplementary file 13 — Additional file 13: Table S9. Examples of rules generated by OneR, JRip and PART for two-class and three-class MIV datasets containing concatenated alignments of IAV proteins. [file 12864_2019_6295_MOESM13_ESM.docx]

**Table S9.** Examples of rules generated by OneR (1R), JRip (JR) and PART (PT) for (A) two-class and (B) three-class MIV datasets containing concatenated alignments of IAV proteins. For the values of the predictor or protein site (displayed as [protein name].[position]), the first letter indicates the amino acid or gap presents at the site and the second letter indicates the mouse strain (a for CD-1, b for BALB/C, c for C57BL/6, d for DBA/2, e for FVB/NJ, f for A/J, g for 129S1/SvImJ, h for NOD/ShiLtJ, i for NZO/HILtJ, j for CAST/EiJ, k for PWK/PhJ, l for WSB/EiJ, m for CBA/J, n for SJL/JOrlCrl, o for C3H and p for 129S1/SvPasCrlVr).

(A) Two-class MIV dataset

| **Method** | **Rule(s)** | **Summary** |
| --- | --- | --- |
| 1R | PB1-F2.87:  -b -> Avirulent  -c -> Virulent  -d -> Virulent  -e -> Avirulent  -f -> Avirulent  -m -> Virulent  -n -> Avirulent  -o -> Avirulent  -p -> Avirulent  Eb -> Virulent  Ec -> Virulent  Ed -> Virulent  Ee -> Avirulent  Ef -> Avirulent  Em -> Virulent  En -> Avirulent  Eo -> Virulent  Ep -> Virulent  Eq -> Avirulent  Ga -> Avirulent  Gb -> Avirulent  Gc -> Avirulent  Gd -> Avirulent  Gf -> Avirulent  Gg -> Avirulent  Gh -> Avirulent  Gi -> Avirulent  Gj -> Avirulent  Gk -> Avirulent  Gl -> Avirulent  (180/276 instances correct) | === Summary ===  Correctly Classified Instances 151 54.7101 %  Incorrectly Classified Instances 125 45.2899 %  Kappa statistic 0.0942  Mean absolute error 0.4529  Root mean squared error 0.673  Relative absolute error 90.5797 %  Root relative squared error 134.5955 %  Total Number of Instances 276  === Confusion Matrix ===  a b <-- classified as  62 76 \| a = Avirulent  49 89 \| b = Virulent |
| JR | JRIP rules:  ===========  (PB1-F2.30 = Lb) => Vir_two_classes=Avirulent (120.0/57.0)  => Vir_two_classes=Virulent (156.0/75.0)  Number of Rules : 2 | === Summary ===  Correctly Classified Instances 144 52.1739 %  Incorrectly Classified Instances 132 47.8261 %  Kappa statistic 0.0435  Mean absolute error 0.499  Root mean squared error 0.4995  Relative absolute error 99.8077 %  Root relative squared error 99.9038 %  Total Number of Instances 276  === Confusion Matrix ===  a b <-- classified as  63 75 \| a = Avirulent  57 81 \| b = Virulent |
| PT | PART decision list  ------------------  PB1-F2.55 = Tc: Avirulent (18.0/2.0)  PB1-F2.87 = Gd: Avirulent (10.0/1.0)  PB1-F2.34 = -b: Avirulent (28.0/12.0)  PB1-F2.34 = Nc: Virulent (25.0/9.0)  PB1-F2.34 = Na: Avirulent (15.0/7.0)  PB1-F2.34 = -c: Virulent (8.0)  PB1-F2.34 = Nb AND  PB1-F2.7 = Tb AND  PB1-F2.41 = Hb AND  PB1-F2.25 = Qb AND  PB1-F2.62 = -b AND  PB1-F2.2 = Gb: Avirulent (8.0/3.0)  PB1-F2.34 = Nb AND  PB1-F2.7 = Tb AND  PB1-F2.41 = Hb AND  PB1-F2.85 = Kb AND  PB1-F2.74 = Tb AND  PB1-F2.73 = Kb AND  PB1-F2.59 = Kb AND  PB1-F2.71 = Sb AND  PB1-F2.14 = Eb AND  PB1-F2.2 = Eb AND  PB1-F2.63 = Sb AND  PB1-F2.30 = Lb AND  PB1-F2.23 = Nb AND  PB1-F2.29 = Kb: Virulent (26.0/12.0)  PB1-F2.34 = Nb AND  PB1-F2.7 = Tb AND  PB1-F2.25 = Qb AND  PB1-F2.41 = Hb AND  PB1-F2.85 = Kb AND  PB1-F2.62 = Lb AND  PB1-F2.59 = Kb AND  PB1-F2.71 = Sb AND  PB1-F2.14 = Eb AND  PB1-F2.2 = Eb AND  PB1-F2.28 = Qb AND  PB1-F2.42 = Yb AND  PB1-F2.75 = Hb: Virulent (37.0/14.0)  PB1-F2.30 = Lb AND  PB1-F2.6 = Db AND  PB1-F2.62 = Lb AND  PB1-F2.34 = Nb AND  PB1-F2.41 = Hb AND  PB1-F2.63 = Sb AND  PB1-F2.75 = Rb AND  PB1-F2.49 = Vb AND  PB1-F2.79 = Rb AND  PB1-F2.22 = Eb: Avirulent (20.0/7.0)  PB1-F2.34 = Nb AND  PB1-F2.26 = Qb AND  PB1-F2.2 = Eb AND  PB1-F2.30 = Lb AND  PB1-F2.59 = Kb AND  PB1-F2.36 = Tb AND  PB1-F2.23 = Sb: Virulent (24.0/10.0)  PB1-F2.55 = Ib AND  PB1-F2.27 = Tb AND  PB1-F2.20 = Kb AND  PB1-F2.36 = Tb: Virulent (11.0)  PB1-F2.77 = Lb: Avirulent (16.0/1.0)  PB1-F2.54 = Qd: Virulent (9.0)  PB1-F2.42 = Ce: Avirulent (4.0)  PB1-F2.2 = Gb: Avirulent (4.0)  : Virulent (13.0/3.0)  Number of Rules : 17 | === Summary ===  Correctly Classified Instances 140 50.7246 %  Incorrectly Classified Instances 136 49.2754 %  Kappa statistic 0.0145  Mean absolute error 0.4922  Root mean squared error 0.6091  Relative absolute error 98.44 %  Root relative squared error 121.8159 %  Total Number of Instances 276  === Confusion Matrix ===  a b <-- classified as  86 52 \| a = Avirulent  84 54 \| b = Virulent |

(B) Three-class MIV dataset

| **Method** | **Rule(s)** | **Summary** |
| --- | --- | --- |
| 1R | NS2.22:  -b -> LOW  Ab -> INTERMEDIATE  Ac -> HIGH  Ae -> HIGH  Eb -> HIGH  Ec -> INTERMEDIATE  Ed -> HIGH  Ee -> LOW  Ef -> HIGH  Em -> INTERMEDIATE  En -> LOW  Eo -> HIGH  Ep -> HIGH  Ga -> INTERMEDIATE  Gb -> LOW  Gc -> INTERMEDIATE  Gd -> INTERMEDIATE  Ge -> HIGH  Gf -> HIGH  Gg -> HIGH  Gh -> HIGH  Gi -> HIGH  Gj -> HIGH  Gk -> LOW  Gl -> HIGH  Rb -> INTERMEDIATE  Rc -> INTERMEDIATE  Rq -> HIGH  (178/366 instances correct) | === Summary ===  Correctly Classified Instances 116 31.694 %  Incorrectly Classified Instances 250 68.306 %  Kappa statistic -0.0246  Mean absolute error 0.4554  Root mean squared error 0.6748  Relative absolute error 102.459 %  Root relative squared error 143.1496 %  Total Number of Instances 366  === Confusion Matrix ===  a b c <-- classified as  27 81 14 \| a = HIGH  32 74 16 \| b = INTERMEDIATE  22 85 15 \| c = LOW |
| JR | JRIP rules:  ===========  (NS2.107 = Ld) => Vir_three_classes=HIGH (18.0/7.0)  (NS2.49 = Ib) => Vir_three_classes=HIGH (15.0/6.0)  (NS2.70 = Sc) => Vir_three_classes=HIGH (20.0/9.0)  => Vir_three_classes=INTERMEDIATE (313.0/199.0)  Number of Rules : 4 | === Summary ===  Correctly Classified Instances 123 33.6066 %  Incorrectly Classified Instances 243 66.3934 %  Kappa statistic 0.0041  Mean absolute error 0.4446  Root mean squared error 0.4734  Relative absolute error 100.042 %  Root relative squared error 100.4189 %  Total Number of Instances 366  === Confusion Matrix ===  a b c <-- classified as  2 120 0 \| a = HIGH  1 121 0 \| b = INTERMEDIATE  2 120 0 \| c = LOW |
| PT | PART decision list  ------------------  NS2.22 = Ab: INTERMEDIATE (24.0/9.0)  NS2.57 = Yc: INTERMEDIATE (11.0)  NS2.22 = Ec: INTERMEDIATE (21.0/8.0)  NS2.4 = Hb AND  NS2.76 = Ib: INTERMEDIATE (9.0/4.0)  NS2.26 = Kc: LOW (7.0/2.0)  NS2.4 = Nc AND  NS2.49 = Vc AND  NS2.40 = Lc AND  NS2.22 = Gc AND  NS2.60 = Nc AND  NS2.89 = Ic: HIGH (10.0/2.0)  NS2.4 = Na: INTERMEDIATE (18.0/10.0)  NS2.4 = Nc AND  NS2.22 = Gc: LOW (10.0/3.0)  NS2.4 = Hd: INTERMEDIATE (4.0)  NS2.55 = Ld AND  NS2.27 = Dd AND  NS2.70 = Gd AND  NS2.107 = Ld: HIGH (15.0/4.0)  NS2.55 = Ld AND  NS2.40 = Id: LOW (5.0)  NS2.55 = Fc: HIGH (3.0)  NS2.64 = Kd AND  NS2.14 = Ld: INTERMEDIATE (3.0)  NS2.64 = Kb AND  NS2.20 = Qb AND  NS2.7 = Sb AND  NS2.37 = Sb AND  NS2.52 = Vb: HIGH (15.0/6.0)  NS2.2 = Db AND  NS2.64 = Kb AND  NS2.20 = Qb AND  NS2.7 = Sb AND  NS2.52 = Mb AND  NS2.92 = -b: LOW (12.0/4.0)  NS2.64 = Kb AND  NS2.2 = Db AND  NS2.71 = Qb AND  NS2.4 = Nb AND  NS2.20 = Qb AND  NS2.52 = Vb: LOW (8.0/3.0)  NS2.64 = Kc: INTERMEDIATE (7.0/2.0)  NS2.64 = Kb AND  NS2.4 = Nb AND  NS2.2 = Db AND  NS2.71 = Qb AND  NS2.20 = Rb: LOW (6.0/1.0)  NS2.64 = Kb AND  NS2.4 = Nb AND  NS2.2 = Db AND  NS2.71 = Qb AND  NS2.42 = Rb AND  NS2.7 = Lb: INTERMEDIATE (5.0/2.0)  NS2.64 = Kb AND  NS2.4 = Nb AND  NS2.2 = Db AND  NS2.71 = Qb AND  NS2.42 = Rb AND  NS2.27 = Gb AND  NS2.14 = Mb: HIGH (5.0/2.0)  NS2.64 = Kb AND  NS2.4 = Nb AND  NS2.27 = Db AND  NS2.2 = Db AND  NS2.71 = Qb AND  NS2.42 = Rb AND  NS2.26 = Eb AND  NS2.107 = Lb AND  NS2.31 = Ib AND  NS2.3 = Sb: HIGH (7.0/3.0)  NS2.56 = Hb AND  NS2.64 = Kb AND  NS2.4 = Nb AND  NS2.27 = Db AND  NS2.42 = Rb AND  NS2.31 = Mb AND  NS2.37 = Sb AND  NS2.26 = Eb AND  NS2.107 = Lb AND  NS2.49 = Vb AND  NS2.14 = Kb: LOW (17.0/9.0)  NS2.56 = Hb AND  NS2.64 = Kb AND  NS2.4 = Nb AND  NS2.27 = Db AND  NS2.42 = Rb AND  NS2.31 = Mb AND  NS2.88 = Kb AND  NS2.26 = Eb AND  NS2.107 = Lb AND  NS2.49 = Vb AND  NS2.60 = Nb AND  NS2.14 = Mb: HIGH (8.0/2.0)  NS2.56 = Hb AND  NS2.64 = Kb AND  NS2.4 = Nb AND  NS2.27 = Db AND  NS2.42 = Rb AND  NS2.31 = Mb AND  NS2.88 = Kb AND  NS2.37 = Cb: HIGH (7.0/2.0)  NS2.56 = Hb AND  NS2.37 = Sb AND  NS2.4 = Nb AND  NS2.27 = Db AND  NS2.31 = Mb AND  NS2.42 = Rb AND  NS2.88 = Kb AND  NS2.26 = Eb AND  NS2.107 = Lb AND  NS2.49 = Vb AND  NS2.14 = Lb AND  NS2.89 = Ib: LOW (6.0/3.0)  NS2.56 = Hb AND  NS2.37 = Sb AND  NS2.4 = Nb AND  NS2.27 = Db AND  NS2.42 = Rb AND  NS2.31 = Mb AND  NS2.60 = Sb AND  NS2.14 = Mb: LOW (69.0/42.0)  NS2.56 = Hb AND  NS2.37 = Sb AND  NS2.4 = Nb AND  NS2.27 = Db AND  NS2.42 = Rb AND  NS2.60 = Nb AND  NS2.22 = Gb AND  NS2.3 = Sb: HIGH (5.0/2.0)  NS2.56 = Hb AND  NS2.3 = Sb AND  NS2.49 = Vb: LOW (12.0/2.0)  NS2.34 = Qb AND  NS2.56 = Hb: INTERMEDIATE (14.0/7.0)  NS2.14 = Kb: HIGH (4.0)  NS2.1 = Md: LOW (3.0)  NS2.1 = Mf: HIGH (3.0)  : LOW (13.0/6.0)  Number of Rules : 33 | === Summary ===  Correctly Classified Instances 148 40.4372 %  Incorrectly Classified Instances 218 59.5628 %  Kappa statistic 0.1066  Mean absolute error 0.3985  Root mean squared error 0.48  Relative absolute error 89.6621 %  Root relative squared error 101.8143 %  Total Number of Instances 366  === Confusion Matrix ===  a b c <-- classified as  12 87 23 \| a = HIGH  2 104 16 \| b = INTERMEDIATE  1 89 32 \| c = LOW |
