# Supplementary material for: Rule-based meta-analysis reveals the major role of PB2 in influencing influenza A virus virulence in mice
Source: BMC Genomics. 2019 Dec 24;20(Suppl 9):973. doi: 10.1186/s12864-019-6295-8 (PMC6929465; doi:10.1186/s12864-019-6295-8)
Supplement: Supplementary file 14 — Additional file 14: Table S10. Examples of rules generated by OneR, JRip and PART for two-class and three-class IV datasets containing concatenated alignments of IAV proteins. [file 12864_2019_6295_MOESM14_ESM.docx]

**Table S10.** Examples of rules generated by OneR (1R), JRip (JR) and PART (PT) for (A) two-class and (B) three-class IV datasets containing concatenated alignments of IAV proteins. The predictor or protein site is displayed as [protein name].[position].

(A) Two-class IV dataset

| **Method** | **Rule(s)** | **Summary** |
| --- | --- | --- |
| 1R | PB1-F2.37:  - -> Virulent  L -> Virulent  Q -> Avirulent  R -> Virulent  (133/226 instances correct) | === Summary ===  Correctly Classified Instances 133 58.8496 %  Incorrectly Classified Instances 93 41.1504 %  Kappa statistic 0.177  Mean absolute error 0.4115  Root mean squared error 0.6415  Relative absolute error 82.3009 %  Root relative squared error 128.2972 %  Total Number of Instances 226  === Confusion Matrix ===  a b <-- classified as  53 60 \| a = Avirulent  33 80 \| b = Virulent |
| JR | JRIP rules:  ===========  (PB1-F2.37 = Q) => Vir_two_classes=Avirulent (86.0/33.0)  (PB1-F2.70 = G) and (PB1-F2.50 = G) => Vir_two_classes=Avirulent (26.0/8.0)  => Vir_two_classes=Virulent (114.0/42.0)  Number of Rules : 3 | === Summary ===  Correctly Classified Instances 133 58.8496 %  Incorrectly Classified Instances 93 41.1504 %  Kappa statistic 0.177  Mean absolute error 0.4781  Root mean squared error 0.4939  Relative absolute error 95.6131 %  Root relative squared error 98.7797 %  Total Number of Instances 226  === Confusion Matrix ===  a b <-- classified as  53 60 \| a = Avirulent  33 80 \| b = Virulent |
| PT | PART decision list  ------------------  PB1-F2.27 = - AND  PB1-F2.75 = -: Virulent (36.0/15.0)  PB1-F2.27 = T AND  PB1-F2.74 = T AND  PB1-F2.34 = N AND  PB1-F2.85 = K AND  PB1-F2.51 = M AND  PB1-F2.62 = L AND  PB1-F2.73 = K AND  PB1-F2.76 = V AND  PB1-F2.2 = E AND  PB1-F2.81 = K AND  PB1-F2.78 = K AND  PB1-F2.63 = S AND  PB1-F2.30 = L AND  PB1-F2.89 = I AND  PB1-F2.42 = Y: Avirulent (27.0/6.0)  PB1-F2.27 = T AND  PB1-F2.4 = E AND  PB1-F2.62 = L AND  PB1-F2.78 = K AND  PB1-F2.67 = P AND  PB1-F2.85 = K AND  PB1-F2.3 = Q AND  PB1-F2.16 = I AND  PB1-F2.73 = K AND  PB1-F2.31 = E AND  PB1-F2.32 = H AND  PB1-F2.56 = V AND  PB1-F2.59 = K AND  PB1-F2.40 = D AND  PB1-F2.42 = Y: Virulent (39.0/15.0)  PB1-F2.48 = Q AND  PB1-F2.74 = T AND  PB1-F2.85 = K AND  PB1-F2.73 = K AND  PB1-F2.76 = V AND  PB1-F2.83 = F AND  PB1-F2.53 = K AND  PB1-F2.4 = E AND  PB1-F2.15 = H AND  PB1-F2.77 = L AND  PB1-F2.22 = G: Avirulent (35.0/14.0)  PB1-F2.27 = I: Avirulent (16.0/1.0)  PB1-F2.4 = E AND  PB1-F2.15 = H AND  PB1-F2.32 = H AND  PB1-F2.55 = I AND  PB1-F2.57 = Y AND  PB1-F2.62 = L AND  PB1-F2.80 = W AND  PB1-F2.59 = K AND  PB1-F2.42 = C: Virulent (15.0/3.0)  PB1-F2.41 = H AND  PB1-F2.48 = Q AND  PB1-F2.21 = R AND  PB1-F2.83 = F AND  PB1-F2.20 = K: Avirulent (17.0/3.0)  PB1-F2.41 = H AND  PB1-F2.26 = Q: Virulent (27.0)  PB1-F2.79 = R AND  PB1-F2.75 = R: Avirulent (6.0/2.0)  PB1-F2.46 = M: Avirulent (5.0)  : Virulent (3.0)  Number of Rules : 11 | === Summary ===  Correctly Classified Instances 150 66.3717 %  Incorrectly Classified Instances 76 33.6283 %  Kappa statistic 0.3274  Mean absolute error 0.3859  Root mean squared error 0.5059  Relative absolute error 77.1821 %  Root relative squared error 101.1871 %  Total Number of Instances 226  === Confusion Matrix ===  a b <-- classified as  47 66 \| a = Avirulent  10 103 \| b = Virulent |

(B) Three-class IV dataset

| **Method** | **Rule(s)** | **Summary** |
| --- | --- | --- |
| 1R | PB1-F2.70:  - -> INTERMEDIATE  A -> HIGH  D -> HIGH  E -> INTERMEDIATE  G -> LOW  V -> HIGH  (130/309 instances correct) | === Summary ===  Correctly Classified Instances 95 30.7443 %  Incorrectly Classified Instances 214 69.2557 %  Kappa statistic -0.0388  Mean absolute error 0.4617  Root mean squared error 0.6795  Relative absolute error 103.8835 %  Root relative squared error 144.1412 %  Total Number of Instances 309  === Confusion Matrix ===  a b c <-- classified as  20 67 16 \| a = HIGH  22 67 14 \| b = INTERMEDIATE  20 75 8 \| c = LOW |
| JR | JRIP rules:  ===========  (PB1-F2.37 = Q) and (PB1-F2.45 = T) => Vir_three_classes=LOW (31.0/13.0)  (PB1-F2.23 = N) and (PB1-F2.37 = R) => Vir_three_classes=LOW (16.0/5.0)  => Vir_three_classes=HIGH (262.0/165.0)  Number of Rules : 3 | === Summary ===  Correctly Classified Instances 126 40.7767 %  Incorrectly Classified Instances 183 59.2233 %  Kappa statistic 0.1117  Mean absolute error 0.4285  Root mean squared error 0.4629  Relative absolute error 96.4236 %  Root relative squared error 98.1955 %  Total Number of Instances 309  === Confusion Matrix ===  a b c <-- classified as  97 0 6 \| a = HIGH  91 0 12 \| b = INTERMEDIATE  74 0 29 \| c = LOW |
| PT | PART decision list  ------------------  PB1-F2.74 = - AND  PB1-F2.2 = -: INTERMEDIATE (50.0/30.0)  PB1-F2.51 = M AND  PB1-F2.74 = - AND  PB1-F2.2 = G: LOW (9.0/3.0)  PB1-F2.63 = -: INTERMEDIATE (8.0/3.0)  PB1-F2.63 = S AND  PB1-F2.74 = P: INTERMEDIATE (6.0/2.0)  PB1-F2.63 = S AND  PB1-F2.74 = I: INTERMEDIATE (4.0/1.0)  PB1-F2.63 = S AND  PB1-F2.39 = M AND  PB1-F2.27 = - AND  PB1-F2.46 = M: LOW (4.0/1.0)  PB1-F2.27 = T AND  PB1-F2.51 = M AND  PB1-F2.80 = W AND  PB1-F2.78 = K AND  PB1-F2.15 = H AND  PB1-F2.85 = K AND  PB1-F2.73 = K AND  PB1-F2.79 = R AND  PB1-F2.64 = L AND  PB1-F2.59 = K AND  PB1-F2.71 = S AND  PB1-F2.69 = Q AND  PB1-F2.2 = E AND  PB1-F2.86 = R AND  PB1-F2.75 = H AND  PB1-F2.45 = I AND  PB1-F2.6 = D: HIGH (20.0/5.0)  PB1-F2.63 = S AND  PB1-F2.27 = I: LOW (4.0)  PB1-F2.63 = S AND  PB1-F2.8 = - AND  PB1-F2.1 = M: HIGH (3.0/1.0)  PB1-F2.63 = S AND  PB1-F2.8 = P AND  PB1-F2.51 = T: LOW (7.0/2.0)  PB1-F2.63 = S AND  PB1-F2.8 = P AND  PB1-F2.85 = - AND  PB1-F2.2 = G: INTERMEDIATE (3.0/1.0)  PB1-F2.63 = S AND  PB1-F2.8 = P AND  PB1-F2.85 = K AND  PB1-F2.73 = K AND  PB1-F2.78 = K AND  PB1-F2.15 = H AND  PB1-F2.79 = R AND  PB1-F2.64 = S: HIGH (15.0/6.0)  PB1-F2.63 = S AND  PB1-F2.34 = N AND  PB1-F2.85 = K AND  PB1-F2.78 = K AND  PB1-F2.73 = K AND  PB1-F2.15 = H AND  PB1-F2.79 = R AND  PB1-F2.59 = K AND  PB1-F2.71 = S AND  PB1-F2.69 = Q AND  PB1-F2.2 = E AND  PB1-F2.75 = R AND  PB1-F2.6 = D AND  PB1-F2.46 = M AND  PB1-F2.18 = I AND  PB1-F2.36 = T AND  PB1-F2.4 = E: INTERMEDIATE (30.0/16.0)  PB1-F2.63 = S AND  PB1-F2.8 = P AND  PB1-F2.85 = K AND  PB1-F2.78 = K AND  PB1-F2.73 = K AND  PB1-F2.15 = H AND  PB1-F2.79 = R AND  PB1-F2.59 = R: HIGH (12.0/6.0)  PB1-F2.63 = S AND  PB1-F2.8 = P AND  PB1-F2.85 = K AND  PB1-F2.87 = E AND  PB1-F2.15 = H AND  PB1-F2.79 = R AND  PB1-F2.2 = G AND  PB1-F2.66 = N AND  PB1-F2.22 = Q: INTERMEDIATE (10.0/4.0)  PB1-F2.88 = W AND  PB1-F2.63 = S AND  PB1-F2.8 = P AND  PB1-F2.78 = K AND  PB1-F2.29 = K AND  PB1-F2.20 = K: LOW (7.0/1.0)  PB1-F2.88 = W AND  PB1-F2.63 = S AND  PB1-F2.35 = S AND  PB1-F2.78 = K AND  PB1-F2.15 = H AND  PB1-F2.79 = R AND  PB1-F2.2 = E AND  PB1-F2.75 = R: LOW (38.0/15.0)  PB1-F2.78 = K AND  PB1-F2.88 = W AND  PB1-F2.63 = S AND  PB1-F2.55 = I AND  PB1-F2.29 = R AND  PB1-F2.15 = H AND  PB1-F2.56 = V AND  PB1-F2.4 = E AND  PB1-F2.2 = E: INTERMEDIATE (39.0/21.0)  PB1-F2.78 = K AND  PB1-F2.3 = Q AND  PB1-F2.87 = E AND  PB1-F2.4 = E AND  PB1-F2.16 = I: HIGH (19.0/6.0)  PB1-F2.78 = K AND  PB1-F2.56 = V AND  PB1-F2.70 = G: HIGH (13.0/4.0)  PB1-F2.78 = K: INTERMEDIATE (6.0/1.0)  : LOW (2.0)  Number of Rules : 22 | === Summary ===  Correctly Classified Instances 175 56.6343 %  Incorrectly Classified Instances 134 43.3657 %  Kappa statistic 0.3495  Mean absolute error 0.359  Root mean squared error 0.4313  Relative absolute error 80.7721 %  Root relative squared error 91.4941 %  Total Number of Instances 309  === Confusion Matrix ===  a b c <-- classified as  45 52 6 \| a = HIGH  6 81 16 \| b = INTERMEDIATE  18 36 49 \| c = LOW |
