# Supplementary material for: Rule-based meta-analysis reveals the major role of PB2 in influencing influenza A virus virulence in mice
Source: BMC Genomics. 2019 Dec 24;20(Suppl 9):973. doi: 10.1186/s12864-019-6295-8 (PMC6929465; doi:10.1186/s12864-019-6295-8)
Supplement: Supplementary file 15 — Additional file 15: Table S11. Examples of rules generated by OneR, JRip and PART for two-class and three-class BALB/C datasets containing concatenated alignments of IAV proteins. [file 12864_2019_6295_MOESM15_ESM.docx]

**Table S11.** Examples of rules generated by OneR (1R), JRip (JR) and PART (PT) for (A) two-class and (B) three-class BALB/C datasets containing concatenated alignments of IAV proteins. For the values of the predictor or protein site (displayed as [protein name].[position]), the first letter indicates the amino acid or gap presents at the site and the second letter b indicates the mouse strain BALB/C.

(A) Two-class BALB/C dataset

| **Method** | **Rule(s)** | **Summary** |
| --- | --- | --- |
| 1R | HA.196:  Ab -> Avirulent  Gb -> Avirulent  Hb -> Avirulent  Ib -> Virulent  Kb -> Virulent  Qb -> Virulent  Rb -> Avirulent  Sb -> Avirulent  Tb -> Avirulent  Vb -> Virulent  (129/188 instances correct) | === Summary ===  Correctly Classified Instances 129 68.617 %  Incorrectly Classified Instances 59 31.383 %  Kappa statistic 0.3723  Mean absolute error 0.3138  Root mean squared error 0.5602  Relative absolute error 62.766 %  Root relative squared error 112.041 %  Total Number of Instances 188  === Confusion Matrix ===  a b <-- classified as  40 54 \| a = Avirulent  5 89 \| b = Virulent |
| JR | JRIP rules:  ===========  (HA.323 = Vb) => Vir_two_classes=Avirulent (65.0/22.0)  (HA.138 = Sb) => Vir_two_classes=Avirulent (14.0/1.0)  => Vir_two_classes=Virulent (109.0/38.0)  Number of Rules : 3 | === Summary ===  Correctly Classified Instances 106 56.383 %  Incorrectly Classified Instances 82 43.617 %  Kappa statistic 0.1277  Mean absolute error 0.463  Root mean squared error 0.4979  Relative absolute error 92.5964 %  Root relative squared error 99.5727 %  Total Number of Instances 188  === Confusion Matrix ===  a b <-- classified as  13 81 \| a = Avirulent  1 93 \| b = Virulent |
| PT | PART decision list  ------------------  M1.219 = Ib AND  PB2.80 = Kb AND  PB2.153 = Db AND  NS1.42 = Sb AND  PB2.110 = Hb AND  M1.215 = Ab AND  PB2.62 = Rb AND  PB1.181 = Ib AND  PB1.678 = Sb AND  PB2.267 = Vb AND  HA.429 = Vb AND  NS2.67 = Eb AND  NA.170 = Nb AND  NP.492 = Nb AND  HA.474 = Db AND  PB2.526 = Rb: Virulent (17.0)  PA.97 = Tb AND  PB2.250 = Vb AND  PB2.526 = Rb: Avirulent (13.0)  PA.97 = Tb AND  PB2.250 = Vb AND  NS1.27 = Lb AND  PB2.461 = Ib AND  HA.502 = Ib AND  PB2.63 = Ib AND  PB2.80 = Kb AND  PB2.153 = Db AND  PB1.180 = Eb AND  HA.194 = Lb AND  PB1.678 = Sb AND  M1.215 = Ab AND  PB2.267 = Vb AND  PB1.181 = Ib AND  HA.429 = Vb AND  NS2.67 = Eb AND  NP.492 = Nb AND  PB2.108 = Tb AND  M2.66 = Eb AND  PA.129 = Ib AND  NP.101 = Db AND  PB2.701 = Db AND  NA.11 = Gb AND  PA.529 = Db AND  PA.581 = Mb AND  HA.17 = Yb AND  HA.227 = Sb AND  HA.327a = Rb AND  PB2.627 = Kb: Virulent (11.0)  PA.97 = Tb AND  PB2.185 = Ib AND  PB2.461 = Ib AND  HA.502 = Ib AND  PB2.2 = Eb AND  PB1.756 = Qb AND  PA.529 = Db AND  NP.41 = Ib AND  NP.492 = Nb AND  NS1.180 = Vb AND  NS1.166 = Lb AND  NA.259 = Nb AND  PB2.63 = Ib AND  PB2.80 = Kb AND  PA.142 = Kb AND  PB1.180 = Eb AND  NP.101 = Db AND  HA.194 = Lb AND  PB2.539 = Ib AND  NS1.123 = Ib AND  PB1.678 = Sb AND  PB2.701 = Db AND  HA.88 = Vb AND  PA.327 = Eb AND  NA.132 = Ib AND  PA.581 = Mb AND  M1.215 = Ab AND  PB2.108 = Tb AND  HA.138 = Ab AND  NA.398 = Sb AND  NP.16 = Gb AND  HA.227 = Sb: Virulent (11.0/4.0)  PA.97 = Tb AND  PB2.185 = Ib AND  PB2.461 = Ib AND  HA.502 = Ib AND  PB2.2 = Eb AND  PB1.756 = Qb AND  PA.529 = Db AND  NP.41 = Ib AND  NP.492 = Nb AND  NS1.116 = Cb AND  NA.266 = Mb AND  NP.101 = Db AND  NP.473 = Nb AND  PB2.701 = Db AND  NS2.90 = Tb AND  HA.121b = Ib AND  NS2.31 = Mb AND  PA.142 = Kb AND  NA.132 = Lb AND  PB2.627 = Eb: Avirulent (12.0)  PB2.6 = Eb AND  PB2.185 = Ib AND  PB2.461 = Ib AND  HA.502 = Ib AND  PA.712 = Tb AND  PA.529 = Db AND  NP.41 = Ib AND  NP.492 = Nb AND  NS1.123 = Ib AND  NP.101 = Db AND  NP.473 = Nb AND  PB2.701 = Db AND  NA.196 = Sb AND  NA.132 = Ib AND  NS1.116 = Cb AND  PB1.317 = Mb: Avirulent (51.0/8.0)  PB2.110 = Hb AND  NS1.211 = Rb AND  PB1.678 = Sb AND  HA.474 = Db AND  NP.450 = Sb AND  NP.492 = Nb: Virulent (48.0/2.0)  NP.492 = Nb AND  PB2.701 = Db: Avirulent (17.0)  NP.492 = Ib: Virulent (3.0)  PB1.13 = Pb: Avirulent (3.0)  : Virulent (2.0)  Number of Rules : 11 | === Summary ===  Correctly Classified Instances 116 61.7021 %  Incorrectly Classified Instances 72 38.2979 %  Kappa statistic 0.234  Mean absolute error 0.4063  Root mean squared error 0.6034  Relative absolute error 81.2683 %  Root relative squared error 120.6866 %  Total Number of Instances 188  === Confusion Matrix ===  a b <-- classified as  94 0 \| a = Avirulent  72 22 \| b = Virulent |

(B) Three-class BALB/C dataset

| **Method** | **Rule(s)** | **Summary** |
| --- | --- | --- |
| 1R | PA.272:  Cb -> LOW  Db -> HIGH  Eb -> INTERMEDIATE  Nb -> LOW  (95/234 instances correct) | === Summary ===  Correctly Classified Instances 95 40.5983 %  Incorrectly Classified Instances 139 59.4017 %  Kappa statistic 0.109  Mean absolute error 0.396  Root mean squared error 0.6293  Relative absolute error 89.1026 %  Root relative squared error 133.4935 %  Total Number of Instances 234  === Confusion Matrix ===  a b c <-- classified as  71 2 5 \| a = HIGH  62 11 5 \| b = INTERMEDIATE  60 5 13 \| c = LOW |
| JR | JRIP rules:  ===========  (PA.101 = Eb) and (PA.204 = Rb) and (PA.400 = Sb) => Vir_three_classes=LOW (20.0/7.0)  (PA.100 = Ab) and (PA.225 = Cb) => Vir_three_classes=LOW (18.0/7.0)  => Vir_three_classes=HIGH (196.0/122.0)  Number of Rules : 3 | === Summary ===  Correctly Classified Instances 98 41.8803 %  Incorrectly Classified Instances 136 58.1197 %  Kappa statistic 0.1282  Mean absolute error 0.4248  Root mean squared error 0.4609  Relative absolute error 95.5859 %  Root relative squared error 97.7681 %  Total Number of Instances 234  === Confusion Matrix ===  a b c <-- classified as  74 0 4 \| a = HIGH  68 0 10 \| b = INTERMEDIATE  54 0 24 \| c = LOW |
| PT | PART decision list  ------------------  PA.399 = Eb AND  PA.2 = Eb AND  PA.298 = Eb AND  PA.343 = Eb AND  PA.327 = Gb AND  PA.505 = Ib: LOW (4.0/1.0)  PA.399 = Kb: LOW (4.0)  PA.2 = Eb AND  PA.298 = Eb AND  PA.379 = Vb AND  PA.627 = Gb AND  PA.343 = Eb AND  PA.327 = Eb: LOW (3.0)  PA.2 = Eb AND  PA.298 = Eb AND  PA.226 = Lb AND  PA.343 = Ab AND  PA.379 = Vb AND  PA.627 = Gb AND  PA.630 = Eb AND  PA.315 = Fb AND  PA.22 = Rb AND  PA.97 = Ib: HIGH (6.0)  PA.22 = Kb AND  PA.2 = Eb AND  PA.298 = Eb AND  PA.226 = Lb AND  PA.379 = Vb AND  PA.627 = Gb AND  PA.343 = Ab AND  PA.630 = Eb AND  PA.315 = Fb AND  PA.556 = Qb AND  PA.97 = Tb AND  PA.557 = Vb AND  PA.668 = Ib AND  PA.321 = Nb AND  PA.59 = Eb AND  PA.261 = Lb AND  PA.142 = Kb AND  PA.5 = Vb AND  PA.465 = Ib AND  PA.149 = Sb AND  PA.127 = Vb AND  PA.28 = Pb AND  PA.664 = Kb AND  PA.535 = Hb AND  PA.272 = Db AND  PA.337 = Ab AND  PA.118 = Ib AND  PA.421 = Sb AND  PA.129 = Ib AND  PA.479 = Db AND  PA.275 = Lb AND  PA.581 = Mb: HIGH (21.0/12.0)  PA.22 = Kb AND  PA.379 = Vb AND  PA.627 = Gb AND  PA.2 = Eb AND  PA.298 = Eb AND  PA.226 = Lb AND  PA.343 = Ab AND  PA.630 = Eb AND  PA.315 = Fb AND  PA.556 = Qb AND  PA.97 = Tb AND  PA.557 = Vb AND  PA.668 = Ib AND  PA.321 = Nb AND  PA.59 = Eb AND  PA.127 = Vb AND  PA.261 = Lb AND  PA.142 = Kb AND  PA.149 = Sb AND  PA.465 = Ib AND  PA.5 = Vb AND  PA.28 = Pb AND  PA.535 = Hb AND  PA.274 = Pb AND  PA.421 = Sb AND  PA.479 = Db AND  PA.664 = Kb: INTERMEDIATE (60.0/33.0)  PA.505 = Ib AND  PA.2 = Eb AND  PA.298 = Eb AND  PA.22 = Kb AND  PA.216 = Db AND  PA.343 = Ab AND  PA.183 = Ab AND  PA.315 = Fb AND  PA.5 = Mb: INTERMEDIATE (8.0/2.0)  PA.505 = Ib AND  PA.2 = Eb AND  PA.298 = Eb AND  PA.343 = Ab AND  PA.22 = Kb AND  PA.216 = Db AND  PA.183 = Ab AND  PA.315 = Fb AND  PA.208 = Tb AND  PA.668 = Ib AND  PA.321 = Nb AND  PA.341 = Vb AND  PA.356 = Rb AND  PA.85 = Tb: INTERMEDIATE (7.0/3.0)  PA.505 = Ib AND  PA.2 = Eb AND  PA.298 = Eb AND  PA.343 = Ab AND  PA.22 = Kb AND  PA.216 = Db AND  PA.183 = Ab AND  PA.315 = Fb AND  PA.208 = Tb AND  PA.465 = Ib AND  PA.63 = Vb AND  PA.287 = Ab AND  PA.149 = Sb AND  PA.204 = Rb AND  PA.668 = Ib AND  PA.379 = Vb AND  PA.556 = Qb AND  PA.90 = Vb: HIGH (45.0/20.0)  PA.59 = Eb AND  PA.627 = Gb AND  PA.550 = Lb AND  PA.22 = Kb AND  PA.183 = Ab AND  PA.315 = Fb AND  PA.208 = Tb AND  PA.90 = Vb AND  PA.241 = Cb AND  PA.321 = Nb AND  PA.2 = Eb AND  PA.298 = Eb AND  PA.216 = Db AND  PA.118 = Ib AND  PA.142 = Kb AND  PA.85 = Tb AND  PA.465 = Ib AND  PA.58 = Gb: INTERMEDIATE (7.0/3.0)  PA.59 = Eb AND  PA.627 = Gb AND  PA.550 = Lb AND  PA.22 = Kb AND  PA.129 = Tb: HIGH (11.0/5.0)  PA.129 = Ib AND  PA.186 = Sb AND  PA.321 = Nb: INTERMEDIATE (7.0/2.0)  PA.129 = Ib AND  PA.186 = Gb AND  PA.352 = Eb AND  PA.226 = Lb AND  PA.272 = Db AND  PA.557 = Vb AND  PA.22 = Kb AND  PA.101 = Eb: LOW (20.0/7.0)  PA.129 = Ib AND  PA.186 = Gb AND  PA.213 = Rb AND  PA.22 = Kb AND  PA.557 = Vb: INTERMEDIATE (11.0/1.0)  PA.129 = Ib AND  PA.186 = Gb: LOW (12.0/3.0)  : HIGH (8.0)  Number of Rules : 16 | === Summary ===  Correctly Classified Instances 97 41.453 %  Incorrectly Classified Instances 137 58.547 %  Kappa statistic 0.1218  Mean absolute error 0.4013  Root mean squared error 0.5163  Relative absolute error 90.2871 %  Root relative squared error 109.5223 %  Total Number of Instances 234  === Confusion Matrix ===  a b c <-- classified as  15 14 49 \| a = HIGH  7 20 51 \| b = INTERMEDIATE  3 13 62 \| c = LOW |
