# Supplementary material for: Rule-based meta-analysis reveals the major role of PB2 in influencing influenza A virus virulence in mice
Source: BMC Genomics. 2019 Dec 24;20(Suppl 9):973. doi: 10.1186/s12864-019-6295-8 (PMC6929465; doi:10.1186/s12864-019-6295-8)
Supplement: Supplementary file 16 — Additional file 16: Table S12. Examples of rules generated by OneR, JRip and PART for two-class and three-class C57BL/6 datasets containing concatenated alignments of IAV proteins. [file 12864_2019_6295_MOESM16_ESM.docx]

**Table S12.** Examples of rules generated by OneR (1R), JRip (JR) and PART (PT) for (A) two-class and (B) three-class C57BL/6 datasets containing concatenated alignments of IAV proteins. For the values of the predictor or protein site (displayed as [protein name].[position]), the first letter indicates the amino acid or gap presents at the site and the second letter c indicates the mouse strain C57BL/6.

(A) Two-class C57BL/6 dataset

| **Method** | **Rule(s)** | **Summary** |
| --- | --- | --- |
| 1R | NS2.63:  Ec -> Virulent  Gc -> Avirulent  (36/44 instances correct) | === Summary ===  Correctly Classified Instances 36 81.8182 %  Incorrectly Classified Instances 8 18.1818 %  Kappa statistic 0.6364  Mean absolute error 0.1818  Root mean squared error 0.4264  Relative absolute error 36.3636 %  Root relative squared error 85.2803 %  Total Number of Instances 44  === Confusion Matrix ===  a b <-- classified as  19 3 \| a = Avirulent  5 17 \| b = Virulent |
| JR | JRIP rules:  ===========  (NS2.63 = Gc) => Vir_two_classes=Avirulent (24.0/5.0)  => Vir_two_classes=Virulent (20.0/3.0)  Number of Rules : 2 | === Summary ===  Correctly Classified Instances 36 81.8182 %  Incorrectly Classified Instances 8 18.1818 %  Kappa statistic 0.6364  Mean absolute error 0.2958  Root mean squared error 0.3846  Relative absolute error 59.1667 %  Root relative squared error 76.9199 %  Total Number of Instances 44  === Confusion Matrix ===  a b <-- classified as  19 3 \| a = Avirulent  5 17 \| b = Virulent |
| PT | PART decision list  ------------------  NS2.63 = Ec: Virulent (20.0/3.0)  NS2.14 = Lc: Avirulent (9.0)  NS2.49 = Lc: Avirulent (4.0)  : Avirulent (11.0/5.0)  Number of Rules : 4 | === Summary ===  Correctly Classified Instances 36 81.8182 %  Incorrectly Classified Instances 8 18.1818 %  Kappa statistic 0.6364  Mean absolute error 0.2192  Root mean squared error 0.3945  Relative absolute error 43.843 %  Root relative squared error 78.8988 %  Total Number of Instances 44  === Confusion Matrix ===  a b <-- classified as  19 3 \| a = Avirulent  5 17 \| b = Virulent |

(B) Three-class C57BL/6 dataset

| **Method** | **Rule(s)** | **Summary** |
| --- | --- | --- |
| 1R | HA..12:  Ac -> INTERMEDIATE  Ec -> HIGH  Ic -> LOW  Qc -> HIGH  Vc -> LOW  (39/54 instances correct) | === Summary ===  Correctly Classified Instances 35 64.8148 %  Incorrectly Classified Instances 19 35.1852 %  Kappa statistic 0.4722  Mean absolute error 0.2346  Root mean squared error 0.4843  Relative absolute error 52.7778 %  Root relative squared error 102.7402 %  Total Number of Instances 54  === Confusion Matrix ===  a b c <-- classified as  11 6 1 \| a = HIGH  1 16 1 \| b = INTERMEDIATE  5 5 8 \| c = LOW |
| JR | JRIP rules:  ===========  (HA.384 = Ic) => Vir_three_classes=HIGH (14.0/1.0)  (PA.388 = Gc) => Vir_three_classes=INTERMEDIATE (26.0/9.0)  => Vir_three_classes=LOW (14.0/1.0)  Number of Rules : 3 | === Summary ===  Correctly Classified Instances 43 79.6296 %  Incorrectly Classified Instances 11 20.3704 %  Kappa statistic 0.6944  Mean absolute error 0.2101  Root mean squared error 0.3242  Relative absolute error 47.2833 %  Root relative squared error 68.7628 %  Total Number of Instances 54  === Confusion Matrix ===  a b c <-- classified as  13 5 0 \| a = HIGH  0 17 1 \| b = INTERMEDIATE  1 4 13 \| c = LOW |
| PT | PART decision list  ------------------  HA.384 = Vc AND  NS1.178 = Vc AND  NA.25 = Qc AND  HA.294 = Fc: INTERMEDIATE (17.0/2.0)  HA..5 = Cc AND  PB2.504 = Vc: LOW (19.0/2.0)  : HIGH (18.0/3.0)  Number of Rules : 3 | === Summary ===  Correctly Classified Instances 33 61.1111 %  Incorrectly Classified Instances 21 38.8889 %  Kappa statistic 0.4167  Mean absolute error 0.2919  Root mean squared error 0.4583  Relative absolute error 65.6839 %  Root relative squared error 97.2271 %  Total Number of Instances 54  === Confusion Matrix ===  a b c <-- classified as  16 0 2 \| a = HIGH  12 0 6 \| b = INTERMEDIATE   1. 0 17 \| c = LOW |
