# Supplementary material for: Rule-based meta-analysis reveals the major role of PB2 in influencing influenza A virus virulence in mice
Source: BMC Genomics. 2019 Dec 24;20(Suppl 9):973. doi: 10.1186/s12864-019-6295-8 (PMC6929465; doi:10.1186/s12864-019-6295-8)
Supplement: Supplementary file 17 — Additional file 17: Table S13. Examples of rules generated by OneR, JRip and PART for two-class and three-class H1N1 datasets containing concatenated alignments of IAV proteins. [file 12864_2019_6295_MOESM17_ESM.docx]

**Table S13.** Examples of rules generated by OneR (1R), JRip (JR) and PART (PT) for (A) two-class and (B) three-class H1N1 datasets containing concatenated alignments of IAV proteins. The predictor or protein site is displayed as [protein name].[position].

(A) Two-class H1N1 dataset

| **Method** | **Rule(s)** | **Summary** |
| --- | --- | --- |
| 1R | NS2.107:  F -> Avirulent  L -> Virulent  (45/68 instances correct) | === Summary ===  Correctly Classified Instances 45 66.1765 %  Incorrectly Classified Instances 23 33.8235 %  Kappa statistic 0.3235  Mean absolute error 0.3382  Root mean squared error 0.5816  Relative absolute error 67.6471 %  Root relative squared error 116.316 %  Total Number of Instances 68  === Confusion Matrix ===  a b <-- classified as  12 22 \| a = Avirulent  1 33 \| b = Virulent |
| JR | JRIP rules:  ===========  (NS2.107 = F) => Vir_two_classes=Avirulent (13.0/1.0)  => Vir_two_classes=Virulent (55.0/22.0)  Number of Rules : 2 | === Summary ===  Correctly Classified Instances 45 66.1765 %  Incorrectly Classified Instances 23 33.8235 %  Kappa statistic 0.3235  Mean absolute error 0.4154  Root mean squared error 0.4557  Relative absolute error 83.0769 %  Root relative squared error 91.1465 %  Total Number of Instances 68  === Confusion Matrix ===  a b <-- classified as  12 22 \| a = Avirulent  1 33 \| b = Virulent |
| PT | PART decision list  ------------------  NS2.107 = L AND  NS2.3 = P: Virulent (14.0/2.0)  NS2.107 = F: Avirulent (13.0/1.0)  NS2.89 = I AND  NS2.60 = N: Virulent (6.0/2.0)  NS2.57 = Y: Virulent (31.0/14.0)  : Avirulent (4.0)  Number of Rules : 5 | === Summary ===  Correctly Classified Instances 49 72.0588 %  Incorrectly Classified Instances 19 27.9412 %  Kappa statistic 0.4412  Mean absolute error 0.3426  Root mean squared error 0.4139  Relative absolute error 68.5183 %  Root relative squared error 82.7758 %  Total Number of Instances 68  === Confusion Matrix ===  a b <-- classified as  16 18 \| a = Avirulent  1 33 \| b = Virulent |

(B) Three-class H1N1 dataset

| **Method** | **Rule(s)** | **Summary** |
| --- | --- | --- |
| 1R | PA.277:  F -> Low  H -> Intermediate  S -> High  Y -> Low  (45/87 instances correct) | === Summary ===  Correctly Classified Instances 45 51.7241 %  Incorrectly Classified Instances 42 48.2759 %  Kappa statistic 0.2759  Mean absolute error 0.3218  Root mean squared error 0.5673  Relative absolute error 72.4138 %  Root relative squared error 120.3443 %  Total Number of Instances 87  === Confusion Matrix ===  a b c <-- classified as  18 11 0 \| a = High  9 17 3 \| b = Intermediate  6 13 10 \| c = Low |
| JR | JRIP rules:  ===========  (NA.108 = T) => Vir_three_classes=Low (18.0/6.0)  (PB2.82 = S) => Vir_three_classes=Low (4.0/0.0)  => Vir_three_classes=High (65.0/38.0)  Number of Rules : 3 | === Summary ===  Correctly Classified Instances 43 49.4253 %  Incorrectly Classified Instances 44 50.5747 %  Kappa statistic 0.2414  Mean absolute error 0.3867  Root mean squared error 0.4397  Relative absolute error 86.9968 %  Root relative squared error 93.2721 %  Total Number of Instances 87  === Confusion Matrix ===  a b c <-- classified as  27 0 2 \| a = High  25 0 4 \| b = Intermediate  13 0 16 \| c = Low |
| PT | PART decision list  ------------------  PB2.108 = A: High (5.0)  HA.453 = S AND  PB1.105 = N AND  NA.330 = S AND  M2.24 = D AND  NA.273 = G AND  HA.75 = E AND  PB2.215 = T AND  PB2.443 = K AND  NP.133 = I: Intermediate (4.0)  HA.453 = S AND  PB1.105 = N AND  NA.330 = S AND  M2.24 = D AND  PB1.391 = K AND  HA.113 = S AND  HA.383 = S AND  PA.529 = D AND  PB2.158 = E AND  NS1.101 = D AND  PB2.251 = R AND  HA.190 = D AND  HA.103 = A: Low (10.0)  HA.453 = S AND  PB1.105 = N AND  PA.688 = E AND  M2.24 = D AND  PB1.391 = K AND  HA.113 = S AND  NS2.22 = E: High (8.0/1.0)  HA.453 = S AND  PB1.105 = N AND  PA.688 = E AND  M2.24 = D AND  NS2.3 = P: Intermediate (7.0)  HA.453 = S AND  PB2.701 = D AND  PB1.105 = N AND  M2.24 = D AND  HA.420 = I AND  HA.225 = G AND  HA.186 = P: High (4.0)  HA.453 = S AND  PB2.701 = D AND  PB1.105 = N AND  M2.24 = D AND  PB2.344 = V AND  PB2.187 = K AND  NA.451 = I AND  NA.300 = H AND  NA.32 = I AND  HA.225 = D AND  HA.186 = S AND  PA.529 = D AND  PB2.82 = N AND  PB1.175 = N AND  PB1.353 = K: Low (8.0/2.0)  HA.453 = S AND  PB2.701 = D AND  PB1.105 = N AND  M2.24 = D AND  HA.420 = I AND  NA.300 = H AND  NA.451 = I AND  PA.581 = M AND  HA.165 = S: Intermediate (15.0/2.0)  PB1.53 = G AND  PB2.82 = N AND  NA.300 = H AND  NA.376 = N: High (15.0/2.0)  : Low (11.0/1.0)  Number of Rules : 10 | === Summary ===  Correctly Classified Instances 69 79.3103 %  Incorrectly Classified Instances 18 20.6897 %  Kappa statistic 0.6897  Mean absolute error 0.1769  Root mean squared error 0.3356  Relative absolute error 39.7962 %  Root relative squared error 71.1988 %  Total Number of Instances 87  === Confusion Matrix ===  a b c <-- classified as  29 0 0 \| a = High  5 20 4 \| b = Intermediate  4 5 20 \| c = Low |
