# Supplementary material for: Rule-based meta-analysis reveals the major role of PB2 in influencing influenza A virus virulence in mice
Source: BMC Genomics. 2019 Dec 24;20(Suppl 9):973. doi: 10.1186/s12864-019-6295-8 (PMC6929465; doi:10.1186/s12864-019-6295-8)
Supplement: Supplementary file 18 — Additional file 18: Table S14. Examples of rules generated by OneR, JRip and PART for two-class and three-class H3N2 datasets containing concatenated alignments of IAV proteins. [file 12864_2019_6295_MOESM18_ESM.docx]

**Table S14.** Examples of rules generated by OneR (1R), JRip (JR) and PART (PT) for (A) two-class and (B) three-class H3N2 datasets containing concatenated alignments of IAV proteins. The predictor or protein site is displayed as [protein name].[position].

(A) Two-class H3N2 dataset

| **Method** | **Rule(s)** | **Summary** |
| --- | --- | --- |
| 1R | NP.18:  D -> Avirulent  E -> Virulent  G -> Avirulent  (20/28 instances correct) | === Summary ===  Correctly Classified Instances 20 71.4286 %  Incorrectly Classified Instances 8 28.5714 %  Kappa statistic 0.4286  Mean absolute error 0.2857  Root mean squared error 0.5345  Relative absolute error 57.1429 %  Root relative squared error 106.9045 %  Total Number of Instances 28  === Confusion Matrix ===  a b <-- classified as  6 8 \| a = Avirulent  0 14 \| b = Virulent |
| JR | JRIP rules:  ===========  (NP.34 = N) => Vir_two_classes=Virulent (6.0/1.0)  (NP.450 = S) => Vir_two_classes=Virulent (7.0/1.0)  => Vir_two_classes=Avirulent (15.0/3.0)  Number of Rules : 3 | === Summary ===  Correctly Classified Instances 23 82.1429 %  Incorrectly Classified Instances 5 17.8571 %  Kappa statistic 0.6429  Mean absolute error 0.2922  Root mean squared error 0.3822  Relative absolute error 58.4354 %  Root relative squared error 76.443 %  Total Number of Instances 28  === Confusion Matrix ===  a b <-- classified as  12 2 \| a = Avirulent  3 11 \| b = Virulent |
| PT | PART decision list  ------------------  NP.65 = R AND  NP.421 = D AND  NP.34 = D: Avirulent (10.0/3.0)  NP.18 = E: Virulent (12.0/1.0)  : Avirulent (6.0)  Number of Rules : 3 | === Summary ===  Correctly Classified Instances 24 85.7143 %  Incorrectly Classified Instances 4 14.2857 %  Kappa statistic 0.7143  Mean absolute error 0.2155  Root mean squared error 0.3282  Relative absolute error 43.0952 %  Root relative squared error 65.647 %  Total Number of Instances 28  === Confusion Matrix ===  a b <-- classified as  13 1 \| a = Avirulent  3 11 \| b = Virulent |

(B) Three-class H3N2 dataset

| **Method** | **Rule(s)** | **Summary** |
| --- | --- | --- |
| 1R | NP.34:  D -> LOW  G -> HIGH  N -> HIGH  (13/21 instances correct) | === Summary ===  Correctly Classified Instances 13 61.9048 %  Incorrectly Classified Instances 8 38.0952 %  Kappa statistic 0.4286  Mean absolute error 0.254  Root mean squared error 0.504  Relative absolute error 57.1429 %  Root relative squared error 106.9045 %  Total Number of Instances 21  === Confusion Matrix ===  a b c <-- classified as  7 0 0 \| a = HIGH  3 0 4 \| b = INTERMEDIATE  1 0 6 \| c = LOW |
| JR | JRIP rules:  ===========  (NP.34 = N) => Vir_three_classes=HIGH (7.0/3.0)  (NP.34 = G) => Vir_three_classes=HIGH (4.0/1.0)  => Vir_three_classes=LOW (10.0/4.0)  Number of Rules : 3 | === Summary ===  Correctly Classified Instances 13 61.9048 %  Incorrectly Classified Instances 8 38.0952 %  Kappa statistic 0.4286  Mean absolute error 0.3088  Root mean squared error 0.393  Relative absolute error 69.4898 %  Root relative squared error 83.3605 %  Total Number of Instances 21  === Confusion Matrix ===  a b c <-- classified as  7 0 0 \| a = HIGH  3 0 4 \| b = INTERMEDIATE  1 0 6 \| c = LOW |
| PT | PART decision list  ------------------  NP.65 = R AND  NP.34 = D: INTERMEDIATE (7.0/3.0)  NP.65 = R: HIGH (11.0/4.0)  : LOW (3.0)  Number of Rules : 3 | === Summary ===  Correctly Classified Instances 14 66.6667 %  Incorrectly Classified Instances 7 33.3333 %  Kappa statistic 0.5  Mean absolute error 0.2878  Root mean squared error 0.3793  Relative absolute error 64.7495 %  Root relative squared error 80.4671 %  Total Number of Instances 21  === Confusion Matrix ===  a b c <-- classified as  7 0 0 \| a = HIGH  3 4 0 \| b = INTERMEDIATE  1 3 3 \| c = LOW |
