# Supplementary material for: Rule-based meta-analysis reveals the major role of PB2 in influencing influenza A virus virulence in mice
Source: BMC Genomics. 2019 Dec 24;20(Suppl 9):973. doi: 10.1186/s12864-019-6295-8 (PMC6929465; doi:10.1186/s12864-019-6295-8)
Supplement: Supplementary file 19 — Additional file 19: Table S15. Examples of rules generated by OneR, JRip and PART for two-class and three-class H5N1 datasets containing concatenated alignments of IAV proteins. [file 12864_2019_6295_MOESM19_ESM.docx]

**Table S15.** Examples of rules generated by OneR (1R), JRip (JR) and PART (PT) for (A) two-class and (B) three-class H5N1 datasets containing concatenated alignments of IAV proteins. The predictor or protein site is displayed as [protein name].[position].

(A) Two-class H5N1 dataset

| **Method** | **Rule(s)** | **Summary** |
| --- | --- | --- |
| 1R | NS2.22:  A -> Virulent  E -> Virulent  G -> Avirulent  (39/64 instances correct) | === Summary ===  Correctly Classified Instances 39 60.9375 %  Incorrectly Classified Instances 25 39.0625 %  Kappa statistic 0.2188  Mean absolute error 0.3906  Root mean squared error 0.625  Relative absolute error 78.125 %  Root relative squared error 125 %  Total Number of Instances 64  === Confusion Matrix ===  a b <-- classified as  29 3 \| a = Avirulent  22 10 \| b = Virulent |
| JR | JRIP rules:  ===========  (NS2.88 = K) and (NS2.52 = M) => Vir_two_classes=Virulent (18.0/3.0)  => Vir_two_classes=Avirulent (46.0/17.0)  Number of Rules : 2 | === Summary ===  Correctly Classified Instances 27 42.1875 %  Incorrectly Classified Instances 37 57.8125 %  Kappa statistic -0.1563  Mean absolute error 0.5362  Root mean squared error 0.5755  Relative absolute error 107.2464 %  Root relative squared error 115.0966 %  Total Number of Instances 64  === Confusion Matrix ===  a b <-- classified as  20 12 \| a = Avirulent  25 7 \| b = Virulent |
| PT | PART decision list  ------------------  NS2.55 = L AND  NS2.26 = E AND  NS2.31 = M: Avirulent (45.0/17.0)  : Virulent (19.0/4.0)  Number of Rules : 2 | === Summary ===  Correctly Classified Instances 43 67.1875 %  Incorrectly Classified Instances 21 32.8125 %  Kappa statistic 0.3438  Mean absolute error 0.4292  Root mean squared error 0.4633  Relative absolute error 85.848 %  Root relative squared error 92.6542 %  Total Number of Instances 64  === Confusion Matrix ===  a b <-- classified as  28 4 \| a = Avirulent  17 15 \| b = Virulent |

(B) Three-class H5N1 dataset

| **Method** | **Rule(s)** | **Summary** |
| --- | --- | --- |
| 1R | M2.28:  - -> INTERMEDIATE  I -> INTERMEDIATE  V -> HIGH  (34/66 instances correct) | === Summary ===  Correctly Classified Instances 34 51.5152 %  Incorrectly Classified Instances 32 48.4848 %  Kappa statistic 0.2727  Mean absolute error 0.3232  Root mean squared error 0.5685  Relative absolute error 72.7273 %  Root relative squared error 120.6045 %  Total Number of Instances 66  === Confusion Matrix ===  a b c <-- classified as  19 3 0 \| a = HIGH  7 15 0 \| b = INTERMEDIATE  14 8 0 \| c = LOW |
| JR | JRIP rules:  ===========  (M2.28 = I) => Vir_three_classes=INTERMEDIATE (8.0/1.0)  (NS1.42 = P) => Vir_three_classes=LOW (5.0/0.0)  (NA.247 = T) => Vir_three_classes=LOW (13.0/5.0)  => Vir_three_classes=HIGH (40.0/20.0)  Number of Rules : 4 | === Summary ===  Correctly Classified Instances 23 34.8485 %  Incorrectly Classified Instances 43 65.1515 %  Kappa statistic 0.0227  Mean absolute error 0.4318  Root mean squared error 0.6382  Relative absolute error 97.1591 %  Root relative squared error 135.3867 %  Total Number of Instances 66  === Confusion Matrix ===  a b c <-- classified as  0 0 22 \| a = HIGH  0 7 15 \| b = INTERMEDIATE  5 1 16 \| c = LOW |
| PT | PART decision list  ------------------  NS1.42 = S AND  PB2.153 = D AND  PB2.185 = I AND  PA.364 = S AND  NA.417 = S AND  PB1-F2.81 = K AND  PB2.627 = K: HIGH (10.0/1.0)  PA.142 = K AND  NP.63 = I AND  PB2.630 = R AND  PB2.607 = L AND  PB1.327 = R AND  NA.34 = V AND  PB2.105 = T AND  NP.236 = K AND  PB2.701 = D AND  HA.227 = S AND  M1.30 = D: INTERMEDIATE (10.0/4.0)  PA.142 = K AND  M1.205 = I: INTERMEDIATE (5.0)  NP.372 = D: INTERMEDIATE (3.0)  NA.225 = G AND  PA.142 = K AND  PB2.318 = R AND  PB2.635 = P AND  M1.215 = A: HIGH (3.0)  PB2.635 = S AND  NA.225 = G AND  M2.43 = - AND  M1.215 = T: LOW (4.0)  PB2.635 = S AND  NA.225 = G AND  M2.43 = - AND  PB2.108 = A: LOW (3.0)  M2.25 = P AND  NA.225 = G AND  PB2.368 = R AND  NS1.42 = S AND  HA.101 = N AND  PB2.318 = R: LOW (7.0/1.0)  NS1.42 = S AND  M2.25 = P AND  NA.225 = G: HIGH (10.0/1.0)  NS1.42 = S: INTERMEDIATE (6.0)  : LOW (5.0)  Number of Rules : 11 | === Summary ===  Correctly Classified Instances 25 37.8788 %  Incorrectly Classified Instances 41 62.1212 %  Kappa statistic 0.0682  Mean absolute error 0.4141  Root mean squared error 0.6435  Relative absolute error 93.1818 %  Root relative squared error 136.5151 %  Total Number of Instances 66  === Confusion Matrix ===  a b c <-- classified as  0 1 21 \| a = HIGH  0 3 19 \| b = INTERMEDIATE  0 0 22 \| c = LOW |
